# Metabolome and transcriptome associated analysis of sesquiterpenoid metabolism in *Nardostachys jatamansi*

**DOI:** 10.3389/fpls.2022.1041321

**Published:** 2022-11-29

**Authors:** Mingkang Feng, Chen Chen, Junzhang Qu-Bie, Axiang Qu-Bie, Xiaoming Bao, Qi Cui, Xinjia Yan, Ying Li, Yuan Liu, Shaoshan Zhang

**Affiliations:** ^1^ Tibetan Plateau Ethnic Medicinal Resources Protection and Utilization Key Laboratory of National Ethnic Affairs Commission of the People's Republic of China, Southwest Minzu University, Chengdu, China; ^2^ Sichuan Provincial Qiang-Yi Medicinal Resources Protection and Utilization Technology and Engineering Laboratory, Southwest Minzu University, Chengdu, China; ^3^ College of Pharmacy, Southwest Minzu University, Chengdu, China; ^4^ Analysis and Application Center, Shimadzu (China) Co., Ltd, Chengdu, China

**Keywords:** *Nardostachys jatamansi*, sesquiterpenoid, transcriptome sequencing, biosynthesis and accumulation, terpene synthase, cytochrome P450

## Abstract

**Background:**

Nardostachys jatamansi, an extremely endangered valuable plant of the alpine Himalayas, can synthesize specific sesquiterpenoids with multiple effective therapies and is widely exploited for the preparation of drugs, cosmetics and even religious functions (e.g., well-known spikenard). However, how accumulation trend of the sesquiterpenoids in tissues and the molecular mechanisms underlying the production of the active ingredients are not well understood.

**Methods:**

The single-molecule real-time (SMRT) and RNA-seq transcriptome sequencing were combined to analyse the roots, rhizomes, leaves, flowers and anthocaulus of N. jatamansi. The phytochemical analysis was performed by gas chromatography‒mass spectrometry (GC‒MS) and ultrahigh-performance liquid chromatography (UPLC).

**Results:**

A high-quality full-length reference transcriptome with 26,503 unigenes was generated for the first time. For volatile components, a total of sixty-five compounds were successfully identified, including fifty sesquiterpenoids. Their accumulation levels in five tissues were significantly varied, and most of the sesquiterpenoids were mainly enriched in roots and rhizomes. In addition, five aromatic compounds were only detected in flowers, which may help the plant attract insects for pollination. For nonvolatile ingredients, nardosinone-type sesquiterpenoids (nardosinone, kanshone C, and isonardosinone) were detected almost exclusively in roots and rhizomes. The candidate genes associated with sesquiterpenoid biosynthesis were identified by transcriptome analysis. Consistently, it was found that most biosynthesis genes were abundantly expressed in the roots and rhizomes according to the functional enrichment and expression patterns results. There was a positive correlation between the expression profile of genes related to the biosynthesis and the accumulation level of sesquiterpenoids in tissues. Gene family function analysis identified 28 NjTPSs and 43 NjCYPs that may be involved in the biosynthesis of the corresponding sesquiterpenoids. Furthermore, gene family functional analysis and gene coexpression network analysis revealed 28 NjTPSs and 43 NjCYPs associated with nardosinone-type sesquiterpenoid biosynthesis.

**Conclusion:**

Our research results reveal the framework of sesquiterpenoids accumulation and biosynthesis in plant tissues and provide valuable support for further studies to elucidate the molecular mechanisms of sesquiterpenoid regulation and accumulation in N. jatamansi and will also contribute to the comprehensive utilization of this alpine plant.

## Introduction

The Himalayas, with deep valleys, high mountains, lowland plains, and the influence of summer monsoons and winter droughts, contribute to an extremely diverse ecosystem, including fauna and a great diversity of medicinal plants. Statistics show that approximately 1,748 medicinal plants were reported from the Indian Himalayan region and Chinese Himalayan region (Tibet, Qinghai, Gansu, Yunnan, and Sichuan) (Chauhan et al., 2021). Because of the safety and lack of side effects, botanical herbs have always been important as therapeutics to treat diseases, and their market is witnessing an ever-increasing demand worldwide (Chen et al., 2016; Kumar and Van Staden, 2016). This kind of demand is particularly strong for residents near the Himalayas due to the relatively backwards economy and technology. To date, a lot of Himalayan medicinal plants have been utilized by the pharmaceutical, nutraceutical and cosmetic industries, which has led many plants to become threatened in the region (Mehta et al., 2020). Nonetheless, of the rich and diverse flora of the Himalayas, only a few have been scientifically studied and fully exploited in the herbal market, mainly because of the unavailability of scientific knowledge (Dhiman and Bhattacharya, 2019).

*Nardostachys jatamansi* is one such Himalayan plant (located in alpine and subalpine areas, 3,200 to 4,800 m) that has a long history of great therapeutic use in Chinese, Tibetan, Japanese, Indian and other traditional systems (Sharma et al., 2000; Chen and Mukerji, 2013; Dhiman and Bhattacharya, 2019). The roots and rhizomes of *N. jatamansi* have been reported to treat various diseases, such as nervous and digestive disorders, and to cure headache disorder, memory loss, mental disorders, heart palpitations, nerve and cardiac diseases, and so on (Rokaya et al., 2010; Dhiman and Bhattacharya, 2019; Kaur et al., 2020). Surprisingly, *N. jatamansi* also has extensive application in religious activities in northern India and Tibetan areas in western China (Kala et al., 2006). Only in India is the annual demand for rhizomes of the plant approximately 500-1,000 metric tons (https://www.nmpb.nic.in/medicinal_list). Because of the huge market demand as well as the low germination rate and short growth period, *N. jatamansi* is now classified as a critically endangered species by the International Union for Conservation of Nature (IUCN) and is also listed in the ‘Convention on International Trade in Endangered Species of Wild Fauna and Flora, (CITES)’ (Nisha et al., 2020; Chauhan et al., 2021). Therefore, alternative approaches aimed at protecting the precious alpine perennial herb are a major priority for scientists.

To date, numerous compounds with a wide range of biological properties have been isolated and identified from *N. jatamansi*, such as lignans, alkaloids, neolignans, coumarins, and sesquiterpenoids (Kaur et al., 2020; Nisha et al., 2020). Of these numerous compounds, the sesquiterpenoids are the major chemical constituents of roots and rhizomes of the plant, which are considered to be directly related to different medicinal purposes (Kaur et al., 2020; Nisha et al., 2020). The essential oils extracted from the roots and rhizomes were extensively exploited for the preparation of drugs and cosmetics, such as spikenard essential oil, which is popular around the world (Paudyal et al., 2012). A number of sesquiterpenoids have been successfully isolated and well identified from essential oils, including α/β/γ-patchoulene, calarene, valerenol, and seychellene. (Nisha et al., 2020). Among the nonvolatile components, nardosinone-type sesquiterpenoids are the main active ingredients in *N. jatamansi* and play a key role in the pharmacological functions of *N. jatamansi* (Wen et al., 2020). Most of these nardosinone-type sesquiterpenoids are nardosinone, and its accumulation level in *N. jatamansi* accounts for more than 2% (dry weight) (Le et al., 2017). As the most abundant component, nardosinone has a variety of pharmacological functions, including neuroprotective, cardioprotective, anti-inflammatory and other potential pharmacological activities (Wen et al., 2021). Nardosinone is also considered to be the only quality marker compound of *N. jatamansi* medicinal materials in the Chinese Pharmacopoeia (Wen et al., 2020). In addition, these valuable metabolites may help the plant adapt to its specific ecological niche of alpine habitats. Presumably, the biosynthetic pathways and regulatory mechanisms of the valuable metabolites in *N. jatamansi* are led by an underlying complex machinery.

Because of its growth in inaccessible places in the Himalayas, there is little molecular background information available on *N. jatamansi*, especially regarding the biosynthesis pathways of sesquiterpenoids. To date, only one study has made an effort to reveal the secondary metabolite biosynthesis genes in *N. jatamansi*, but concentrated mainly on phenolics (Nisha et al., 2020). With the technological development of biosynthesis and molecular marker-assisted breeding, metabolic engineering and breeding fine varieties may be effective methods for obtaining increased yields of the abovementioned valuable metabolites. Such alternative methods of metabolite production could not only eliminate the need to indiscriminately harvest roots and rhizomes of *N. jatamansi* from wild, but could also halt the decline of natural populations and protect the ecological environment of the Himalayas. However, these strategies largely rely on the elucidation of their biosynthetic pathway, which still has not been characterized. In addition, studies based on phytochemical and molecular analysis whether other tissues can be used as an alternative source for bioactive compounds have not been reported. In the current study, the findings attempt to provide useful information about the accumulation trend and biosynthetic pathway genes of these valuable sesquiterpenoids found in *N. jatamansi*, especially for nardosinone-type sesquiterpenoids. The key regulators characterized in this research will help the production of important metabolites in heterologous systems such as *Escherichia coli* and yeast.

## Results

### Phytochemical analysis

**Figure 1:**
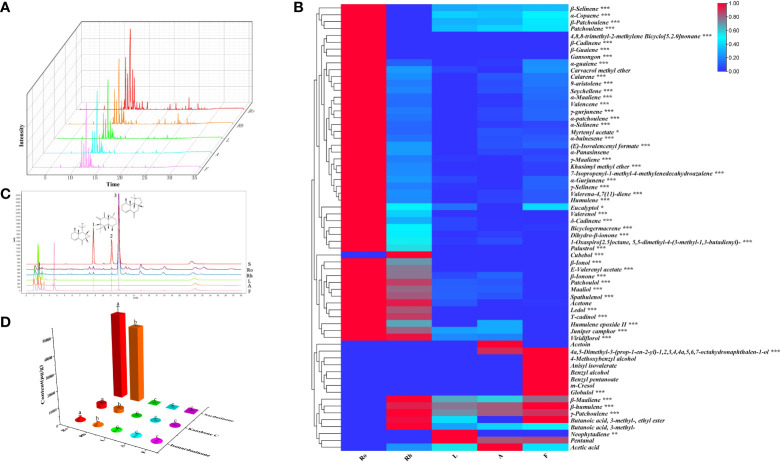
Analysis of phytochemicals in *N. jatamansi*. **(A)** Total ion chromatograms (TIC) of roots (Ro), rhizome (Rh), leaf (L), flower (F), and anthocaulus **(A)**; **(B)** Heat map of volatile components in five tissues; **(C)** UPLC chromatograms of the five tissues (1: isonardosinone, 2: kanshone C, 3: nardosinone). **(D)** Total content of three typical types of nardosinone-type sesquiterpenoids in five tissues.

Previous studies have shown that the major chemical compounds of the roots and rhizomes of *N. jatamansi* are terpenoids. However, the distribution of terpenoids in different tissues in *N. jatamansi* is not very clear. Here, the volatile components in different tissues (roots, rhizomes, leaves, flowers, and anthocaulus) of *N. jatamansi* were analysed by gas chromatography-mass spectrometry (GC-MS). Sixty-five compounds were successfully identified, including fifty sesquiterpenoids (**Table S1**). In the heatmap analysis results, the accumulation levels of these sixty-five compounds in various tissues were significantly different, and most of the terpenoids were mainly enriched in the roots and rhizomes (**Figures 1A, B**). Of these sixty-five compounds, the total amount of β-maaliene, β-cadinene, aristolene, calarene, valerena-4,7(11)-diene, and seychellene accounted for the largest proportion (more than 6%). Eleven sesquiterpenoids were unique to the roots and rhizomes, namely, 4,8,8-trimethyl-2-methylene bicyclo[5.2.0]nonane, β-cadinene, β-guaiene, valerenol, palustrol, cubebol, β-ionol, ledol, T-cadinol, gansongon, and E-valerenyl acetate. Two sesquiterpenoids named 4a,5-dimethyl-3-(prop-1-en-2-yl)-1,2,3,4,4a,5,6,7-octahydronaphthalen-1-ol and globulol, one diterpenoid named neophytadiene, two aliphatics named acetoin and pentanal, five phenolic acids named benzyl alcohol, benzyl pentanoate, m-cresol, 4-methoxybenzyl alcohol, and anisyl isovalerate were unique to the leaves, flowers, and anthocaulus (**Table S1**).

Among nonvolatile components, the concentrations of nardosinone-type sesquiterpenoids were relatively richer than those of other phytochemicals, especially nardosinone. Coincidentally, three nardosinone-type sesquiterpenoids, nardosinone, isonardosinone, and kanshone C, were isolated and identified from *N. jatamansi* in an earlier stage of our research. Consequently, the accurate contents of nardosinone, isonardosinone and kanshone C in different tissues were quantified by ultrahigh-performance liquid chromatography (UPLC). All validation methods used for detecting the three typical types of nardosinone-type sesquiterpenoids from UPLC-UV analysis satisfied the quantitative requirements (**Table S2**). The results indicated that nardosinone was mainly accumulated in the roots and rhizomes but distributed in a small amount in the flowers and could not be detected in the leaves or anthocaulus (**Figures 1C, D**). Isonardosinone was only distributed in the roots and rhizomes, and kanshone C was only detected in the roots.

Generally, if the natural products have obvious tissue or species accumulation specificity, it will greatly help us to mine the genes related to the biosynthesis of those products, because it will allow us to accurately predict the candidate genes through some simple bioinformatics analysis. Interestingly, the sesquiterpenoids in *N. jatamansi* have strong distribution specificity in different tissues, which may be related to the biosynthetic mechanism of plant secondary metabolites.

### Transcriptome sequencing

To obtain the complete transcriptome of *N. jatamansi* and uncover the molecular mechanisms of the valuable metabolites present in different tissues, the Illumina next-generation sequencing (NGS) and PacBio Single Molecule Real-time (SMRT) sequencing platforms were combined to sequence five different tissues of *N. jatamansi*. First, roots, rhizomes, leaves, flowers, and anthocaulus with different kinds and concentrations of the sesquiterpenoids of *N. jatamansi* were selected simultaneously to obtain libraries and then sequenced on Illumina HiSeq X Ten platform with 2 x 150 bp paired-end reads (**Table S3**). A set of 106.5 Gb raw sequencing reads were obtained from 15 libraries. After removing the adapter sequences, low quality and contaminated reads, a total of 102.5 Gb clean reads were generated. More than 94% of the sequences in each library had a quality score above Q30, indicating that the library had high-quality raw reads. After *de novo* assembly using Trinity and clustering using CD-HIT (Li et al., 2001), a set of 82,772 unigenes (average length: 1,169 bp and N50 value: 1,676 bp) were obtained. 59,362 (71.72%) unigenes were >500 bp, and 36,934 (44.62%) unigenes were > 1,000 bp in length (**Figure S1**). The statistical summary of the data is presented in **Table S4**. The Illumina sequencing results in this study were better than those of previous transcriptome sequencing of *N. jatamansi* (Nisha et al., 2020).

Due to the technical limitation, some fragmented or misassembled contigs are often generated after *de novo* transcriptome assembly, which seriously misleads the transcriptome analysis (Conesa et al., 2016). On the contrary, SMRT sequencing technology has longer reads (average 4~8kb) and fewer errors caused by reverse transcription, and is considered to be the most reliable method to obtain full-length cDNA molecules (Chaisson et al., 2014). Thus, the full-length cDNA reads for the pooled RNA from *N. jatamansi* were sequenced on a SMRT platform (PacBio Sequel II), and a number of 13,538,998 raw reads (approximately 57.2 billion bases) were generated. After performing the IsoSeq protocols (Salah et al., 2016), a total of 35,050 high-quality consensus sequences (approximately 158.4 million bases) were obtained, with an average length of 4,520 bp. After removing redundant sequences using CD-HIT (identity = 0.98), 26,503 full-length unigenes (approximately 59.3 million bases) with an average length of 2,237 bp were generated. In addition, 25,610 (96.63%) unigenes were > 1,000 bp, and only 893 (3.37%) unigenes were ≤ 1,000 bp in length (**Figure S2**). Comparison of transcripts obtained from the Illumina and PacBio platforms, it was obvious that the *de novo* assembled transcripts from Illumina short reads resulted in fragmented or misassembled contigs. Therefore, the full-length transcripts obtained by third-generation SMRT sequencing were used as the reference transcriptome for subsequent analysis.

### Gene annotation and functional classification

**Figure 2:**
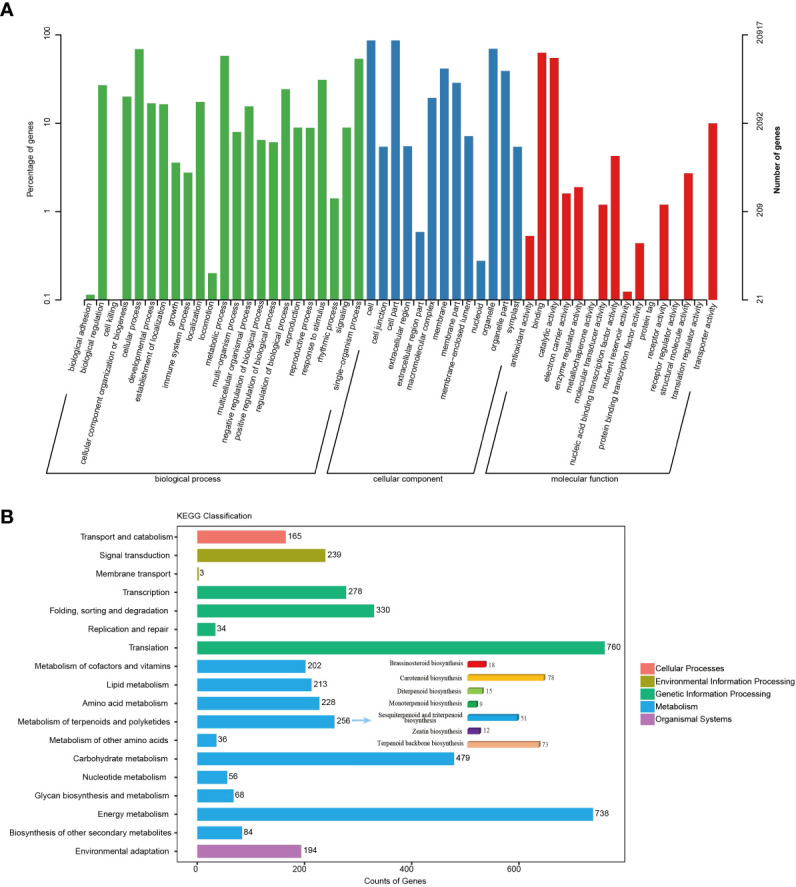
Annotation and classification of transcripts from SMRT sequencing. **(A)** Transcript distribution in GO categories under Biological process, Cellular component and Molecular function. **(B)** KEGG functional classification of unigenes.

The unigenes obtained above were annotated against seven databases, NR, GO, KEGG, SwissProt, Pfam, eggNOG, and KOG, using BLAST searches (**Table S5**). Then, 26,069 (98.36%), 23,333 (88.04%), 4,388 (14.23%), 17,091 (64.49%), 25,639 (96.74%), 20,917 (78.92%), and 24,988 (94.28%) unigenes were significantly matched in NR, Swiss-Prot, KEGG, KOG, eggNOG, GO, and Pfam, respectively. In GO analysis, unigenes related to “metabolic process” from the biological process category and “catalytic activity” from the molecular function category accounted for relatively higher proportions than unigenes related to other GO subcategories (**Figure 2A**). Among the unigenes searched against the KEGG pathway database, there was a large group of unigenes (256) related to “metabolism of terpenoids and polyketides”. Of these unigenes in “metabolism of terpenoids and polyketides”, 124 belong to “sesquiterpenoid and triterpenoid biosynthesis” and “terpenoid backbone biosynthesis”, which was consistent with the richness of sesquiterpenoids in *N. jatamansi* (**Figure 2B**). Notably, “environmental adaptation” was also a large group, including 194 unigenes, and these genes may play a critical role in *N. jatamansi* adaptation to the high-altitude environments.

For unigenes without coding for proteins, homologous searches against the Coding Potential Calculator (CPC2), Coding-Non-Coding Index (CNCI), Predictor of Long noncoding RNAs and messenger RNAs based on an improved K-mer scheme (PLEK), and Pfam reference protein databases, and finally 397 of these unigenes were predicted to be long noncoding RNAs (lncRNAs) with an average length of 1,723 bases including 33 microRNAs (**Figure S3**). After simple sequence repeat (SSR) analysis, the total number of identified SSRs was 43,562 and all 26,503 unigenes contained SSR; furthermore, the repeat numbers of the mononucleotides to hexanucleotides were 33,246, 4,828, 5,015, 240, 101, and 132, respectively (**Figure S4**).

### Identification and analysis of the TPS gene family

Terpenoids are the largest family of natural products. In the biosynthetic pathway of terpenoids, prenyltransferases (PTs) convert the universal isopentenyl diphosphate (IPP) and dimethylallyl diphosphate (DMAPP) into polyprenyl diphosphates, such as C10 geranyl diphosphate (GPP), C15 farnesyl diphosphate (FPP), and C20 geranylgeranyl diphosphate (GGPP) (Vandermoten et al., 2009). Subsequently, terpenoid synthases (TPSs) catalyse polyprenyl diphosphates into diverse hydrocarbons or oxygenated scaffolds, which are considered to be one of the most complex biochemical reactions and play an important role in determining the complex diversity of terpenoids (Chen et al., 2011; Christianson, 2017). Therefore, *TPS* genes should play crucial roles in the formation of the large number of terpenoids in *N. jatamansi*. Nevertheless, no studies have been performed on the TPSs in *N. jatamansi*.

**Figure 3:**
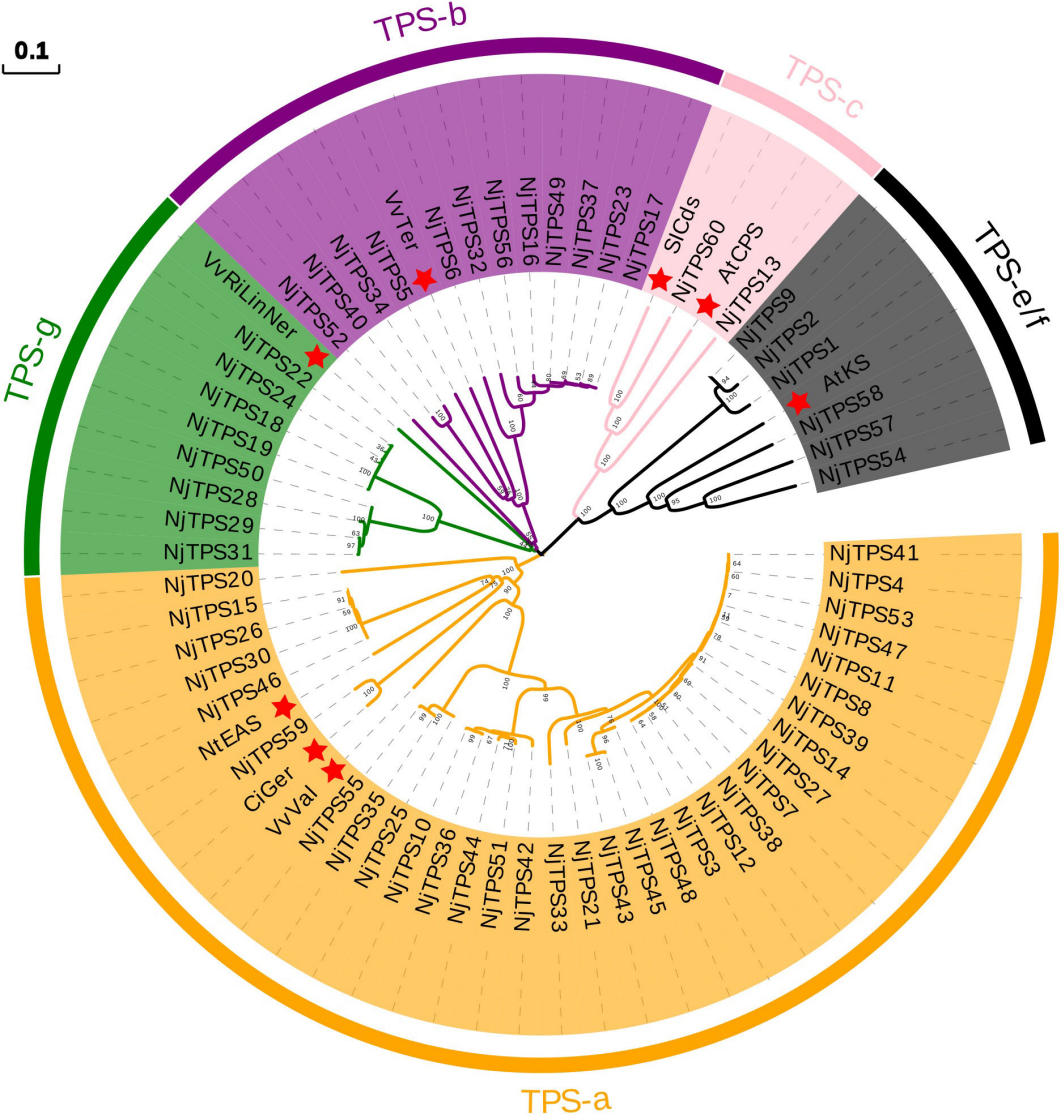
Phylogenetic tree of non-redundant NjTPSs. The results illustrated that most of the NjTPSs belong to the members of TPS-a subfamily, which was mainly composed of sesquiterpene synthases. Grouping into subfamilies is based on previous study (Chen et al., 2011). Sequence abbreviations (marked by red star): VvTer, (−)-α-terpineol synthase (AAS79352, *Vitis vinifera*); VvVal, (+)-valencene synthase (AAS66358, *V. vinifera*); NtEAS, 5-epi-aristolochene synthase (Q40577, *Nicotiana tabacum*); SlCds, copalyl diphosphate synthase (BAA84918, *Solanum lycopersicum*); AtCPS, ent-copalyl diphosphate synthase (Q38802, *Arabidopsis thaliana*); AtKS, ent-kaurene synthase (Q9SAK2, *A. thaliana*); CiGer, germacrene A synthase (AAM21658, *Cichorium intybus*); VvRiLinNer, riesling linalool/nerolidol synthase (JQ062931, *V. vinifera*). The full-length amino acid sequence of NjTPSs described in **Additional File 3**. The tree was derived by neighbour-joining distance analysis based on a ClustalW protein sequence alignment in MEGA7. Numbers above branches are bootstrap values.

**Figure 4:**
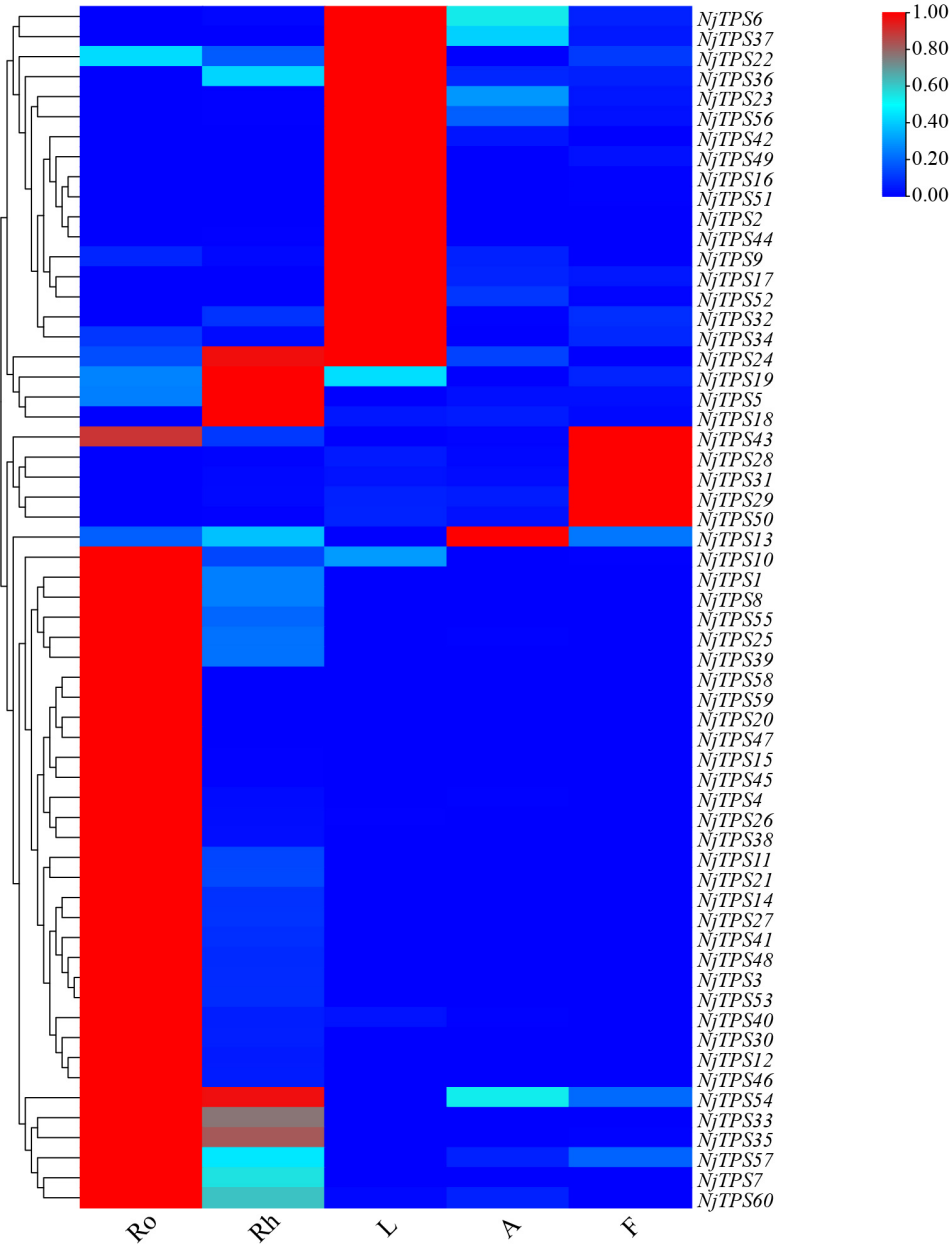
Heat map of non-redundant *NjTPSs*. The results illustrated that expression levels of NjTPSs in different tissues were significantly varied, especially for the members of TPS-a subfamily.

Here, sixty-three nonredundant *NjTPSs* were identified from the transcriptomes using Pfam annotation and the BLAST algorithm. Then, these sixty-three gene models were designated *NjTPS1* through *NjTPS63*, among which *NjTPS61*, *NjTPS62* and *NjTPS63* appeared to have fragmentary open reading frames (ORFs) because their protein coding regions failed to meet the combination of the γ, β, and α domains and the presence of unique motifs (“RR(X_8_)W”, “DDXXD”, “DXDD”, “NSE/DTE”) of plant TPSs (Alicandri et al., 2020). In plants, *TPS* genes are generally classified into seven major subfamilies (or clades) designated TPS-a through TPS-g, and the function and taxonomic distribution of TPSs are closely related to their subfamilies (Chen et al., 2011). Therefore, eight other branch TPS genes, which used to group and infer the function of the TPS genes, and the sixty complete NjTPSs were used to construct a composite neighbour-joining tree (**Figure 3**). The phylogenetic tree indicated that these NjTPSs were classified into the TPS-a, TPS-b, TPS-c, TPS-g, and TPS-e/f subfamilies. However, more than half of the NjTPSs were grouped into the TPS-a subfamily. In past research, most of the functionally identified TPSs of TPS-a subfamily were sesquiterpene synthases (Chen et al., 2011; Alicandri et al., 2020). The metabolites extracted from *N. jatamansi* (e.g., nardosinone-type sesquiterpenoids) are sesquiterpene derivatives. Therefore, the NjTPSs of the TPS-a subfamily may be involved in the formation of sesquiterpene skeletons in *N. jatamansi*. Notably, partial members in the TPS-g and TPS-e/f clades can also produce the formation of sesquiterpene skeletons (Chen et al., 2011; Alicandri et al., 2020). The plant hormones gibberellins are crucial to plant growth and development. In the gibberellin biosynthetic pathway, the enzyme *ent*-copalyl diphosphate (*ent*-CPP) synthase (CPS, TPS-c calde) catalyses its linear substrate GGPP conversion into the two-ring cyclization product *ent*-CPP. Subsequently, the enzyme encoded by the *ent*-kaurene synthase (KS, TPS-e/f clade) gene catalyses *ent*-CPP to produce *ent*-kaurene, which is an important precursor to gibberellins (Hayashi et al., 2010). Therefore, the *NjTPSs* in these two clades should be closely related to the growth and development of *N. jatamansi*. In addition, the *NjTPSs* of these two clades had a long unique terminal branch, indicating that these genes may have evolved more rapidly. Moreover, the expression levels of these NjTPSs in five tissues were also analysed (**Figure 4**). The results showed that their expression levels were significantly varied in different tissues, and the genes of the TPS-a family were more likely expressed in the underground part (UP: root and rhizome).

### Identification and analysis of the CYP gene family

**Figure 5:**
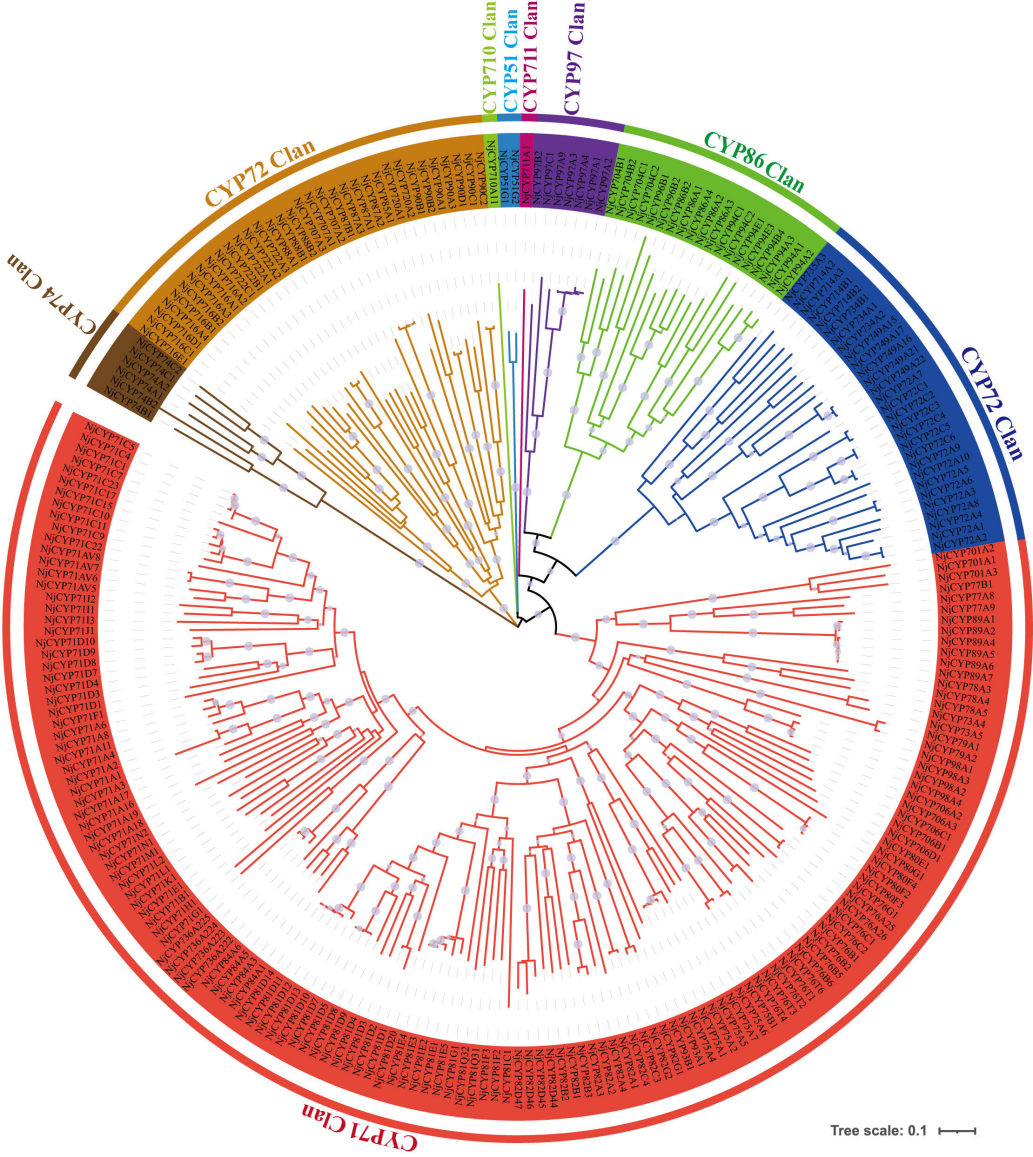
Phylogenetic tree of non-redundant NjCYPs. Phylogenetic tree illustrating the NjCYPs were separated into nine clans. The full-length amino acid sequence of NjCYPs described in **Additional File 4**. Clan separation referenced to the data in website: http://www.p450.kvl.dk/cyp_allsubfam_NJ_102103.pdf. The CYP71 clan belongs to the A-type CYPs, while the other clans belong to the non-A-type CYPs. Phylogenetic relationship was derived by neighbour-joining distance analysis based on a ClustalW protein sequence alignment in MEGA7. Quality of the tree was analyzed by bootstrapping, where values over 50% are indicated above the nodes using symbol.

**Figure 6:**
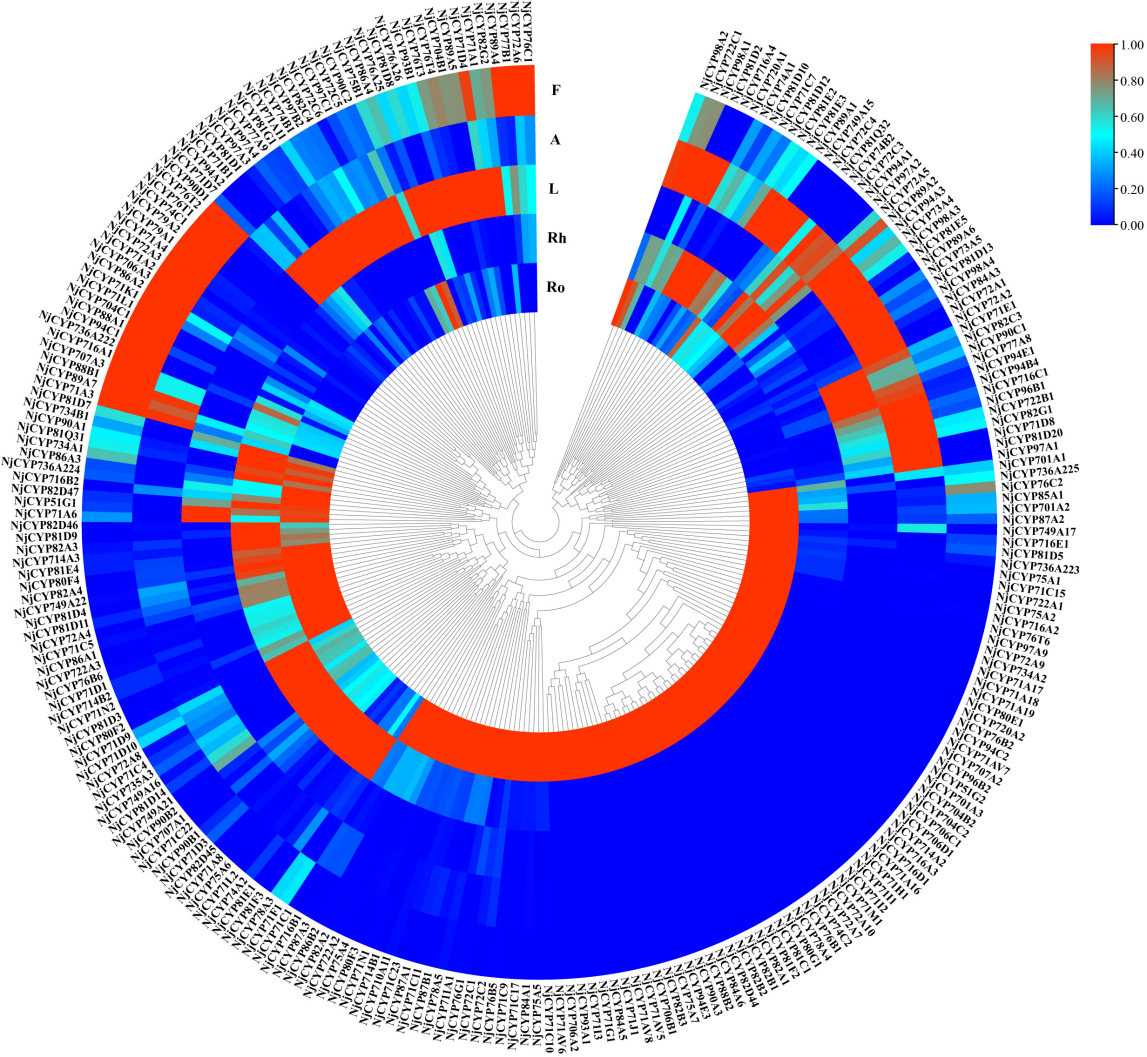
Heat map of non-redundant NjCYPs. The results illustrated that expression levels of NjCYPs in different tissues were significantly varied.

In terpenoid biosynthesis, the complex scaffolds are often oxidatively functionalised by the action of cytochromes P450 (CYPs), resulting in stereospecific hydroxylation to terpenoid scaffolds, and then serve as anchoring points for further modifications, such as glycosylation, alkylation or esterification. Notably, the oxidative functionalization of terpenoid scaffold can also be the result of water quenching of the carbocation during catalysis by TPSs (Pateraki et al., 2015). In the current study, two hundred eighty-nine nonredundant *NjCYPs* were characterized from the transcriptomes. Based on the cross-kingdom nomenclature system of CYPs with reference to the classification of CYPs in *Arabidopsis* (Suzanne et al., 2000; Nelson, 2019; Hansen et al., 2021), the 289 NjCYPs were assigned to 39 families (**Table S6**). Of these NjCYPs, 38 genes lacked the heme-binding motif (FxxGxxxCxG), ExxR motif in the helical K region, or the electron-transfer channel motif (PERF), which are well-conserved motifs in P450s (Werck-Reichhart et al., 2002). Based on the CYP clan separation on the website http://www.p450.kvl.dk/cyp_allsubfam_NJ_102103.pdf, the genes with complete ORFs were further clustered into clans defined by the phylogenetic tree (**Figure 5**). The expression levels of these complete NjCYPs in five tissues are shown in **Figure 6**.

To date, most functional plant P450s with activity as sesquiterpene oxidases have been clustered into the CYP71 clan. In land plants, functionally characterized members of the CYP736 and CYP706 families show activities related to small compounds (e.g., monoterpenes and sesquiterpenes) (Boachon et al., 2019; Hansen et al., 2021). Members of the CYP82 family often show broad catalytic activities towards various terpenes, flavones, coumarins, and alkaloids (Lee et al., 2010). Until now, the A-type CYPs of the CYP71 family have exhibited broad catalytic activity in monoterpene and sesquiterpene biosynthesis (Bathe and Tissier, 2019). In maize, CYPs from the CYP81 family can catalyse diverse oxygenation and desaturation reactions of sesquiterpene and its derivatives (Ding et al., 2020). In chicory, the enzyme encoded by CYP76AV8 conversed valencene to result in predominant accumulation of the alcohol nootkatol, which was very similar to that of 4A,5-dimethyl-3-(prop-1-en-2-yl)-1,2,3,4,4a,5,6,7-octahydrophthalen-1-ol and isoalenyl formate in *N. jatamansi* (Cankar et al., 2011). Notably, members (e.g., MAX1) of CYP711A can also oxygenate the sesquiterpenoids (Yoneyama et al., 2018). In our phylogenetic tree, more than half of the NjCYPs belonged to the CYP71 clan, including the CYP71, CYP76, CYP81, CYP82, CYP706, and CYP736 families. One gene (*NjCYP711A1*) from the CYP711A family was also obtained. It was reasonable to believe that NjCYPs in these families might be candidates for a possible role in sesquiterpenoid biosynthesis in *N. jatamansi*.

### Analysis of differentially expressed genes in tissue

First, 26,503 transcripts from SMRT sequencing were mapped by the NGS clean reads in each tissue, and then the FPKM value of each transcript in the tissues was obtained. A total of 26,482 expressed unigenes (FPKM > 0) were successfully determined. The expression of unigenes in each sample is shown in **Figure S5**. The cluster analysis of sample-to-sample based on gene expression showed that samples from the UP and the aerial part (AP: leaf, flower and anthocaulus) of *N. jatamansi* were clustered into separate groups, indicating that there was a huge difference in gene expression between them (**Figure S6**).

A total of 11,794, 11,571, 9,479, 11,281, 6,381, 11,350, 10,582, 10,287, 9,810, and 11,882 genes were differentially expressed in the Nj_Ro-vs.-Nj_L, Nj_Ro-vs.-Nj_A, Nj_L-vs.-Nj_F, Nj_Rh-vs.-Nj_F, Nj_Rh-vs.-Nj_Ro, Nj_Ro-vs.-Nj_F, Nj_Rh-vs.-Nj_A, Nj_A-vs.-Nj_F, Nj_L-vs.-Nj_A, and Nj_Rh-vs.-Nj_L paired groups, respectively (**Figure S7**). The Nj_Rh-vs.-Nj_Ro group had fewer specific *DEGs* than did the other groups, indicating that the difference between roots and rhizomes was relatively small (e.g., the content of nardosinone). One hundred and ten common *DEGs* were found in these paired comparisons (**Figure S8**). Compared to other tissues, there were more upregulated genes than downregulated genes in leaves, implying that the biological processes in the leaves of *N. jatamansi* are more vigorous. From the phytochemical analysis results, the sesquiterpenoids mainly accumulated in the UP than in the AP, especially for the precise quantification of three compounds (nardosinone, kanshone C, and isonardosinone). Therefore, we performed a comparative analysis between the gene set in the AP and the gene set in the UP. However, only 86 and 22 genes were found to be specifically expressed in the UP and AP sets, respectively (**Figure S9**). Further analysis revealed that none of these specifically expressed genes were annotated as genes in the 1-deoxy-D-xylulose-5-phosphate/methyl-erythritol-4-phosphate (MEP) or mevalonate (MVA) pathways, which both proved to be the initial synthetic pathways for terpenes (Zhou and Pichersky, 2020). Only one CYP gene (*NjCYP716A3*) was specifically expressed in the UP. These results indicated that the genes involved in sesquiterpenoid synthesis in *N. jatamansi* are not fully tissue-specific but may only be differentially expressed in tissues.

### Sesquiterpenoids biosynthesis

Typically, limited to paired sample analysis, *DEGs* is difficult to systematically analyze large datasets from heterogeneous sources simultaneously. One coexpression network approach, weighted gene coexpression network analysis (WGCNA) proved to be a powerful method to systematically describe the correlation between clusters of highly correlated modules and sample traits (Pei et al., 2017). In this study, WGCNA was used to analyse the potential genes involved in sesquiterpene biosynthesis. All 26,482 transcripts from SMRT were used as raw data input for coexpression network analysis. After filtering out the genes with low fluctuation expression (standard deviation ≤ 1), 7,708 genes remained for subsequent analysis. These genes were clustered into seven modules (**Figure S10**). The modules related to the traits (accumulation levels of nardosinone, kanshone C, and isonardosinone) were screened depending on the correlation coefficient (absolute value ≥ 0.3) and *p* valu*e* (<0.05). Interestingly, the module-trait relationships indicated that the blue module was positively associated with nardosinone, kanshone C, and isonardosinone (r = 0.99, correlation *p* value ≈ 0) (**Figure S11**). Among the genes in the blue module belonging to the TPSs, including twenty-four genes of TPS-a, two genes of TPS-g, and two genes of TPS-e/f, seventy-seven genes were belong to members of the CYP superfamily, including twenty genes of the CYP71 family, five genes of the CYP76 family, one gene of the CYP736 family, one gene of the CYP706 family, nine genes of the CYP81 family, six genes of the CYP82 family, and the only gene of the CYP711 family. These families were proven to be extensively involved in the catalytic reactions of sesquiterpenoids.

**Figure 7:**
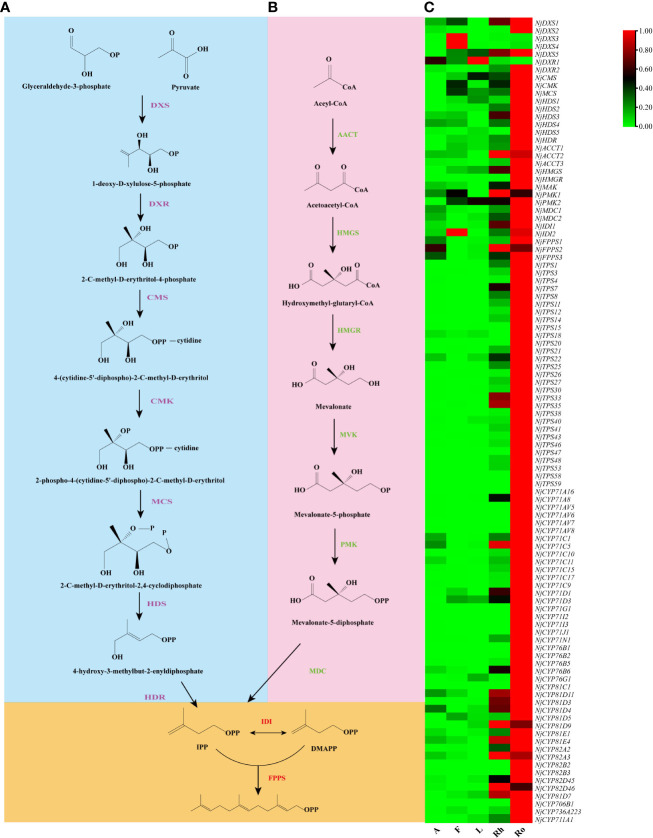
Co-expression analysis. **(A)** MEP pathway. **(B)** MVA pathway. **(C)** Heat map of the twenty-eight NjTPSs and fourty-three NjCYPs potential genes obtained from the WGCNA, and the MEP and MVA genes known to be involved in sesquiterpenoids biosynthesis (data shown in **Table S7**). All potential NjTPSs and NjCYPs are co-expressed with upstream genes.

In plants, the precursors of sesquiterpenoids are actually synthesized *via* the MEP pathway including eight genes (*DXS*, *DXR*, *CMS*, *CMK*, *MCS*, *HDS*, *HDR*, and *IDI*) or the MVA pathway including seven genes (*AACT*, *HMGS*, *HMGR*, *MVK*, *PMK*, *MDC*, and *IDI*). Both pathways synthesized one molecule of IPP and one molecule of DMAPP. IPP and DMAPP undergo sequential condensation reactions by farnesyl diphosphate synthase (FPPS) to form FPP (Jain et al., 2014). Accordingly, genes that catalyze sesquiterpene biosynthesis may exhibit a similar co-expression patterns. Therefore, a similar coexpression analysis between the genes involved in sesquiterpenoid biosynthesis, including the NjTPSs and NjCYPs of the blue module, was further performed. Notably, twenty-nine NjTPSs and forty-three NjCYPs were then identified as being highly coexpressed with the genes in upstream biosynthesis pathway (**Figure 7**; **Table S7**), and there is reason to believe that the coexpressed genes may be involved in the synthesis of the related sesquiterpenoids, which needs more research. In addition, the expression of genes in the MVA pathways appeared to be more correlated with changes in accumulated sesquiterpenoids in *N. jatamansi*. This is consistent with the dominance of the MVA pathway for the synthesis of sesquiterpenes and triterpenoids in plants.

### Validation of gene expression profiles by qRT–PCR

**Figure 8:**
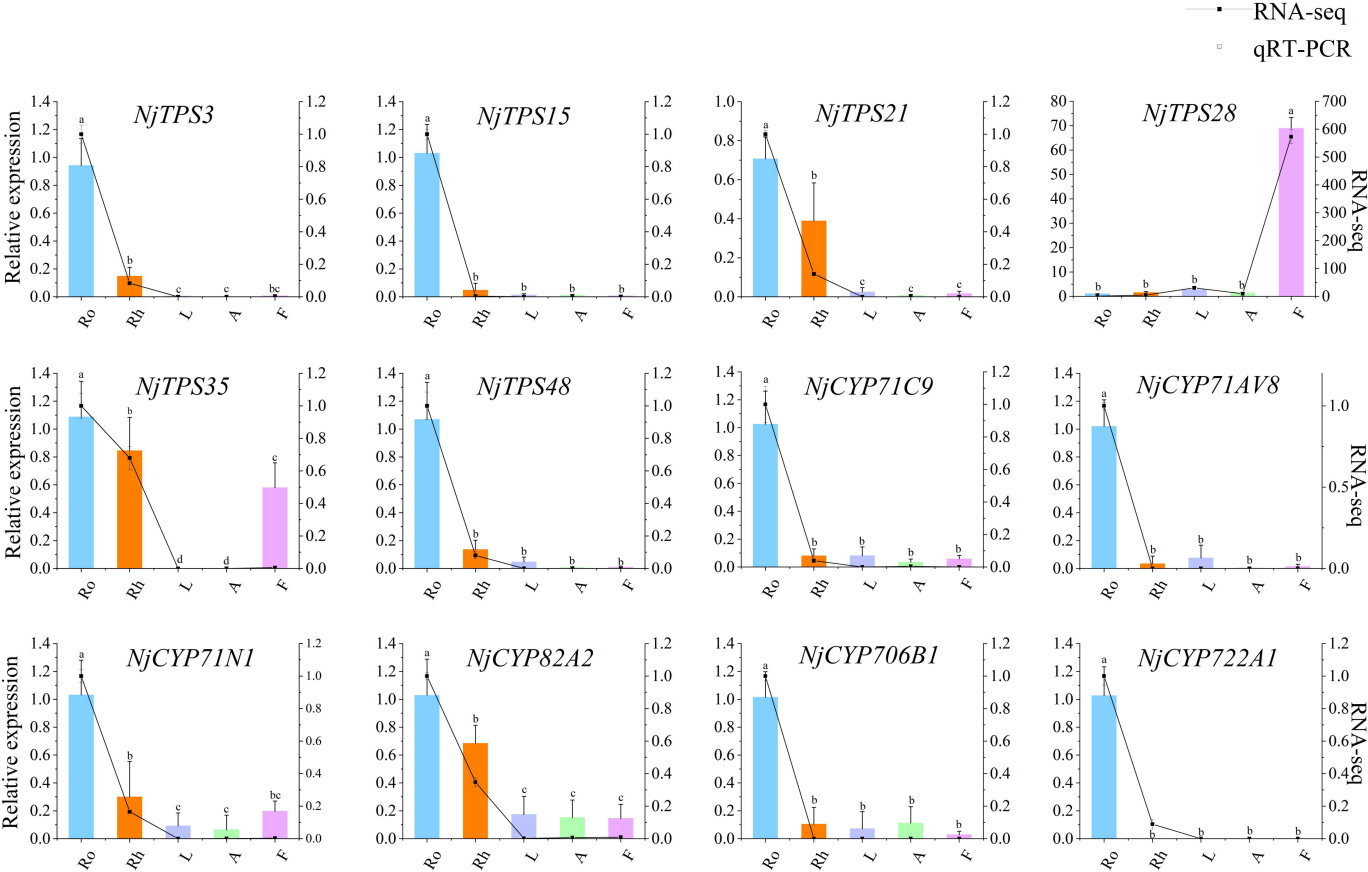
qRT-PCR validation of twelve potential NjTPSs and NjCYPs identified by RNA-seq. The letter denotes statistical signifcance (different letters denote *P* < 0.05).

To confirm the reliability of the transcriptome sequencing results, twelve genes from the *NjTPSs* and *NjCYPs* were chosen for quantitative analysis. The primers of qRT–PCR are shown in **Table S8**. The housekeeping gene is *Actin* (**Additional File 5**). The expression tendency between the qRT–PCR and transcriptomic analyses was similar, which indicating that the transcriptome analysis results were reliable (**Figure 8**).

## Discussion

In response to biotic and abiotic environments, plants often synthesize specialized metabolites (Yang et al., 2012). In terrestrial organisms, sesquiterpenoids possess a wide range of biological activities as semiochemicals, such as defensive agents or pheromones (Laura et al., 2020). For example, *Quercus coccifera* interacts with light and temperature by emitting monoterpene and sesquiterpene (Staudt and Lhoutellier, 2011). In the Qinghai-Tibet Plateau, the most important environmental factors should be the strong ultraviolet rays and extremely low soil temperature. In our study, sesquiterpenoids were found to be highly accumulated in *N. jatamansi*, especially in root and rhizome tissues, implying that these compounds may have the ability to adapt to the specialized ecological environment. Volatile chemicals, including aromatic compounds, help flowering plants attract pollinators (Li et al., 2020). Bag mark experiments showed that outcrossing was the main form of pollination for *N. jatamansi*. From the results of GC-MS analysis, aromatic volatiles, including *m*-cresol, 4-methoxybenzyl alcohol, benzyl alcohol, benzyl pentanoate, and anisyl isovalerate, specifically accumulated in the flowers of *N. jatamansi* (**Table S1**); *m*-cresol and benzyl alcohol were proven to be responsible for attracting beneficial pollinating insects (Zito et al., 2019; Li et al., 2020). These results suggest that different tissues of *N. jatamansi* acquired independent functions to allow this herb to adapt to specific ecological niches during its long-term evolution. Due to its excessive overexploitation and special biological properties (e.g. low germination rate), *N. jatamansi* is now on the verge of extinction (Kaur et al., 2020). In addition, the excavation of *N. jatamansi* has also caused great ecological damage to the plateau. Unfortunately, in the phytochemical analysis, the main active ingredients almost exclusively accumulated in root and rhizome tissues, suggesting that the AP substitution is not a potential choice to protect this endangered alpine plant.

Generally, the accumulation of bioactive compounds in medicinal plant tissues is influenced by *in situ* biosynthesis and transport phenomena (del Baño et al., 2003). The nardosinone-type sesquiterpenoids (nardosinone, kanshone C, and isonardosinone), except for a small amount of them detected in flowers, were mainly detected in roots and rhizomes (**Figure 1D**). In the subsequent transcriptome analysis, genes related to the biosynthesis of nardosinone-type sesquiterpenoids were also highly expressed in roots and rhizomes (**Figure 7**). These results demonstrate that nardosinone-type sesquiterpenoids appear to be mainly synthesized in roots and rhizomes, but their tissue transport abilities needed more studies (e.g. isotope labeling). The volatile sesquiterpenoids appear to be biosynthesized in whole plants, as evidenced by chemical composition analysis (**Table S1** and **Figure 1B**) and associated gene expression patterns (**Figure 7**). Furthermore, we initially identified and functionally characterized thirty-one genes involved in the upstream pathway of sesquiterpene biosynthesis. Almost all genes from the MVA pathway were more highly expressed in the UP than in the MEP pathway, implying the dominant role of the MVA pathway in nardosinone-type sesquiterpenoid biosynthesis; the result is consistent with sesquiterpene biosynthesis in plants.

Combining short-read NGS and long-read SMRT sequencing has greatly promoted the study of the important metabolic pathways in nonmodel plants (Xiao et al., 2013; Xu et al., 2015). For this study, we used NGS and SMRT together to sequence *N. jatamansi* and finally obtained a high-quality full-length transcriptome (unigenes: 26,503, mean length: 2,237 bp) and a large-scale NGS assembled transcriptome, reducing the misassembly and redundancy of genes compared to the NGS data reported by Nisha et al. (Nisha et al., 2020). To our knowledge, this is the first report to characterize the complete structure of transcripts in *N. jatamansi*. These transcriptome data will advance our understanding of plants in the Himalayas, especially on the Qinghai-Tibet Plateau.

Theoretically, it is difficult to systematically reveal the roles of large gene families (e.g. CYP family) in metabolite synthesis through a single method. In the current study, we systematically revealed the correlation relationships between *NjTPSs*, *NjCYPs* and nardosinone-type sesquiterpenoids biosynthesis based on the following rules: (1) all *NjTPS* and *NjCYP* genes were identified and functionally classified (e.g., TPSs should belong to the TPS-a subfamily, which were identified in the formation of the sesquiterpene skeleton); (2) they were coexpressed with metabolites; (3) they were coexpressed with genes in the biosynthetic pathway of metabolites; and (4) genes with fragmentary ORFs were excluded. We believe that this systematic method can accurately identify target genes.

Taken together, current studies illuminate that sesquiterpenoids are the major chemical constituents of *N. jatamansi* and their biosynthesis and accumulation patterns in the underground plant parts and the aerial plant parts are different. The underground plant parts (root and rhizome) are primarily responsible for the biosynthesis of sesquiterpenoids. This study will also help to uncover genes involved in the biosynthetic pathway of sesquiterpenoids (especially nardosinone-type) to achieve future heterologous microbial synthesis.

## Methods

### Plant material

Four-year-old *N. jatamansi* plants were cultivated in the experimental field on the Qinghai-Tibet Plateau of Southwest Minzu University (Hongyuan, Sichuan Province in China). Five tissues (root, rhizome, leaf, flower, and anthocaulus) with different concentrations of the sesquiterpenoids were separately harvested from twenty independent healthy plants in the later period of blooming. Each tissue had three repetitions. Then, all samples were equally divided into two portions. One portion was immediately frozen in liquid nitrogen and stored at -80°C for RNA sequencing. To obtain the full-length transcriptome of *N. jatamansi*, samples from whole plants in different growth periods were separately collected and then equally mixed. The collection of all experimental materials was performed in accordance with local legislation and and in compliance with the Convention on the Trade in Endangered Species of Wild Fauna and Flora.

### Analysis of volatile components by headspace GC-MS

The analysis was performed by headspace GC-MS using a GCMS-TQ8050 NX triple quadrupole (Shimadzu company, Japan) system with an AOC-6000 autosampler (Shimadzu company). An InterCap Pure-wax (30 m × 0.25 mm × 0.25 μm) column (Shimadzu company) use for the chromatographic separation. The injection system’s optimum parameters were set as follows: accurately weighing approximately 0.2 g powder, incubation at 50 °C for 5 min, oscillator speed 500 rpm, SPME arrow injection, and enrichment at 50 °C for 20 min. The oven temperature started from 40 °C for 3 min, then increased with a 15 °C/min rate to 120 °C, and then increased with a 2 °C/min rate to 140 °C, and finally increased with a 10 °C/min rate to 250 °C for 6 min. Helium (> 99.999%) was used as a carrier gas and was set at a constant flow rate of 1 mL/min. The inlet temperature was set at 250 °C, and split injection mode was applied at a ratio of 1:30. Electron ionization was applied at 70 eV, and the ion source temperature was 200 °C. The interface temperature was 250 °C. The solvent cutting time was 1.5 min. Full scan spectra were acquired from 35 to 500 amu with a 300 ms scan time. A Shimadzu gas quality workstation (GC-MS solution Ver 4.53) and NIST20 Mass Spectral Library were used to analyse the chromatographic data. The peak integration parameters were set as follows: slope (S) was 100, peak width at half-height (W) was 3, drift (D) was 0, parameter change time (T) was 1000, minimum peak area (M) was 1000, smoothing times (F) was 1, and smoothing peak width at half-height (O) was 1. Under the above peak integration parameters, chromatographic peaks are automatically integrated. The mass spectrum of each peak was compared with the standard spectrums in NIST20 spectrum library, and compounds with a similarity score of more than 85 were selected. In addition, the selected compounds were further compared with those reported in the literatures of *N. jatamansi*.

### Determination of main nardosinone-type sesquiterpenoid content

The authentic standards of nardosinone, kanshone C, and isonardosinone were accurately weighed and then mixed with methanol. All standards were isolated and identified in our laboratory, and their purity was more than 98%. Powdered tissues (0.5 g) were accurately weighed and transferred to a conical bottle (150 mL). Then, 20 mL methanol (1:40 ratio, g/mL) was added and extracted for 45 min at 300 W using an ultrasonic machine (KQ-300DE, Kun-shan Ultrasonic Instrument Co., Ltd., China). After cooling, the lost weight was made up with methanol. Finally, the well shaken mixture was filtered with a 0.22 μm microporous membrane. UPLC analysis was performed on a Waters Acquity UPLC^®^ system (Waters, USA). The samples were separated on an ACQUITY UPLC^®^ HSS T3 column (2.1×100 mm, 1.8 μm) with the following conditions: injection volume, 2 μL; flow rate, 0.2 mL/min; column temperature, 30°C; and wavelength, 254 nm. The mobile phases of acetonitrile (A) and water with 0.1% phosphoric acid (B) were used for elution as follows: 0-8 min, 30% A to 55% A; 8-15 min, 55% A to 60% A; 15-25 min, 60% A to 60% A; and 25-28 min, 60% A to 90% A. The limit of detection (LOD), limit of quantitation (LOQ), calibration curve, mean correlation coefficient, linear range, accuracy, injection precision, stability, and system suitability were used to validate the method.

### RNA isolation and library preparation

Total RNA was extracted using the mirVana miRNA Isolation Kit (Cat. AM1561, Invitrogen, Thermo Fisher Scientific Inc., USA) according to the manufacturer’s instructions. NanoDrop 2000 spectrophotometer (Thermo Fisher Scientific, Waltham, MA, USA) was used to evaluate the RNA purity and quantification. Agilent 2100 Bioanalyzer (Agilent Technologies, Santa Clara, CA, USA) was used to assess the RNA integrity. TruSeq Stranded mRNA LT Sample Prep Kit (Illumina, San Diego, CA, USA) was used to construct libraries following the manufacturer’s protocol. OE Biotech Co., Ltd. (Shanghai, China) performed the transcriptome sequencing.

One microgram of total RNA extracted from whole plants in different growth periods was used for cDNA synthesis using a SMARTer PCR cDNA Synthesis kit (Clontech, USA). 12 PCR cycles of amplification were performed using PrimeSTAR GXL DNA Polymerase (Clontech). The cDNA purified by AMPure PB Beads was subjected to construct SMRTbell template libraries using the SMRTbell Template Prep Kit 1.0 (PacBio, Menlo Park, USA).

### RNA sequencing and annotation

The libraries of fifteen samples were sequenced on the Illumina HiSeq X Ten platform, and 150 bp paired-end reads were generated. First, raw reads were processed using Trimmomatic (v0.36, parameter: LEADING:3 TRAILING:3 SLIDINGWINDOW:4:15 MIN) (Bolger et al., 2014). The reads containing poly-N and the low-quality reads were removed to obtain clean reads. After removing low-quality sequences and adaptors, the remaining clean reads were assembled into expressed sequence tag clusters (contigs) and *de novo* assembled into transcripts by Trinity (version: 2.4.0, parameter: –seqType fq –SS_lib_type RF) with the paired-end method (Grabherr et al., 2011). The longest transcript was chosen based on length analysis. Then, the transcripts were clustered using CD-HIT (version: 4.6, parameter: with default) to generate nonredundant unigenes (Li et al., 2001). SMRT cells were sequenced on a PacBio Sequel II platform using sequencing kit 2.1 with 10 h movie recordings at OE Biotech Co., Ltd. First, SMRT raw reads were processed using SMRTlink (version 6.0), and CCSs (circular consensus sequences) were generated from subread BAM files. Then, the CCSs were trimmed and clustered to obtain FLNC (full-length nonchimeric). These FLNC reads were clustered to obtain a consensus, and nonredundant unigenes were obtained.

Functional annotation of the unigenes was conducted by alignment of the unigenes with the NCBI nonredundant (NR), Swiss-Prot, evolutionary genealogy of genes: Non-supervised Orthologous Groups (eggNOG) and Clusters of orthologous groups for eukaryotic complete genomes (KOG) databases using diamond with a threshold E-value of 10^-5^ (Benjamin et al., 2015). The proteins with the highest hits to the unigenes were used to assign functional annotations. The unigenes were also mapped to the Kyoto Encyclopedia of Genes and Genomes (KEGG) database to annotate the potential metabolic pathways. Gene Ontology (GO) classification was performed through the mapping relation between Swiss-Prot and GO terms. The annotation of protein families was performed by HMMER3 analysis (Mistry et al., 2013).

The LncRNA prediction was followed as: (1) unigenes with length upper than 200 bp were screened out; (2) removing the screened unigenes annotated on the coding libraries; (3) the coding ability of remaining unigenes was further analysed by CPC2 (version: v2.0, parameter: with default), CNCI (version: v2.0, parameter: -m ve), PLEK (version: v1.2, parameter: with default) and Pfam (version: v2015-06-02, parameter: -e_seq 0.001).

SSR analysis was performed using MISA (version: v1.0, parameter: with default) and Primer3 (version: v2.4.0, parameter: -default_version=2 -io_version=4). The SSR types were clustered as mononucleotide, dinucleotide, trinucleotide, tetranucleotide, pentanucleotide, and hexanucleotide. The repeat times were divided into 5, 6, 7, 8, 9, 10, 11, and > 11 times.

### Differentially expressed gene analysis

The clean reads from Illumina sequencing were mapped into the nonredundant unigenes from Pacific Biosciences to obtain the reads on unigenes for each tissue using Bowtie2 software (version: 2.3.3.1, parameter: –reorder -k30 -t) (Langmead and Salzberg, 2012) and then calculated per kilobase per million (FPKM) values using eXpress software (version: 1.5.1, parameter: –rf-stranded) (Roberts and Pachter, 2013). *DEGs* between different tissues were identified using the DESeq software (version: 1.18.0, parameter: *p* value<0.05, |log2FoldChange|>1) (Anders and Huber, 2012). GO enrichment and KEGG pathway enrichment analyses of *DEGs* were conducted by R based on the hypergeometric distribution.

### Quantitative real time PCR verification analysis

qRT–PCR was used to verify the reliability of the transcriptional data. Extracted RNA from fifteen sequencing samples was reversed into cDNA using a HiScript^®^ II Q RT SuperMix for qPCR (+gDNA wiper) Kit (Vazyme, China). *Actin* was chosen as the housekeeping gene. All qRT–PCR primers were designed using Primer6.0 (Premier Biosoft, Canada). RT–PCR was used to check the specificity of all primers. qRT–PCR in triplicate was carried out using the 2 × Taq Pro Universal SYBR qPCR Master Mix kit (Vazyme, China). The PCR procedure was as follows: 95°C for 30 s (hold stage); 40 cycles at 95°C for 5 s and 57°C for 30 s (PCR stage); 95°C for 15 s, 60°C for 60 s, and 95°C for 15 s (melting curve stage). The relative expression levels were calculated by the 2^-ΔΔCt^ comparative threshold cycle (Ct) method (Schmittgen and Livak, 2008).

### Phylogenetic and heatmap analysis

Unigenes belonging to *TPSs* and *CYPs* were identified using BLAST software according to the characteristic domains. In order not to miss candidate genes, all *TPS* and *CYP* genes obtained from PacBio and ILLUMINA sequencing were combined. Unigenes with fragmentary ORFs were removed. Guided by the nomenclature system (Nelson, 2019), the CYPs were systematically classified and named. Numerous functional sequences identified from other plant species were selected and pooled with those TPSs to construct the composite tree using MEGA7. The phylogenetic trees of TPSs and CYPs were constructed using the neighbour-joining method with the amino acid sequences of the ORFs. The topology of phylogeny was evaluated by a bootstrap resampling analysis with 1,000 replicates. The heatmaps of *TPSs* and *CYPs* were constructed by TBtools software (Chen et al., 2020).

### Coexpression network analysis

WGCNA to *N. jatamansi* transcriptome datasets was conducted to identify potential transcript modules related to the biosynthesis of major sesquiterpenoids. The R package WGCNA was used to complete statistical analysis. Unsigned, weighted correlation networks were constructed by the R package WGCNA with a default power of twenty-eight. The network visualization was accomplished by R language, Python, and heatmaps.

## Data availability statement

The original contributions presented in the study are publicly available. This data can be found here: NCBI, NGS sequencing- PRJNA858921 and SMRT sequencing- PRJNA841858.

## Author contributions

MF and SZ performed most of the experiments and data analysis and wrote the manuscript. CC, JQ-B, QC and YLi helped to plant and collect the materials. AQ-B and XY assisted in the HPLC experiments. XB was responsible for GC-MS experiment and data analysis. YLiu designed and coordinated the studies. All authors contributed to the article and approved the submitted version.

## Funding

This work was supported by the National Key Research and Development Program of China (2018YFC1706101), the Natural Science Foundation of Sichuan Province (Grant No. 2022NSFSC1434) and the Southwest Minzu University Research Startup Funds (Grant No. RQD2021048). The supporters had no role in study design, data collection, data analysis, the writing of the manuscript or decision to publish.

## Conflict of interest

Author XB was employed by Shimadzu China Co., Ltd.

The remaining authors declare that the research was conducted in the absence of any commercial or financial relationships that could be construed as a potential conflict of interest.

## Publisher’s note

All claims expressed in this article are solely those of the authors and do not necessarily represent those of their affiliated organizations, or those of the publisher, the editors and the reviewers. Any product that may be evaluated in this article, or claim that may be made by its manufacturer, is not guaranteed or endorsed by the publisher.

## Supplementary material

Additional file 1 This file includes all additional tables (**Tables S1**-**S8**) used in this manuscript. Table numbers and titles were listed as follows:

Supplementary Table 1 Phytochemicals present in *N. jatamansi* by GC-MS and their relative abundance in different tissues.

Supplementary Table 2 Validation method parameters for quantification of three Sesquiterpenoids.

Supplementary Table 3 Summary of transcriptome data sequenced.

Supplementary Table 4 Statistics of NGS sequencing assembly in *N. jatamansi*.

Supplementary Table 5 BLAST analysis of unigenes against seven public databases.

Supplementary Table 6 The NjCYPs families and the members number in each family.

Supplementary Table 7 The FPKM values of genes co-expressed in the sesquiterpenoids biosynthesis.

Supplementary Table 8 Primers and annealing length in qRT-PCR.

Additional file 2 This file includes all additional figures (**Figures S1**-**S11**) used in this manuscript. Figure numbers and titles were listed as follows:

Supplementary Figure 1 Assembled sequence length distribution from Illumina HiSeq X Ten platform.

Supplementary Figure 2 Non-redundant read length distribution from Pacific Biosciences Sequel platform.

Supplementary Figure 3 Length distribution of IncRNA from Pacific Biosciences Sequel platform.

Supplementary Figure 4 SSR type statistics from Pacific Biosciences Sequel platform.

Supplementary Figure 5 The expression of unigenes obtained from SMRT sequencing in each sample.

Supplementary Figure 6 Cluster map of sample-to-sample based on gene expression obtained from SMRT sequencing.

Supplementary Figure 7 The number of up-down regulated *DEGs* of the ten paired comparisons.

Supplementary Figure 8 Venn diagram of *DEGs* from the ten paired comparisons.

Supplementary Figure 9 Venn diagram of genes from AP and UP.

Supplementary Figure 10 Network visualization plots: Clustering dendrograms of genes with dissimilarity based on topological overlap together with assigned module colours.

Supplementary Figure 11 Network visualization plots: Module-trait relationships.

Additional file 3 The full-length amino acid sequence of NjTPSs in this study.

Additional file 4 The full-length amino acid sequence of NjCYPs in this study.

Additional file 5 The nucleotide sequence of the *actin* in this study.

## Glossary

**Table:**

TPS	terpene synthase
CYP	cytochrome P450
SMRT	single-molecule real-time
NGS	next-generation sequencing
GC-MS	gas chromatography-mass spectrometry
UPLC	Ultra Performance Liquid Chromatography
WGCNA	weighted gene co-expression network analysis
MEP	1-deoxy-D-xylulose-5-phosphate/methyl-erythritol-4-phosphate
MVA	mevalonate
IPP	isopentenyl diphosphate
DMAPP	dimethylallyl diphosphate
GPPS	geranyl diphosphate synthase
GPP	geranyl diphosphate
qRT-PCR	quantitative real-time polymerase chain reaction
ORF	open reading frames
CCS	Circular Consensus Sequences
FLNC	full-length non-chimeric
FPKM	fragments per kilobase per million
ACCT	acetyl-coenzymeA (CoA) C-acetyltransferase
HMGS	3-hydroxy-3-methylglutaryl-CoA synthase
HMGR	3-hydroxy-3-methylglutaryl-CoA reductase
PMK	phosphomevalonate kinase
MDC	mevalonate-5-diphosphate decarboxylase
IDI	IPP isomerase
DXS	deoxyxyulose-5-phosphate synthase
DXR	deoxyxyulose-5-phosphate reductoisomerase
CMS	4-diphosphocytidyl-2-C-methyl-D-erythritol synthase
CMK	4-diphosphocytidyl-2-C-methyl-D-erythritol kinase
MCS	4-diphosphocytidyl-2-C-methyl-D-erythritol 2*4-cyclodiphosphate synthase
HDS	1-hydroxy-2-methyl-2(E)-butenyl 4-diphosphate synthase
HDR	1-hydroxy-2-methyl-2(E)-butenyl 4-diphosphate reductase
FPPS	farnesyl diphosphate synthase
FPP	farnesyl diphosphate
AP	aerial part
UP	underground part
