# Supplementary material for: Metabolome and transcriptome associated analysis of sesquiterpenoid metabolism in Nardostachys jatamansi
Source: Front Plant Sci. 2022 Nov 29;13:1041321. doi: 10.3389/fpls.2022.1041321 (PMC9746346; doi:10.3389/fpls.2022.1041321)
Supplement: Additional file 1 — This file includes all additional tables ( Tables S1 - S8 ) used in this manuscript. Table numbers and titles were listed as follows: [file DataSheet_1.zip › Data Sheet 1.PDF]

Table S1 Phytochemicals present in *N. jatamansi* by GC-MS and their relative abundance in different tissues.

Peak No	structure type	Compounds	Molecular formula	Structural formula	Root		Rhizome		Leaf		Anthocaulus		Flower		Reference
					RT/min	RC/%	RT/min	RC/%	RT/min	RC/%	RT/min	RC/%	RT/min	RC/%	
1	Aliphatic	Acetone	C_3H_6O	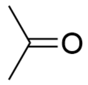	2.244	0.08	2.244	0.15	2.238	0.06	2.245	0.04	2.241	0.03	
2	Aliphatic	Pentanal	$C_5H_{10}O$	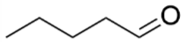	-	-	-	-	3.836	0.05	3.841	0.04	3.838	0.03	
3	Aliphatic	Butanoic acid, 3-methyl-, ethyl ester	$C_7H_{14}O_2$	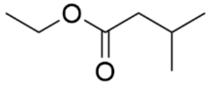	-	-	5.186	0.11	5.183	0.08	-	-	5.183	0.15	
4	Monoterpenoid	Eucalyptol	$C_{10}H_{18}O$	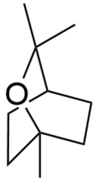	7.008	0.54	7.007	0.65	7.005	0.69	7.004	0.49	7.005	0.77	10.1016/s0367-326x(02)00062-x
5	Aliphatic	Acetoin	$C_4H_8O_2$	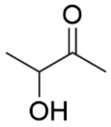	-	-	-	-	-	-	7.764	0.07	-	-	

6	Sesquiterpenoids	4,8,8-trimethyl-2-methylene Bicyclo[5.2.0]nonane	$C_{13}H_{22}$	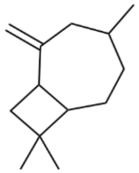	8.014	0.05	-	-	-	-	-	-	-	-	10.1007/s00468-019-01861-2
7	Aliphatic	Acetic acid	$C_2H_4O_2$	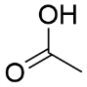	-	-	9.548	0.09	9.545	0.32	9.54	0.53	9.545	0.2	
8	Sesquiterpenoids	*α*-guaiene	$C_{15}H_{24}$	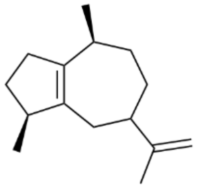	10.017	0.14	10.012	0.1	10.01	0.13	10.01	0.12	10.01	0.15	10.1080/10412905.1997.10554251
9	Sesquiterpenoids	*β*-Patchoulene	$C_{15}H_{24}$	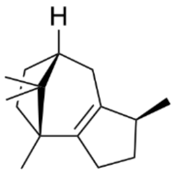	10.384	0.19	-	-	10.374	0.17	10.375	0.16	10.375	0.19	10.1016/j.ejphar.2016.04.028
10	Sesquiterpenoids	*α*-Copaene	$C_{15}H_{24}$	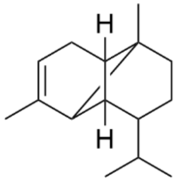	10.475	0.11	-	-	10.464	0.12	10.465	0.1	10.466	0.12	.1016/S0040-39(00)75403-7

11	Sesquiterpenoids	*γ*-Selinene	$C_{15}H_{24}$	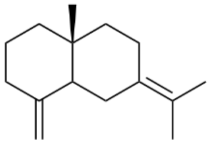	10.832	0.56	10.808	0.4	10.801	0.4	10.8	0.36	10.803	0.43	10.1021/np9601824
12	Sesquiterpenoids	*α*-Gurjunene	$C_{15}H_{24}$	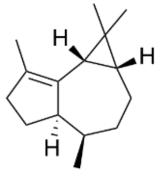	11.088	2.77	11.057	2.13	11.047	2.07	11.047	1.66	11.05	2.01	10.1021/jo01017a009
13	Sesquiterpenoids	*β*-Maaliene	$C_{15}H_{24}$	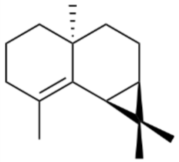	-	-	11.274	10.64	11.261	10.83	11.259	8.97	11.265	10.51	10.1007/s11418-009-0340-x
14	Sesquiterpenoids	*β*-Cadinene	$C_{15}H_{24}$	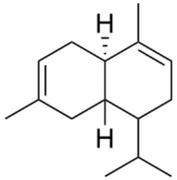	11.308	12.69	-	-	-		-	-	-		10.1135/cccc19663012
15	Sesquiterpenoids	*β*-humulene	$C_{15}H_{24}$	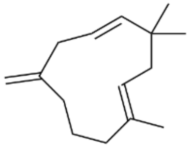	-	-	11.367	0.21	11.361	0.26	11.362	0.26	11.364	0.28	10.1135/cccc19611832

16	Sesquiterpenoids	aristolene	$C_{15}H_{24}$	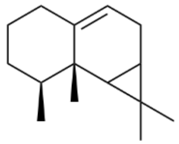	11.726	9.09	11.692	7.56	11.682	8.44	11.684	8.9	11.688	9.23	10.1039/C19680001070
17	Phenolic acids	Carvacrol methyl ether	$C_{11}H_{16}O$	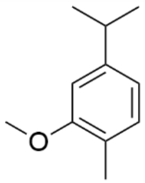	11.921	0.46	11.899	0.39	11.884	0.41	11.888	0.34	11.893	0.48	
18	Sesquiterpenoids	Calarene	$C_{15}H_{24}$	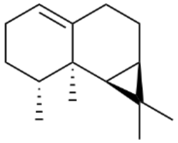	12.249	38.37	12.169	37.86	12.144	43.22	12.153	45.39	12.161	44.3	10.1016/j.phytochem.2005.09.012
19	Sesquiterpenoids	*α*-Maaliene	$C_{15}H_{24}$	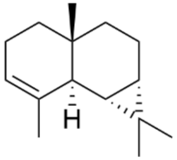	12.334	2.67	12.272	2.3	12.254	2.84	12.26	2.71	12.266	2.83	10.1016/s0031-9422(98)00283-0
20	Sesquiterpenoids	*γ*-gurjunene	$C_{15}H_{24}$	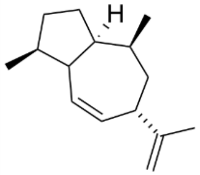	12.446	0.33	12.412	0.25	12.402	0.25	12.405	0.27	12.408	0.28	10.1016/s0031-9422(98)00283-0

21	Sesquiterpenoids	Valencene	$C_{15}H_{24}$	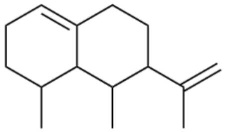	12.542	0.3	12.511	0.23	12.502	0.27	12.504	0.26	12.506	0.27	10.1021/jf60220a054
22	Sesquiterpenoids	Valerena-4,7(11)-diene	$C_{15}H_{24}$	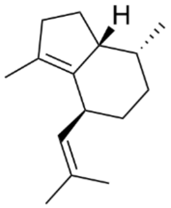	12.732	7.58	12.689	6.73	12.673	7.07	12.676	7.05	12.679	6.87	10.1016/S0031-9422(01)00018-8
23	Sesquiterpenoids	*α*-patchoulene	$C_{15}H_{24}$	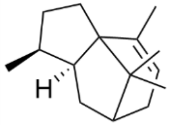	12.944	1.68	12.9	1.3	12.888	1.47	12.891	1.51	12.895	1.53	10.1080/10412905.1997.10554251
24	Sesquiterpenoids	Seychellene	$C_{15}H_{24}$	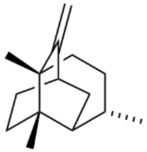	13.045	6.81	12.993	5.8	12.977	6.26	12.982	6.73	12.986	6.65	10.1080/10412905.1997.10554251
25	Aliphatic	Butanoic acid, 3-methyl-	$C_5H_{10}O_2$	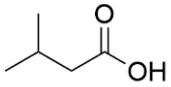	13.164	0.72	13.152	3.73	13.157	3.05	13.155	3.25	13.154	3.24	
26	Sesquiterpenoids	*γ*-Patchoulene	$C_{15}H_{24}$	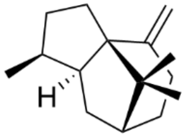	-	-	13.293	0.32	13.282	0.36	13.284	0.38	13.287	0.36	10.1246/bcsj.40.597 Affiliations

27	Sesquiterpenoids	*β*-Guaiene	$C_{15}H_{24}$	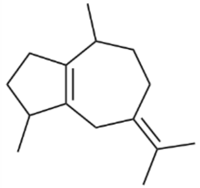	13.335	0.39	-		-		-		-	-	10.1016/j.fct.2016.11.017
28	Sesquiterpenoids	Humulene	$C_{15}H_{24}$	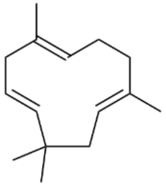	13.564	1.12	13.542	0.92	13.532	0.94	13.535	0.95	13.536	0.94	10.1021/jo3000942
29	Sesquiterpenoids	*γ*-Maaliene	$C_{15}H_{24}$	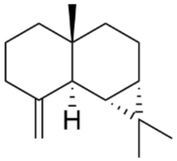	13.688	0.13	13.674	0.1	13.666	0.11	13.668	0.1	13.669	0.1	10.1016/S0031-9422(98)00283-0
30	Monoterpenoid	Myrtenyl acetate	$C_{12}H_{18}O_2$	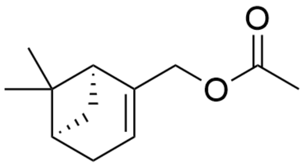	13.819	0.24	13.812	0.16	13.808	0.19	13.808	0.21	13.808	0.18	
31	Sesquiterpenoids	*(E)*-Isovalencenyl formate	$C_{16}H_{24}O_2$	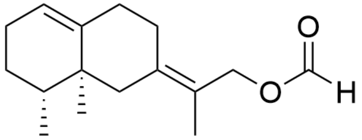	13.96	0.13	13.946	0.11	13.939	0.1	13.94	0.12	13.94	0.11	10.1016/j.chroma.2018.08.050

32	Sesquiterpenoids	4a,5-Dimethyl-3-(prop-1-en-2-yl)-1,2,3,4,4a,5,6,7-octahydronaphthalen-1-ol	$C_{15}H_{24}O$	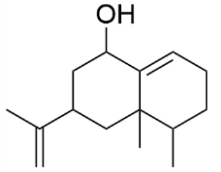	-	-	-	-	-	-	14.098	0.53	14.099	0.51	
33	Sesquiterpenoids	Valerenol	$C_{15}H_{24}O$	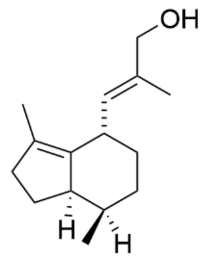	14.121	1.01	14.106	0.72	-	-	-	-	-	-	10.1177/1934578x1300801103
34	Sesquiterpenoids	Khusimyl methyl ether	$C_{16}H_{26}O$	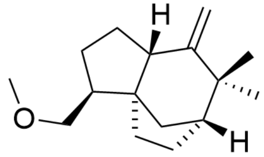	14.261	0.12	14.245	0.09	14.238	0.08	14.239	0.07	14.239	0.07	10.1002/(SICI)1099-1026(200003/04)15:2<61::AID-FFJ865>3.0.CO;2-S

35	Sesquiterpenoids	*β*-Selinene	$C_{15}H_{24}$	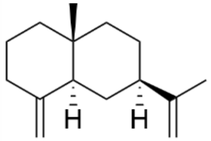	14.394	0.09	-	-	14.376	0.08	14.376	0.08	14.376	0.07	10.1071/CH9780163
36	Sesquiterpenoids	Patchoulene	$C_{15}H_{24}$	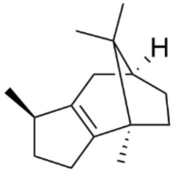	14.505	0.12	-	-	14.48	0.12	14.487	0.13	14.488	0.12	
37	Sesquiterpenoids	7-Isopropenyl-1-methyl-4-methylenedecahydroazulene	$C_{15}H_{24}$	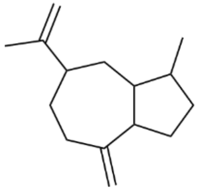	14.641	1.71	14.619	1.33	14.614	1.26	14.615	1.15	14.614	1.08	
38	Sesquiterpenoids	*α*-Selinene	$C_{15}H_{24}$	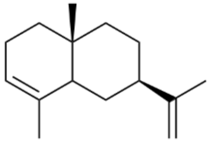	14.765	0.36	14.746	0.26	14.74	0.3	14.742	0.29	14.741	0.27	10.1016/0031-9422(95)00577-t
39	Sesquiterpenoids	*α*-bulnesene	$C_{15}H_{24}$	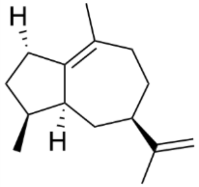	14.834	0.24	14.815	0.18	14.81	0.21	14.811	0.22	14.81	0.2	10.1002/cbdv.201100077

40	Sesquiterpenoids	Bicyclogermacrene	$C_{15}H_{24}$	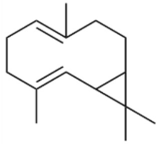	14.964	0.21	14.949	0.23	14.944	0.16	14.946	0.13	14.945	0.12	10.1016/S0040-4039(01)88358-1
41	Sesquiterpenoids	*δ*-Cadinene	$C_{15}H_{24}$	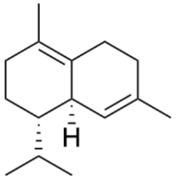	15.581	0.27	15.56	0.23	15.56	0.19	15.56	0.15	15.555	0.13	10.1002/(SICI)1097-458X(199602)34:2<156::AID-OMR859>3.0.CO;2-2
42	Sesquiterpenoids	*α*-Panasinsene	$C_{15}H_{24}$	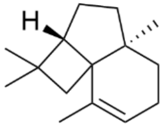	15.694	1.5	15.672	1.33	15.665	1.27	15.667	1.34	15.666	1.14	doi.org/10.1246/bcsj.48.2078
43	Sesquiterpenoids	Dihydro-β-ionone	$C_{13}H_{22}O$	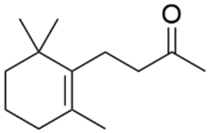	17.457	0.08	17.45	0.09	17.449	0.07	17.45	0.07	17.448	0.06	1007/s1088016-0669-z
44	Phenolic acids	Benzyl alcohol	C_7H_8O	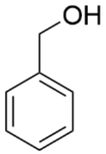	-	-	-		-	-	-		18.378	0.18	

45	Phenolic acids	Benzyl pentanoate	$C_{12}H_{16}O_2$	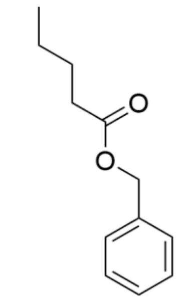	-	-	-	-	-		-	-	19.137	0.2	
46	Sesquiterpenoids	1-Oxaspiro[2.5]octane, 5,5-dimethyl-4-(3-methyl-1,3-butadienyl)-	C14H22O	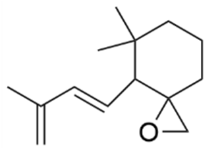	19.798	0.31	19.792	0.36	19.791	0.21	19.793	0.22	19.79	0.16	10.1002/agj2.20923
47	Sesquiterpenoids	Palustrol	$C_{15}H_{26}O$	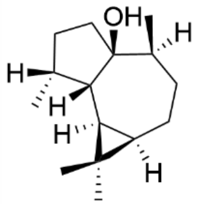	20.028	0.08	20.025	0.08	-	-	-	-	-		10.1016/S0031-9422(00)00291-0
48	Sesquiterpenoids	*β*-Ionone	$C_{13}H_{20}O$	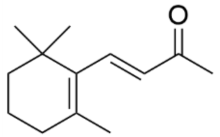	20.22	0.81	20.216	1.24	20.212	0.71	20.212	0.76	20.21	0.5	1007/s10886-016-0669-z

49	Sesquiterpenoids	Cubebol	$C_{15}H_{26}O$	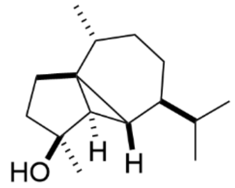	-	-	20.357	0.12		-	-	-	-		10.1002/chem.200501299
50	Diterpenoids	Neophytadiene	$C_{20}H_{38}$	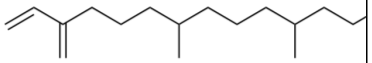	-		-		20.549	0.05	-		-		
51	Sesquiterpenoids	*β*-Ionol	$C_{13}H_{22}O$	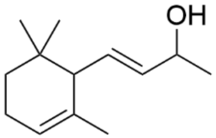	20.623	0.1	20.619	0.13	-	-	-	-	-	-	10.1021/acscatal.6b01882
52	Sesquiterpenoids	Maaliol	$C_{15}H_{26}O$	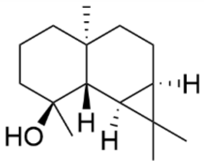	21.417	0.93	21.414	1.43	21.41	0.68	21.41	0.62	21.408	0.35	10.3390/molecules26154456
53	Sesquiterpenoids	Juniper camphor	$C_{15}H_{26}O$	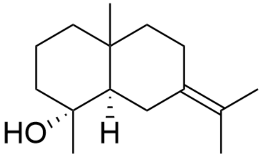	21.576	0.16	21.574	0.24	21.572	0.12	21.572	0.12	-	-	10.1016/S0040-4039(00)89530-1
54	Sesquiterpenoids	Ledol	$C_{15}H_{26}O$	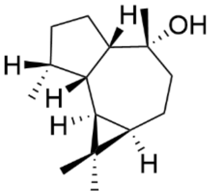	21.95	0.08	21.947	0.12	--	-	-	-	-	-	10.1016/S0031-9422(00)00291-0

55	Sesquiterpenoids	Humulene epoxide II	$C_{15}H_{24}O$	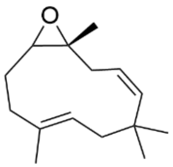	22.055	0.11	22.051	0.13	-	-	22.054	0.08	-	-	10.1016/j.phytochem.2005.09.012
56	Phenolic acids	*m*-Cresol	C_7H_8O	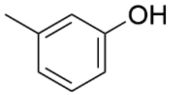	-	-	-	-	-	-	-	-	22.606	0.12	10.1016/0021-9517(83)90229-4
57	Sesquiterpenoids	Globulol	$C_{15}H_{26}O$	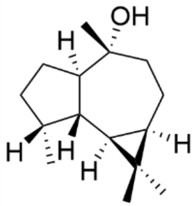	-	-	-	-	-	-	-	-	22.701	0.08	10.1016/S0031-9422(00)00291-0
58	Sesquiterpenoids	Viridiflorol	$C_{15}H_{26}O$	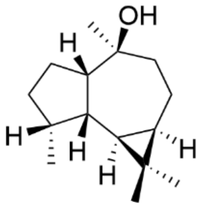	22.707	0.21	22.705	0.38	22.704	0.13	22.703	0.11	-	-	10.1016/S0031-9422(00)00291-0
59	Sesquiterpenoids	Spathulenol	$C_{15}H_{24}O$	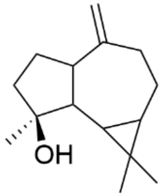	23.391	0.43	23.389	0.67	23.388	0.29	23.387	0.24	23.386	0.14	10.1016/0031-9422(95)00577-t

60	Sesquiterpenoids	T-cadinol	$C_{15}H_{26}O$	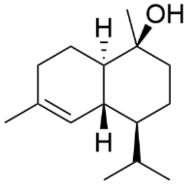	23.778	0.06	23.776	0.09	-	-	-	-	-	-	10.3390/molecules26154456
61	Sesquiterpenoids	Patchoulol	$C_{15}H_{26}O$	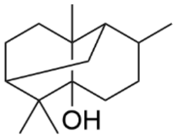	24.05	1.57	24.049	2.59	24.043	1.16	24.042	1	24.04	0.62	10.1016/j.fct.2008.06.069
62	Sesquiterpenoids	Gansongon	$C_{15}H_{22}O$	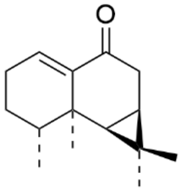	24.345	0.11	-	-	-		-	-	-	-	10.1055/s-2006-962810
63	Phenolic acids	4-Methoxybenzyl alcohol	$C_8H_{10}O_2$	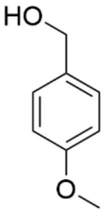	-	-	-	-	-	-	-	-	25.113	0.14	

64	Phenolic acids	Anisyl isovalerate	$C_{13}H_{18}O_3$	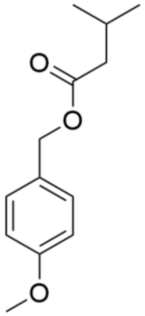	-	-	-	-	-	-	-	-	25.659	0.24	
65	Sesquiterpenoids	*E*-Valerenyl acetate	$C_{17}H_{26}O_2$	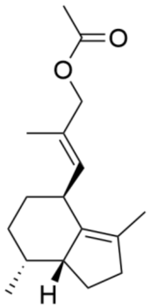	26.085	0.18	26.084	0.25	-	-	-	-	-	-	10.1016/S0031-9422(00)94517-5

- Indicates that it is not detected or the similarity match is below 85 points.

Table S2 Validation method parameters for quantification of three Sesquiterpenoids

Parameters	Nardosinone	Kanshone C	Isonardosinone
LOD (μg/ml)	0.59	1.29	0.65
LOQ (μg/ml)	1.97	4.30	2.15

Calibration curve	y = 17,955,059.65 x + 137,374.20	y = 8,250,215.37 x - 19,682.80	y = 13,003,028.02 x + 11,509.69
Mean correlation coefficient (R^2)	0.9999	0.9997	0.9996
Linear range (μg/ml)	13.4~1450.0	11.7~93.8	12.1~72.3
Accuracy (%, n=3)	98.10	101.08	99.32
Injection precision (RSD%, n=6)	0.41	1.41	1.59
Stability (RSD%)	1.13	1.45	1.47
System suitability (RSD%, n=6)	1.10	1.43	0.92

Table S3 Summary of transcriptome data sequenced

Sample	RawReads	RawBases	CleanReads	CleanBases	ValidBases	Q30	GC
Nj_A_1	48.50M	7.28G	47.85M	6.98G	95.95%	94.96%	43.23%
Nj_A_2	47.21M	7.08G	46.43M	6.77G	95.60%	94.65%	43.32%

Nj_A_3	48.07M	7.21G	47.42M	6.92G	96.02%	95.02%	43.25%
Nj_F_1	47.16M	7.07G	46.37M	6.76G	95.53%	94.70%	42.50%
Nj_F_2	47.16M	7.07G	46.38M	6.76G	95.50%	94.69%	42.51%
Nj_F_3	48.84M	7.33G	48.05M	7.04G	96.14%	94.66%	42.56%
Nj_L_1	48.17M	7.23G	47.39M	6.92G	95.79%	94.66%	43.45%
Nj_L_2	42.65M	6.40G	42.00M	6.14G	96.01%	94.81%	43.53%
Nj_L_3	47.35M	7.10G	46.56M	6.77G	95.27%	94.69%	43.48%
Nj_Rh_1	46.83M	7.02G	45.98M	6.70G	95.35%	94.36%	42.89%
Nj_Rh_2	47.92M	7.19G	47.21M	6.91G	96.13%	94.64%	42.96%
Nj_Rh_3	47.49M	7.12G	46.79M	6.84G	96.00%	94.65%	43.00%
Nj_Ro_1	49.21M	7.38G	48.59M	7.10G	96.20%	95.17%	43.83%
Nj_Ro_2	46.05M	6.91G	45.38M	6.62G	95.77%	94.69%	43.56%
Nj_Ro_3	47.55M	7.13G	46.83M	6.82G	95.62%	94.66%	43.51%
Summary	710.16M	106.5G	699.23M	102.5G			

Note: (1) raw_reads: the number of original reads; (2) raw_bases: the amount of original sequencing, i.e. the base number; (3) clean_reads: the number of clean reads after filtration; (4) clean_bases: the amount of sequencing after filtration, i.e. the base number; (5) valid_base: the percentage of effective bases; (6) Q30: the percentage of the base with the Phred value greater than 30; (7) GC: the percentage of the total number of G and C bases in clean bases.

Table S4: Statistics of NGS sequencing assembly in *N. jatamansi*

Terms	Value
Raw read count	710.16M
Processed read count	699.23M
Unigenes Generated	82772
Maximum Contig Length	14547
Minimum Contig Length	301

Average Contig Length	1169.01
Total Contigs Length	96761698
Total Number of Non-ATGC Characters	0
Percentage of Non-ATGC Characters	0
Contigs >= 500 bp	59362
Contigs >= 1 Kbp	36934
N50 value	1676

Table S5: BLAST analysis of unigenes against seven public databases

Database	Annotated_Number	300<=length<1000	length>=1000
NR	26069(98.36 %)	771(2.91 %)	25290(95.42 %)
Swissprot	23333(88.04 %)	679(2.56 %)	22646(85.45 %)
KEGG	4363(16.46 %)	240(0.91 %)	4119(15.54 %)
KOG	17091(64.49 %)	356(1.34 %)	16733(63.14 %)
eggNOG	25639(96.74 %)	741(2.80 %)	24890(93.91 %)
GO	20917(78.92 %)	652(2.46 %)	20257(76.43 %)
Pfam	24988(94.28 %)	655(2.47 %)	24333(91.81 %)

Table S6: The NjCYPs families and the members number in each family.

**family**	CYP51	CYP71	CYP72	CYP73	CYP74	CYP75	CYP76	CYP77	CYP78	CYP79

**gene number**	2	54	20	3	6	7	16	4	4	3
**family**	CYP80	CYP81	CYP82	CYP84	CYP85	CYP86	CYP87	CYP88	CYP89	CYP90
**gene number**	5	30	16	5	1	6	5	3	7	8
**family**	CYP93	CYP94	CYP96	CYP97	CYP98	CYP701	CYP704	CYP706	CYP707	CYP710
**gene number**	3	10	2	10	6	3	4	5	3	1
**family**	CYP711	CYP714	CYP716	CYP720	CYP722	CYP734	CYP735	CYP736	CYP749	
**gene number**	1	4	10	2	5	3	1	4	7	

Table S7: The FPKM values of genes co-expressed in the sesquiterpenoids biosynthesis.

**Genes**	**Tissues**				
	**A**	**F**	**L**	**Rh**	**Ro**
NjDXS1	17.6320	29.6546	9.0425	49.9603	66.9443
NjDXS2	21.5082	17.4880	15.6217	16.9924	143.2900
NjDXS3	2.9452	243.5864	2.4769	6.6050	9.7231
NjDXS4	0.0886	231.5821	1.1783	0.6968	10.6498
NjDXS5	1.8253	5.0985	6.5462	12.4020	15.8445
NjDXR1	20.1725	10.8917	31.0113	6.5850	4.9533
NjDXR2	37.5227	25.1180	28.8003	86.3609	321.4990
NjCMS	8.2224	11.0135	20.4798	17.8848	35.5445
NjCMK	37.5544	98.2633	42.2101	98.3653	178.4038
NjMCS	57.7314	93.5206	75.8496	83.0809	147.6258
NjHDS1	0.4389	2.3813	7.0435	2.8167	31.3054
NjHDS2	2.6869	4.5943	4.7814	20.8453	73.7858
NjHDS3	12.3128	13.8461	6.2316	52.0217	77.8637
NjHDS4	33.6552	30.0211	14.1325	44.1896	123.1207
NjHDS5	0.7903	0.4611	1.8007	0.3296	24.9666

NjHDR	179.5321	220.8094	209.8608	296.5107	714.7424
NjACCT1	11.1011	22.3235	17.0583	32.5168	118.4769
NjACCT2	31.8609	31.4621	26.5123	73.1669	68.7510
NjACCT3	1.7985	1.4459	0.7337	5.1870	43.4442
NjHMGS	52.5172	55.3558	57.0817	71.4145	81.9025
NjHMGR	0.0094	0.0000	0.0000	0.0219	121.0889
NjMAK	29.1147	25.1208	20.3165	57.6624	103.9055
NjPMK1	5.7895	8.9334	3.3344	14.2816	9.8769
NjPMK2	1.6301	2.9112	3.4134	3.4014	4.9567
NjMDC1	16.9273	5.1725	3.8844	17.6607	72.6946
NjMDC2	15.1069	5.1374	8.2536	28.8780	75.6287
NjIDI1	26.8354	19.5966	21.8908	98.9173	137.2960
NjIDI2	39.8726	318.3886	15.6509	95.1031	300.6993
NjFPPS1	304.4339	35.2533	55.2060	162.0209	1110.5947
NjFPPS2	31.1614	17.7111	18.4194	40.0563	33.8446
NjFPPS3	5.8702	0.3441	0.8056	6.9709	16.6501
NjTPS1	0.0000	0.0000	0.0000	4.9945	20.0400
NjTPS3	0.0139	0.0206	0.0000	78.9296	943.6391
NjTPS4	0.0474	0.0000	0.0000	0.1780	8.3349
NjTPS7	0.0012	0.0000	0.0000	12.4511	22.2858
NjTPS8	0.0073	0.0000	0.0000	36.6243	146.5837
NjTPS11	0.0079	0.0000	0.0000	23.1901	170.2432
NjTPS12	0.0113	0.0000	0.0000	20.4048	392.6237
NjTPS14	0.0000	0.0000	0.0000	1.7630	18.1748
NjTPS15	0.0760	0.0006	0.5182	4.1695	1088.5167
NjTPS18	1.3344	0.5527	1.1482	0.1506	22.4265

NjTPS20	0.0000	0.0000	0.0000	0.0120	15.3339
NjTPS21	0.0000	0.0047	0.0000	50.9706	364.0831
NjTPS22	2.7446	0.6164	1.8659	8.6942	20.2709
NjTPS25	0.0490	0.0000	0.0153	2.3263	10.4662
NjTPS26	0.1796	0.6783	1.1152	11.1567	438.1761
NjTPS27	0.0029	0.0000	0.0000	27.9838	280.3934
NjTPS30	0.1801	0.0303	0.1401	17.3488	292.5605
NjTPS33	0.0501	0.0000	0.0000	49.0292	63.7860
NjTPS35	0.0000	0.0329	0.0000	4.7901	5.8152
NjTPS38	0.0001	0.0000	0.0000	10.8583	415.2749
NjTPS40	0.2399	0.0729	1.4942	2.2327	39.0284
NjTPS41	0.0518	0.0000	0.0000	7.1480	79.8970
NjTPS43	0.0116	0.3832	0.0269	3.2411	98.7183
NjTPS46	0.0003	0.0000	0.0010	9.7312	180.5911
NjTPS47	0.0000	0.0000	0.0000	0.0205	44.0081
NjTPS48	0.0000	0.0000	0.0000	14.3107	177.6261
NjTPS53	0.0000	0.0000	0.0000	16.9831	199.4735
NjTPS58	0.0000	0.0000	0.0000	0.0000	6.3216
NjTPS59	0.0000	0.0000	0.0000	0.0000	45.9632
NjCYP71A16	0.0000	0.0000	0.0000	0.0000	26.1123
NjCYP71A8	0.4534	0.2976	0.6988	139.6183	294.9636
NjCYP71AV5	0.0101	0.0000	0.0000	0.0206	100.8097
NjCYP71AV6	0.0102	0.0000	0.0000	0.0000	55.9618
NjCYP71AV7	0.0000	0.0000	0.0000	0.0000	167.8570
NjCYP71AV8	0.0212	0.0000	0.0000	0.0641	324.6819
NjCYP71C1	20.6301	2.8401	3.1542	29.0615	107.7936

NjCYP71C5	25.6836	6.6367	7.6304	87.3240	91.8272
NjCYP71C10	1.8295	0.0011	0.0000	21.0297	322.3656
NjCYP71C11	1.6697	0.5323	0.6171	2.4707	15.8020
NjCYP71C15	2.3320	0.0544	0.0000	35.0402	393.2540
NjCYP71C17	0.0690	0.0001	0.0000	22.5164	496.7364
NjCYP71C9	0.9530	0.0114	0.0000	8.8193	226.5904
NjCYP71D1	0.2537	5.6135	0.0119	40.1767	66.4602
NjCYP71D3	1.1647	3.8464	3.3700	8.1116	15.8767
NjCYP71G1	0.0106	0.0000	0.0000	0.0000	33.4363
NjCYP71I2	0.0000	0.0000	0.0000	0.0000	24.8733
NjCYP71I3	0.0117	0.0000	0.0000	0.0000	35.3944
NjCYP71J1	0.0187	0.0000	0.0000	0.0000	42.1056
NjCYP71N1	0.0722	0.3733	0.0291	22.9742	138.5493
NjCYP76B1	0.0000	0.0000	0.0000	0.0000	22.3773
NjCYP76B2	0.0000	0.0000	0.0000	0.0120	21.5313
NjCYP76B5	2.0176	1.8244	1.2821	7.4940	161.6724
NjCYP76B6	62.7984	30.2379	22.3236	318.7901	567.8963
NjCYP76G1	1.9603	2.0681	4.1388	2.0679	16.6482
NjCYP81C1	0.0000	0.0000	0.0000	0.0000	28.7834
NjCYP81D11	15.9292	7.1385	2.0082	58.1565	71.0880
NjCYP81D3	0.3581	0.0097	0.2781	42.6602	59.1936
NjCYP81D4	33.6258	8.9887	13.9283	70.5980	97.4999
NjCYP81D5	0.7222	4.8582	2.1768	3.2830	24.8039
NjCYP81D9	5.6762	4.7668	25.0158	281.5794	210.5039
NjCYP81E1	15.5250	11.3951	12.6740	26.2306	73.9342
NjCYP81E4	11.0188	8.1907	5.8104	42.6067	47.1393

NjCYP82A2	0.6602	0.9187	0.1818	35.7119	102.7917
NjCYP82A3	6.7373	1.9872	0.2240	65.6177	54.9332
NjCYP82B2	0.0000	0.0000	0.0000	0.0000	32.2092
NjCYP82B3	0.0311	0.0000	0.0000	0.0215	138.5983
NjCYP82D45	2.3794	0.3228	3.0153	40.8834	82.0064
NjCYP82D46	1.4177	0.3168	0.5191	61.0534	38.5323
NjCYP81D7	1.8095	4.6420	8.8551	75.7420	87.9373
NjCYP706B1	0.0000	0.0000	0.0000	0.0135	70.1716
NjCYP736A223	1.3919	10.2944	1.3229	17.9127	207.2526
NjCYP711A1	0.2315	0.3567	1.7538	7.1454	29.8977

Table S8: Primers and annealing length in qRT-PCR

Gene name	F-primer(5'to3')	R-primer(5'to3')
NjTPS3	CCACCCTTTCCCAGTTCCAA	ACTCGGGATGCTCTCTATCA
NjTPS15	AGCGCGTACTACGAACCAAA	ATTTCGCATAAGCAACGCGG
NjTPS21	CGTAGGCATGGGAGTAACTG	TGTCCAACCTTATCGTCCGC
NjTPS28	GGCGGCACGTACTTCTATGT	CCAATACCGTCGATTGCTGG
NjTPS48	CATCCCTCATAGACGACACCT	TTCCGGAAGTTGGTTTTTGGC
NjTPS35	TAAGCGTCCTCGAGCCTACT	CGCCTATCACCACTATTCACT
NjCYP722A1	GATGGTGAAGCTATCAAAAGAGC	AGTCTCCCAGGTACACCAACT
NjCYP71C9	CATCCGGTGGATCGCCTTTA	GGCCACTCGAGGTGATGAAA
NjCYP706B1	TGTACTGGCGTAACATGCGT	CACACCTTCGTCCCCATTGA
NjCYP71AV8	TGCACCTTCCTCTTCCGTTG	CACATTTGTGGGGTTGTCGC
NjCYP71N1	AAATGGAGCAACCCGCTTTC	AGGACCGTACTTTTGCGAGA
NjCYP82A2	TGGATGGAAGAGCATCGACA	TGTATCACTACCTCCGGCAA
NjActin	GGACCGCCGATTTTGAGACT	AGACCCTTCCATCAGGCAAAG
