# Supplementary material for: Metabolome and transcriptome associated analysis of sesquiterpenoid metabolism in Nardostachys jatamansi
Source: Front Plant Sci. 2022 Nov 29;13:1041321. doi: 10.3389/fpls.2022.1041321 (PMC9746346; doi:10.3389/fpls.2022.1041321)
Supplement: Additional file 1 — This file includes all additional tables ( Tables S1 - S8 ) used in this manuscript. Table numbers and titles were listed as follows: [file DataSheet_1.zip › Data Sheet 2.PDF]

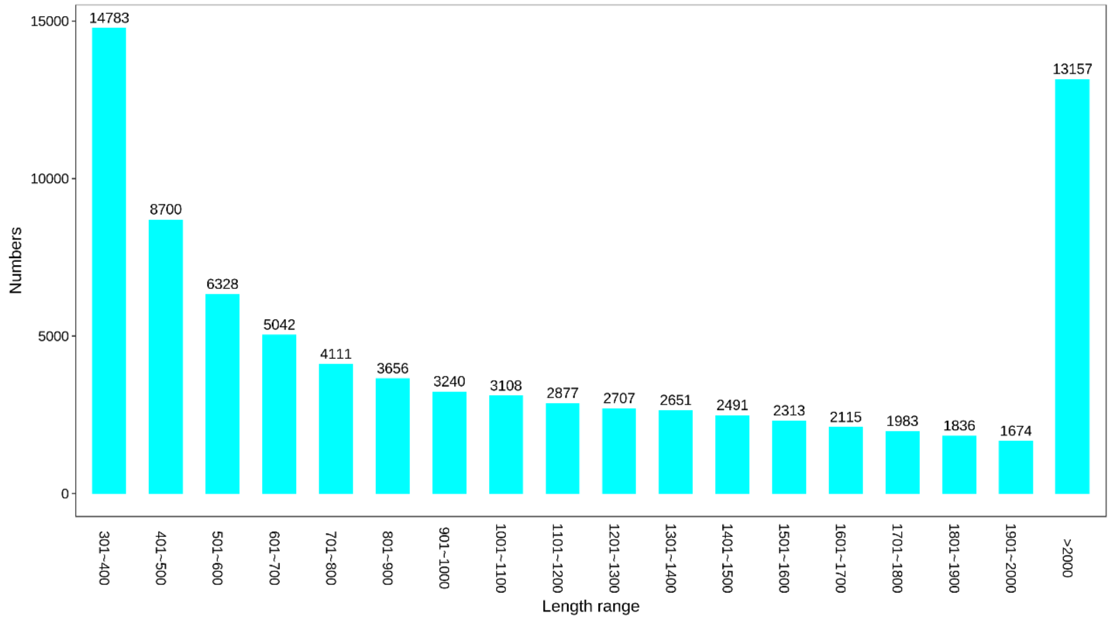

Fig. S1 Assembled sequence length distribution from Illumina HiSeq X Ten platform.

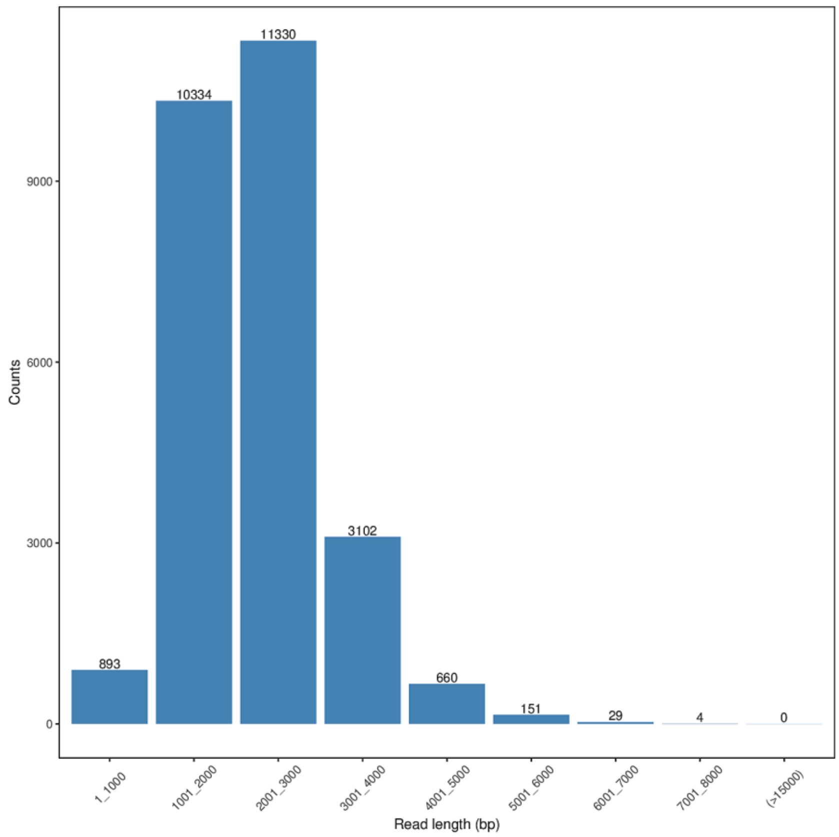

Fig. S2 Non-redundant read length distribution from Pacific Biosciences Sequel platform.

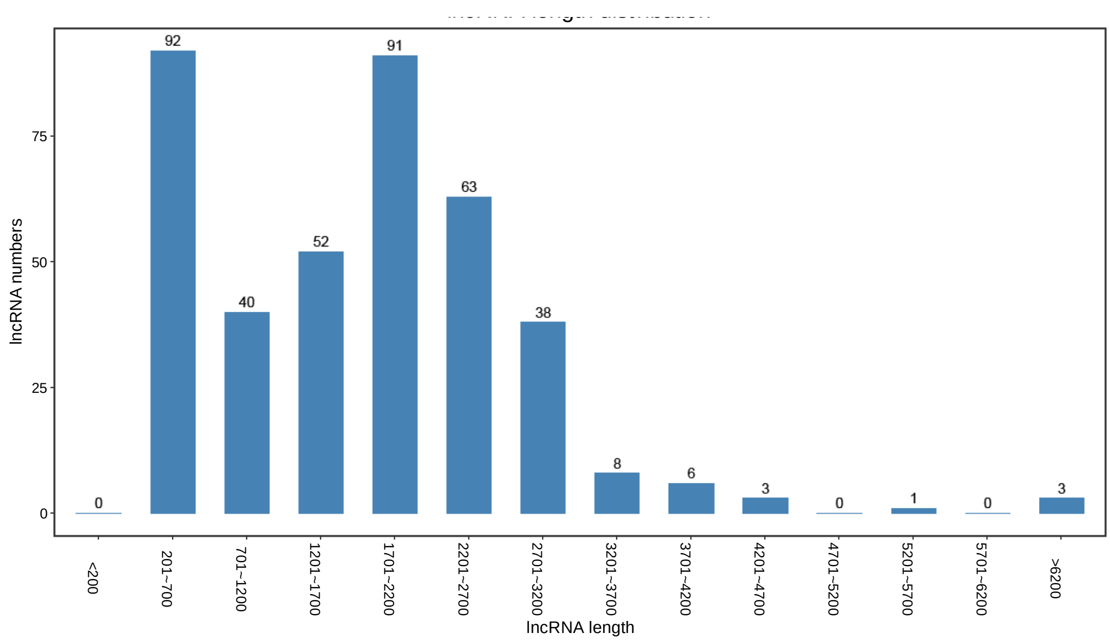

Fig. S3 Length distribution of lncRNA from Pacific Biosciences Sequel platform.

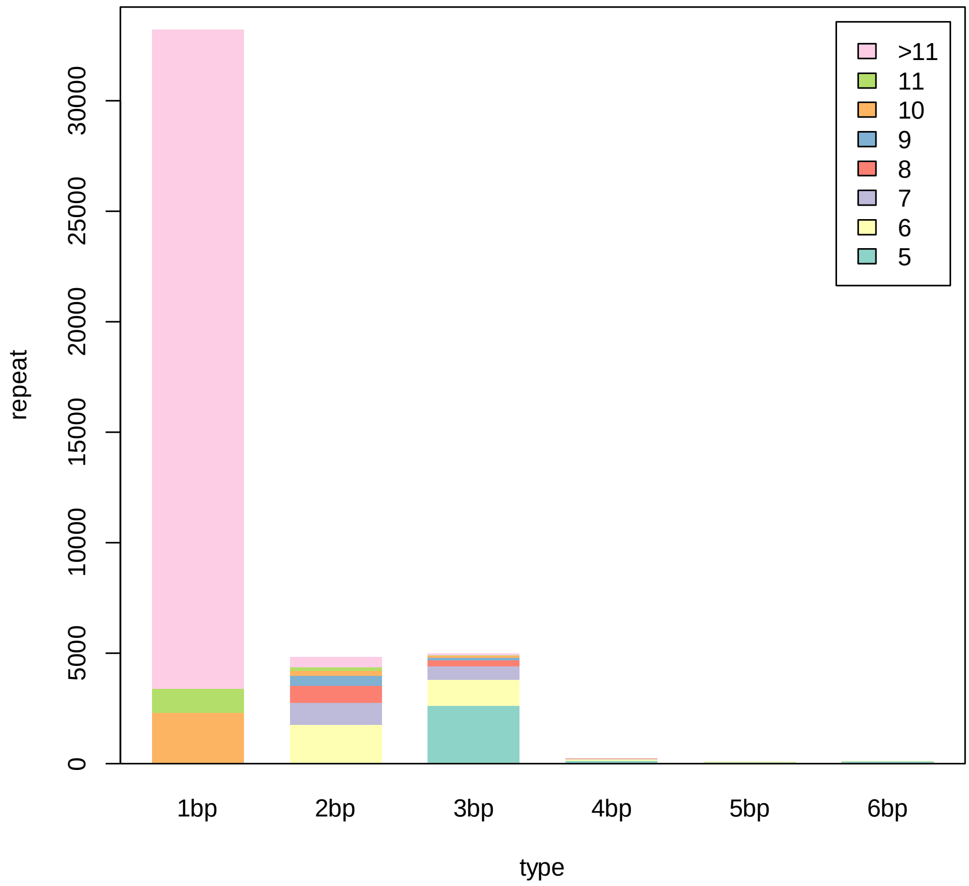

Fig. S4 SSR type statistics from Pacific Biosciences Sequel platform.

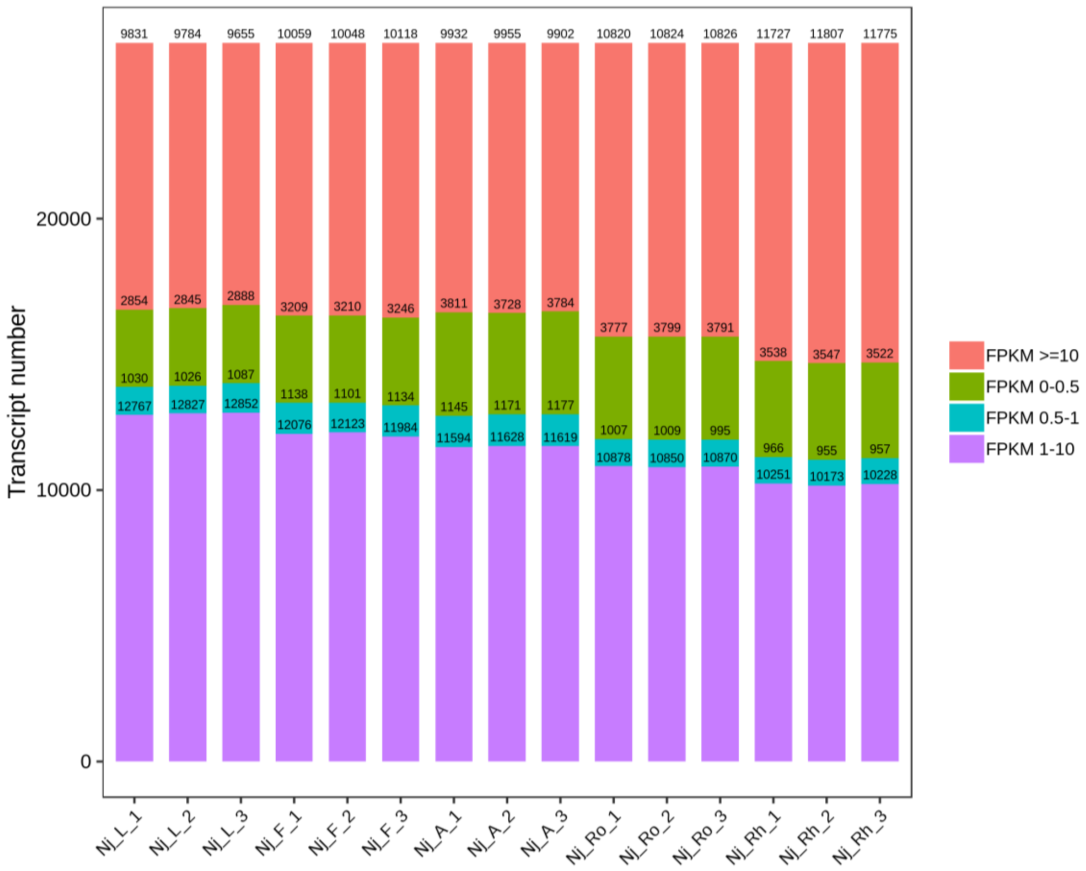

Fig. S5 The expression of unigenes obtained from SMRT sequencing in each sample.

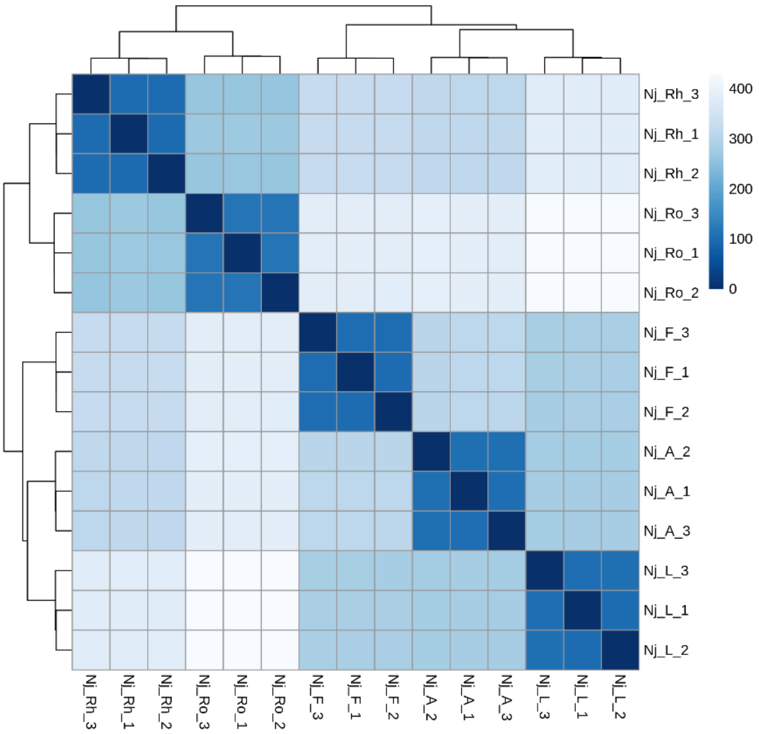

Fig. S6 Cluster map of sample-to-sample based on gene expression obtained from

SMRT sequencing.

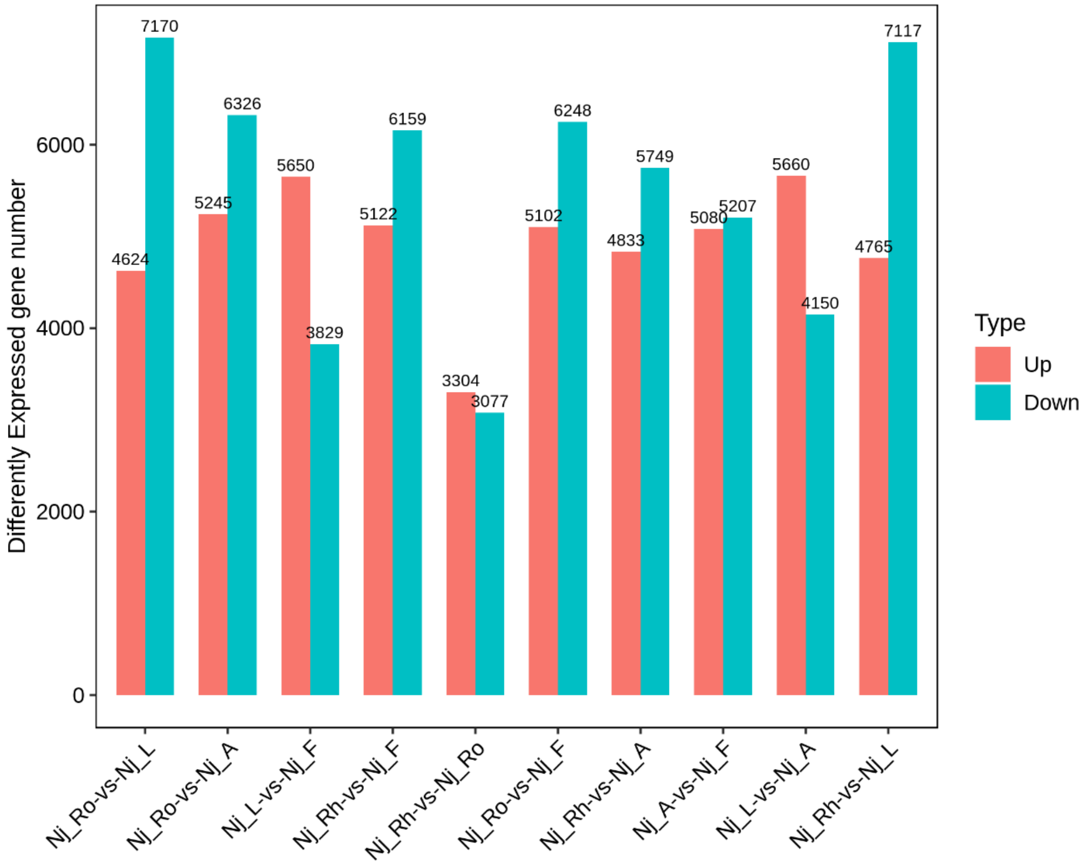

Fig. S7 The number of up-down regulated *DEGs* of the ten paired comparisons.

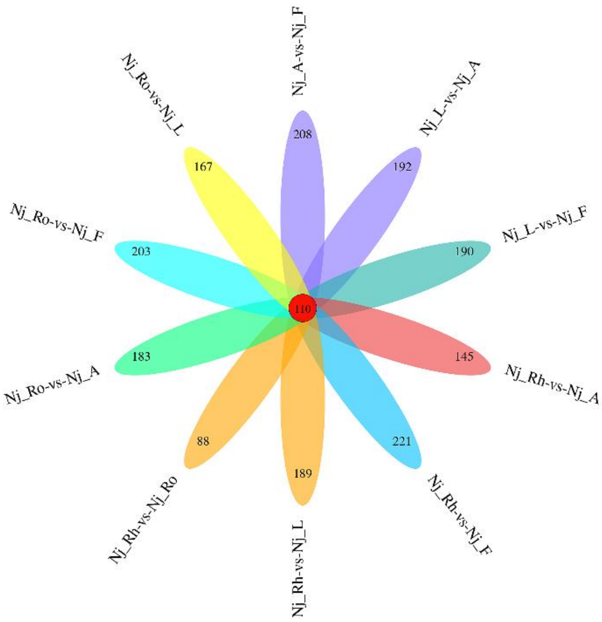

Fig. S8 Venn diagram of *DEGs* from the ten paired comparisons.

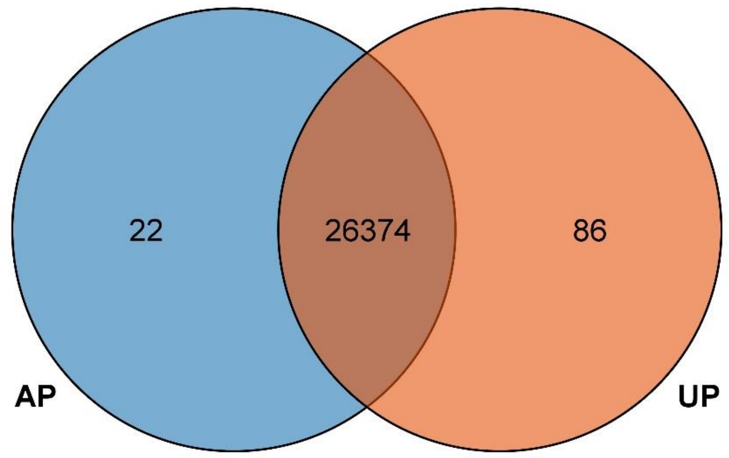

Fig. S9 Venn diagram of genes from AP and UP.

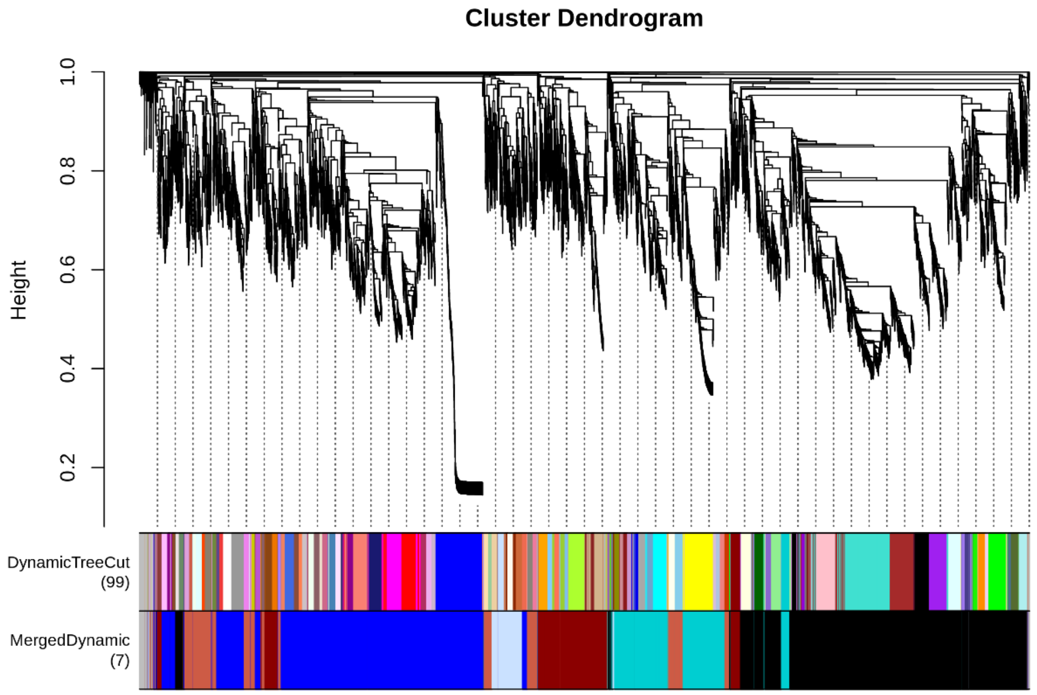

Fig. S10 Network visualization plots: Clustering dendrograms of genes with dissimilarity based on topological overlap together with assigned module colours. The same colour represents the same module, and the modules with certain correlations are merged into the same module.

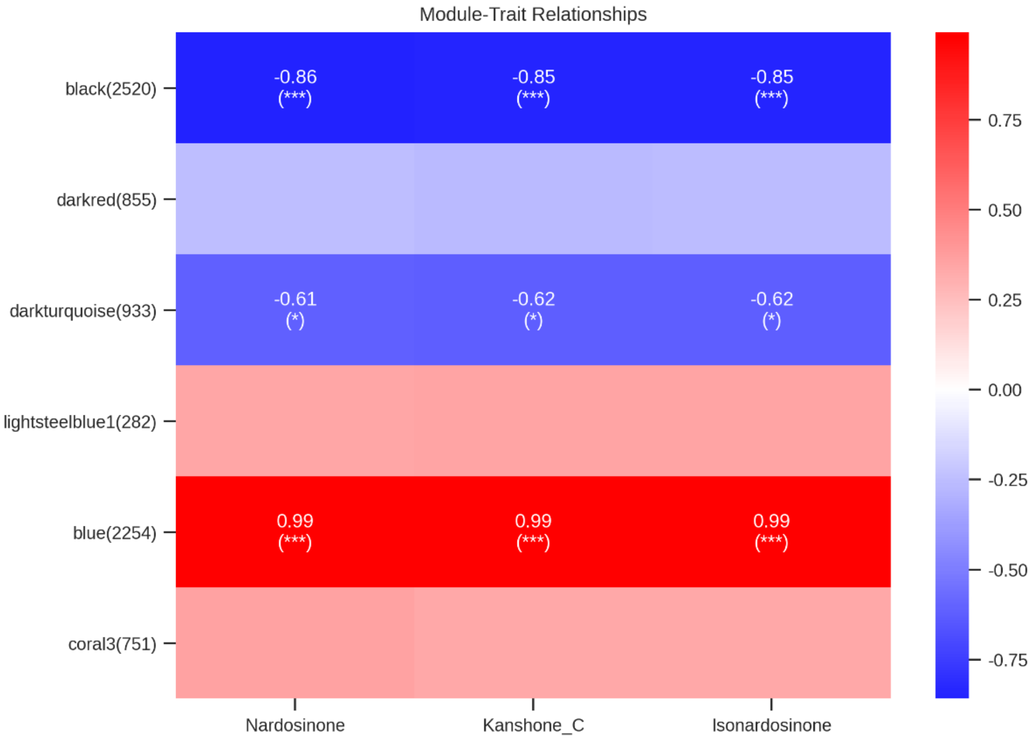

Fig. S11 Network visualization plots: Module-trait relationships.
