# Supplementary material for: Metabolome and transcriptome associated analysis of sesquiterpenoid metabolism in Nardostachys jatamansi
Source: Front Plant Sci. 2022 Nov 29;13:1041321. doi: 10.3389/fpls.2022.1041321 (PMC9746346; doi:10.3389/fpls.2022.1041321)
Supplement: Additional file 1 — This file includes all additional tables ( Tables S1 - S8 ) used in this manuscript. Table numbers and titles were listed as follows: [file DataSheet_1.zip › Data Sheet 3.pdf]

>NjTPS1
MVRILDLKPSMDAVYHWINPSPLDKNTEELIEKIRERFEKVDLSISAYDT
AWVAMVTSALRHQEPCFPGCLEWILENQKADGSWGLNLSHPSLLKDSLSS
TLACVIALQKLNVGEKHIQRGLVFIGSKKYAAVDKYQSSPIGFDINFPAM
IKYANNLGLNLPLDSAFIDLMLHYRDIELQRCKPRDLAYFAEGLVGESSY
DWEEIIKNGQGSNGSLFNSPAATAVALINTRNDKCYDYLNSLLKINHGKG
VPTIYPFHLYTRLCMVDTIDRLGINRHFANELKVILDETYRCWLQKSEEI
YSDVSCCSMAFRLLRKNGYHVSSDALEEFIDEEHFFSTLSPQFRNTSTVV
QLYRASQMSFFQKEPVLDKINEWTTNFLRHQLLNHEIYDDDLLREVNYAF
EYPIDNMPRLTNRRGIELYNTDSFRMLKTSYRCCSVNNEEFLVLSQQEFN
KTQQIHLEEYKQVEEWLKKHRIGGLEFEWHMVTSSYFLAASGYIIPELSD
ARIIWAKVSVLGTIIDDLFDIDGTQEELLNLLHLIQNWDGNPNISSLDYS
SERTEIMFLALTEIINEQAAIGLIRQGRCIKKELIQIWQNFCKSCFKEVE
WWANKSTPTLDEYLANGCQTIGIGLWSITFYCVGIQLSQDTLISEEYQII
YKHLGLIMRLFNDYQGVIDERDKVQRKMNGCLLLVSLSGGALTVEEARTE
VRKMIDISRKEVLRMMLTASSTTQKILMESFFCFHQQAYYLYTGNDEYRI
PSKKGLNDINRLLYEPLNLI
>NjTPS2
MVRILDLKPRMDAVYHWINPSPLDKNTEELIEKIRERFEKVDLSISAYDT
AWVAMVTSALRHQEPCFPGCLEWILENQKADGSWGLNLSHPSLLKDSLSS
TLACVIALQKWNVGEKHIQRGLVFIGSKKYAAVDKYQSSPIGFDINFPAM
IKYANNLGLNLPLDSAFIDLMLHNRDTKLQRCKPRDLAYFAEGLVGESSY
DWEEIIKNGQGSNGSLFNSPAATAAVLINTRNDKCYDYLNSLLKINHGKG
VPTIYPFHLYTRLCMVDTIDRLGINRHFANELKVILDETYRCWLQKSEEI
YSDVSCCSMAFRLLRKNRYHVSSDALEEFIDEEHFFSTLSPQFTNTSTVV
QLHRASQMSFFQKEPVLDKINEWTTNFLRHQLLNHEIYDDHLLREVKYAF
EYPIDNLPRLTNRRAIELYNIDSFRMLKTSYRCCSVNNEDFLVLSQQEFT
RTQQIHLEEYKQVEEWLKKHRIGGLEFEWHMVTCAYFIGASSYIIPELSD
VRIMWAKGCVVATIIDDLFDIDGTQEELLNIVHLIQNWDGNSNISSLDYS
SERTEIMFLALTEIINEQAAIGLIRQGRCIKKELIQVWQNFCKSCFKDVE
WWANKSTPTLDEYLANGCQTSGIGLWSTTFYFVGIQLSQDTLISEEYQIM
YKHIGLIMRLFNDYQGVMDERDKVERKVNGCLLLVSLSGGALTIEEARTE
VRKMIENSRKEVLRMMLTASSATQKILMESFFCFHQQAYYLYTGNDKYRI
PSKKGLKDINGLLYEPLNLLPSYIREDKFA
>NjTPS3
MDSYLNASSAPPPKKNMQEPVRPIANYHPSVWGNQFLKYASNPKQSDSGA
EEQHEQLKEALRKKLVVNVANERAGEQLKLIDAIQRLGVAYQFENEIDVV
LNNQLQLLNNEDDDLHMVSLRFRLLRQHGHNVSCGVFGKFKDIEGRFKEC
LMDDVRGLLSLYESTHMRVHKEDILEEALEFTTTHLEQVVKSPLSGSVLA
SQVVHALNMPIRKGLTRIEARHFIPIYQQDESHDETLLKFAKLDFNMLQK
VHQREVADITMWWKDLNVSEKLPYARDRAVECYFWILGVYFEPQYSRARR
ILTKVICMTSLIDDTYDSYGTFEELILFTDAIQRWDVNAKNQLPEYMRHI
FGELLDVYGAMEEELSKEGISYRVDYAKQIMIQLVTAYNHEAIWYHDGYV
PTLEEYLEVALVSCGYIMAATTSFVGMGVKAVPKQAFDWVSSNPLMVQAS

SIINRLTDDRVGHELEQQRGHVASGVECYMKQHNATEEEVLVEFNKRITS
AWKDMNQECLHPFPVPIHLLERVLNLARFMNIFYKDEDCYTHSNTRMKGI
ITSILIESIPS
>NjTPS4
MDSYLNASSAPPPKKNMQEPVRPIANYHPSVWGNQFLKYASNPKQSDGGA
EEQHEQLKEALRKKLVVNVANERAGEQLTLIDAIQRLGVAYQFETEIDVV
LNNQLQLLNNQDDDLHMVSLRFRLLRQHGHNVSCGVFGKFKDIEGRFKEC
LMDDVRGLLSLYESTHMRLHKEDILEEALEFTTTHLEQVVKSPLSGSVLV
SQVVHALNMPIRKGLTRIEARHFIPIYQQDESHDETLLKFAKLDFNMLQK
VHQREVADITMWWKDLNVSEKLPYARDRAVECYFWILGVYFEPQYSRARR
ILTKVICMTSLIDDTYDSYGTFEELILFTDAIQRWDVNAKNQLPEYMRHI
FGELLDIYGAMEEELSKEGISYRVDYAKQIMIQLVTAYNHEAIWYHDGYV
PTLEEYLEVALVSCGYIMAATTSFVGMGVTAVPKQAFDWVSSNPLMVQAS
SIINRLTDDRVGHELEQQRGHVASGVECYMKQHNATEEEVLVEFNKRITS
AWKDMNQECLHPLPVPIHLLERVLNLARFMNIFYKDEDCYTHSNTRMKGI
ITSILIESIPS
>NjTPS5
MAFSILSNFHSSTLIPRPLVLPPSQNSIKRTLTGPKFSKFAKCLALVSPS
THEKSIERREANYHPSIWDDDYVQSLTSIYQGETCTKRVDKLKSDVREML
INKAESFMYKLELVDTLQRLGIYYHFKIEIKEILESIYNHNICSTEDLYG
TSLKFRLLRQHGYHVPQEVFNIFTDESGKFKTYLSEDTKAMLYLYEASYL
STRGEHIMDEAREFASNHLKEYLKNNIDNNNFLEKLVIHSLELPLQWRVV
RLEARWFIDMYENKQDMNPTLLQLAKLDYNMVQAVHLEDLKHLSRWWKTT
KLGENLSFARDRLVENFLWTVEGNFHPQSQDYRRLVTMANSFITVIDDVY
DVYGTLDELRIFTDVIERWDVNAIDQLPDYMKICCLALFNSINDVAYNVL
TEQNFNIIPYLKKAWASLCKCYLVEAEWYNNAYIPTLDEYLENAWISISA
PAILVHAYSSILNPLTKDGFEFLESHPDLIRSASMILRLSDDLGTSKAEL
ERGDNLKSIQCYMHETGVSEEKAREHIRYLISETWKKMNEDRVIKDYPFN
EIFVDTTLNLARMSQCMYQHGDGHGIETRETKDRVLSLLINPIPLI
>NjTPS6
MSLTIFSSLKFVTSSTQLPSNWRIRQTQFIKCIPDDTRTCDQAIAMPRRL
ANYHPSIWEDDYVQSFGNNYTGESWSKRASELKEEVRGMFGKVEDSLMKV
ELIDTLQRLGISYQFEHEIKNTLENIYNDHYRSERWNNKEDYNLFAIALE
FRILRQHGYNVTQEIFNKFKDDHGNFKSSLCQDIKGMLYFYEASFLSIRG
ESILDDARDFTTKTLEEYLKNNKLVINNLDEILDLLVDHALELPLHWRML
RLEARWFIDVYERKQDVDTTLLEFAKIDFNMVQASHQEELKQMSRWWKST
YLGEKLRFARDRLMENYFWTTGVMFEPEYEYSRRMSTKINSIVTIIDDIY
DVYGSLQELQLFTNAVQRWDMNAMDELPDYMKICFLALYNSTNEMAYNTL
VQKGVYVISPLTKAVYKIFLTPQSSILKDSFYMKTQFWRRFRHAGKCQIN
VGHFSVNLEKCRHKFFRTFW
>NjTPS7
MDSYLNASSAPPPKKNMQEPVRPIANYHPSVWGNQFLKYASNPKQSDGGA
EEQHEQLKEALRKKLVVNVANERAGEQLKLIDAIQRLGVAYQFENEIDVV
LNNQLQLLNNEDDDLHMVSLRFRLLRQHGHNVSCGVFGKFKDIEGRFKEC

LMDDVRGLLSLYESTHMRVHKEDILEEALEFTTTHLEQVVKSPLSGSVLA
SQVVHALNMPIRKGLTRIEARHFIPIYQQDESHDETLLKFAKLDFNMLQK
VHQREVADITMWWKDLNVSEKLPYARDRAVECYFWILGVYFEPQYSRARR
ILTKVICMTSLIDDTYDSYGTFEELILFTDAIQRWDVNAKNQLPEYMRHI
FGELLDVYGAMEEELSKEGISYRVDYAKQIMIQLVTAYNHEAIWYHDGYV
PTLEEYLEVALVSCGYIMAATTSFVGMGVKAVPKQAFDWVSSNPLMVQAS
SIINRLTDDRVGHELEQQRGHVASGVECYMKQHNATEEEVLVEFNKRITS
AWKDMNQECLHPFPVPIHLLERVLNLARFMNIFYKDEDCYTHSNTRMKGI
ITSILIESIPS
>NjTPS8
MDSYLNASSAPPPKKNMQEPVRPIANYHPSVWGNQFLKYASNPKQSDGGA
EEQHEQLKEALRKKLVVNVANERAGEQLTLIDAIQRLGVAYQFETEIDVV
LNNQLQLLNNQDDDLHMVSLRFRLLRQHGHNVSCGVFGKFKDIEGRFKEC
LMDDVRGLLSLYESTHMRLHKEDILEEALEFTTTHLEQVVKSPLSGSVLA
SQVVHALNMPIRKGLTRIEARHFIPIYQQDESHDETLLKFAKLDFNMLQK
VHQREVADITMWWKDLNVSEKLPYARDRAVECYFWILGVYFEPQYSRARR
ILTKVICMTSLIDDTYDSYGTFEELILFTDAIQRWDVNAKNQLPEYMRHI
FGELLDIYGAMEEELSKEGISYRVDYAKQIMIQLVTAYNHEAIWYHDGYV
PTLEEYLEVALVSCGYIMAATTSFVGMGVTAVPKQAFDWVSSNPLMVQAS
SIINRLTDDRVGHELEQQRGHVASGVECYMKQHNATEEEVLVEFNKRITS
AWKDMNQECLHPLPVPIHLLERVLNLARFMNIFYKDEDCYTHSNTRMKGI
ITSILIESIPS
>NjTPS9
MVRILDLKPSMDAVYHWINPSPLDKNTKELIEKIRERFEKVDLSISAYDT
AWVAMVTSALRHQEPCFPGCLEWILENQNADGSWGLNLSHPSLLKDSLSS
TLACVIALQKWNVGEKHIQRGLVFIGSKKYAAVDKYQSSPIGFDINFAAM
IKYANNLGLNLPLDSAFIDLMLHNRDTKLQRCKPRDLAYFAEGLVGESSY
DWEEIIKNGQGSNGSLFNSPAATAAALINTRNDKCYDYLNSLLKINHGKG
VPTIYPFHLYTRLCMVDTIDRLGINRHFANELKFILDETYRCWLQKSEEI
YSDVSCCSMAFRLLRKNRYHVSSDALEEFIDEEHFFSTLSPQFTNTSTVV
QLHRASQMSFFQKEPVLDKINEWTTNFLRHQLLNHEIYDDHLLREVKYAF
EYPIDNLPRLTNRRAIELYNIYSFRMLKTSYRCCSVNNEDFLVLSQQEFN
KTQQIHLEEYKQVEEWLKKHRIGGLEFEWHMVTCAYFIGASSYIIPELSD
VRIIWAKGCVVATIIDDLFDIDGTQEELLNIVHLIQNWDGNSNISSLDYS
SERTEIMFLALTEIINEQAAIGLIRQGRCIKKELIQVWQNFCKSCFKDVE
WWANKSTPTLDEYLAEWT
>NjTPS10
MNTCIQALSPPPPTKAIQLRPIANFHPSIWGNYFLKYASDHHTQSDDGTD
EQHGQLKEDARKKLVVNDERAGEQLMLIDAIQRLGVAYHFQTEIDVVLNN
QLLKFNEDDDLYMVSLRFRLLRQQGHHVSSGVFEKFKDVEGRFKEILIND
VRGLLSLYESTHMRVHKEEILEEALQFTTTHLEHVVKASLTDTILLSQVV
HALNMPIRKGLTRIEARNYIPIYQQDKSHDETLLKFAKLDFNMLQKVHQR
ELGDITRWWKDLNVAEKLPYARDRLVEGYFWILGVYFEPRYSRARKILTK
VFSLTSLIDDTYDSYGTFEELILFTDAVQRWDVNAKNQLPEYMRHVYGEL

LDVYSAMEEELLKEGISYRVEYAKQAMKQLVRAYNDEAIWYHKDYVPTLE
EYLKVALVSCGYIILATTSFVGMGVSDVTKQDFDWVSSNPLIVQASSVIC
RLTDDDVGHEFEQERGHVASAVECYMKQHNATKEEALVEFKKRITNAWKD
MNRECLHPLPVPMHLLERVPNFARFMYLFYKGEDCYTHSETRMKSFITSL
LVESAPN
>NjTPS11
MDSYLNASSAPPPKKNMQEPVRPIANYHPSVWGNQFLKYASNPKQSDGGA
EEQHEQLKEALRKKLVVNVANERAGEQLTLIDAIQRLGVAYQFETEIDVV
LNNQLQLLNNQDDDLHMVSLRFRLLRQHGHNVSCGVFGKFKDIEGRFKEC
LMDDVRGLLSLYESTHMRLHKEDILEEALEFTTTHLEQVVKSPLSGSVLA
SQVVHALNMPIRKGLTRIEARHFIPIYQQDESHDETLLKFAKLDFNMLQK
VHQREVADITMWWKDLNVSEKLPYARDRAVECYFWILGVYFEPQYSRARR
ILTKVICMTSLIDDTYDSYGTFEELILFTDAIQRWDVNAKNQLPEYMRHI
FGELLDIYGAMEEELSKEGISYRVDYAKQIMIQLVTAYNHEAIWYHDGYV
PTLEEYLEVALVSCGYIMAATTSFVGMGVTAVPKQAFDWVSSNPLMVQAS
SIINRLTDDRVGHELEQQRGHVASGVECYMKQHNATEEEVLVEFNKRITS
AWKDMNQECLHPLPVPIHLLERVLNLARFMNIFYKDEDCYTHSNTRMKGI
ITSILIESIPS
>NjTPS12
MDSYLNASSAPPPKKNMQEPVRPIANYHPSVWGNQFLKYASNPKQSDSGA
EEQHEQLKEALRKKLVVNVANERAGEQLKLIDAIQRLGVAYQFENEIDVV
LNNQLQLLNNEDDDLHMVSLRFRLLRQHGHNVSCGVFGKFKDIEGRFKEC
LMDDVRGLLSLYESTHMRVHKEDILEEALEFTTTHLEQVVKSPLSGSVLA
SQVVHALNMPIRKGLTRIEARHFIPIYQQDESHDETLLKFAKLDFNMLQK
VHQREVADITMWWKDLNVSEKLPYARDRAVECYFWILGVYFEPQYSRARR
ILTKVICMTSLIDDTYDSYGTFEELILFTDAIQRWDVNAKNQLPEYMRHI
FGELLDVYGAMEEELSKEGISYRVDYAKQIMIQLVTAYNHEAIWYHDGYV
PTLEEYLEVALVSCGYIMAATTSFVGMGVKAVPKQAFDWVSSNPLMVQAS
SIINRLTDDRVGHELEQQRGHVASGVECYMKQHNATEEEVLVEFNKRITS
AWKDMNQECLHPFPVPIHLLERVLNLARFMNIFYKDEDCYTHSNTRMKGI
ITSILIESIPS
>NjTPS13
MMFSSRYALPLGHNPLIRIPFSSSSPSHSSNFIASTHSLGAKGIHSNSIS
IFPSVPIKSINLDCTRVSQRINQSIEIDNDDYDEVEACKNPPPPAPEVEK
CIKEIKAILGRMEGGELTTSAYDTAMVALVKDINGLIQRARKLEIEVPND
SHPILKEIYAKRNLKLSKIPEEIFHTEHTTLLFSTEGLEDLEMERVIKLQ
CPDGSMLYSPASTAHAFIHTKDLKCLTYLTQVVDKFNGRVPYIYPVDMFA
RNWAIDQIQRLGISRYFVAEIDELVSYVSRYWDPKRGAYSISNSPFHNID
DTALCFRNLRLHGYQISPDAFWHFKYGDAFCCFPRQSSESVTAIFDLLRA
SQLQFPGEKVLDETKNFSYKFLREREANNTLIDKWVLLKDLPGEVKYALD
MPWYANLPRVESRFYIEQYGGEEDVWIGKTLYQMKEVNNNTYLKLAKLDY
NLCQEQHQLEWLHMLRWYKKNNLMEFGISERMVLLDYFLATANIFELERS
NERLGWAKSSILLHIVSTYINKHLTSNQQKLSFINTFKTHTINALHKIST
NSNSVEDRVISLVCKTLYQQSVHVFNACGRDIHRPLFHAWEIWLMTWQEE

DVNHVDSQSSVVLLATTINMFGGRLVTDQISSHPQYHRLTQLIAEICNHL
HPHHVSDDEYCLDRKKIITVEIESNMQELLQLVLQKSSDVDRINCNIKQT
FLAIAKTAYYAAYCSSATINMHVHNVLFQPVI
>NjTPS14
MDSYLNASSAPPPKKNMQEPVRPIANYHPSVWGNQFLKYASNPKQSDGGA
EEQHEQLKEALRKKLVVNVANERAGEQLKLIDAIQRLGVAYQFENEIDVV
LNNQLQLLNNEDDDLHMVSLRFRLLRQHGHNVSCGVFGKFKDIEGRFKEC
LMDDVRGLLSLYESTHMRLHKEDILEEALEFTTTHLEQVVKSPLSGSVLA
SQVVHALNMPIRKGLTRIEARHFIPIYQQDESHDETLLKFAKLDFNMLQK
VHQREVADITMWWKDLNVSEKLPYARDRAVECYFWILGVYFEPQYSRARR
ILTKVICMTSLIDDTYDSYGTFEELILFTDAIQRWDVNAKNQLPEYMRHI
FGELLDVYGAMEEELSKEGISYRVDYAKQIMIQLVTAYNHEAIWYHDGYV
PTLEEYLEVALVSCGYIMAATTSFVGMGVTAVPKQAFDWVSSNPLMVQAS
SIINRLTDDRVGHELEQQRGHVASGVECYMKQHNATEEEVLVEFNKRITS
AWKDMNQECLHPFPVPIHLLERVLNLARFMNIFYKDEDCYTHSNTRMKGI
ITSILIESIPS
>NjTPS15
MSIIIATNGTEHPIFRPLANFPPSLWGNLFTSFSMDNQAREIYAKEHEGL
KEKVRVMLLDTTNYKISEKINFINTVERLGVSYHFEKEIEELLHQMFDAH
SKLLDDIQEFDLFTLGIYFRILRQHGYKISCDVFNKLKDSNGEFKDELKD
DVNGMLSLYEATHVRTHGENILDEALIYTKAQLESMAAASLSPFLAKQVK
HALMQALHKGIPRIEARNYISVYEEDPNKNDLLLRFSKIDFNLVQMIHKQ
ELCDTFRWWKDLEFESKLSFARNRVVEAYLWTLSAYYEPKYSSARIILVK
LMVIISVTDDTYDAYGTLDELQLFTDAVQRLDMSSINQLPDYMKTIYKAL
LDLFDEIEDRLSKHETDHSYRVAYAKYVYKEIVRCYDMEYKWFNKNYVPA
FEEYMQKALVTSGNRLLITFSFLGMDEVATIQAFEWVKSNAKMIVSSNKV
LRLIDDIMSHEEEDERGHVATGIECFVKEHGLTREEVIVEFHKRIDDAWK
DINEEFITPNNLPIEILTRVLNLTRIGDVVYKYDDGYTHPEKALKDHIIS
LFVDPVSI
>NjTPS16
MSLTIFSSLKFVTSSTQLPSNGRIRQTQFIKCIPDDTRTCDQAIAMPRRL
ANYHPSIWEDDYVQSFGNNYTGESWSKRASELKEEVRGMFGKVEDSLMKV
ELIDTLQRLGISYQFEHEIKNTLENIYNEHYRSERWNNKEDYNLFAIALE
FRILRQHGYNVPQEIFNKFKDDHGNFKSSLCQDIKGMLYFYEASFLSIRG
ESILDDARDFTTKTLEEYLKNNKLVINNLDEILDLLVDHALELPLHWRML
RLEARWFIDVYERKQDVDTTLLEFAKIDFNMVQASHQEELKQMSRWWKST
YLGEKLRFARDRLMENYFWTTGVMFEPEYEYSRRMSTKINSIVTIIDDIY
DVYGSLQELQLFTNAVQRWDMKAMDELPDYMKICFLALYNSTNEMAYNTL
VQKGVYVISPLTKAWADLCKSYLLEARWYYSGYTPTLEEYMENAWISISA
PLTLVNAYLLVTNKLTTNSLECLKDYPNIIRWSSMILRFANDLGTSADEL
KRGDNPKSIQCYMHETGTVSEIEAREHIKHLISELWKKLNEEVYHHHHCR
RQNLDSPFDEVLAMNLARMAQCMYQHGDGHGIEDQETKDPKRKKNPKNAQ
RKR
>NjTPS17

MSLTIFSSLKFVTSSTQLPSNGRIRQTQFIKCIPDDTRTCDQAIAMPRRL
ANYHPSIWEDDYVQSFGNNYTGESWSKRASELKEEVRGMFGKVEDSLMKV
ELIDTLQRLGISYQFEHEIKNTLENIYNEHYRSERWNNKEDYNLFAIALE
FRILRQHGYNVPQEIFNKFKDDHGNFKSSLCQDIKGMLYFYEASFLSIRG
ESILDDARDFTTKTLEEYLKNNKLVINNLDEILDLLVDHALELPLHWRML
RLEARWFIDVYERKQDVDTTLLEFAKIDFNMVQASHQEELKQMSRWWKST
YLGEKLRFARDRLMENYFWTTGVMFEPEYEYSRRMSTKINSIVTIIDDIY
DVYGSLQELQLFTNAVQRWDMKAMDELPDYMKICFLALYNSTNEMAYNTL
VQKGVYVISPLTKAWADLCKSYLLEARWYYSGYTPTLEEYMENAWISISA
PLTLVNAYLLVTNKLTTNSLECLKDYPNIIRWSSMILRFANDLGTSADEL
KRGDNPKSIQCYMHETGTVSEIEAREHIKHLISELWKKLNEEVYHHHHCR
RQNLDSPFDEVLAMNLARMAQCMYQHGDGHGIEDQETKDRVLALLINPIN
>NjTPS18
MISSSSVRSLYFPKTNIITSKVPSLLINNINVPSNNSSIRACISMSSLPV
SKSTSSSTAAPLIRDNGSLLKFITQTPQVEVDESKRIMELVETTRRTLRK
ASSDPTDKMKLIDSLQRLGLNHHFEEDINVVLQEFANEQKNTNEDLFTTS
LRFRLLRHNGYNVTPDIFNKFTEKNGKFKESLSEDTIGILSLYEASYLGA
KGEEILSEAIKFSESKLRESAGHVAPQIRRQILQSLELPRHLRMARLESR
RFIEEDYSKEIGCDLSLLELAKLDFNYVQSLHQMELAEISRWWKQLGLAD
KLPFARDRPLECFLWTVGLLPEPKHSECRIELAKTIAVLLVIDDIFDTYG
SFDQLVLFTNAIRRWDLDAMEELPEYMKICYMALYNTTNEICYKVLKENG
WSVLPYLKRTWIDMIEGFMVEAEWLNRGQVPNLEEYIENGVTTAGSYMAL
VHIFFLIGDGVTDDNVKLLLDPYPKLFSSAGRILRLWDDLGTAKEEQERG
DVSSSIQLYMKENNISSESEGRKQIIEIIHNLWKDLNGELIGSNAMPLPI
IKTSFNMARTSQVVYQHEDDSYFSSVDNYVQSLFFTPI
>NjTPS19
MISSSSVRSLYFPKTNIITSKVPSLLINNINVTSNNSSIRACISMSSLPV
SKSTSSSTAAPLIRDNGSLLKFITQTPQVEVDESKRIMELVETTRRTLRK
ASSDPTDKMKLIDSLQRLGLNHHFEEDINVVLQEFANEQKNTNEDLFTTS
LRFRLLRHNGYNVTPDIFNKFTEKNGKFKESLSEDTIGILSLYEASYLGA
KGEEILSEAIKFSESKLRESAGHVAPQIRRQILQSLELPRHLRMARLESR
RFIEEDYSKEIGCDLSLLELAKLDFNYVQSLHQMELAEISRWWKQLGLAD
KLPFARDRPLECFLWTVGLLPEPKHSECRIELAKTIAVLLVIDDIFDTYG
SFDQLVLFTNAIRRWDLDAMEELPEYMKICYMALYNTTNEICYKVLKENG
WSVLPYLKRTWIDMIEGFMVEAEWLNSGQVPNLEEYIENGVTTAGSYMAL
VHIFFLIGDGVTDDNVKLLLDPYPKLFSSAGRILRLWDDLGTAKEEQERG
DVSSSIQLYMKENNISSESEGRKQIIEIIHNLWKDLNGELIGSNAMPLPI
IKTSFNMARTSQVVYQHEDDSYFSSVDNYVQSLFFTPI
>NjTPS20
MPVQQQVVIRPAITFPPSVWGDQFLVYEQQEDEDITHKVIDDLKEEVTKE
ISASLNDPSEHLNLLKLVDTIQRLGIAYYFEKEIKEALQHIYDSYGDNWT
GAGTSLWFRLMRQQGFLVPSDIFNKYKDKEGAFNYESLNNDVQELLELYE
ATYVSMPGEVILDDALLFTRNRLHEIAKESNSKQIQDALKQPLHKRLPRL
EAIRYIPFYESQTSHNHSLLKLAKLGFNMLQSQHKKELSEISKWWKAFDV

PNNLPFARDRLVECYFWATGMYFEPQYSQSRMFLAKVSAIGTTLDDTYDA
YGTYEELLLLTEAFQRWPNACLDELPETMKLIYRMQVNLYEKMGEILAKM
GKGHHLNYIEEAMQEYIRSYMTEATWFHEGYMPTVEEHIELTYISTAYKY
MITAGFAAMGDLITDETFKWVFTNPPIVRACCVICRIMDDVLANKEEQER
KNVPSAVECYMTQFNVTDEQHVRDLLNAKVGDAWKEINKESLVSKDVERP
IIMMIINLARVMDVLYKNKDEFKYGGEQFRTNIKALFVDAIII
>NjTPS21
MDSYLNASSAPPPKKNMQEPVRPIANYHPSVWGNLFLKYASNPEVKQSDG
GAEEQHEQLKEALRKKLVVNVANERAGEQLKLIDAIQRLGVAYHFETEID
VILNNQLQLLNNQDDDLHMVSLRFRLLRQHGHNVSCGVFGKFKDIEGRFK
ECLMDDVRGLLSLYESTHMRVHKEEILEEALEFTTAHLEQVDKSPLSGSV
LASQVVHALNMPIRKGLTRIEARHFIPIYQQDESHDETLLKFAKLDFNML
QKVHQREVADATMWWKDLNVSEKLPYARDRVVECYFWILGVYFEPKYSRA
RRMLTKVICLASLIDDTYDSYGTFEELILFTDAIERWDVNAKNQLPEYMR
HIFEELLDVYGAMEEELSKEGISYRVDYAKQIMKQLVTAYNHEAIWYHDG
YVPTLEEYLEVALVSCGYMMLATTSFVGMGVTAVTKQALDWVSSKPLMVQ
ASSIINRLADDKVGHEFEQQRGHVVSGVECYMKQHNATKEEVLVEFNRRI
TSAWKDMNQECLHPLPVPMHLLERVLNLACFMNIFYKDEDCYTHSNTRMK
DFITSLLIESVPS
>NjTPS22
MISSSSVRSLYFPKTNIITSKVPSLLINNINVPSNNSSIRACISMSSLPV
SKSTSSSTAAPLIRDNGSLLKFITQTPQVEVDESKRIMELVETTRRTLRK
ASSDPTDKMKLIDSLQRLGLNHHFEEDINVVLQEFANEQKNTNEDLFTTS
LRFRLLRHNGYNVTPDIFNKFTEKNGKFKESLSEDTIGILSLYEASYLGA
KGEEILSEAIKFSESKLRESAGHVAPQIRRQILQSLELPRHLRMARLESR
RFIEEDYSKEIGCDLSLLELAKLDFNYVQSLHQMELAEISRWWKQLGLAD
KLPFARDRPLECFLWTVGLLPEPKHSECRIELAKTIAVLLVIDDIFDTYG
SFDQLVLFTNAIRRWDLDAMEELPEYMKICYMALYNTTNEICYKVLKENG
WSVLPYLKRTWIDMIEGFMVEAEWLNSGQVPNLEEYIENGVTTAGSYMAL
VHIFFLIGDGVTDDNVKLLLDPYPKLFSSAGRILRLWDDLGTAKEEQERG
DVSSSIQLYMKENNISSESEGRKQIIEIIHNLWKDLNGELIGSNAMPLPI
IKTSFNMARTSQVVYQHEDDSYFSSVDNYVQSLFFTPI
>NjTPS23
MSLTIFSSLKFVTSSTQLPSNGRIRQTQFIKCIPDDTRTCDQAIAMPRRL
ANYHPSIWEDDYVQSFGNNYTGESWSKRASELKEEVRGMFGKVEDSLMKV
ELIDTLQRLGISYQFEHEIKNTLENIYNDHYRSERWNNKEDYNLFAIALE
FRILRQHGYNVPQEIFNKFKDDHGNFKSSLCQDIKGMLYFYEASFLSIRG
ESILDDARDFTTKTLEEYLKNNKLVINNLDEILDLLVDHALELPLHWRML
RLEARWFIDVYERKQDVDTTLLEFAKIDFNMVQASHQEELKQMSRWWKST
YLGEKLRFARDRLMENYFWTTGVMFEPEYEYSRRMSTKINSIVTIIDDIY
DVYGSLQELQLFTNAVQRWDMKAMDELPDYMKICFLALYNSTNEMAYNTL
VQKGVYVISPLTKAWADLCKSYLLEARWYYSGYTPTLEEYMENAWISISA
PLTLVNAYLLVTNKLTTNSLECLKDYPNIIRWSSMILRFANDLGTSADEL
KRGDNPKSIQCYMHETGTVSEIEAREHIKHLISELWKKLNEEVYHHHHCR

RQNLDSPFDEVLAMNLARMAQCMYQHGDGHGIEDQETKDRVLALLINPIN
>NjTPS24
MISSSSVRSLCFPKTNIITSKVPSLLINNINVPSNNSSIRACISMSSLPV
SKSTSSSTAAPLIRDNGSLLTKFITQTPQVEVDESKRIMELVETTRRTLR
KASSDPTDKMKLIDSLQRLGLNHHFEEDINVVLQEFANEQKNTNEDLFTT
SLRFRLLRHNGYNVTPDIFNKFTEKNGKFKESLSEDTIGILSLYEASYLG
AKGEEILSEAIKFSESKLRESAGHVAPQIRRQILQSLELPRHLRMARLES
RRFIEEDYSKEIGCDLSLLELAKLDFNYVQSLHQMELAEISRWWKQLGLA
DKLPFARDRPLECFLWTVGLLPEPKHSECRIELAKTIAVLLVIDDIFDTY
GSFDQLVLFTNAIRRWDLDAMEELPEYMKICYMALYNTTNEICYKVLKEN
GWSVLPYLKRTWIDMIEGFMVEAEWLNSGQVPNLEEYIENGVTTAGSYMA
LVHIFFLIGDGVTDDNVKLLLDPYPKLFSSAGRILRLWDDLGTAKEEQER
GDVSSSIQLYMKENNISSESEGRKQIIEIIHNLWKDLNGELIGSNAMPLP
IIKTSFNMARTSQVVYQHEDDSYFSSVDNYVQSLFFTPI
>NjTPS25
MPIRKGLTRLEARHYIPIYQMDNSHDETLLKFAKLDFNKLQKLHQSELGD
MTRWWKDFNVAEKLPYARDRFVECYFWALGVYFEPQYSHARRMLTKVIAF
ISLIDDTYDSYGTFEELSLFTDAIQRWDVNAKNQLPEYMRHIYGELLDVY
NAMEEELSKEGISYRIDYAKQTMKQQVRTYFDEAIWYNNGYVPTMEEYLK
VALVSCGYIMLSTTSFVGMGVSVVPNQAFDWVTSNPLIVEASSVVNRLSD
DKVGHKIEQERGHVVSAVECYMKQHNRTEEETIAEFKKRVTTAWKDMNQE
CLHPIAVPMHLLERVLNFARFMNVFYEDEDCYTNSKSRMKDCITSLLVES
IPI
>NjTPS26
MSIIIATNSTEHPIFRPLANFPPSLWGNLFTSFSMDNQAREIYAKEHEGL
KEKVRVMLLDTTNYKISEKINFINTVERLGVSYHFEKEIEELLHQMFDAH
SKLLDDIQEFDLFTLGIYFRILRQHGYKISCDVFNKLKDSNGEFKDELKD
DVNGMLSLYEATHVRTHGENILDEALIYTKAQLESMAAASLSPFLAKQVK
HALMQALHKGIPRIEARNYISVYEEDPNKNDLLLRFSKIDFNLVQMIHKQ
ELCDTFRWWKDLEFESKLSFARNRVVEAYLWTLSAYYEPKYSSARIILVK
LMVIISVTDDTYDAYGTLDELQLFTDAVQRLDMSSINQLPDYMKTIYKAL
LDLFDEIEDRLSKHETDHSYRVAYAKYVYKEIVRCYDMEYKWFNKNYVPA
FEEYMQKALVTSGNRLLITFSFLGMDEVATIQAFEWVKSNAKMIVSSNKV
LRLIDDIMSHEEEDERGHVATGIECFVKEHGLTREEVIVEFHKRIDDAWK
DINEEFITPNNLPIEILTRVLNLTRIGDVVYKYDDGYTHPEKALKDHIIS
LFVDPVSI
>NjTPS27
MVSLRFRLLRQHGHNVSCGVFGKFKDIEGRFKECLMDDVRGLLSLYESTH
MRVHKEDILEEALEFTTTHLEQVVKSPLSGSVLASQVVHALNMPIRKGLT
RIEARHFIPIYQQDESHDETLLKFAKLDFNMLQKVHQREVADITMWWKDL
NVSEKLPYARDRAVECYFWILGVYFEPQYSRARRILTKVICMTSLIDDTY
DSYGTFEELILFTDAIQRWDVNAKNQLPEYMRHIFGELLDVYGAMEEELS
KEGISYRVDYAKQIMIQLVTAYNHEAIWYHDGYVPTLEEYLEVALVSCGY
IMAATTSFVGMGVKAVPKQAFDWVSSNPLMVQASSIINRLTDDRVGHELE

QQRGHVASGVECYMKQHNATEEEVLVEFNKRITSAWKDMNQECLHPFPVP
IHLLERVLNLARFMNIFYKDEDCYTHSNTRMKGIITSILIESIPS
>NjTPS28
MSCMRSISSPSQLLSKSYDNIIDSSSSSSRSFSWSFKSTNPAAAARTSMC
LSSRSSPSAVVVPPTSNNGSFLKYLQQSTVLVPQEIDDNSRTMELIEETR
KELVKVREPVEKMRLIEALQRLGISYHFENEINIILENLSAGGGRHSDED
LFTTSLRFRLLRHNGHHISNDVFEKFVDENGKFKESLKEDTMGMLSLYEA
SYMGANGEDILLQAMEFTKNHLKESLPLMESNLGKQVLQSLELPKNLRMA
RLEARRYIEEYSNESDHNLALLELAKLDYNQVQSLHQMELAEISRWWKHL
GLVDKLSFARDRPLECFLWTVGILPEPKDSGCRIELAKTIAILLVIDDIF
DTHGSYDELVLFTNAIRRWDLNAMEELPEYMKICYMALYNTTNEICYKVL
KENGWSVLPFLKTTWIDMIEGFMVEAKWLNNEEVPNLEEYIENGVTTAGS
YMALVHIFFLIGEGVNEDNVKLLLNPYPKLFSSAGRILRLWDDLGTSKEE
QERGDVASSIQLFMKENNITCEEEARNQIIQIVQNLWKELNGELMAPNAL
PLPIIKACLNMARASQVVYQHDGDSYFSNVDNYVQSCFIHQFVCNAFLVI
IFFYIYTFYIYLFFHINKFMHID
>NjTPS29
MSCMRSISSPSPSQLLSKSYDNIIDSSSSSIRSFSWSFKSKNPAAAAACT
SMCLSSRSSPSAVVVPPTSNNGSFLKYLQQSTVLVPQEIDDNSRTMELIE
ETRKELVKVREPVEKMRLIEALQRLGISYHFENEINIILENLSAGGGRHS
DEDLFTTSLRFRLLRHNGHHISNDVFEKFVDENGKFKESLKEDTMGMLSL
YEASYMGANGEDILLQAMEFTKNHLKESLPLMESNLGKQVLQSLELPKNL
RMARLEARRYIEEYSNESDHNLALLELAKLDYNQVQSLHQMELAEISRWW
KHLGLVDKLSFARDRPLECFLWTVGILPEPKDSGCRIELAKTIAILLVID
DIFDTHGSYDELVLFTNAIRRWDLNAMEELPEYMRICYMALYNTTNEICY
KVLKENGWSVLPFLKTTWIDMIEGFMVEAKWLNNEEVPNLEEYIENGVTT
AGSYMALVHIFFLIGEGVNEDNVKLLLNPYPKLFSSAGRILRLWDDLGTS
KEEQERGDVASSIQLFMKENNITCEEEARNQIIQIVQNLWKELNGELMAP
NALPLPIIKACLNMARASQVVYQHDGDSYFSNVDNYVQSLFYTPIRM
>NjTPS30
MLLDTTNYKISEKINFINTVERLGVSYHFEKEIEELLHQMFDAHSKLLDD
IQEFDLFTLGIYFRILRQHGYKISCDVFNKLKDSNGEFKDELKDDVNGML
SLYEATHVRTHGENILDEALIYTKAQLESMAAASLSPFLAKQVKHALMQA
LHKGIPRIEARNYISVYEEDPNKNDLLLRFSKIDFNLVQMIHKQELCDTF
RWWKDLEFESKLSFARNRVVEAYLWTLSAYYEPKYSSARIILVKLMVIIS
VTDDTYDAYGTLDELQLFTDAVQRLDMSSINQLPDYMKTIYKALLDLFDE
IEDRLSKHETDHSYRVAYAKYVYKEIVRCYDMEYKWFNKNYVPAFEEYMQ
KALVTSGNRLLITFSFLGMDEVATIQAFEWVKSNAKMIVSSNKVLRLIDD
IMSHEEEDERGHVATGIECFVKEHGLTREEVIVEFHKRIDDAWKDINEEF
ITPNNLPIEILTRVLNLTRIGDVVYKYDDGYTHPEKALKDHIISLFVDPV
SI
>NjTPS31
MSCMRSISSPSPSQLLSKSYDNIIDSSSSSIRSFSWSFKSKNPAAAAACT
SMCLSSRSSPSAVVVPPTSNNGSFLKYLQQSTVLVPQEIDDNSRTMELIE

ETRKELVKVREPVEKMRLIEALQRLGISYHFENEINIILENLSGGGGRHS
DEDLFTTSLRFRLLRHNGHHISNDVFEKFVDENGKFKESLKEDTMGMLSL
YEASYMGANGEDILLQAMEFTKNHLKESLPLMESNLGKQVLQSLELPKNL
RMARLEARRYIEEYSNESDHNLALLELAKLDYNQVQSLHQMELAEISRWW
KHLGLVDKLSFARDRPLECFLWTVGILPEPKDSGCRIELAKTIAILLVID
DIFDTHGSYDELVLFTNAIRRWDLNAMEELPEYMKICYMALYNTTNEICY
KVLKENGWSVLPFLKTTWIDMIEGFMVEAKWLNNEEVPNLEEYIENGVTT
AGSYMALVHIFFLIGEGVNEDNVKLLLNPYPKLFSSAGRILRLWDDLGTS
KEEQERGDVASSIQLFMKENNITCEEEARNQIIQIVQNLWKELNGELMAP
NALPLPIIKACLNMARASQVVYQHDGDSYFSNVDNYVQSLFYTPIRM

>NjTPS32

MCTRESKMWILLCSNSRKNFNMVQASHQEELKQMSRWWKSTYLGEKLRFA
RDRLMENYFWTTGVMFEPEYEYSRRMSTKINSIVTIIDDIYDVYGSLQEL
QLFTNAVQRWDMKAMDELPDYMKICFLALYNSTNEMAYNTLVQKGVYVIS
PLTKAWADLCKSYLLEARWYYSGYTPTLEEYMENAWISISAPLTLVNAYL
LVTNKLTTNSLECLKDYPNIIRWSSMILRFANDLGTSTDELKRGDNPKSI
QCYMHETGTVSEIEAREHIKHLISELWKKLNEEVYHHHHCRRQNLDSPFD
EVLAMNLARMAQCMYQHGDGHGIEDQETKDRVLALLINPIN

>NjTPS33

MDSYLNVSSGPPPKKNIQEPVRPIANFHPSVWGNYFLKYASNPEQSDGGA
EEQHEQLKEALRKKLVVNDNDELAGEQLKLIDAIQRLGVAYHFETEIDVV
LNNQLQLLNNQDDDLHMVSLRFRLLRQQGHNVSCGVFGKFMDIEGRFKEY
LVDDVRGLLSLYESTHMRVHKEDILEEALEFTTAHLEQVVKSPFSCSVLA
SQVVHALKLPIRKVLTRIEARHFIPIYQQDKSHDETLLKFAKLDFNMLQK
IHQREVADLTLWWKDLNVSEKLPYARDRAVECYFWILGAYFEPQYSRARR
ILTKVLCMTSLIDDTYDSYGTFEELILFTDAIQRWDVNAKNQLPEYMRHI
FGKLLDVYGEMEEELSKEGISYRVDYAKQIMIQLVTAYNQEAIWYHDGYV
PTLEEYLENAVVSSSYLMIATTSFVGMGVTTVSKQDFDWVSSKPLMVRAS
SIMNRLANDKLGHELEQQRGHVASGVECYMKQHNATKEEMLVEFNRRITS
AWKDMNQECLHPLPVPIHLLERVLNLARCKNIFYIDEDGYTQSSTRTKDF
ITSLLIESVPN

>NjTPS34

MALAYSHLSTFHLSTIPSNNKLLPPPQLTSIRTSKGSKSSKCMAIPTPQD
HIIVRRVADYHPPIWDYDYVQSLTSKYLGDSYQKRADKLKGEVRNMLNKV
EDPLSKLELVDTLQRLGIYYHFEDEIKRILQSIHNDDNYSSDEENKEDLY
TTSLKFRLLRQHGYDIPQEVFNTFKDESETFKVHVPEDIKGILSFYESTF
LSTRGESILDEAREFTTQNMKEYLKKIIDINKSDDVMATQVSHALEMPLH
WRMLRLETRWFIDVYEKTDNKNEILLEFAKLDYNMVQAIHQKDLKYTSRW
WKSTKLGEKLSFARDRLVENFFWNVGFTFEPQFEYRRRMETKLLSFITII
DDIYDIYGTLDELQLFTNAVQRWDINAMEQLPDYMKTCFLSLYNTINEFA
YDALKEQNVNVISILKNSWADLCNSYFIEAKWYHTGYKPSLDEYLENARI
SVSAPLILTHAYYFLTYPHPNVALECFEKYSSLICSTSIIVRLADDLATS
QSEMKRGDTPKSIQCYMYETGASEEEAREYIRYLISETWKKMNEDRVVNK
DDSLFSPIFVEMAMNIARMGQCIFDHGDGFGIANRETMDRVRSLFIEPIS

L
>NjTPS35
MDRSIQASSAPLPLSVLEPTRPIANFHPTIWGNYFLKFASDPGTNDDSDI
NQQIAQMKEDVRKMIVNSGDRREQQLKLIDEIQRLGVSYHFKSEIDVVLN
DHLLTLNDNGDDLYMESLRFRLLRQHGHNVSCDVFEKFKDGEGRFKEYLT
DDVRGLLSLYEATHMRVHKEEILDEALEFTTSHLEQVVKYSLSDHVLASK
VVHALKMPIRKGLTRLEARHYIPIYQMDNSHDETLLKFAKLDFNKLQKLH
QSELGDMTRWWKDFNVAEKLPYARDRFVECYFWALGVYFEPQYSHARRML
TKVIAFISLIDDTYDSYGTFEELSLFTDAIQRWDVNAKNQLPEYMRHIYG
ELLDVYNAMEEELSKEGISYRIDYAKQTMKQQVGTYFDEAIWYNNGYVPT
MEEYLKVALVSCGYIMLSTTSFVGMGVSVVPNQAFDWVTSNPLIVEASSV
INRLSDDKVGHKIEQERGHVVSAVECYMKQHNRTEEETIAEFKKRVTTAW
KDMNQECLHPIAVPMHLLERVLNFARFMNVFYEDEDCYTNSKSRMKDCIT
SLLVESIPI
>NjTPS36
MNTCIQALSPPPPTKAIQLRPIANFHPSIWGNYFLKYASDHHTQSDDGTD
EQHGQLKEDVRKKLVVNDERAGEQLMLIDAIQRLGVAYHFQTEIDVVLNN
QLLKFNEDDDLYMVSLRFRLLRQQGHHVSSGVFEKFKDVEGRFKEILIND
VRGLLSLYESTHMRVHKEEILEEALQFTTTHLEHVVKASLTDTILLSQVV
HALNMPIRKGLTRIEARNYIPIYQQDKSHDETLLKFAKLDFNMLQKVHQR
ELGDITRWWKDLNVAEKLPYARDRLVEGYFWILGVYFEPRYSRARKILTK
VFSLTSLIDDTYDSYGTFEELILFTDAVQRWDVNAKNQLPEYMRHVYGEL
LDVYSAMEEELLKEGISYRVEYAKQAMKQLVRAYNDEAIWYHKDYVPTLE
EYLKVALVSCGYIILATTSFVGMGVSDVTKQDFDWVSSNPLIVQASSVIC
RLTDDDVGHEFEQERGHVASAVECYMKQHNATKEEALVEFKKRITNAWKD
MNRECLHPLPVPMHLLERVPNFARFMYLFYKGEDCYTHSETRMKSFITSL
LVESAPN
>NjTPS37
MSLTIFSSLKFVTSSTQLPSNGRIRQTQFIKCIPDDTRTCDQAIAMPRRL
ANYHPSIWEDDYVQSFGNNYTGESWSKRASELKEEVRGRFGKVEDSLMKV
ELIDTLQRLGISYQFEHEIKNTLENIYNDHYRSERWNNKEDYSLFAIALE
FRILRQHGYNVTQEIFNKFKDDHGNFKSSLCQDIKGMLYFYEASFLSIRG
ESILDDARDFTTKTLEEYLKNNKLVINNLDEILDLLVDHALELPLHWRML
RLEARWFIDVYERKQDVDTTLLEFAKIDFNMVQASHQEELKQMSRWWKST
YLGEKLRFARDRLMENYFWTTGVMFEPEYEYSRRMSTKINSIVTIIDDIY
DVYGSLQELQLFTNAVQRWDMKAMDELPDYMKICFLALYNSTNEMAYNTL
VQKGVYVISPLTKAWADLCKSYLLEARWYYSGYTPTLEEYMENAWISISA
PLTLVNAYLLVTNKLTTNSLECLKDYPNIIRWSSMILRFANDLGTSADEL
KRGDNPKSIQCYMHETGIVSEIEAREHIKHLISELWKKLNEEVYHHHHCR
RQNLDSPFDEVLAMNLARMAQCMYQHGDGHGIEDQETKDRVLALLINPIN
>NjTPS38
MDDVRGLLSLYESTHMRVHKEDILEEALEFTTTHLEQVVKSPLSGSVLAS
QVVHALNMPIRKGLTRIEARHFIPIYQQDESHDETLLKFAKLDFNMLQKV
HQREVADITMWWKDLNVSEKLPYARDRAVECYFWILGVYFEPQYSRARRI

LTKVICMTSLIDDTYDSYGTFEELILFTDAIQRWDVNAKNQLPEYMRHIF
GELLDVYGAMEEELSKEGISYRVDYAKQIMIQLVTAYNHEAIWYHDGYVP
TLEEYLEVALVSCGYIMAATTSFVGMGVKAVPKQAFDWVSSNPLMVQASS
IINRLTDDRVGHELEQQRGHVASGVECYMKQHNATEEEVLVEFNKRITSA
WKDMNQECLHPFPVPIHLLERVLNLARFMNIFYKDEDCYTHSNTRMKGII
TSILIESIPS
>NjTPS39
MVSLRFRLLRQHGHNVSCGVFGKFKDIEGRFKECLMDDVRGLLSLYESTH
MRLHKEDILEEALEFTTTHLEQVVKSPLSGSVLASQVVHALNMPIRKGLT
RIEARHFIPIYQQDESHDETLLKFAKLDFNMLQKVHQREVADITMWWKDL
NVSEKLPYARDRAVECYFWILGVYFEPQYSRARRILTKVICMTSLIDDTY
DSYGTFEELILFTDAIQRWDVNAKNQLPEYMRHIFGELLDIYGAMEEELS
KEGISYRVDYAKQIMIQLVTAYNHEAIWYHDGYVPTLEEYLEVALVSCGY
IMAATTSFVGMGVTAVPKQAFDWVSSNPLMVQASSIINRLTDDRVGHELE
QQRGHVASGVECYMKQHNATEEEVLVEFNKRITSAWKDMNQECLHPLPVP
IHLLERVLNLARFMNIFYKDEDCYTHSNTRMKGIITSILIESIPS
>NjTPS40
MAYSLLSTFHLSTIPSNDKLLPPPQLTSIRTSKGSKSSKSSKCMAIATPQ
DHIIVRRVADYHPPIWDYDYVQSLTSKYLGDSYQKRADKLKEEVRNMLNK
VEDPLSKLELVDTVQRLGIYYHFEDEIKRILQSIHNDDNYSSDEENKEDL
YTTSLKFRLLRQHGYDIPQEVFSTFKDESGTFKVHVPEDIKGILSFYEST
FLSTRGESILDEAREFTTQNMKEYLKKIIDINKSDDIMATQVSHALEMPL
HWRMLRLETRWFIDVYEKTDNKNEILLEFAKLDYNMVQVIHQEDLKYTSR
WWKSTKLGEKLSFARDRLEENFFWNVGFTFEPQFEYCRRMETKLLSLITI
IDDIYDIYGTLDELQLFTNAVERWDINAMEQLPDYMKTCFLSLYNTTNET
AYDALKEQNVNIISFLKNSWADLCKSYFIEAKWYHTGYKPNLDEYLENAR
ISVTAPLILTHAYYFLTNPHPNVALECFEKYSSLIRSTSIILRLADDLAT
SESEMKRGDTPTSIQCYMYETGASEEEAREYIRYLISETWKKMNEDRVVN
KDDSLFSPIFVEMALNIARMGQCIYDHGDGFGIANRETMDRVTSLFVEPI
SL
>NjTPS41
MVSLRFRLLRQHGHNVSCGVFGKFKDIEGRFKECLMDDVRGLLSLYESTH
MRLHKEDILEEALEFTTTHLEQVVKSPLSGSVLVSQVVHALNMPIRKGLT
RIEARHFIPIYQQDESHDETLLKFAKLDFNMLQKVHQREVADITMWWKDL
NVSEKLPYARDRAVECYFWILGVYFEPQYSRARRILTKVICMTSLIDDTY
DSYGTFEELILFTDAIQRWDVNAKNQLPEYMRHIFGELLDIYGAMEEELS
KEGISYRVDYAKQIMIQLVTAYNHEAIWYHDGYVPTLEEYLEVALVSCGY
IMAATTSFVGMGVTAVPKQAFDWVSSNPLMVQASSIINRLTDDRVGHELE
QQRGHVASGVECYMKQHNATEEEVLVEFNKRITSAWKDMNQECLHPLPVP
IHLLERVLNLARFMNIFYKDEDCYTHSNTRMKGIITSILIESIPS
>NjTPS42
MNTCIQALSPPPPTKAIQLRPIANFHPSIWGNYFLKYASDHHTQSDDSTD
EQHGQLKEDVRKKLVVNDERAGEQLMLIDAIQRLGVAYHFQTEIDVVLNN
QLLKFNEDDDLYMVSLRFRLLRQQGHHVSSDVFEKFKDVEGRFKEILIND

VRGLLSLYESTHMRVHKEEILEEALQFTTTHLEHVVKASLTDTILLSQVV
HALNMPIRKGLTRIEARNYIPVYQQDKSHDETLLKFAKLDFNMLQKVHQR
ELGDITRWWKDLNVAEKLPYARDRLVEGYFWILGVYFEPRYSRARKILTK
VFSLTSLIDDTYDSYGTFEELILFTDAVQRWDVNAKNQLPEYMRHVYGEL
LDVYSAMEEELLKEGISYRVEYAKQLMKQLVRAYNDEAIWYHKDYVPTLE
EYLKVALVSCGYIMVATTSFVGMGVSDVTKQDFDWVSSNPLIVQASSEIS
RLTDDEVGHEFEQERGHVVSAVECYMKQHNATKEEALVEFKKRITNAWKD
MNQECLHPLPVPMHLLERVQNIVRFMYLFYKGEDCYTHSETRMKSFITSL
LVESAPN
>NjTPS43
MDSYLNASSAPPPKKNMQEPIRPIANYHPSVWENHFLKYASNPKQSDGGA
EEQHEQLKEALRKKLVLNVANERAGEQLKLIDAIQQLGVAYQFETEIDVV
LNNQLQLLNNQDDDLHMVSLRFRLLRQHGHNVSCGVFGKFKDIEGRFKEC
LMDDVRGLLSLYESTHMRVHKEDILEEALEFTTTHLEQVVKSPLSGSVLA
SQVVHALNMPIRKGLTRIEARHFIPIYQQDESQDETLLKFAKLDFNMLQK
VHQREVADITMWWKDLNVSEKLPYARDRAVECYFWILGVYFEPQYSRARR
ILTKVICMTSLIDDTYDSYGTFEELILFTDAIQRWDVNAKNQLPEYMRHI
FGELLDVYGAMEEELSKEGISYRVDYAKQIMIQLVTAYNHEAIWYHDGYV
PTLEEYLEVALVSCGYIMAATTSFVGMGVTAVPKQAFDWVSTNPLMVQAS
SIINRLTDDRVGHEVCRFVRIESRNVGRDSHV
>NjTPS44
MNTCIQALSPPPPRKAIQLRPIANFHPSIWGNYFLKYASDHHTQSDDGTD
EQHGQLKEDVRKKLVVNDERAGEQLMLIDAIQRLGVAYHFQTEIDVVLNN
QLLKFNEDDDLYMVSLRFRLLRQQGHHVSSGVFEKFKDVEGRFKEILIND
VRGLLSLYESTHMRVHKEEILEEALQFTTTHLEHVVKASLTDTILLSQVV
HALNMPIRKGLTRIEARNYIPIYQQDKSHDETLLKFAKLDFNMLQKVHQR
ELGDITRWWKDLNVAEKLPYARDRLVEGYFWILGVYFEPRYSRARKILTK
VFSLTSLIDDTYDSYGTFEELILFTDAVQRWDVNAKNQLPEYMRHVYGEL
LDVYSAMEEELLKEGISYRVEYAKQAMKQLVRAYNDEAIWYHKDYVPTLE
EYLKVALVSCGYIILATTSFVGMGVSDVTKQDFDWVSSNPLIVQASSVII
RLTDDEVGHEERGHVASAVECYMKQHNATKEEVLVEFKKRITNAWKDMNR
ECLHPHPVPMHLLERVQNFVRFMYLFYKGEDCYTHSETRMKSFITLLLVE
SAPN
>NjTPS45
MDSYLNASSAPPPKKNMQEPIRPIANYHPSVWENHFLKYASNPKQSDGGA
EEQHEQLKEALRKKLVLNVANERAGEQLKLIDAIQQLGVAYQFETEIDVV
LNNQLQLLNNQDDDLHMVSLRFRLLRQHGHNVSCGVFGKFKDIEGRFKEC
LMDDVRGLLSLYESTHMRVHKEDILEEALEFTTTHLEQVVKSPSSGSVLA
SQVVHALNMPIRKGLTRIEARHFIPIYQQDESQDETLLKFAKLDFNMLQK
VHQREVADITMWWKDLNVSEKLPYARDRAVECYFWILGVYFEPQYSRARR
ILTKVICMTSLIDDTYDSYGTFEELILFTDAIQRWDVNAKNQLPEYMRHI
FGELLDVYGAMEEELSKEGISYRVDYAKQIMIQLVTAYNHEAIWYHDGYV
PTLEEYLEVALVSCGYIMAATTSFVGMGVTAVPKQAFDWVSTNPLMVQAS
SIINRLTDDRVGHEVCRFVRIESRNVGRDSHV

>NjTPS46
MLSLYEATHVRTHGENILDEALIYTKAQLESMAAASLSPFLAKQVKHALM
QALHKGIPRIEARNYISVYEEDPNKNDLLLRFSKIDFNLVQMIHKQELCD
TFRWWKDLEFESKLSFARNRVVEAYLWTLSAYYEPKYSSARIILVKLMVI
ISVTDDTYDAYGTLDELQLFTDAVQRLDMSSINQLPDYMKTIYKALLDLF
DEIEDRLSKHETDHSYRVAYAKYVYKEIVRCYDMEYKWFNKNYVPAFEEY
MQKALVTSGNRLLITFSFLGMDEVATIQAFEWVKSNAKMIVSSNKVLRLI
DDIMSHEEEDERGHVATGIECFVKEHGLTREEVIVEFHKRIDDAWKDINE
EFITPNNLPIEILTRVLNLTRIGDVVYKYDDGYTHPEKALKDHIISLFVD
PVSI
>NjTPS47
MRLHKEDILEEALEFTTTHLEQVVKSPLSGSVLASQVVHALNMPIRKGLT
RIEARHFIPIYQQDESHDETLLKFAKLDFNMLQKVHQREVADITMWWKDL
NVSEKLPYARDRAVECYFWILGVYFEPQYSRARRILTKVICMTSLIDDTY
DSYGTFEELILFTDAIQRWDVNAKNQLPEYMRHIFGELLDIYGAMEEELS
KEGISYRVDYAKQIMIQLVTAYNHEAIWYHDGYVPTLEEYLEVALVSCGY
IMAATTSFVGMGVTAVPKQAFDWVSSNPLMVQASSIINRLTDDRVGHELE
QQRGHVASGVECYMKQHNATEEEVLVEFNKRITSAWKDMNQECLHPLPVP
IHLLERVLNLARFMNIFYKDEDCYTHSNTRMKGIITSILIESIPS
>NjTPS48
MDSYLNASSAPPPKKNMKEPVRPIANYHPSVWGNQFLKYASNPKQSDGGA
EEQHEQLKEALRKKLVVNVANERAGEQLTLIDAIQRLGVAYQFETEIDVV
LNNQLQLLNNQDDDLHMVSLRFRLLRQHGHNVSCGVFGKFKDIEGRFKEC
LMDDVRGLLSLYESTHMRLHKEDILEEALEFTTTHLEQVVKSPLSGSVLA
SQVVHALNMPIRKGLTRIEARHFIPIYQQDESHDETLLKFAKLDFNMLQK
VHQREVADITMWWKDLNVSEKLPYARDRAVECYFWILGVYFEPQYSRARR
ILTKVICMTSLIDDTYDSYGTFEELILFTDAIQRWDVNAKNQLPEYMRHI
FGELLDIYGAMEEELSKEGISYRVDYAKQIMIQLVTAYNHEAIWYHDGYV
PTLEEYLEVALVSCGYIMAATTSFVGMGVTAVPKQAFDWVSSNPLMVQAS
SIINRLTDDRVGHEVCI
>NjTPS49
MLYFYEASFLSIRGESILDDARDFTTKTLEEYLKNNKLVINNLDEILDLL
VDHALELPLHWRMLRLEARWFIDVYERKQDVDTTLLEFAKIDFNMVQASH
QEELKQMSRWWKSTYLGEKLRFARDRLMENYFWTTGVMFEPEYEYSRRMS
TKINSIVTIIDDIYDVYGSLQELQLFTNAVQRWDMKAMDELPDYMKICFL
ALYNSTNEMAYNTLVQKGVYVISPLTKAWADLCKSYLLEARWYYSGYTPT
LEEYMENAWISISAPLTLVNAYLLVTNKLTTNSLECLKDYPNIIRWSSMI
LRFANDLGTSADELKRGDNPKSIQCYMHETGIVSEIEAREHIKHLISELW
KKLNEEVYHHHCRRQNLDSPFDEVLAMNLARMAQCMYQHGDGHGIEDQET
KDRVLALLINPIN
>NjTPS50
MGMLSLYEASYMGANGEDILLQAMEFTKNHLKESLPLMESNLGKQVLQSL
ELPKNLRMARLEARRYIEEYSNESDHNLALLELAKLDYNQVQSLHQMELA
EISRWWKHLGLVDKLSFARDRPLECFLWTVGILPEPKDSGCRIELAKTIA

ILLVIDDIFDTHGSYDELVLFTNAIRRWDLNAMEELPEYMKICYMALYNT
TNEICYKVLKENGWSVLPFLKTTWIDMIEGFMVEAKWLNNEEVPNLEEYI
ENGVTTAGSYMALVHIFFLIGVGVNEDNVKLLLNPYPKLFSSAGRILRLW
DDLGTSKEEQERGDVASSIQLFMKENNITCEEEARNQIIQIVQNLWKELN
GELMAPNALPLPIIKACLNMARASQVVYQHDGDSYFSNVDNYVQSLFYTP
IRM
>NjTPS51
MNTCIQALSPPPPRKAIQLRPIANFHPSIWGNYFLKYASDHHTQSDDGTD
EQHGQLKEDVRKKLVVNDERAGEQLMLIDAIQRLGVAYHFQTEIDVVLNN
QLLKFNEDDDLYMVSLRFRLLRQQGHHVSSGVFEKFKDVEGRFKEILIND
VRGLLSLYESTHMRVHKEEILEEALQFTTTHLEHVVKASLTDTILLSQVV
HALNMPIRKGLTRIEARNYIPIYQQDKSHDETLLKFAKLDFNMLQKVHQR
ELGDITRWWKDLNVAEKLPYARDRLVEGYFWILGVYFEPRYSRARKILTK
VFSLTSLIDDTYDSYGTFEELILFTDAVQRWDVNAKNQLPEYMRHVYGEL
LDVYSAMEEELLKEGISYRVEYAKQAMKQLVRAYNDEAIWYHKDYVPTLE
EYLKVALVSCGYIILATTSFVGMGVSDVTKQDFDWVSSNPLIVQASSVII
RLTDDEVGHEFEQERGHVASAVECYMKQHNATKEEVLVEFKKKKKKKKKK
>NjTPS52
MKGTNSDVRAMLQLYEASHLALVDEHVLNEEKCLSEEILKNSINSGLIND
ESLIKQIKIALKLPLQWSVEWYNVKIHIQILGEGDKTSINKTLLKLAKLN
FNMVQATHQKELRQISRWWRDLGVAESLNFTRNRIVESYLWAMGVAFEPS
NGSLRKWLAKVIKLIIIIDDIYDIYASPEELVIFTGAVYRWDIDEIEELP
ECMKICFQTLHDTTNEIASEIQKEKGWSSLVVPYLRKAWGDFCKALHVEA
EWDNKGYTPSLEEYLENGWISSSGPLLSIHIFFSVTQQVQQQEQVENLLQ
RSQDLVHCTSLIIRLCNDEGTSAAELERGDAPSSILCLMREENVSEEVAR
KKIRSRIRNIWMKINKECIDQSPLMLKPFFKCAVNTARVAHFIYQNGDGF
GVQDRETRDQVLSLLVEPLVLD
>NjTPS53
MPIRKGLTRIEARHFIPIYQQDESHDETLLKFAKLDFNMLQKVHQREVAD
ITMWWKDLNVSEKLPYARDRAVECYFWILGVYFEPQYSRARRILTKVICM
TSLIDDTYDSYGTFEELILFTDAIQRWDVNAKNQLPEYMRHIFGELLDIY
GAMEEELSKEGISYRVDYAKQIMIQLVTAYNHEAIWYHDGYVPTLEEYLE
VALVSCGYIMAATTSFVGMGVTAVPKQAFDWVSSNPLMVQASSIINRLTD
DRVGHELEQQRGHVASGVECYMKQHNATEEEVLVEFNKRITSAWKDMNQE
CLHPLPVPIHLLERVLNLARFMNIFYKDEDCYTHSNTRMKGIITSILIES
IPS
>NjTPS54
MMLASSHYGHKTTTASTSTVPYKKQSIEGTKERIRKMFKSNKVELSASPY
DTAWVAMVPSPNSSNAPCFPECLDWLLKNQLGNGSWGLPSHSHLLLKDTL
SSTLASVLALKRWNVGQSHINKGLHFMELNFQSAIDKNQHSPIGFDIIFP
GMLNYAKDLDLKLPLEPTLLNAMLHTRDLELNRCYESKAEAYLAYVSEGM
GKLQQDWEIVMKKYQRKNGSIFNSPATTAAVLTHHLPDAASLNYIRLLLD
KFGNAVPTVYPLDIYVRLCMIDNLERLGIDWHFRDEIQTVLDETYRCWLQ
GDEQIFTDISTCAIAFRLLRMNGYDVSSDALTQIAEEGNYLNSPGDRNLK

GISDELELYKASQIIISPDESSALRKQNLQSSNFLKQMLSDDSYCSDKLS
RSISQEVDDALNFPFCASLERMANRRYIEQYNVDTSTIRVLKTSYFSSNI
GNKDFLKLAVEDFNKCQSRHREDAAYLARWVIENRLDKLKFVRQKSFGYM
SFSAAATSFTPKLSDARMSWAKNALLTTVVDDFFDIGGSMDELLNLIYLV
DKWDNVDIESDCCSEHVGIIFSALQRGINEIAELAFVYQERNVTSHIVEI
WLDLLKSMLREAEWSRDRYVPSMEEYMENGYVSFALGPILLPALYLVGPK
LSDESARSFQLKKLFRLMSNCGRLLNDIQGFKRESKEGKLNSVSLRMMMM
MNEGDTDEGIIVNELKMLVESYKEELLRIVIEEKESVLPRECKELFWKMT
KVVHQFYLKDDGFTSQHMMKAVNDVIYQPIILEEHQLK
>NjTPS55
MDRSIQASSAPLPLSVLEPTRPIANFHPTIWGNYFLKFASDPGTNDDSDI
NQQIAQMKEDVRKMMVNSGDRREQQLKLIDEIQRLGVSYHFKSEIDVVLN
DHLLTLNDNGGDLYMESLRFRLLRQHGHNVSCDVFEKFKDGEGRFKEYLT
DDVRGLLSLYEATHMRVHKEEILDEALEFTTSHLEQVVKYSLSDHVLASK
VVHALKMPIRKGLTRLEARHYIPIYQMDNSHDETLLKFAKLDFNKLQKLH
QSELGDMTRWWKDFNVAEKLPYARDRFVECYFWALGVYFEPQYSHARRML
TKVIAFISLIDDTYDSYGTFEELSLFTDAIQRWDVNAKNQLPEYMRHIYG
ELLDVYNAMEEELSKEGISYRIDYAKQTMKQQVGTYFDEAIWYNNGYVPT
MEEYLKVALVSCGYIMLSTTSFVGMGVSVVPNQAFDWVTSNPLIVEASSV
VNRLSDDKVGHKIEQERGHVVSAVECYMKQHNRTEEETIAEFKKRVTTAW
KDMNQECLHPIAVPMHLLERVLNFARFMNVFYEDEDCYTNSKSRMKDCIT
SLLVESIPI
>NjTPS56
MSLTIFSSLKFVTSSTQLPSNGRIRQTQFIKCIPDDTRTCDQAIAMPRRL
ANYHPSIWEDDYVQSFGNNYTGESWSKRASELKEEVRGRFGKVEDSLMKV
ELIDTLQRLGISYQFEHEIKNTLENIYNDHYRSERWNNKEDYNLFAIALE
FRILRQHGYNVPQEIFNKFKDDHGNFKSSLCQDIKGMLYFYEASFLSIRG
ESILDDARDFTTKTLEEYLKNNKLVINNLDEILDLLVDHALELPLHWRML
RLEARWFIDVYERKQDVDTTLLEFAKIDFNMVQASHQEELKQMSRWWKST
YLGEKLRFARDRLMENYFWTTGVMFEPEYEYSRRMSTKINSIVTIIDDIY
DVYGSLQELQLFTNAVQRWDMKAMDELPDYMKICFLALYNSTNEMAYNTL
VQKGVYVISPLTKAWADLCKSYLLEARWYYSGYTPTLEEYMENAWISISA
PLTLVNAYLLVTNKLTTNSLECLKDYPNIIRWSSMILRFANDLGTSAVSI
FDPSRISYIIIFYDKINYIFVFLV
>NjTPS57
MYLSQSFTLTKTYYPYRITASFHSHSTAKTATNATTTTTVPSIEDGSKER
IRKLFKKVEVSVSSYDTAWVAMIPGRDGYGPIFPECLDWLLNNQLHDGSW
GLNPPFLLKDTLSSTLASILALKRWGVGQHHINKGMKFMDLNFHSAIDKN
HHSPIGFDIIFPGMLDYAKDLDLKLPLKPALSDAIFLNRESELKRCYESE
AHERDAYLAYVSEGIGKSQDWKMVMKHQSKNGSLFNSPATTAAALTHHLP
SDSGLLNYIRLLLHKFGNAVPTSYPVDIFVRLCMIDNLERLGIDWHFRDE
IQSVLDETYRCWIKGDEELFMDVSTCAIAFRLLRMNGYDVSSDLLIRTAE
ENDCFSSSGGHLKGISDTLELYRASQIIISPDESNLRKHNSKSSHILKYA
LSNDSFCSDKLATYIRQEVDDGLKFPFYASLERMENRRNIEQYSADCSVI

RVLKTSYCSPNIGNKDFLKLAIEDFNFIQSIHREEIKHVESWVIEGKLDK
LKFARQREAYCLFSAAATFFTPELSDARIIWAQNAVLTIVVDDFFDNAGS
PHEFLNLIRLAEKWNVDIESDCCSKEVGILFSAIHNANTENADKAFICQG
RSVTNHIVQIWLDYLKALWIEAEWTRNKYVPSVDEYMENAYITVALGPII
LPALYVVGEELSEEAVRSIEFNKMFQLVSSCGRIINDTQTLERETEQGKL
NIVSLLMIHGGITKEEAIDEARNLIKRQREELLSLVVKKGSVVPRACKEL
FWKMAKVLHLFYDHDDGLTSHHLMKAVNDIIYEPIP
>NjTPS58
MNITQTTSSVMLVPSSNRAHRSLVVTCCMVQCDPSGLRTASSYAGQVDPT
AMTPDMTKERIRNLFNNVELSVSSYDTAWVAMVPSPNSPKSPCFPNCLNW
LLDNQLDDGSWGLLHHNPSLTKDNLSSTLACILALKKWNVGKYEINKGLH
FIESNFASVNDKNQVSPIGFEIIFPGMLEYAKDLSIKLPLNQTYLNSMLH
ERELELRRCHSNGRDTYLAYISEGLGNYNDWNLVMKYQMKNGSLFNSPSA
TASVLIHHQNAGCLNYLTSLLDKFGNAVPTVYPLDLYVRLCMVDTLERLG
IKHHFKLEIQNVLDETYRCWMQGDVLIFMDVDTCALAFRVLRSNGYEVSS
DPLAKLIKEGNHMNSPEKPFKDVYTALEVYKASQITYQDELAFEEQNLTS
YLPLSKEVDDALKFPFNGSLERMSTRRNIEHYNINPTRILKTSYSSSNIR
NRDYLNLAVQDYNTCQSIYREELKDLERWVVENRLDRLKFARQKTAYCYF
SAASFLSAPELSDARLSWAKSSILTTVIDDFFDVGGSMDELVNFVHILEK
WNVDVESNCCSEEVGIIFLALKNAICWIGDKAFKWQERDITSHVIEIWLD
LVKSMLQEAIWARDGSVPTINEYMENGYVSFALGPIVLPTLYFLGVKLSK
EIELMSTRGRLMNDIHSFKVSFHSVTVCCLTSCVNAERNDGWEIECCGIV
HE
>NjTPS59
MALVRNNSSNGREPVLSPRSLTSPRGLTSPRPLAARPTPEPVRPLANFPP
SIWADRFITFSLDNSELEAYANALEEPKEAVRSLITDTTIDATTKLKLIY
SVHRLGLSYLYPEEIDSELDKLFKKIDLDYYEQVDLYTISVQFQVFRHHG
YKLSSDIFKKFKDTTTGIFTDEVSKDVKGMLSLYESAHLRLHGEDILDEA
LAFTESQLKKIVSTLEGDLARQVNQVLKRPFHTGMPMVEARLYFNTHEED
FSCHEAVVKLAKINFNYLQLQQKEELRMVSQWWKDMEFQTSVPYIRDRVP
EIYLWILGLYFEPYYSRARIIATKITLFLVVLDDTYDAYATIDEIRLITD
AINRWEMSAIDQLPQYIKPFFRILLNEYDDLEKEYSKDGRAFSVHASKQA
FQEIARGYLEEAEWLNNNYVATFPEYMRNGLITSAYNVISKSALVGMGAI
ADEEALAWFETHPKILKASELISRLQDDVMTFQFERKRGQSATGVDAYIK
EYNVSEEVAIKELMKIIENAWKDINEGCLKPTEVSMALLTPILNLARMID
VVYKFDDGFTFPGKTLKDYITLLFVSPPPSLESC
>NjTPS60
MLESMDDGEISVSAYDTAWVALVEDINGSGAPQFPSSLDWIADNQLPDGS
WGDRYIFSAFERIINTLACIIALRTWNIHHQKGEKGMLFIKENIGKLEEE
NEEHMPIGFEVAFPSLVEIAKNLGIQIFPEESAVLQEIYARRNLKLTRIP
KDIMHKIPTTLLHSLEGMKELEWEKLIKLQCKDGSFLFSPSSTAFALIHT
HDSNALSYLTNTVHKFNGGVPSVYPVDLFEHIWAVDRLQRLGISRHFKSQ
IKDCLDYVHKYWREEGICWARNSPVQDIDDTAMGFRLLRLHGYPVSADVF
ENFKNGEEFFGFNGQSNQAVTGMYNLLRASQIVLPGEKILEDAKKFSYQF

LKHKRAANQILDKWIITKDLPGEVSCALDMPWYASLSRVETRYYLEQYGG
QDDVWIGKILYRMSKVNNNTYMELAKLDYNNCQAMHLVEWSRIKQWYEEC
KLGEYGASQESLVLLAYYLASASIYEPEKSKERIAWAKTTTLMETIMSYF
GTKNMSIEQRRSFVRDFKKCSNNLHFTYGRYKTGQGLMGVLLDTVYQFSL
EALRTHGRDIHRHLTQAWETWMLTWQEEGDVYRGQSELVVRTINLCAGSC
SSEEQLLNMMSHPQYHRLSQLTNKICHRLRLFQSHKEYEGESINTENGGI
NEEEIEADMRELVEAVIYNEDDNVDGNIKQTFLTVAKSFYYTAYCNPHTI
SLHISKVLFESLF
