# Supplementary material for: Metabolome and transcriptome associated analysis of sesquiterpenoid metabolism in Nardostachys jatamansi
Source: Front Plant Sci. 2022 Nov 29;13:1041321. doi: 10.3389/fpls.2022.1041321 (PMC9746346; doi:10.3389/fpls.2022.1041321)
Supplement: Additional file 1 — This file includes all additional tables ( Tables S1 - S8 ) used in this manuscript. Table numbers and titles were listed as follows: [file DataSheet_1.zip › Data Sheet 4.pdf]

>NjCYP51G1
MGETEVDNKVLNMGLVIVATIVIAKLISILVMPRSSKRLPPVVKAWPVLG
GLVRFLKGPVIMLREEYPKLGSVFTLSFLNKNITFLIGPEVSAHFFKASE
SDLSQQEVYQFNVPTFGPGVVFDVDYSVRQEQFRFFTESLRVSKLKGYVD
QMVMEAEQYFSKWGDSGEVDLKYELEHLIILTASRCLLGEEVRNKLFEDV
SALFHDLDNGMLPISVIFPYLPIPAHRRRDQARKKLSSIFANIINSRKQT
SKSENDMLQCFIDSKYKDGRPTTESEVTGLLIAALFAGQHTSSITSTWTG
AYLLRHKGFLSQVVEEQKNIMKKHGNKVDHDILSEMEVLYRCIKESLRLH
PPLIMLLRSSHSDFTVTTREGKNYDIPKGHIVATSPAFANRLPHIYTEPD
SYDPDRFSPGRDEDKTAGAFSYISFGGGRHGCLGEPFAYLQIKAIWSHLL
RNFEFELVSPFPEIDWNAMVVGVKGKVMVKYKRRVLSVE
>NjCYP51G2
MEVDSKILNVGALLIATLVAAKIIAAFLIPRSRKRLPPVVKAFPVVGGLL
RFLKGPIVMLRQEYPKLGSVFTLNLVNKNITFLIGPEVSAHFFKASESDL
SQQEVYQFNVPTFGPGVVFDVDYSVRQEQFRFFTESLRVNKLKGYVDHMV
FEATEYFSKWGESGEVDLKDELEKLIILTASRCLLGEEVRNKLFDDVSAL
FHDLDNGMLPISVIFPYLPIPAHRRRDQARKKLADIFAKIISSRKQSGKS
ENDMLQCFIDSKYKNGRPTSESEVTGLLIAALFAGQHTSSITSTWTGAYL
LCNQKHMAAVVDEQKTLVQKHGDKVDHDILSEMNVLYRCIKEALRLHPPL
IMLLRSSHSDFTVTTKEGKDYDIPKGHIVATSPAFANRLPHIFKNPDTYD
PDRFLPDREEDKAAGAFSYISFGGGRHGCLGEPFAYLQIKAIWSHLLRNF
ELELVSPFPEIDWNAMVVGVKGKVMVRYKRKVLQ
>NjCYP71A1
MFTLQQISFSTLLPLLFTTLFLIKWLFSTKKTLKNLPPSPQGLPMIGNLH
QLGSIPHRSLRSLAQKHGPLILLHLGRVPVLVVSSSDAAREIMKTQDLIT
ANRPRSRITDVLIYGSKDITFTTYGEYWRQLKSIIVLHLLSTKRVNSYRQ
VRDEEVALMVENITKICSSSNPLLNLSDVFMSLPNNVVCRIVTGRIYGEG
DNGRKFKELLKELVELLGVFKVGDYIPWLSFVDRITGLEARLDRVANELD
EILESVVSDHMEKEEGDNGEGGQDFVDILLEIQKENKIGFSIQKDTVKAI
ILDMFAAGTDTTSTMLEWAMAELLRHPNVMKKLQTEIREMVQNKSKVTED
DLERMQYLKMVIKETLRLHAPLPLLILRESSQDFKIMGYDIRAGTQVIIN
AWAIGRDASFWKDPDEFRPERFLNTCMEDKGQNFCPDKRGWMAAPDGSFS
TRSFFATLVKPPTGGDFQVANFLWKGTVPTKIKIFGWLAMYGKISTADVI
QHRRPDLAISPNWCYLCRASTESVNHILISCTFAWSLWARLFNCFGIQGV
IPRSWLEFMGNYKLRKQLWRFFSLALLWRIWLERNNRIFNGKGRSSEDIW
FQVCVLVGNWAISCKIFDSVDNYLFCLDPFAFLH
>NjCYP71A2
MFTLQQISFSTLLPLLFTTLFLIKWLFSTKKTLKNLPPSPQGLPMIGNLH
QLGSIPHRSLRSLAQKHGPLILLHLGRVPVLVVSSSDAAREIMKTHDLIT
ANRPRSRITDVLTYGSKDITFTTYGEYWRQLKSIIVLHLLSTKRVNSYRH
VRDEEVALMVENITKICSSSNPLLNLSDVFMSLSNNVVCRIVTGRIYGEG
DSGRKFKELLKVFVELLGVFKVGDYIPWLSFVDRITGLEARLDRVANELD
EILESVVSDHMEKKEGDNGEGGQDFVDILLEIQKENKIGFSIQKDTVKAI
ILDMFAAGTDTTSTMLEWAMAELLRHPNVMKKLQTEIREMVQNKSKVTED

DLERMQYLKMVIKETLRLHAPLPLLIPRESSQDFKIMGYDIRAGTQVIIN
AWAIGRDASFWKDPDEFRPERFLNTCMEDKGQNFDLIPFGAGRRGCPGIL
FGVTIGELVLANLVHKFDFALPNGTEVENLDMSENNGLVVHMKSPLVVTA
TPYC
>NjCYP71A3
MFFLQEFSFLILLPLLCTITLFVIKWLLSTKKSLNNLPPSPRGLPIIGNL
HQLGSIFHRSLHSLAQKHGPLIRLQLGRVPVLVVSSSDGAREIMKTHDLI
TSNRPHSRITDVLIYSSKDITFSSNGEYWRQVKSIAVLHLLSNKRVNSYR
HVRDEEVGLMVENIIKNPLVNLSDVFMSLPNNVVCRVVTGKKYGEGDSGR
KFKELLMEFVELLGVFKVGDYISWLSFVDRITGLEARLNRVANGLDDILE
GVISEHMEEKGGDKGDDFVDILLEIQKQNQTAFSFQTDTIKALILDMFAA
GTDTTYIMLEWAMAELLRHPSVMKKLQTEIREMVQNKSKVTEDDLERMQY
LKMVIKETLRLHPPLPLLVPRESSQDFKIMGYDIRAGTQVIINAWAIGRD
ASFWKDPHEFKPERFLSTCIEYKGLNFDFIPFGAGRRGCPGILFAITIGE
LGLANLVHKFDFALPNGAKVENLDMRENSGFTLHMKSPLVVTATPYHLQ
>NjCYP71A4
MMFNILLLSFVTLLLFIAPLFKLFRNHKKNLPPSPSKLPFIGNFHQLGNY
PHRSLQKLAQKHGPLILLHLGQVPVLVVTSPNAAQEIMKTHDQIFSSRPK
ATIPSKLLYGSIDMAFSPYGEYWRQVKSIGVVNLLSNKRVQSYKTVREEE
MSVMVEKIIEASNNNNGLINITDMFNSLTNDVVSKVALGFKLSDTENGRR
IKKLLGELGEYLGVFCIGDYVPCLWWVDRVNGLFGRVERVAKGIDEILQG
IINDHIHKLGESEDGDGNKNFVEILLEIQTQNKLGFPISTDTIKALLLDM
FSAGTDTTYTLLEWTVAELLRHPQIMIKLRNEVRQVVKNKPNVTEEDLEK
MSYLKVVIKETLRVHPPVPLLVPRESTQDAKILGYDIEAGTQVFINAWAI
GRDPSVWEDPEEYKPERFLNNSVDFKGFNFEFIPFGAGRRGCPGIQFAIA
VAELALATLLYKFDIALPYGEKAEDMDMTETTGITCHKESPILVVTTPYL
>NjCYP71A6
MMFNILLFSFIALLLFIAPLFKWFRNHQKNLPPSPSKLPIIGNFHQLGNY
PHRSLQKLAQKHGPLILLHLGQVPVLVVTSPNAAREIMKTYDQIFSSRPK
ATIPSKLLYNSQDMAFSPYGEYWRQVRSIGVLNLLSNKRVQSYKTVREEE
TRVMVEKIIEASNNNQVININEMFNSLTNDVVSRVALGFKVSEGEKGRRF
KELLGQLGQLLGVFCIGDYVPWLWWLDRVNGLFGKVEGVAKGIDEFLEGI
VNDHMEKMKKGESEEDGKNFVEILLDIQRENKLGFPISTDTIKALILDMF
AAGTETTSIGMEWMVAELLRHPQIMIKLRNEVRQVVKNKPNVTEEDLEKM
SYLKVVIKETLRLHTPVPLLVPRESTEEAKILGYDVEAGTQVIINAWAIG
RDPSVWEDPEEFRPERFLNSCIDFKGFDFELIPFGAGRRGCPGTQFGIAV
TELALATLLYKFDIALPDGKRAEDLDMTETPGVNAHTKSTIFVVATPYVG
>NjCYP71A8
MMFNILLLSFITLLLFIAPLFKWFRNQKKNLPPSPSKLPFIGNFHQLGNY
PHRSLQKLAQKHGPLILIHLGQVPVLVVTSPNAAREIMKTHDQIFSSRPK
ATIPSKLLYNYKDMAFSPYGEYWRQVKSIAVVNLLSNKRVQSYKTVREEE
TRVMVEKIIEASKNNQVININDMFKSLTNDVVSRVALGFKVSEGEKGRRF
KELLGQLGQLMGVFCIGDYVPWLWWLDRVNGLFGKVEKVAKGIDEFLEGI
VNDHLEKMKKGESEEDGKNFVEILLDIQTENKLGFPISTDTIKALILDMF

AAGTDTTSIGMEWMVAELLRHPQIMIKLRNEVRQVVKNKPNVTEEDLEKM
SYLKVVIKETLRLHTPIPLLVPRESTQEAKILGYDVEAGTQVIINAWAIG
RDPSVWEDPEEFRPERFLNNSIDFKGFDFELIPFGAGRRGCPGTQFGIAV
TELALATLLSKFDIALPDGKRAEDLDMTETPGINAHTKSTIFVVARPYVG
>NjCYP71A11
MMFNILILLSFITLLLLLFIADPLYKLFRNLKKNLPPSPSKLPVIGNFHQ
LGNYPHRSLQKLAQKHGPLILLHLGQVPVLVVTSPNAAREIMKTYDQIFS
SRPKATIPSKLLYNSKDIAFSPYGEYWRQVKSIGVLNLLSNKRVQSYKTV
REEETRVMVDKIIEASNNNELININDIFKSLTNDVVSRVALGFKISEGEK
GRRFKELLGEFGQLLGVFCIGDYVPWLWWLDRVNGLFGKVEKVAKEIDEF
LEGIVNDHLEKMKKLGESEEDGKNFVEVLLDIQRQNKLGFPISTDTIKAL
ILNMFAAGTDTTSIGMEWIVAELLRHPHIMIKLRNEVRQVVKNKPNVTED
DLEKMSYLKLVIKETLRLHTPAPLLVPRESTEEAKILGYDVEAGTQVIIN
AWAIGRDPSVWEDPEEFRPERFLNSCIDFKGFDFELIPFGAGRRGCPGTQ
FGIAVTELALATLLYKFDIALPDGKRAEDLDMTETPGINPHTKSTIFVVA
TPYVG
>NjCYP71A16
MMFLSFDLHLFPLIPMIIVPLLFILHKNLTSSATKTHKNQPPSPRKLPII
GNLHQLGSKPHGFLQTLTQTHGPLVLIQLGSVPVLVASSPESAREILKTH
DAVFASRPILKIPDTLTYGSKSIAFSPYGEYWRQARSIAVLALLSSRRVQ
SFKKVREEETRFMIDKIGESCGSVFDLGELINSLTNNVVCRVALGRTYHG
TNVNDLLARFVYLLGAFSVGNYIPWMSWVDRLSGLEGRTQKIAKEFDDVL
EVALEEHINKKRVVKGENDESHDLVDILLDAQRENTTSFTLHRDIIKAVI
MDVFAAGTDTTFAAIEWAISELIKNPKVMKNLQKEIREIAQGKSMISEED
LEKMHYLQAVIKESLRLHTPLPLLIFRETIQDVKLMGYDIKAGTQVFINA
WAIGRDPSLWEEAEKFKPERFLNSSVDYKGMSFEFLPFGAGRRGCPGIQF
ATVIVELVLANLVYRYDFSLPDGVGGEELDMSEIGGLTLHRKSPLLVVAT
RRF
>NjCYP71A17
MFSFSKNSLLFYFPFIISIFFISFKWISSRIKTNKNLPPSPPKLPIIGNL
HQLGSSPHRSLHALSQKHGPLMLMQFGSVPVLVASSPETAKEIMKTHDLN
FCNRPKLKIPDILVYGSNDITFSPYGDYWRQVKSIAVIHLLNNTRVQSFR
QMREKEVASTLDLIEKNSGSLVDLSELLFWLTNNIVCKAALGRSYRGLKL
EGLLERFVQVLGAVSLGTYIPWLSWMDRLSGLDEKAHKVAKEFDDFLEGV
VAEHVDKRRHGKNGEDDQDLVDILLDVQRENTSGFTFHRNTVKALILDVF
AAGTDTTFASLVWSISELLRHPKVMKKLQQEVTKIAQGRPMIQEKDLDNM
QYLKAIIKETLRLYPPIPLLIPHESTQDVKILGFDIPSGTQTVVNAWAIG
RDPTVWENPEEFRPERFLNSSIDYKGLHFELLPFGGGRRGCPGISFATII
FELVLANVIYKFDLGLPDGVKDKDLDMREKSGVVTLHKESPLLVVATPRF
H
>NjCYP71A18
MSSLMHQETMVPLVYATPLLLLVFFLINFYYFTRPSAKLNLPPSPPKLPV
IGNIHQLGPILHRSLHNLSHRYGGPLMLMHLGSVPTLVISSAEAAREIMK
TQDLNFATRPDVRRWHQILYDQKEVSVAPYGEEWRQFKSIMVLHFLSKSK

VEAYREVREEETAIAVEKIEKSCDLQEAVNLSEMFSKLTNDVVCRVTFGK
KYSEGENERKFNKMLKEYFDVLSELNFEDMIPWLWWVDRLRGTAARVEKV
AKDVDEFLDGLVEERLRKHSTGGGGGDVDGREDFLDILIKIQKEGVNSLL
DRDAMKGLLLDVYTAGVDTTTTVLEWVFAELLKHPKNFKKLQEEVRTVLQ
DKKRITQQDLDNMKYLKAVIKETLRLHPPAPTLIPRVSREDAKVMGYDVK
KGTRVIINGWAIQRDPKVWDEPDEFQPERFLNNSIDYKGQDFDLIPFGAG
RRGCPGMAFALAIDEQVLATLLHKFDWELPNGGKEEDLDMEEEPGITVHK
KIPLLAMAKRFSS
>NjCYP71A19
MWSFLEQAPIYTLPLFLAAAFVLFRWYNSTSSSTTTTAAKKLPPSPPKLP
VIGNLHQLGASVHHSFFSLAKRYGDSLMLLHIGAVPSLVVSSSDAAREIM
KTNDIAFASRPNTRMFRAISYNLKEITVAPYGEYWRQAKSILTLQLLSNK
KVQTYNGIREKTIADCVDKITKCYVSNTPADLSDLFSSLTNDITCMATFG
KTYNDGEIGRKFKKIMQEFSEVLGSFYFEDSIPQLAVIDRLRGLSAKVDR
VADDFDVFLQGVVDETIEKVSKNPEQIGEDGVETFIEGLLKVQKEDIIGI
TIDADVIKALLLDAYVAGTDTSSSVLEWAMTELLLHPDELKKVQDEVRGI
LNGKEEITDEDLDKMTYLKAVIMETTRLHPPLPILPPRVARHDAEVMGYH
IAEGTRVYVNVYAIMRDPKVWENPESFLPERFLDSSIDFVRHNFELLTFG
AGRRGCPGRVFAMAINEKVMATVLSRFDWSLPSGVRPKDVDMNETFGLAN
HRKVPLLALGKPVTMVGK
>NjCYP71AV5
MEISVTATLGLAAIIFILFKLVTRPKSKKNLLPEPWRLPIIGHMHHLIGT
MPHRGVMDLARKHGPLMHLQLGEVSTIVVSSPRWAKEVLTTYDITFANRP
ETLTGEIIAYHNTDIVLAPYGEYWRQLRKLCTLELLSNKKVKSFQYLREE
ECWNLVKTIRSTGQGPVNLSENIFKMIATILSRAAFGKGIKDQKKFTELV
KEILRLTGGFDVADIFPSKTLLHHLSGKRAKLTNIHNKLDSLIDNIIAEH
PGNRTTSSQETLLDVLLRLKESVEFPMTADNVKAVILDMFGAGTDTSSAT
VEWAISELIRCPRAMEKVQAELRQALNGKERIQEEDIQELNYLKLVIKET
LRLHPPLPLVMPRECREPCVLGGYDIPSKTKLIVNVFAINRDPEFWKDGE
TFIPERFENSPITVMGAEYEYLPFGAGRRMCPGASLGLANVELPLAHILY
YFNWKLPNGIQDLDMTESFGATVQRKSELVLVPTDFKTLEEST
>NjCYP71AV6
MELSVTTSIALATIVFFIYKLATRPKSTKKLLPEASRLPIIGHMHHLIGT
MPHRGVMDLARKHGPLMHLQLGEVSTIVVSSPKWAKEILTTYDITFANRP
ETLTGEIIAYHNTDIVLAPYGEYWRQLRKLCTLELLSVKKVKSFQSIREE
ECWNLVKEVKESGSGNPISLSESIFKMIATILSRAAFGKGIKDQKEFTEI
VKEILRQTGGFDVADIFPSKKFLHHLSGKRARLTSIHKKLDTLINNIVAE
HHVSTSSKANETLLDVLLRLKDSAEFPLTADNVKAIILDMFGAGTDTSSA
TVEWAISELIRCPRAMEKVQAELRQALNGKDKIQEEDIQELPYLNLVIRE
TLRLHPPLPLVMPRECREPVNLAGYDIANKTKLIVNVFAINRDPEYWKDA
EAFIPERFENNPNNIMGADYEYLPFGAGRRMCPGAALGLANVQLPLASIL
YHFNWKLPNGASHDQLDMTESFGATVQRKTELILVPSF
>NjCYP71AV7
MEFSYTTLFGLALFVFFLRKLFTPSKAKQNLPPEPWRLPIIGHMHHLIGS

LPHVGLRDLAKKYGPLMHLQLGELSTIIISDPRWAKEVLTTHDIAFADRP
VVLTTEIVAYQNTDVVWSPYGDYWRQLRKLMTLELMSVKKVKSFHYIRED
ECYNFLVKTIRESGGAPVNLSQMIFDTIARIVCRTSFGKGCKDQEEFIDI
VKELFHLTGGFDVADVFPSKKIIHTLTGKRQKLENIHKRLDKILTDVITQ
HPGQHKESNEVESLLDVLLRLQASGEFKLTTKNVKAVTLDMFGGGTDTSS
ATLEWAVSELIRNPRVLKKAQAELRDALKGKERITEADIQDLDYLKLVAR
ETLRMHLPLPLLFPRECREACKLGGYDIPVGTKLMVNGWAINRDPQFWAN
PDSFIPERFSDNPTNVLGSEFEYLPFGAGRRMCPAAVLGIANVEVPLAHM
LYYFDWELPNGAKGDDLDMVELFGASVQRKNELYVVMKPHNFAKN
>NjCYP71AV8
MEFSYTTFLGLALFVFFLRKLFSPSKAKQNLPPEPWRLPIIGHMHHLIGS
LPHVGLRDLAKKYGPLMHLQLGELSTIIISDPRWAKEVLTTHDIAFADRP
VVLTTEIVAYQNTDVVWSPYGDYWRQLRKLMTLELMSVKKVKSFHYIRED
ECYNFLVKTIRESGGAPVNLSQMIFDTIARIVCRTSFGKGCKDQEEFIDI
VKELFHLTGGFDVADVFPSKKIIHTLTGKRQKLENIHKRLDKILTDVITQ
HPGQHKESNEVESLLDVLLRLQASGEFKLTTKNVKAVTLDMFGGGTDTSS
ATLEWAVSELIRNPRVLKKAQAELRDALKGKERFTEADIQDLDYLKLVSR
ETLRMHLPLPLLFPRECREACKLGGYDIPVGTKLMVNGWAINRDPQYWAN
PDSFIPERFSDNPTNVLGAEFEYLPFGAGRRMCPAAVLGIANVEVPLAHM
LYYFDWELPNGAKGDDLDMVELFGASVQRKNELYVVMKPHNFAKN
>NjCYP71B1
MDNFTPRLPYILFIAAAAAATTAYLLRRRQTCTKPSPDTTPPSPRKLPII
GHLHLLTDMPHHSFTRLAQKLGPIIYLQLGRVPTLLISSPELARLVLRNH
DHVFSNRPQIIAAQYLSFGCSDVTFSSFGPYWRQVRKICVTELLSPKRVN
SFHFIRDEEINRLINAVNNGAGGVINVSELLFGLANDILCRVAFGKRFMG
EDRERKKGDLASVLTETQALLAGFCIGDFFPEWEWVNSVSGMKRRLLKNL
EDLSRVCDEIISEHVEKKKKKEIAENEVSSREDFVDVLLRVKEREDLEVP
ITDDNLKALILDMFVAGTDTTSATLEWIMTELVRHPEVMKIAQKEVRDIV
AHKGKVEESHLQNLHYLKSVIKETMRLHPPVPLLVPRESMEKCTLNGYEI
PKKTRVLINTYAIGRDPQSWESPLEYKPERFEEEDIDFRGQDFRFLPFGG
GRRGCPGFAFGLATVELALASLLYHFDWELLPGQGPNHVDLTETFGLATR
KKTALLLIPTINKSM
>NjCYP71C1
MDSLYSILIPLLLLFLFFLLQWKNSPKNLPPGPPKLPLIGSLLHMGQLPH
RSLKDLATKYGPLMHIQLGEISAIVVSSPRVAKEVTKTHDLSFASRPIIL
ASEIVGYNNTDIAFAPYGDYWRQMRKIATLELLSAKKVRSFCSIREDEAR
NLIQSIHSSSTTGLPFDLTEKVFSLTNNVICRATFGDRYKDQDYLIKILK
QVVTLAGGFDVADLFPSIKLLHLVTGMRPKLQNLRKDLDRIFDSIIDERT
EKRKNGTSSGNEDEEDIVDVLVRLKESGALQFPITQSNIKAVILDMFLAG
SDTSSTTIEWAMAEMMRNPRVMEKAQADLRQAPRGKQEIEESDIKEFDGY
FKFVIKETLRLHPPVALLLPRECREECEIDGYTIPVKTKVMVNAWAIGRD
PEHWKDADSFYPERFENSDVDYLGSNYEFIPFGSGRRICPGMTFGLANVE
LPLANLLYHFNWKLPNGMKPEDVDMSEEFGASLRIRNNLHVIATRQTYSS
>NjCYP71C4

MDSLYSILIPLLLLFLFFLLRWKNSPKNLPPGPPKLPLIGSLLHMGQLPH
RSLKDLATKYGPLMHIQLGEISAIVVSSPRVAKEVTKTHDLSFASRPIIL
ASEIVGYNNTDIAFAPYGDYWRQMRKIATLELLSAKKVRSFCSIREDEAR
NLIQSIHSSSTTGLPFDLTEKVFSLTNNVICRATFGDRYKDQDYLIKILK
QVVTLAGGFDVADLFPSIKLLHLVTGMRPKLQNLRKDLDRIFDSIIDERT
EKRKNGTSSGNEDEEDIVDVLVRLKESGALEFPITQSNIKAVILDMFLAG
SDTSSTTIEWAMAEMMRNPRVMEKAQADLRQALKGKQEIEESDIKEFDGY
FKLVIKETLRLHPPVALLLPRECREECEIDGYTIPVKTKVMVNAWAIGRD
PEHWKDADSFYPERFENSDVDYLGSNYEFIPFGSGRRICPGMNFGLANVE
LPLANLLYHFNWKLPNGMKPEDVDMSEEFGASLRIRNNLHVIATRQTYSS
>NjCYP71C5
MDSLYLILINIIIPLLLLLLFLFFLLRWKNSPKNLPPGPPKLPLIGSLLH
MGQLPHRSLKDLATKYGPLMHIQLGEISAIVVSSPRVAKEVTKTHDLSFA
SRPIILASEIVGYNNTDIAFAPYGDYWRQMRKIATLELLSAKKVRSFCSI
REDEARNLIQSIHSSSTTGLPFDLTEKVFSLTNNVICRATFGDRYKDQDY
LIKILKQVVTLAGGFDVADLFPSIKLLHLVTGMRPKLQNLRKDLDRIFDS
IIDERTEKRKNGTSSGNEDEEDIVDVLVRLKESGALEFPITQSNIKAVIL
DMFLAGSDTSSTTIEWAMAEMMRNPRVMEKAQADLRQALKGKQEIEESDI
KEFDGYFKLVIKETLRLHPPVALLLPRECREECEIDGYTIPVKTKVMVNA
WAIGRDPEHWKDADSFYPERFENSDVDYLGSNYEFIPFGSGRRICPGMNF
GLANVELPLANLLYHFNWKLPNGMKPEDVDMSEEFGASLRIRNNLHVIAT
RQTYSS
>NjCYP71C7
MEEFTKNLPPGPPKLPLIGSLLHMGQLPHRSLKDLATKYGPLMHIQLGEI
SAIVVSSPRVAKEVTKTHDLSFASRPIILASEIVGYNNTDIAFAPYGDYW
RQMRKIATLELLSAKKVRSFCSIREDEARNLIQSIHSSSTTGLPFDLTEK
VFSLTNNVICRATFGDRYKDQDYLIKILKQVVSLAGGFDVADLFPSIKLL
HLVTGMRPKLQNLRKDLDRIFDSIIDERTEKLKNGTSSGNEDEEDIVDVL
VRLKESGALEFPITQSNIKAVILDMFLAGSDTSSTTIEWAMAEMMRNPRV
MEKAQADLRQALKGKQEIEESDIKEFDGYFKLVIKETLRLHPPVALLLPR
ECREECEIDGYTIPVKTKVMVNAWAIGRDPEHWKDADSFYPERFENSDVD
YLGSNYEFIPFGSGRRICPGMNFGLANVELPLANLLYHFNWKLPNGMKPE
DVDMSEEFGASLRIRNNLHVIATRQTYSS
>NjCYP71C9
MSLVCAMDITVPLFIFIIFLFFLFKSVNEKSSGGSPLPPGPRKLPLIGSL
LHTMGPLPHRTLKDLASKHGPLMHLQLCQISAVVVSSPRVAKQVLKTHDL
SFASRPSLLSTEIILYNGKDIVFAPYGDYWKQMRKIATMEFLSAKKVRSF
NSIRKEEVQNLVESINSSSSGSLPIDLTQKIFSLIRGIVARSSFGNKCKD
QDSFIKLTNEVISLVGGFYVADLFPSFKLLHLVTGMRPKLENLGKDIDRI
FDTIVNDRIQEKANNTALGTEGLLDVLLRLKDDGGLEFPITLINIKAIIL
DLFIAGTDTSTVLIEWAMAELLKDPRVMEKAQAELRQALKGKQVIEESDI
MELGYLKLVIKETLRLHPPAPLLLPRECREECEIDGYTIPIKTKIIINAW
AIGRDPEYWKDAERFYPERFENGGVDFMGSNYEFIPFGAGRRMCPGITFG
LASVELPLANLVYHFNWKLPNQMKSEDLDMSESFGTTVKKRNHLCLIATR

QTPAL
>NjCYP71C10
MDLTVPLFIFIIFLFFLFKSVNKKSSGGSPLPPGPRKLPLIGSLLHMMGP
LPHHTLKDLASKHGPLMHLQLGQISAVIVSSPPVAKQVMKTHDLSFASRP
SILASEIVLYNGKDILFAPYNDYWRQMRKIATMELLTAKKVRSFNSIREE
EVQNLVESINSSSSGSLPIDLTQKIFSLISGIAARSSFGNKCKDQESFIK
LTKEVISLGGGFDVADLFPSFKLLHLVTGLRPKLENLGKDIDRIIDTIVN
DRIQEKANNTALGTEGLVDVLLRLKDDGGLEFPITLINIKAIILDVFMAG
TDTSSVLIEWAMAELLKDPRVMEKAQAELRQALRGKQVIEESDIKELGYL
KLVINETLRLHPPIPLLLPRECREECEIDGYTIPVKTKIIINAWAIGRDP
KYWKDAERFYPERFENGGVDFMGNNYEFIPFGAGRRMCPGITFGLASVEL
PLANLLYHFNWKLPNEMKSEDLDMSESFGATVKKRNHLCLIATRQTPAL
>NjCYP71C11
MDLTVPLFIFIIFLFFLFKSVNKKSSGGSPLPPGPRKLPLIGSLLHMMGP
LPHRTLKDLASRHGPLMHLQLGQISAVVVSSPPVAKQIMKTHDLSFATRP
SLLSTEIVLYNGKDIVFAPYGDYWRQMRKIATMELLSAKKVRSFSSIREE
EVQNLVESINSSSSGSLPIDLTQKIFSLIIGIAARSSFGNKCKDQDSFIK
LIYEVISLGGGFDVADLFPSFKLLHLVTGMRHKLENLGKDIDRIIDTIIN
DHIQEKRNNTALGTEGLVDVLLRLKDDGGLEFPITLINIKAIILDVFMAG
TDTSSVLIEWAMAELLKDPRVMEKAQAELRQALRGKQVIEESDIKELGYL
KLVINETLRLHPPIPLLLPRECREECEIDGYTIPVKTKIIINAWAIGRDP
EYWKDAERFYPERFENGGVDFMGSNYEFLPFGAGRRMCPGITFGLASVEL
PLANLLYHFNWKLPNEMKFEDLDMSESFGATVKKRNHLCLIATRQTPAL
>NjCYP71C15
MSLVCAMDLTVPLFIFIIFLFFLFKSVNKKSSGGSPLPPGPRTLPLIGSL
LHMMGPLPHRTLKDLASKHGPLMHLQLGQISAVVVSSPPVAKQVMKTHDL
SFASRPSILASEIVLYNGKDIAFAPYSDYWRQMRKIATMELLSAKKVRSF
HFIREEEVQNLVESINSSSSESRPIDLTQKIFSLISGIAARSSFGNKCKD
QDSFIKLIYEVISLLGGFDVADLFPSFKLLHLVTGMRRKLENLGKDIDRI
IDTIVNDRIQEKRNNTALGTEGLVDVLLRLKDDGGLEFPITLINIKAIIL
DVFIAGTDTSSVLIEWAMAELLKDPRVMEKAQAELRQALRGKQVIEESDI
KELGYLKLVINETLRLHPPIPLLVPRECREECEIDGYTIPVKTKIIINAW
AIGRDPEYWKDAERFYPERFENGGVDFMGSNYEFIPFGAGRRMCPGITFG
LASVELPLANLLYHFNWKLPNEMKSEDLDMSESFGASVKKRNPLCLIATR
QTPAL
>NjCYP71C17
MSLVCAMDLTVPLFIFIIFLFFLFKSVNKKSSGGSPLPPGPRRLPLIGSL
LHMMGPLPHRTLKDLASKHGPLMHLQLGQISAVVVSSPPVAKQVMKTHDL
SFASRPSILASEIVLYNGKDIAFAPYSDYWRQMRKIATMELLSAKKVRSF
DFIREEEVQNLVESINSSSSESRPIDLTQKIFSLISGIAARSSFGNKCKE
QDSFIKLIYEVISLLGGFDVADLFPSFKLLHLVTGMRRKLENLGKDIDRI
IDTIVNDRIQEKRNNTALGTEDLVDVLLRLKDDGGLEFPITLINIKAIIL
DVFIAGTDTSSVIIEWAMAELLKDPRVMEKAQAELRQALRGKQVIEESDI
KELGYLKLVINETLRLHAPIPLLVPRECREECEIDGYTIPVKTKIIINAW

AIGRDPEYWKDAERFYPERFENGGVDFMGSNYEFIPFGAGRRMCPGIKFG
LASVELTLANLLYHFNWKLPNEMKSEDLDMSESFGASVKKRNPLYLIALR
ETPAL
>NjCYP71C22
MDPFSLSTALISFFFIATLFMLKLVNAKDKPPLNTPPGPWKLPIIGSMLH
LIAGGSLPHRILKHLASKHGPLMHIQLGEISAIVVSSPKMALEVLKTHDL
GFSSRPELLIGQKVFYNCTDVAGAPYGAGAPYGAYWRQMRKICTLKLLST
NKVRSFRSIRENEVSKFVDLIRQSTGALNLSEEIFSLTYKIVCRAGFGSI
GREYEHTFKLLMHEIVYLAAGFHVSDLFPSFKLIHLLTGMKPKLEKLHRK
VDRILDDVIDQHRRKIMVKSSKIDLADDGGGGGVDRGDEDLVDVLLKVAE
SDDLEIPITSENIKAVILDMFVGGTDTSSTVLEWAMSELIRNPRVMKKAK
VELKNAFKGKKVIFETDIQDLMYLKLVIKETLRLHPPAPLLVPRECREQC
NVNGYDIPVKTKVIVNAWAIGRDPVYWEDSECFFPERFENSTSIQNNYLG
NDYEFIPFGAGRRMCAGIMFGLANVELPLAQMLYHFDWKLPHEMDGRNLD
MSETFGMVCNRRDALYLIATPYLVA
>NjCYP71C23
MDLKSSSSVVNFNVVPVLLLLLSFLFFLYKYGGLKSGNNNRVKRLPPGPW
KLPVIGSLSHMVGPLPHRTLKNLATKHGPLMHLQLGQSPAIFVSSARVAK
EVLKTHDVSLASRPKILAAEIVGYNCTGIGFAPYGDYWRQMRKICTSELL
SSKKVRSFRSIREDEVRNLIESIRSSSSRSLPIDVSEKIFSLTNGIVCRS
TFGNRCKDQDSLIKIIKELISLAGDLDVSDMFPSFKFLHVIFSMRPKLEK
LRQYLDRIFDSIINERIQEKGTNTGDHTEDLVDVLLRLKDSGGLEFPITH
VNIKAIILDLFIAGTDTSSTTIEWAMSEIIRNPRVMEKVQAELRRPLKGK
QEIEESDIKEFCYLKLVIKETLRLHPPVVLLVPRECREECEIDGYTIPVG
TKVMINAWAIGRDPEHWKDADSFYPERFENSDVDFIGSNYEFIPFGGGRR
MCPGMNFGLANVELPLANLLYHFNWKLPNGMKSEDIDMSESFGGSVRKRN
SLCLIATLHSNSQF
>NjCYP71D1
MEDLEIFSLVFPLLIIILVLVIIKLIFNGSDNNKVVLPPGPWQLPLIGNM
HNLLGFSLPPHHILSELSNKYGPLMFLRLGTVPALVVSSAELAEHVLKTH
GVNFADRPRLLAVKILTYDCTEIAFSPYGDHWRQLRKICAMELLSTKRVH
AFRFTREDEVFNLLQSISSSSSSSSHSVINLSKLIYSLTYSIITRATFGE
KCTDQLEEFSSLLDQAVMLFSGFSIADMYPSIKFLHAVGGFRTRVEKVHR
QMDNTLVNIITAHKERINTTTSGTGELNDQEYLVDVLLRIQKQRDTELPL
TDDIIKGVIFDIFSAGSETSSTTMDWAMSEMVKNPRVMERAQAEVRQVFG
GRRNVDETGLDELKYFHCVIKETLRLHPPGPLLIPRENREECEINGYVIP
AKSRVLVNVWAISRDPKYWGPDAHLFKPERFLDSNTSYDYKGTNFEYIPF
GAGKRICPGMLFGMASMELSLAQLLYHFDWKLPDGLKHEELDMTEEFGVT
TRRKSNLNLIPLPYRSSFLAPVEK
>NjCYP71D3
MELQLQYPLSVSVSLIFSFLLFLFLLTKISIKKKPSSKLPPGPWQLPLIG
NMHQLLGSLTHHILRDLSNKHGPLMFLRLGLVPTIVISSPKIAQEVLKTH
GINFADRPYVMALNIFSYNSLDIGFSEYGDYWRQLRKICTIELFSAKRVQ
TFRSIRESEVLNLVKAIHSNAGSTVNLSKLIFDLTYTITARSALGGKVKD

QELFIKLMEEAVKETAGFSVVDLYPSVKLLQLLSRSRIRLEKLQRKWDKI
LEDVVFEHRKTGEGEMHEDLVDVLLRIQKQGDTEIPLTDEGIKAVIFDIF
TAGSETSSTIVEWAISEMIRNPRVMERAQAEVRKVFDEKRNVDETGLDEL
KYLQSVIKETLRLHPAAPLLVPRQNREECEINGYVIPAKSRVIVNAWAIS
RNPKYWGAEAEIFKPERFLDSSIDYKGTNFEYIPFGAGRRICPGMFLAIA
NIELPLAQLLYHFDWKLGSKQEDLDMTEEFAITVRRKNDLNLIPVPYRRS
LLQECP

>NjCYP71D4

MELQFSLIFSFILFLFILAKISKKKNPSSKLPPGPWQLPLIGNMHQLMGS
LVHHILRDLADKHGQLMFLRLGSVPTIVVSSPEIAEEVLKTHGIVFADRP
YILASRILSYNSTNIAFSPYGDYWRQLRKICTMELLSTKRVLKSRSIREE
EVMNLVRRISSNAGSVFNLSKAIFSLTYTTTARSALGKKNEEQEACELLM
DQAFKALGGFSIADMYPRAKLLHLITGVRRSIEKTQRELDKILQNIIVDH
RTRNESREGDFDEDLVDVLLRIQKNGDLEFPVTDDCIKAVVLDIFSAGSE
TSSTTVEWAMSEMLKNPQVMERAQAEVRKVFDERRNVDETGLDQLKYLQC
VIKETLRLHPSAPLLVPRENSEQCKINGYTIPPKTKVIVNAWAIGRDPRY
WGAESELFKPERFIDSPIDYKGNNFEYIPFGAGRRICPGISFAIVNIELP
LAQLLYHFNWKFPNGSKQEHVDMTEQFGLTVRRKNDLYLVPVPYRLSPLH
AELG

>NjCYP71D7

MIMEFHLPSISLFFSFFIFLIILIKTFKKSNNSTLNLPPGPPQLPLIGNL
HNLLGSSLIHKTLKNLSNKYGPLIYLKLGELPTVIVSSPEIAMQIMKTHD
IVFAQRPTSLVSTILMYNSTGVGFTPNNDYWRQIRKICVMELLSPKRVQT
FRSIREEEVWNLVTDLKSSANYPVNLSKKIFSLTYGITGRAALGQKSRDQ
EQVIELVEVLTKDMAGFSIAELYPSVNFLQVVSGDRRRLEKMHKKMDTLL
QRIVDQHKQGSTRRKTGEEDLVDVLLRIQKHGELEFPLTDNNIKAVIWDI
FTAGSETSSTTVEWAMSEMLKNPRIMKIAQTEIRRVFTPRGNVDETGLNE
LKYLKAVIKETLRLHPSVPLLLPRESNDDCEINGYTIPVKTKVMINAWAI
GQDPRFWPEPETFNPERFLESEIDFKGTNFEYIPFGAGRRICPGILFALP
NIDLPLAELLYHFDWKLDGNLKFEDLDMTESFGLSVRRKNDLYLIPVPYF
PSFAK

>NjCYP71D8

MIMEFHLSSLSLLISFFIFLLILIKTFKKSNSTLNLPPGPPQLPLIGNLH
NLLGSSLTHKTLKNLSNKYGPLIYLKLGELPTVIVSSPEIAMQIMKTHDI
IFAQRPISLFSTILIYNSTGIGFSPNNDYWRQLRKICVMELLSPKRVQTF
RSIREEEVWNLVTDLKSSANYPVNLSKKIFSLTYGITGRAALGQKSRDQE
QVIELVEVLTKDMAGFSIAELYPSVNFLQVVSGDRRRLEKMHKKMDTLLQ
RIVDQHKQGSTKTKTGEEDLVDVLLRIQKHGELDFPLTDNNIKAVIWDVF
TAGSETSSTTVEWAMSEMLKNPRIMKIAQTEIRRVFTPRGNVDETGLNEL
KYLKAVIKETLRLHPSVPLLLPRESNDDCEINGYTIPVKTKVMINAWAIG
QDPRFWPEPETFNPERFLESEIDFKGTNFEYIPFGAGRRICPGILFALPN
IELPLAELLYHFDWKLDGNLKFEDLDMTESFGLSVRRKNDLYLIPVPYFP
SFAK

>NjCYP71D9

MIMEFHLPCVCLLIPFFIFLLILIKTFKKSNSTIKLPPGPPQLPLIGNLH
NLLGSSLIHKTLKNLSNKYGPLIYLKLGQLPTVIVSSPEIAMQIMKTHDL
IFAQRPISLVSTILFYDSAGLIFSPNNDYWRQLRKICVMELLSQKRVQTF
RSIREEEVCNLVTDLKSSENSRVNLSRKIFSLTYGITGRAALGQKSRDQE
QVIELVDIVIKAADGFSIAELYPSVKFLQVMSGARPRLEKMHKKMDTLLQ
RIVDQHKQGSTKTKTGEEDLVDVLLRIQKHGELDFPLTDNNIKAVIWDIF
TAGSETSSTTVEWAMSEMLKTPRIMEIAQTEIRRVFTPKGNVDETGLTEL
KYLKAVIKETLRLHPAAPLLLPRESNDDYEINGYTIPVKTKVMINAWAIG
QDPRFWPEPETFNPERFLESEIDFKGTNFEYIPFGAGRRICPGILFALPN
IELPLAQLLYHFNWKLDRNLKFEDLDMTESFGATVRRKNDLYLIPVPYFP
SFA
>NjCYP71D10
MIMEFHLPSVSLLIPFFIFLLILIKTFKKSNSTVKLPPGPPQLPLIGNLH
NLLGSSLTHKTLKNLSNKYGPLIYLKLGQLPTVIVSSPEIAMQIMKTHDL
IFAQRPISLVSTILFYDSAGLVFSPNNDYWRQLRKICVMELLSPKRVQTF
RSIREEEVWNLVTDLKSSENSPVNLSKKILSLTYGITGRAALGQKSRDQE
QVIELVEIVRKAVAGFSVAELYPSVKFLQVVSGARPRLEKMHKKMDTLLQ
RIVDQHKQGSTKTKTGEEDLVDVLLRIQKHGELEFPLTDNNIKAVIWDIF
TAGSETSSTTVEWALSEMLKNPRIMEIAQTEIRRVFTPKGNVDETGLTEL
KYLKAVIKETLRLHPAAPLLLPRESNDDWEINGYTIPVKTKVMINAWAIG
QDPRFWPQPETFNPERFLESEIDFKGTNFEYIPFGAGRRICPGILFALPN
IELPLAQLLYHFDWKLDRNLKFEDLDMTESFGVTVTRKNDLYLIPVPYFP
SFA
>NjCYP71E1
MATLQWLKEESFITSLLVASVLLVLSLKLIFKRKRNVNNLPPSPPKLPII
GNLHQLGKNPHLSLQNLASKFGPIIYLQLGQIPTVVVSSARTAKEVMKTH
DLALSSRPQIFSAKHLFYNCTNIVFSPYGAYWRHIRKICILELLSAKRVQ
SYSFVRQEEVARLIFRVSESCDGTTDLTKILGLYSNDVLCRVAFGRDYSG
GGDYDRHGFQKMLEEYQVLLGGFNVGDFFPSMEFLNTLTGTTSRLKRTFH
RFDMFFDEVIKEHLNPARQKEEKHKDLVDVLLDIQKSGDTEIPLTMNNIK
AIILDMFAAGTDTTFITLDWGMTELILNPKVMKRAQAEVRTVLKDRKTVL
ESDLIQLKYLKAVIKEIFRLHPPAPLLVPRESMEDINIDGYDIPANTRFF
VNAWAIGRDPESWGDPYKFEPERFTGSSIDFKGQDFELIPFGAGRRGCPA
ITFGTASVEFALAQLLYSFNWELPSGIQPKDLDMTEVFGITMHRISHLIV
VAKPYFP
>NjCYP71F1
MLMTRSSIVHTILAKLAEKYGPIMQLNVGEISMVVISSAELAEAAFTTND
LALAQRPDNVFLETITYNNSGIALSPYGNHWRFFRKLCSNHVLNLKHVQS
FKSIREDEVSKFTNSIYTSKNTVVNLSKKLLRLTSSIVSRATIGRKINSK
SDIDDDCDDDELTFLLNRLMTLRGSFIVYQMFPSLEILHRLSGLRAAAED
IHWKMDKMLDDILEEHKSKCGSGDEEEYLVDVLISLQANPESDVPITRDI
VKAIILDMLIAGLENSATIIEWAMAELLRNPRILQKAQAEVREALKGNTG
IEETNNIQEQKYLKAIVKETLRLHPPIPVIPRETRENCEIGGYLIPKNTK
FFINAWAIGRDPKYWKDDPDCFYPERFLQPMSLNYEYIPFGGGKRMCPGI

SFGITVVELTLAYLLYCFDWKLPNGIKPQELDMTELYLVTAKKKKDLLVV
PIPYIPRRG
>NjCYP71G1
MDIFTNYFPSSLLSTVSILLFSLILIYSLRKKKTSSKAAPNLPPSPPRLP
IIGNLHQVIGKNFHQILWKVSRQYGPIMTVHLGATPYVIISSSEFANQAL
KTHDQILCNRPRSKGFQRLTFDYMDVAFSPHAEQWKEMRKVLVNEFLGSK
RSKLFKKVVVAEVKGMLDSFSSEHLNTTVNMDERLFHLVTDIVSKVAVGK
SYREVTFRGKKLKEMLDDLVISLCGSVSDIYPGRIGLILDEILGFNRRLE
KTFSTFDAFLQMVLDEHIDHTETSGHEKDMIDACRSQLTTNELKALLMNV
FNGAIDTTTTTAVWTMSEIIKNPRVMHKLQEEIRRCVGKKASVEESDIAQ
MPYLKLVVKEALRLHSTVPFLLTRECVKHCQIGGYDIYPGTRVLINAWGI
GRDPKVWTETASVFNPERLENAELDRSEMIPFGGGRRACPASSVATQVVE
YTIANLFYSFDWQLPSGMKNEELDMEEVGSLIVVRKTPLCLVPIKHDW
>NjCYP71H1
MEFFTIFPSWLLTSLLVLSVFCFFLYNARSNRSSMVVPKLPPNPPKLPII
GNLHQLLGKPRHKALWQLSKQYGPLMLLHIGKKPYIVISSSSLAKQVFRN
LDHILCSRPLSKATKRLTYNYLDIAFSPCDNHWKKMRKLLVSEFLGPKRA
RLFNHVLMTEIELMVRSISSYPSNDVVNLNKLFLRTVKAVVCKVAFGNNY
REEPLKGPPWEVMLDESMEILNGSVGDSFPWLGQLIDQFSGWNYKLEKCF
SNLDAYIETIIDDHKNHKIGQVSDDDKDFVHSLLDLSSIEHDNDDRLTKG
DIKALVMDVLTGGIDTTVVTMVWAMSEIIRNPRVMQKLQTEIRNCTRRKQ
QVQEVDITKMSYLKMVVKETLRLYPPAPLLIPHESLSHCQIGGYDVFPGT
CVLVNAWVIGKDRDTWGENAGEFYPERFENLDVDYGGGNFEMVPFGGGRR
SCPAMNTVPVTIESTIANILYWFDWEVPGGLKNEDLNMLEEGSLVVRKKL
PLCLVPKKHKWED
>NjCYP71I1
MDTEIAISLIVSCFFLFLYFILKPSKPLNLPPGPRKLPLIGNVHQLAGKL
PHRAFRDLARKHGPIMHIQLGQISAVIISSPRLAEQVLKTNDIALADRPT
TFGSELVLYGNTDIALAPYGEYWRQMKKIASLELLSAKKVRSFGRIREQE
LNAFMELLRLNSGKPIDIQKTVTEVINNVVCIASFGKNCKQQHALLDFLD
EFARVNTGFYVADLFPDLKFLYVVSGHRSRLMKLYKTLDKIFDDIWEEHE
GGIKDHGGDQEEDLLQVLLRIKEEGGLGFPITNNNIKAIFVDIFAGGTDT
SSITIGWAMTEIIRHPHVMEKVQAEVREAFKGKGKISESELQNLSYLQSV
IKETLRLHPPIPLLLPRVCREQCKIGGYDIPVKMKVFVNGWACSTDPEYW
EDADTFNPERFENASVDFMGTNYHFIPFGSGRRMCPGITFGMVSVELLLA
QMLYYFDWKLPDGLNPADIDMSETEGSLVAKKVHLHLIPTSYVHVS
>NjCYP71I2
MSATMYTEIAFSVIVSCIFLFLYSVLSNNRAKGTKSLNLPPGPTKLPLIG
NVHQLAGLLPHRGFRDLARKHGPIMHIQLGQISAVIISSPRLAKEVLKTH
DVSFADRPKTFGSELVLYRNTDIALAPYGEYWRQMKKIATLELLSAKKVQ
SFGQIRETELEGFMKFLGLSSGKPVNIHKAITELVNNVVCKASFGKNCKH
QHALLEFLDEFARANSGFYVADLFPDYKFVYVVSGLRSKLMKLHKTLDKI
FDDIFEEHDSRKRDGGEQEEDLLEILLRIKEDGGLEFPITNDNIKAIFVD
IFAGGTDTSSVTIEWAMTEMMRHPTVMKKARAEVREALKGKTKIMESDIQ

GLSYLQSVIKETLRLHPPIPMLLPHVCREHCKVDGYDIPMKMKLIINAFA
CSTDPEYWDDADSFKPERFQDSPVDFMATNYHFIPFGSGRRMCPGINFGL
TTVGFFLAQMLYYFNWELPDGLNAIDVDMTETEGLIAAKKVALTFDSKFL
CSLQLANFPFQGF
>NjCYP71I3
MAIDITIALIFSSFILSLYVILNRNRKGTESLHLPPGPRKLPIIGNLHQI
DLALPHRGFLDLAKKYGPIMSVQIGQVPMIVISSPKLAEEALKTNDLAIA
SRPYLKFADLIMYGGMDVVFGRYGDYWRQMKKLMTTELLSVKKVQSFMGV
RKQEIDGLMDTIRSRCGEPVRLYRMITKVNNTIICKSLFGNNCRQQDDLI
KAMDEVTLLGSSFLIVDLFPKLGFLSGLSGTTTKLKNLHKEIDKMYNEIF
EDRRIKRKTTGALEDDLTDVLFNLKEQGGLRFPITDNNIKAIFTNLITGG
TDTSALTVTWAMTELMRNPRIMKKAQTEVREAFKGERSVIETELHKLVYL
KLIIKETLRLHHTTPLLLPRECREQCQIGGYDIPVKMRIVINAFACGIDP
VSWDDPETFRPERFEKSSHDFYGTNFDYIPFGAGRRMCPGITFGLANVEQ
TFAKLLFHFNWKLPDGMQPKDIDMSETFGGTLTKKEPLEVIPTLHIPY
>NjCYP71J1
MEPLTIVSIAVASFIFFVFWALSPKTSKNLPPGPPKLPIIGNIHQLKSPT
PHRVLRSLAKKYGPIMHLQLGQVSTVVVSTPRLAREIMKTNDISFADRPT
TTTSQIFFYKAQDIGWAPYGEYWRQMKKICTLELLSAKKVRSFSTIREEE
LSRITNVLASKAGTPINFTEMTVEMVNNVICKATLGDCCKDQATLIEVLY
DVLKTLSAFNLASYYPGLQFLNVILGKKAKWLKMQKQLDDILEDVLKEHR
AKGRNKSDQEDLVDVLLRVKDTGGLDFTVTDEHVKAVVLDMLTAGTDTSS
ATLEWAMTELMRNPEMMKRAQSEVRSVVKGNTITETDLQSLHYLKLIVKE
TLRLHAPTPLLVPRECRQDCNVDGYDIPAKTKILVNAWACGTDPDSWKDA
ESFIPERFENCPINYMGADFEFIPFGAGRRICPGLTFGLSMVEYPLANFL
YHFDWKLPNGMKPHELDITEITGISTSLKHHLKIVPIVPKA
>NjCYP71K1
MIPPFFLFHFLIFLLLLLLIKKKWRSIHRPPGPKKLPLIGNLHQLGDSPH
KSLHILSIKHGPIMFLQLGSIPTLVISSPNLAKEIFKTHDLFFSSRPVLY
AAKKLSYNCSNISFAPYGEYWREVRKIAVLELLSLKKVASFGAIRRNEVT
IMIDFIARSLASPINLSELTLSLANNVICRVLFGKKGDDGKFRILDETQA
LLGEVNVADFFPCMWWLNKLNGVERRLEKNFKELDELYNKVIADHLDPDR
PKPDQEDLVDVLLHIQKDQTQGISLANEQIKGILSDMFIAGTDTTAATLV
WIMAELIKNPSSMKKAQEEVRQVANVKGKLLEENEVSKLVYLKSVVKEAL
RLHPPAPLLLPRETTETCIIGNYKVAAKTRVFINAKSIATDPNHWENPNE
FKPERFVNRSFDFEGQSFEMVPFGAGRRGCPGSNFAIQLIEFALANILYW
FDWSLPVGMKTEDVDMEEGVGITVHKKNPLFLVASASCVYEEDELFK
>NjCYP71L1
MGVYIYLPLLLIVPLILIVLKKMKRKKKQLPPGPPKLPIIGNLHQLGSLP
HQSLWKLSQKYGPVMFLKLGGVPTVIISSSESAKEVLKVHDLHCCSRPPL
AGTGKLSYNYLDIAFSPYGDYWREMRKLGSLQLFSTNRVHSFRSIREEEL
AFLVHSFADSASLGCAVDLSRELLTLAANVTCRVAFGKSFRDSGFDGDRF
QDIIHEAMAMLACRSASDFFPSVGWIIDRLTGLHGRLERSFLELDAFYQE
VIDDHLNCSEMNKEEDIIDVLLKIERDQKESDVLQLSKDHIKAVLMNIFL

AGVDTGTITMVWAMAELVRKPSAMKKAQDEIRKCIGDKGKVDESEMEQLE
FLKNVVKETLRLHPPGVLLVPRETISHFKLNGYDIEPKTRMHVNVWGIGR
DPQIWEDPEEFRPERFNDSPIDYKGQHFELLPFGAGRRGCPGIYMGTTLV
ELTLANLLYAFDWKLPDGTKEINMEEGPGQTVYKKLPLSLVAVMAVT
>NjCYP71L2
MALYFLLPLLLMLPLIFLNLIKNMKRKKNKFPPGPPKVPIIGNLHQLGSL
AHQSLWNLSQKYGPVMLMKVGSFPTFIISSSELAKEVVKVHDLDCCSRPV
LVGTGILSYNYLDIAFSPYGEYWREVRKLSVLELLSTKRVMSYRSVREEE
VGFLVDLLAESSSSGCAVDLSKILLTFSMNITCRMAFGKSFRESGFDGDR
FQEAIYEALDMLGSGSASDFFPYVGWIIDRINGYHGRLEKLFKELDGFYQ
EVIDDHINSSKMDKEEEEDIIDVLLKIEMNQKESDVLQLTKDHIKAILMN
IFLAGVGTTMVTLVWALAELVRKPLVMKKAQEEIRNYIGNKGKLVGESEI
HHLKFLKNVVKETLRLHPPAPLISRETLSHFKLNGYDIEPKTRLQVNIWG
IGRDPKTWNEPEEFRPERFNNSSLDYNGQNFEYLPFGAGRRGCPGIYMGA
TTVELTLANLLYSFDWKLPDGIKEIDMEEAAEGLTVRKKVDLSLVPVIYQ
GP
>NjCYP71M1
MLLCLPLLYFLPKIINRSRSCPPGPLGLPFIGNLHQIHHSSLHTSLWNLS
KSYGPILSLNLGFIPAIVVSSATVVKEVLKTQDIIFCNRPSLIGQQKISY
NGLELIFSPYNEYWKEMRKVFMLHLLGPKRVESFRYIREEEVSSTMEKIR
ELALSCNPINLSELMKSVASTIMMRVGFGKRYQDDEHEKYEIPRLLLEVQ
AIFAEFFVSDLWPGLPFVGLVDRLLGKKDRLDKCFQYFDKLYQELIDEHL
SSRGPKSHEKAEDFIDILLRLKEDQIVDLTYDHIKALVMDVLVAGTDTST
ATVVWAMTALIKNPKVMKKAQEEVRNVVGKKGKVDEDDLPKLTYLKAVVK
EIFRLYPPVPLLVVRETTKDAILHGYKIKAKTLVFVNALAIGRDSESWEN
PEEFLPERFLGSNIDFKGNDFELIPFGAGRRICPGISMGVVTVDLLLANL
VYLFDWGLPNGMKEEDIDFDVMPGITMHKKNDLCLLAHV
>NjCYP71N1
MVIIVIALLALPLFTFFLISLMKKNGATRFPPGPRGLPFIGNLHQLDNSF
LQHQLWKLSQKYGPIMSLKLGSVPTIVVSSAKMAKEIMKTHDNIFCTRPS
LFGQQKLSYNGKDVAFSPYNAYWREMKKIVTLRLFSSKRVQSFRPIREEE
VSRMVSQVANSSELVNLSETVMSVTYTIICRLAFSKRFDEGESGNRLGAL
MLECQEMFMSFYFSDYFPLMGWVDKMSGLRARLDKSFREMDSFYQQLIDE
HLDLDKKESKREDIIDIFLQLIKDKSPSIDFTFSHMKALLMNILVGGTDS
SAATVVWTMTELMKNPRVMDIAQKEVRNLIGKKGSVDEEDLQQLPYLTSV
VKETMRLHPPFPLLLPRVAMESCIIDGYNIEAKTKVYINAYAIGRDPESW
NDPEKFLPERFSGNSTDYKGQDFEWIPFGAGRRVCSGISMASTTVELILS
NLLYSFDWKLPIGMNKEDIDTVPLPGLVVHKKTPLCLLAKSK
>NjCYP71N2
MSLKLGFVPTLVVSSARMAKEVMKIHDLEFASRPSLTGQQKLSYNGLDLV
FAPYNDYWREMRKICVLHLFNSKRVQSFRALREDEVKMMIQKIMKCEGKV
VNLSESFMGLTSSIICRVAFGKRYEDEGHERSRFHSLLNEAQAMMGVFFL
CDYFPMMGWVDKLSGLLKRLDKNFREFDDFYQEIIDDHLDTKREKSEQED
ITDVLLKLKSEGLFSTGLTWDHIKAVLMNIFIAGTDTSAATLVWAFTALM

KNPEVMKRVQEETRNLVKKKENSYDNIIIEEDDLQNLPYLKAVVKETMRL
YPAVPLLVPRETVQDCIIDGYRIPSKTLVFVNAWAIGRDPEAWNNPDEFL
PERFLSNSIDFKGQDFELVPFGAGRRGCPGIYMGAVTVELALANLLYSFN
WELDDGMEKEDIDLNVIPGITMHKKNALCLVARNYVHK
>NjCYP72A1
MEISIYYEIAIICTILYVVIQGSRFLNWVWLRPRKLEKYLRKQGLKGNSY
RLLFGDMKEMYLMTKEAKSKPINLSGDVVPRVMPFLHKSFTNYGKYFFTW
LGPKPNIIMDPDLIKEIMTKNNNFQKPKGGNPLGKLLATGLVDYDGDKW
VKHRKIINPAFHLEKLKCMVPAFKLSCSEMVGKWEKLILEKGSSCELDVW
PYLQTMTSDVISRTAFGSSYEEGRRIFELQTEQAELVIQASQTVYIPGSR
FLPTKRNNRMKEIEKQVQISIRGIINKRVKAMEMGETSDDDLLGILLESN
LKEIKQHGNKNYGMSINEVVEECKLFYFAGQETTNNLLVWTMILLSQYPS
WQTRAREELLQVFGNAKPDLEGLNRLKIVTMIINEVLRLFPPGILFTRTI
HEETKIGDIYIPAESIISLPILMVHYDRELWGDDASDFKPERFSEGISKV
TKGQVSYFPFGWGPRICIGQNFAMLEAKMVLAMILQHFSFELSPSYTHAP
HTIVTLQPQHGAHLILRKVESKV
>NjCYP72A2
MEISIYYEIAIICTILYVVIQGFRFLNWVWLRPRKLEKYLRKQGLKGNSY
RLLFGDMKEMYLMTKEAKSKPINLSGDVVPRVMPFLHKSFTNYGKYFFTW
LGPKPNIIMDPDLIKEIMTKNNNFQKPKGGNPLGKLLATGLVDYDGDKW
VKHRKIINPAFHLEKLKVFPQSFFTNYFKFRFLLGLLLICIFDQIQCMVP
AFKLSCSEMVGKWEKLILEKGSSCELDVWPYLQTMTSDVISRTAFGSSYE
DGRRIFELQTEQAELVIQASQTVYIPGSRFLPTKRNNRMKEIEKQVQISI
RGIINKRVKAMEMGETSDDDLLGILLESNLKEIKQHGNKNYGMSINEVVE
ECKLFYFAGQETTNNLLVWTMILLSQYPSWQTRAREELLQVFGNAKPDLE
GLNRLKIVTMIINEVLRLFPPGILFTRTIHEETKIGDIYIPAESIISLPI
LMVHYDRELWGDDASDFKPERFSEGISKVTKGQVSYFPFGWGPRICIGQN
FAMLEAKMVLAMILQHFSFELSPSYTHAPHTIVTLQPQHGAHLILRKVES
KV
>NjCYP72A3
MEITIIYGVAICCIIYVVILGWRVLDWVWLSPRKMEKYLRTQGFKGNPYR
LLVGDVKEMTLMTEQAKLNPINLSDDVVPRLIPFLHKSLHAYGKNFFVWL
GPKPAVHIMDPDVIKEVTTKNFQFEKPKGGNPLVRLLASGLVSYDGQKWA
KHRKIINPAFHQEKLKYMVPAFKLSCGEMIRKWEEKVVEKGSSCEIDVWP
YLQTMTSDVISRTAFGSSYEEGRKIFELQTEQAGLVIQAIQSVYIPGSRF
LPTKRNNRMKEIAKQVEASITGIINKRVESLKAGEASNDDLLGILLESNY
KEIQQHGNKKFGMNIKEVIEECKLFYFAGQETTSSLLVWTMILLSQYPSW
QYRARQEILDVFGNTEPDFDGLNRLKIVNMILYEVLRLYPPAVSLDRTVH
EETKIGDMTLPPGVIISLPILLVHYDKELWGEDAKEFKPERFSVGLSTAT
KGQVCFFPFGWGPRICIGQNFAMLETKMALAMILQNFTFKLSPSYSHAPH
TFLLFQPQHGAHLILEKI
>NjCYP72A4
MEISIFYEIAIICTISYVVIQGLRFLNWVWLRPRKLEKYLRKQGLNGNSY
KFLFGDMKEMYIMTKQAKSKPINLSDDLSPRVLPFVHKSFTNYGKYFFTW

LGPKPNINIMDPDLMKEIFVKSNHFQKPKGGNPLSKLLATGVITYDGDKW
AKHRKIINPAFHHEKLKCMLPAFMVSCSEMVEKWEKMSCEKGSSCEINVW
PYLQTMTSDVISRTAFGSSYEEGRKIFELQIEQAELVIQALQSFYIPGSR
FLPTKRNNRMKEIEKQVQGSIRGIINKRVKAMEVGEASHDDLLGILLESN
FKEMNQNGNKNCGMSINDVIEECKLFYFAGQETTSGLLVWTMILLSRYPS
WQTRAREEVLQVYGNAKPDLDGLNRLKIVTMIINEVLRLYPPAVEVDRTI
YEETKIGDIYIPAGSIFTLPILMVHYDRKIWGEDANDFKPERFSEGISKV
TKGKVSYLPFGWGPRICIGQNFALIEAKIALAMILQHFSFELSPSYAHAP
QKIITLQPQHGAHLILRKV
>NjCYP72A5
MDETVSYNSIAAWCAIAAVLLWWAWRGFYRVWWRPMKLDMFLRNQGLKGT
SYNFLFGDLKMITTLIKQSKSNPISLSHDIVPRVVPWIHQSIEKYGNNFY
LWFGTIPRIHIMEPELVREIFIKYQIFQKPNSNPLVKLLATGLANYEGEK
WAKHRKIINPAFHVEKLKHMVPAFYLSCSELITKWESLVSKNGGSCELDV
WLDLETLTSDAISRTAFGSSYEEGTKIFQLQKQQARLAIQAALSIYIPGW
RFVPTKNNRRMKEIAKEVQLSVQKIIDKKMKAIKEGETGDDDLLSLLLES
NIKEIQQHGNKNVGISIYDVIEECKLFYFAGQETTSVLLVWTMICLSMHQ
EWQVKAREEVLQAFRNEKPNFDGLNHLKIVTMILYEILRLYPPVVALTRK
VIKETKLGEISLPAGVQISVPVIIIHRDYELWGDDANEFKPERFAEGISK
ATKNQAAFLPFSNGPRICVGQNFAMLEAKMALAMILQRFYFELSSSYAHA
PSSFITLQPQHGAHLILHKL
>NjCYP72A6
MMDEKVSYNSIAAWCAIAAVLLWWAWRGFYRVWWRPMKLDMFLRNQGLKG
TSYNFLFGDLKMITTLIKQSKSNPISLSHDIVPRVVPWIHQSIEKYGNNF
YLWFGTIPRIHIMEPELVREIFNKYQIFQKPNSNPLVKLLATGLANYEGE
KWAKHRKIINPAFHVEKLKHMVPAFYLSCSEIITKWESMVSKNGGSCELD
VWLDLETLTSDAISRTAFGSCYEEGTKIFQLQKQQARLAIQAALSIYIPG
WRFVPTKNNRRMKEIAKEVQLSVQKIIDKKMKAIKEGETGDDDLLSLLLE
SNIKEIQQHGNKNVGISINDVIEECKLFYFAGQETTSVLLVWTMICLSMH
QEWQVKAREEVLQAFRNEKPNFDGLNHLKIVTMILYEILRLYPPVVALTR
KVIKETKLGEISLPAGVQISIPVIIIHRDYELWGDDANEFKPERFAEGIS
KATKNQAAFLPFSNGPRICVGQNFAMLEAKMALAMILQRFYFELSSSYAH
APSSSITLQPQHGAHLILHKL
>NjCYP72A7
MEILGKAVIAIVIAVIGRWGWKVLNWLWLKPKKLEKWLRDEGYNGNSYKL
LMGDMVELATMASKSKRDPIPITHDIVPHILPFDHKTISTYGEKSFLWLG
PNPRVYIRDPELIKEILMRPNEFQKPHPEPFRDSIIGGLVITEGHKWSKH
RKIINPAFNLQNLKNMFSVICSSCSDMVHQWELLTMEKGSAEVDVWPYID
NLAGDVISRTAFGGSYEEGRKIFRIQKEQIELVFKMISILYLPGRRFIPT
KANRKYNANITELQSVLRGIINKRKKAIEIGEGSCDDLLGILLESNLKEI
EECGVGLSMEEVIEECKLFYIAGSETTSNLIVWTMVCLSLDQKWQIKARE
EILEVFGTGELDFERLKDLKIVTMILYEVLRLYPPVIMTIRATLKETKLG
NMMIPPGVHLALAMIFIHHDRKVWGEDATEFRPERFSEGVSVATKGKGFF
PFSNGPRICIGQNFAMTEARAAVAMILRRFSFDLSPSYKHSPFPVFTMPP

QFGAKLILRNIS
>NjCYP72A8
MEIILINYAIPISIFCIILYVVIQGFRFLNWVWLRPRQVEKYLRVQGLNG
NSYKLLFGDMKEMLFMIKEAKSKPINLSDDVVARVMPFHHKSVTTYGKSF
FSWLGPNPNINIMDPDLIKEIFIKSNHFQKPKEAHPLGRLLATGIVTYDG
HKWAKHRKIINPAFHHEKLKYMLPAFKLSCNEMIEKWEKMISEKGTSCEM
DVWPYLQTMTSDVLSRTSFGSSYEEGRRILELQTEQAELCIQAVQSFYIP
GSRFLPTKRNNRMMDIEKQVQASIRGIINKRIKAMEEGEASHDDLLGILL
ESNLKEIKQHGNKNDGMSISDVIEECKLFYFAGQETSTGLLVWAMILLSQ
HPSWQIRARDEVLQVFGNANPDLDGLNRLKIVTMIIHEVLRLFPPGVALA
RTIYEETKIGNLSVPAGSILSLPILMVHYDRELWGDDANDFNPERFSEGV
SKATKGQVSYFPFGWGPRICIGQNFAMIEAKLALAMILQHFSFVLSPSYT
HAPHTIVTLQPQHGAYLILQKV
>NjCYP72A9
MELSTEFYVAGVVFAVTLVLITWRILDPLWFHPKKIEGSLRAQGLKGSSY
KFMFGDSKEMAQMTRQAKSKPIDLKDDIGTRVLPFVQKSVAAYGKMCFTW
MGPTPTVQVSEPAIIKEILADYYKFQKSRGHPLFRKLMKGLIDVEGDQWV
KHRKIINPAFHLEKLKQMIPAFYVSCDDMIQKWKELVINESSCEVDVCPY
IEAMSNDVISRTAFGSSFEEGRKIFELLHELLVLVVKSIQSDYFPGSRIW
PTARNRRMNEIDQKVKDSIRVIVDKRLVAMKAGEIGSTDDLLGILLKSNN
EEIKQDGNTKSGLSIEEIIEECKLFYVAGQETTRNVLIWTMVLLGQHTDW
QARARDEVLHIFKDKKPDVEGLSHLKVVNMIFNEVLRLYPPVALLGRKIH
KETKLGDIMLPAGTLVQVNTLLLHYDREIWGDDVDEFKPERFSEGISKVT
KGLTCYLPFGGGPRICIGQNYAILEAKLVLAMILQNFAFEISPSYAHAPH
LLGTIQPQFGTHLILHKL
>NjCYP72A10
MMDTVTNTVAISCAIAVVLCAWQVVNILWVRPKKLEKYLRNQGFGGNKYR
FLYGDMKEFSLMFQESKSKPLSLDDDDGVLTRVVAFTHHSLQKHGANFIT
WAGWKPRVTIMDPELIKEVFAKSNDFKKMEPSPLTKFIATGLVTYEGDQW
AKHRKLINPTFHMEKLKNMVPAFQLSGSEMLGKWEKLVSSKGSCELDIWP
DLQALTSDVISRTAFGSNYEEGLQIFELIKEQSVLVQEAVMSIYIPGSRF
FPTKRNTRMNAIDKKVKHSIRGIIDNKLKAMEAGESNNRDLLGIMLESNM
KEVEEHQNKSHGMTSDEVIEECKLFYFAGQETTSSLLVWTMVLLSKHQEW
QSRAREEVLNVLGDKKMELDALNHLKVVNMIFHEVLRLYPPIVGMYRKVD
KDITLGGFSLPSGTQIELPIMNIHYDEKMWGHDAKKFNPDRFSEGISKAT
KNKVIYFPFGWGPRICVGQNFALIEAKIALAMILQRFSFELSPSYVHAPH
KLLTLQPQYGAHLIMHKL
>NjCYP72C1
MTMTIYDLVGGCLVMGVITYWLYDFLNWVWFRPKRLEKCLREQGFSGNAY
RLFLGDQQESAVMIREAMSKPISLTDDIKQRTIPHILHTIKNHGNNSFMW
VGRIPRLHITEPELVREVLTKYYKFHKNFHNFDPITKHLLCGIGGLEDEL
WVQRRRIVSSAFHFEKLKLMLPAFYMSCSNLVNKWEKIVSAKGGSAEIEV
YHDLESLTGDVISRTLFGSSFEEGKKIFELIKELSVLTIQVIQSIYIPGW
RFMPTKRNNRIKTIDKDIRASIRKIIDNKMKAIKVGESNSSDFLGILLET

NMNEVEQTKTKNNVKMSIDEIVDECKLFYFAGEDSSSALLTWTMVLLSRF
PEWQQRAREEVLQVFGDKKPDYESINSLKIVTMILNETLRLYPPIFELTK
LVHEETKLGDLTLPAGVQVMLPTIIIHHSREIWGDDVDEFKPERFAQGVG
NATKSQGSFFPFSVGPRMCVGQNFALMEAKMAMALILPRFSFELSPSYVH
APYTLITVQPQLGAHLILHKI
>NjCYP72C2
MAMTIYDLVGGCLVMGVITYWLYDFLNWVWFRPKRLEKCLREQGFSGNAY
RLFLGDQQESAVMIREAMSKPISLTDDIKQRTIPHILHTIKNHGNNSFMW
VGRIPRLHITEPELVREVLTKYYKFHKNFHNFDPITKHLLCGIGGLEDEP
WVQRRRIVSSAFHFEKLKLMLPAFYMSCSNLVNKWEKIVSAKGGSAEIEV
YHDLESLTGDVISRTLFGSSFEEGKKIFELIKELTVLTIQVIQSVYIPGW
RFMPTKRNNRIKTIDKDVRASIRKIIDNKTKAIKVGESNSSDFLGILLET
NMNEVEQTKTKNNVKMSIDEIIDECKLFYFAGEDSSSALLTWTMVLLSRF
PEWQQRAREEVLQVFGDKKPDYESINSLKIVTMILNETLRLYPPIFELTK
LVHEETKLGDLTLPAGVQLMLPTIIIHHSREIWGDDVDEFKPERFAQGVG
NATKSQGEFFPFSVGPRMCVGQNFALMEAKMAMALILPRFSFELSPSYVH
APYTLITVQPQFGAHLILHKI
>NjCYP72C3
MSYYRSIIISCVVVFVTYWVYRFVNWIWLRPKKLEKCLREQGFSGNPYRL
LKGDQEESSMLIMEALSKPINLEDDIKQRVIPHVLHTIKNHGVNSFMWDG
RIPRVHITEPELVKEVLVKHTKFRKNLHDNDPIANLFQTGMVTLDGEIWS
SRRRILNTSFHFEKLKLMLPEFYLSCVDMVTKWENKTSKNGSVEVDAHHE
FETLTGDIIARTLFGSNFEECKKIFELIKQLLVLSIEIMRSVYIPGRRFL
PTKRNNRIKEVNKNVISLIMGIINKKMKALKGGEISSASDFLGILLEYNL
NQSENDKNKCDNISIDEIIDECKLFYFAGQETTANLLSWTLVLLSTNPEW
QKRAREEILEVFGDSEPNYDNINRLKIVTMILYEVLRLYPPVGELTKVAS
EDIKLGELFLPAGVQVMLPTIILHHDREIWGEDVNEFKPERFSEGVLKAT
KKQGTYLPFSLGPRVCIGQNYALLEAKMALSLILSRFSFKLSPSYLHAPC
RLIAMQPQFGVPLILRKL
>NjCYP72C4
MSYRSIIISCVVVFVTYWVYRFVNWIWLRPKKLEKCLREQGFSGNPYRLL
KGDQEESSMLIMEALSKPINLEDDIKQRVIPHVLHTIKNHGVNSFMWDGR
IPRVHITEPELVKEVLVKHTKFRKNLHDNDPIANLFQTGMVTLDGEIWSS
RRRILNTSFHFEKLKLMLPEFYLSCVDMVTKWENKTSKNGSVEVDAHHEF
ETLTGDIIARTLFGSNFEECKKIFELIKQLLVLSIEIMRSVYIPGRRFLP
TKRNNRIKEVNKNVISLIMGIINKKMKALKGGEISSASDFLGILLEYNLN
QSENDKNKCDNISIDEIIDECKLFYFAGQETTANLLSWTLVLLSTNPEWQ
KRAREEILEVFGDSEPNYDNINRLKIVTMILYEVLRLYPPVVELTKVASE
DIKLGELFLPAGVQVMLPTIILHHDREIWGEDVNEFKPERFSEGVLKATK
KQGTYLPFSLGPRVCIGQNYALLEAKMALSLILSRFSFKLSPSYLHAPCR
LIAMQPQFGVPLILRKL
>NjCYP72C5
MEMSYSSITISCVVVFLTYWVYRFVNWIWLRPKMLEKCLRKQGFSGNPYR
LLKGDQEESSMLLMEAMSKPINLDDDIKHRVIPHVLQTIKNHGVNSFMWE

GRIPRVHITEPELIKEVLVKHSKFRKNLHDHDPITYLFQTGIPTLEGDLW
SNRRRILNTSFHFEKLKLMLPEFYSSCLDMVTKWENKASENGSIEVDAHH
EFETLTGDVIARTLFGSNFQECKKIFQLIKELLVLTIEVIRSVYIPGRRF
LPTKRNNRMKQVHKEVTGLVMGIINRKMKALKGGEISSASDFLGILLEYN
LNQTQSGKNKSENISYQDIIDECKIFYFAGQDTTSSLLSWTMVLLSMYPE
WQKRAREEILEVFGDSEPNYDSINRLKIVSMILYEVLRLYPPVVELTKVA
TEDIKLGELFLPAGVQVMLPTIILHHDPEIWGEDVKEFKPERFSEGVLKA
TKKQGTYFPFSLGPRVCIGQNYALLEAKMALSLILSRFSFELSPSYRHAP
HTLIIMQPQFGLPLILRKL
>NjCYP72C6
MEMSYSSITISCVVVFLTYWVYRFVNWIWLRPKMLEKCLRKQGFSGNPYR
LLKGDQEESSMLLMEAMSKPINLDDDIKHRVIPHVLQTIKNHGVNSFMWE
GRIPRVHITEPELIKEVLVKHSKFRKNLHDHDPITYLFQTGIPTLEGDLW
SNRRRILNTSFHFEKLKLMLPEFYSSCLDMVTKWENKASENGSIEVDAHH
EFETLTGDVIARTLFGSNFQECKKIFQLIKELLVLTIEVIRSVYIPGRRF
LPTKRNNRMKQVHKEVTGLVMDIINRKMKALKGGETSSASDFLGILLEYN
LNQTQSGKNKSENISYQDIIDECKIFYFAGQDTTSSLLSWTMVLLSMYPE
WQKRAREEILEVFGDSEPNYDSINRLKIVSMILYEVLRLYPPVVELTKVA
TEDIKLGELFLPAGVQVMLPTIILHHDPEIWGEDVKEFKPERFSEGVLKA
TKKQGTYFPFSLGPRVCIGQNYALLEAKMALSLILSRFSFELSPSYRHAP
HTLIIMQPQFGLPLILRKL
>NjCYP73A4
MDLLLLEKALLALFAATIIAITVSKLRGKKFKLPPGPIPVPVFGNWLQVG
DDLNHRNLTDFAKKFGEIFLLRMGQRNLVVVSSPDLAKEVLHTQGVEFGS
RTRNVVFDIFTGKGQDMVFTVYGEHWRKMRRIMTVPFFTNKVVQQYRHGW
EDEMARVVDDVRSNDEASTVGIVLRKRLQLMMYNNMFRIMFDRRFESEED
PLFMKLKALNGERSRLAQSFEYNYGDFIPVLRPFLRGYLKICREVKEKRF
KLFKDYFVDERKKLGSTKSMDNNSLKCAIDHILEAQQKGEISEDNVLYII
ENINVAAIETTLWSVEWGIAELVNHPKIQQKLRQELDTVLGPGVQITEPD
THKLPYLQAVIKETLRLRMAIPLLVPHMNLHDAKLGQHDIPAESKILVNA
WWLANNPAHWKDPEEFRPERFLEEEAKVEANGNDFRYLPFGVGRRSCPGI
ILALPILGITLGRLVQNFELLPPPGQDKLDTSEKGGQFSLHILKHSTIVA
KPRSF
>NjCYP73A5
MDLFLLEKSLLALFAAAVIAIAVSKLRGKRFKLPPGPIPVPIFGNWLQVG
DDLNHRNLADLAKRFGDVFMLRMGQRNLVVVSSPDLAKDVLHTQGVEFGS
RTRNVVFDIFTGKGQDMVFTVYGEHWRKMRRIMTVPFFTNKVVQQYRYGW
EDEAARVVEDVRKMPEAATTGIVLRRRLQLLMYNNMYRIMFDRRFESEED
PLFVKLKALNGERSRLAQSFEYNYGDFIPILRPFLRGYLKLCKEVKDKRL
QLFKDYFVDERKKVGSIKSMDNNSLRCAIDHILEAQEKGEINEDNVLYIV
ENINVAAIETTLWSIEWGIAELVNNQEIQNKLRHELDTVLGPGVQITEPD
TYKLPYLQAVIKETLRLRMAIPLLVPHMNLHDAKLGGFDIPAESKILVNA
WWLANNPSKWKNPEEFRPERFLEEEAKVEANGNDFKYLPFGVGRRSCPGI
ILALPILGITIGRLVQNFELLPPPGQAKIDMTEKGGQFSLHILKHSTIVA

KPRSF
>NjCYP74A1
MESSASHLPFSALQLQLPKHRSLVSSRKPYAGRISVHPIISSLSEKPPVK
LMVAEPAKLPTRRLPGDYGLPLIGPLQDRLDYFYNQGRDDYFKSRIQKHQ
STIFRVNMPPGPFISSNPKVIVLLDGKSFPVLFDLDKVEKKDLFTGTYMP
STELTGGYRVLSYLDPSEPNHGKLKKLMFFMLQSQRHSVIPEFHTSFTEL
FDGLENELASNGKANFNDPNDQASFNFLARSLFGTNPAETNLGLDGPKLI
RKWVLFQLSPLLVLGLPKVIEELLIHTFRLPPFLVKKDYQRLYDFFYSSS
TNLLDEAEKIGISRDEACHNLLFATCFNSFGGMKILFPNMVKWIGRAGVK
LHTELAQEIRSVIRSNGGKVTMAAMEQMPLMKSVVYESFRIEPPVALQYG
RAKKDLVIESHDAVFKVKEGELLFGYQPFATKDPKIFDRPEEFVADRFVG
EEGEKMLKHVLWSNGPETDTPKVGNKQCAGKDFVVLASRLLLVYLFLRYD
SFEIQVAKGPLGASVTLTSLKRASF
>NjCYP74A2
MAISLSSFTLSPLQLHNQLTKSPKILSRRFASAPITASVSERPNIPAPLT
SATSEHAALPTKQLPGDYGLPFVGPIKDRLDYFYNQGKEEFFKSKVEKYK
STVFRTNMPPGPFISSDPKVVALLDGKSFSTLFDVSKVEKKNVFTGTFMP
STDLTGGYRILSYLDPSEPSHAKLKQLVLFLLKSRRDRVIPEFGNSFGEV
FKSLETELKSKGKVSFNGAVEQGSFNFLGRALCGKNPSETELGTDGPTLI
GKWVLIQLAPVLTLGLPKLIEDFLLHTFSLPSFIVKKDYQRLYNFFYESS
TFFLDEAEKTGVAREEACHNLVFATCFNSYGGMKILFPGIIKWVHSAGSE
LQTRLAKEIRSAVKNAGGNVTMAALENMPLTSSVIYETLRIEPPVTAQYG
RAKKDLVIESHDAAFEVKKGEMLFGYQPFATKDARIFENPEEFVGDRFLG
DGEKLLKHVLWSNGPETETPTVGNKQCAGKDFVVLASKLFLTEFFLRFDT
FEADISKSALGPTVTLTSLTKASY
>NjCYP74B1
MSSIIPKITMTVSPGLPSVPLKQSSPAVRTIPGGYGWPLIGPIKDRLDYF
WFQGAESFFRKRIEKHKSTVFRTNVPPTFPFFTGVDPNVIAVLDVKSFSH
LFDMEIVEKKDVLVGDFMPSLSFTGGLRVCAYLDTSESHHTKVKNFAIDI
LKRSSTIWVPTLTSKLDTMWDTLESELTTSGSASYAKPIQQFVFGFFTRC
LIGAEPSASKEIAESGYFMLDKWLALQLLPTVKAGLPQPLEEIFLHSFRY
PFALVKNDYNKLINFIETEGKDVIQRGQSEFQLTKEETLHNLLFILGFNA
FGGFSIFLPALLQNLGSGKPGLQEKLREEVRGKIKLGSGLSFDSVKEMDL
VNSFVYESLRFSPPVPNQFARARKDFQLSSHDSVFEIKKGELLCGFQKLV
MRDPKVFDDPETFVPDRFTKEKGRALLDYLFWSNGPQTGSPSASNKQCPG
KDLVTLTASLFVAHIFMRYDSITCSSGSITAVEKRS
>NjCYP74B2
MMPKIMNTSAPPQPKPAPPLLIRTVPGSYGIPMLGALRDRLDYFWFQGPE
KFYKSRIEKHKSTVFRTNVPPSFPLFTGINPNVIALLDTKSYSHLFDNDL
VDKAETLLGEYRPNVNFAGGVRVCPYLDTYEPQHAKMKGHILDILKQSSD
DWVEILKSKLDKMWSTIESNLSATGSASYRGPLQELVFAFLVRCFTGVDP
TPDLATSAPTTLTVWLALQVAPTIHIGKLQPLEEIFLHSFAYPFFLISRG
YNKVVEFMETYGKETVERGVTEFKLSKKESLHNLTFVMGFNAFGGLSLLF
LALFGRLGSDKTGLQQRMREEARSKGGSTLTFDSVKQMELIHSFVYESLR

FNPTVPTQYGRAKKDFLLTSHDAVYEIKKGELLCGFQPVVMRDPKVYENP
DTFDCERFTKEKGRELLNYLYWSNGPQTGAPTTSNKMCAAKDMVIFVACF
LIADMLRRYDSIILDDSEITKVVKAQVV
>NjCYP74C1
MQSSYEDQSKSLPLKQIPGNYGLPFIGPIKDRRDYFYNQGEDAFFRTRIQ
KYGSTVFRTNMPPGPFISPNSKVVTVLDSVSFQILFDTSKVEKKNVLDGT
YMPSTAFTGGYRTLAFLDPAEPNHKILKSFFLSVIASLHHKLIPTFRNSL
ADFFTNIEDELADKSKADFNALNDIMSFDFVFRLLCNRSPSDTVVGTDGA
KTATSWIALQLVPLMTLGIKYLPNFIEDLFLHTIPLPFFLIKSKYKKLYD
AFYTNATSILDDAEKHGIKRDEACHNLVFLAEFNAFGGMKTLFPALIKWI
GSAGENLHAQLANEVRTVVKAEGGVTLSALNKMPLVKSVVYEALRIEPPV
PYQYGKAKEDLVVNSHEAAYTIKKGEMIFGFQPLATKDPKVFANPEEFVG
DRFVGDGEELLKYVYWSNGRETENPTADDKQCPAKDIVVVLSRVMVVEFF
LRYDSLTVESAKIMLGSAVTITSLTKAK
>NjCYP74C2
MTSSPFNQSMALPENLPLKPIPGDYGIPFFGPIKDRYDYFYNQGEDDFFR
TRIAKHKSTVFRTNMPPGPFISSNSKVIAVLDSKSFPILFDTSKVEKKNL
LDGTYMPSTTFFGGYRVCAFLDPSESTHHALKSLFLSFLASSHNKFIPYL
RSSLSELFTNLETEISDANSADFNTNSDDMAFDFVFRLFTGVHPSETKLK
SKGPGYTNTWLALQLAPLGTLGLKYLPNFIDDIIHTFPLPFFIAKPGYKK
LYNAFNDSATSFLDEAEKQGIKREEACHNLVFLAGFNAFGGMKVLFPSLI
KWIGTAGESLHRRLADEIRIVVKQEGDVTFSALEKMPLMKSAVYEALRIQ
PPVPYQYATAKEDLVVESHDGAFQIKKGETIFGFQPIATKDPEVFENPEE
FIADRFVGDGEELLKYVYWSNGRETESPTADNKQCPAKDLVVLCSRIMLV
EFFLRYDTFTVEIGKVALGASVKITSFTKAT
>NjCYP75A1
MEPQSWSIILLALFLATTLFLLRHRPKKNFPPGPKAWPIIGNIHQIGPLP
HHSFHSLSETYGPLLHLHLGSRPVVVASSPHMAEQILKTHDQTFASRPAL
ASAKYTSYNSSNVLWAPYGPHWRQARKIYSTELLSPKKLESYDYIIEEEN
ILFIRRLYKTRGESIKVKEELTRYMLSNLSRVVMGNEYFGSIMKLEELGE
ILDEWFLLNGVINLGDWIPWLGFLDLQGYVKRMKSLHKKFDMFNNSVLSY
HKNKVNEKEDMVDVLLKLANQPNLEVKLTNDGVKGLVQDLLVGGTDTSAT
TVEWAILELLKNPHILKKAIKELEEVIGNQRWVQEKDFGKLPYIESIIKE
TLRLHPLGTLLAPHFAIQDCKIDGYDILKGTTVFINTWSIGRNPLYWDNP
LEFNPDRFSGKYSNIDVKGLNFELLPFGSGRRMCPGYRLALNVVRSTLAN
LLHGFIWKLPNDIKVEDICMDEVYGIASHPKMSLDLVLEPRLPSTLYY
>NjCYP75A2
MEPQSWSIILLALFLATTLFLLRHRPKKNFPPGPKAWPIIGNIHQIGPLP
HHSFHSLSETYGPLLHLHLGSRPVVVASSPHMAEQILKTHDQTFASRPAL
ASAKYTSYNSSNVLWAPYGPHWRQARKIYSTELLSPKKLESYDYIIEEEN
ILFIRRLYKTRGESIKVKEELTRYMLSNLSRVVMGNEYFGSIMKLEELGE
ILDEWFLLNGVINLGDWIPWLGFLDLQGYVKRMKSLHKKFDMFNNSVLSY
HKNKVNEKEDMVDVLLKLANQPNLEVKLTNDGVKGLVQDLLVGGTDTSAT
TVEWAILELLKNPHILKKAIKELEEVIGNQRWVQEKDFGKLPYIESIIKE

TLRLHPLGTLLAPHFAIQDCKIDGYDILKGTTVFINTWSIGRNPLYWDNP
LEFNPDRFSGKYSNIDVKGLNFELLPFGSGRRMCPGYRLALNVVRSTLAN
LLHGFIWKLPNDIKIEDICMDEVYGIASHPKMSLDLILEPRLPLSLYY
>NjCYP75A4
MEPQSWSIILLALFLATTLFLLRHRPKKNFPPGPKAWPIIGNIHQIGPLP
HHSFHSLSETYGPLLHLRLGSRPVVVASSPHMAEQILKTHDQTFASRPAL
ASAKYTSYNSSNVLWAPYGPHWRQARKIYSTELLSPKKLESYDYIIEEEN
ILFIRRLYKTRGESIKVKEELTRYMLSNLSRVVMGNEYFGSIMKIEELGE
ILDEWFLLNGVINLGDWIPWLGFLDLQGYVKRMKSLHKKFDMFNNSVLSY
HKNKVNEKEDMVDVLLKLANQPNLEVKLTNDGVKGLVQDLLVGGTDTSAT
TVEWAILELLKNPDILKKAIKELEEVIGNQRWVQEKDFGKLPYIESIIKE
TLRLHPLGTLLAPHFAIQDCKIDGYDILKGTTVFINTWSIGRNPLYWDNP
LEFNPDRFSGKYSNIDVKGLNFELLPFGSGRRMCPGYRLALNVVRSTLAN
LLHGFIWKLPNDIKVEDICMDEVYGIASHPKMSLDLVLEPRLPSTLYY
>NjCYP75A5
MEPQSWSIILLALFLATTLFHLHHRHRPKKNFPPGPKAWPIIGNIHQIGP
FPHHSFHTLSETYGPLLHLHLGSRPVVVASSPHMAEQILKTHDQTFASRP
ALAIGKYTSYNSSNVLWAPYGPHWLQARKIYSTEIFSPKRLESYGYILEE
ENILFIRRLYKTRGESIKVKEELTRYMLSNLSRMVMGNEYFGSIMKIEEL
GEILDEWFLLNGVINLGDWIPWLGFLDLQGYVKRMKSLHKKFDMFNNSVL
SYHKKSNEKEDMVDMLLKLANHPNLEVKLTNDGVKGLVQDLLAGGTDTSA
TTVEWAMLELLKNPHILKKAIKEVEEVIGNQRWVEEKDFGELPYIESIIK
ETLRLHPLATLLAPHFAIQDCNIDGYDILKGTTVFINTWSIGRNPLYWDN
PLEFNPDRFSGKYSNIDVKGLNFELLPFGSGRRMCPAYRLALNVVRSTLA
NLLHGFIWKLPNDIKVEDICMDEVYGLASHPKMSLDLVLEPRLPLSLYY
>NjCYP75A6
MESPAPTWSPYAAAWFAILALFLLSRHLRRRKMNPPPGPKAWPIIGNLNL
MGSLPHRSVHDLSLKYGPIMQLRFGSFPVVVGSSVEMAKIFLKTMDLTFV
DRPKTAAGKYTTYNYSDITWSPYGPYWRQARKMCVLELFSSKRLESYEYI
RVEEMNSLIKELYASKGKVITLKDHLSTLSLNVISRMVLGKRYLDESENS
IVGPEEFKKMLDELFLLNGVLNIGDSIPWIDFMDLQGYIKRMKVVSKKFD
KFLEHVLDEHNQKRRSVENYVANDMVDVLLQLADDPDLEIKLERHGVKAF
TQDLLAGGTESSAVTVEWAISELLKKPEIFEKAREELDRVIGKKRWVQEH
DMPNLPYIDAICKETMRLHPVAPFLVPRMAREDCKIGDYDIAKGSRVLVN
VWTIGRDPKIWEQPNEFIPDRFIGKEIDVKGHDFELLPFGSGRRMCPGYS
LGLKVIQSSLANLVHGFKWKLPNTMKKEDLDMNEIFGLSTPKKYTLEAVV
EPRLPLDLYFSM
>NjCYP75A7
MESLSYTSYAAAWLATAVLLLLARRLRRPRNLNPPPGPKPWPVIGNLNLM
GTLPHRSIHDLSQKYGEIMQLKFGSHNVVVGSSAEMAKVFLKTMDVNFAC
RPKTAAGKYTTYNYSDITWSPYGSYWRQARKMCLMELFSVKRLESYEYIR
VEETNSLLKSIFESAGEEILLKDLLSTVSLNVISRMVLGKSYLDESIVSP
DEFKKMLDELFLLNGVFNIGDSIPWIDFMDLQGYVKRMKTVSKKFDKFLE
HVLDEHNERRKAGGEKFEAKDMVDLLLQLADDPNLDVKLERHGVKAFTQD

LLAGGTESSAVTVEWAIAELLKKPEIFHKAIEELDRVIGKGKWVNEKDMP
NLPYIEAIAKETMRLHPVAPMLVPRRARENCKVAGYDITEGTRVLVSVWS
IGRDPNLWENPEEFWPERFIGKEIGVKGHDFELLPFGAGRRMCPGYSLGL
KVIESTLANLLHGFDWKLPGTMSEKDLNMEEIFGLSTPKKFPLLTVAQPR
LPVEMYRF
>NjCYP75B1
MNTSISIFLYTFLAAILLYCLFNLRTLLLNRHPKPLPPGPKPWPIVGNLP
HLGAKPHQSLAALARTHGPLMHLRLGFVDVVVAASASVAAQFLKTHDANF
SSRPPNSGAKHIAYNYQDLVFAPYGARWRMLRKICSVHLFSAKALDDFRH
VRQEEVAIMSKALVNAGNRAVNLGLLLNVCTTNALGRVMLGRRVFGGEDA
KAEEFKDMVVEMMVLAGVFNLGDFIPALEWLDLQGVASKMKKLHARFDAF
LTAILEEHSSGKVSGGNYVDFLSILISLKQDSGGDGEKLTDTEIKALLLN
LFSAGTDTSSSTVEWAIAELLRHPKILSRAKEELDAVVGSSRLVADTDLS
QLTYLQAIIKETFRLHPSTPLSLPRMAAESCEVNGYFIPKGSTLLVNVWA
IARDPESWSDPLEFRPERFLPGGEKPNADVRGNDFEVIPFGAGRRICAGM
SLGLRTVQLLTASLIHAFDWELPEGELVEKLSMEEAYGLTLQRATPLMVH
PKPRLTPQVFNS
>NjCYP76A25
MELSCLCSVNILSVSLLVALLFVALKLVTGKNSIPAGKKLPPGPPGWPVV
GNMFQLGTLPQKTLYDLGQKYGPVLWMQLGSVNTMVVQTAAAASVLFKEH
DVVFSDRKQKEGLNAFNFNDGSLGQARFGADTNWRVLRRLCSSEFMVNKR
INDTTDIRRRLEDSLVRWIEEEARAGEVEVGRLFFLVAFNVIGNLLLSRD
LMDASDPESKVFFKCMNKIVELAGTPNISDFLPWLKFLDPSGVKRGIHKH
MEKTLKVTSRFVQERLEQRKRADDHKRKKDFLDTLLEYEGDGKGGPDKMT
LHNAQILIMEMFFAGSETTSISIEWAFTEMMRNPHVLKRAQDEIDTVVGL
DRKVEESDFDKLPYLQAIVKEALRLHPALPLGLPRLTTADTEYMGYFVPK
DTQVFVNSWGIGRDPDAWEDPLTFKPERFLNSDIEYKGQHFSLIPFGSGR
RICVGFPLAHRIVHLTLSTIIQNFDWELPAGITPKTIDMEEQFGLTLRKK
TPFKAVPKKAK
>NjCYP76A26
MELSCLCSVNILSVSLLVALLFVALKLVTGKNSIPAGKKLPPGPPGWPVV
GNMFQLGTLPQKTLYDLGQKYGPVLWMQLGSVNTMVVQTAAAASVLFKEH
DLVFSDRKQKEGLNAFNFNDGSLGQARFGADTNWRVLRRLCSSEFMVNKR
INDTTDIRRRLEDSLVRWIEEEARAGEVEVGRLFFLVAFNVIGNLLLSRD
LMDASDPESKVFFKCMNKIVELAGTPNISDFLPWLKFLDPSGVKRGIHKH
MEKTLKVTSRFVQERLEQRKRADDHKRKKDFLDTLLEYEGDGKGGPDKMT
LHNAQILIMEMFFAGSETTSISIEWAFTEMMRNPHVLKRAQDEIDTVVGL
DRKVEESDFHNLPYLQAIVKEALRLHPALPLGLPRLTTADTEYMGYFVPK
DTQVFVNSWGIGRDPDAWEDPLTFKPERFLNSDIEYKGQHFSLIPFGSGR
RICVGFPLAHRIVHLTLSTIIQNFDWELPAGITPKTIDMEEQFGLTLRKK
TPFKAVPKKRSNLKF
>NjCYP76B1
MEFLVIALGLFLSYFLTRASLSVFGVGKPKNLPPGPTQLPIIGNLHLLGD
QPHQSLAKLASIHGPIMFLKLGRISVLVISSAAAAKEVLQKQDLAFSSRY

IPDALTSHNHSHYSVVWLPVATQWRSLRRFLNINIFSNNSLDAKQHLRRQ
KVEELVAYCRKACQSGEPVNISRAAFRTTLNLLSNTFFSKDLTDPYEDSG
KEFKELVGDIMVEAGKPNLVDFFPVLRKIDPQGIRKRLTRYFGKVFEIFE
ALIEERLGINGSKHDDVLDECLKFSEKNPDEINRTHINSLLLDLFAAGTD
TTSNSLEWAMTEVLRNPHTMTKAKQELEDVIGKGKMVEESDISRLPYLGC
IVKETLRIHPPVPFLIPRKNVNEVKLNGYNVPKGTQVFVNAWAIGRDSTI
WEDSMKFMPERFLTSSLDVHGRDFELVPFGAGRRICPGLPLAARMLPVML
GSLLNNFNWNLDGGLRLDELDMDEKFGITLQKATPLCVVPFPMD
>NjCYP76B2
MEFVAIVVCLSLSYALIRATFSLFGVGKPKNLPPGPRPLPIIGNLHLIGD
QPQQSLTKLAKIHGPIMFLKLGRISALVISSAAAAKEVLQKQDLAFSTRF
VPDGLHAHNHSLNSVVWIPVGHQWRTLRRILTTNIFAGNCLDANQHLRRQ
KIQELVSFCRKASDSNESVDIGRAAFRTSLNLLSNTIFSKDLTDPFEDSG
REFKEVVGNIMMEVGKPNLVDYFPVLKKIDPQGIRRRLTHHIGKVLAIFE
ELIKERLMMKRSNQEDVLDVCLKISEDNPEELNREVLKSMFLDLFVAGTD
TTSSTLEWAMTEVIRNPHTIKKSKVELEECIGKGAIVEENDLIRLPYLVS
IVKETLRIHPPIPFLVPRKVDNEIKISGYTIPKGTQVLVNAWAIGRDDSI
WEDSLAFKPERFMTTEIDVRGQDFELIPFGAGRRICPGLPLATRMIPIML
GSLLNNFDWVLDTCKYGELNMTERFGITIQKADPLCVVPISLE
>NjCYP76B5
MDFLTIALSLLFVFTLYQAFLSLTNSARNKKLPPGPTPLPIIGSLHKLGD
QPHQSLAQLAKIHGPVMSLKLGSITTVVFSSSEAAKEVLQKKDISFANRH
VPDALHAHNQFKFSVVWLPVAPQWRSLRKILNSNIFSGSRLDANQHLRNR
KVEELITYCRKNSQSGDAIDIGRAAFRTSLNLLSNTIFSKDLTDPYQDSA
KEFKELVGNVMVEAGKPNLVDFFPVLKTIDPQGIRRRMTIHFGKVIDLFD
GLINERLESKKSRDAEVKNDVLDVCLGISEENPEEIDRTHIERMCLDLFV
AGTDTTSSTLEWAMAEVLRNPNTMVKAKAELEEVIGKGKLLAESDVSRLP
YQRCIVKETLRIHPPVPFLIPRKVDTDVEACGYIVPKNSQVFVNVWAIGR
DPDLWVDPLSFKPERFMESELDVRGRDFELLPFGAGRRICPGLPLAIRMV
PVMLGSLLNSFGNSMEGLRPKSWIWERNLGLLYKKHNRFEQFRVRFLCKI
>NjCYP76B6
MDFLTIALSLLFVFTLYQAFLSLTNSARNKKLPPGPTPLPIIGSLHKLGD
QPHQSLAQLAKIHGPVMSLKLGSITTVVFSSSEAAKEVLQKKDISFANRH
VPDALHAHNQFKFSVVWLPVAPQWRSLRKILNSNIFSGSRLDANQHLRNR
KVEELITYCRKNSQSGDAIDIGRAAFRTSLNLLSNTIFSKDLTDPYQDSA
KEFKELVGNVMVEAGKPNLVDFFPVLKTIDPQGIRRRMTIHFGKVIDLFD
GLINERLESKKSRDAEVKNDVLDVCLGISEENPEEIDRTHIERMCLDLFV
AGTDTTSSTLEWAMAEVLRNPNTMVKAKAELEEVIGKGKLLAESDVSRLP
YLRCIVKETLRIHPPVPFLIPRKVDTDVEACGYIVPKNSQVFVNVWAIGR
DPDLWVDPLSFKPERFMESELDVRGRDFELLPFGAGRRICPGLPLAIRMV
PVMLGSLLNSFNWKLDGGIAPKELDMGEKFGITLQKAQPLRAIPSPLSV
>NjCYP76C1
MEWVSESNFIFWSLIIITPPLLLHFLRRGPPNHRPPGPRGWPVFGYMFNL
GIMPHRTLFALKQKYGDVVWLKLGSKNTIVILTAKFAADFFKNHDVSFAD

RLITHTTRSHDFHKSSISVAPYGAYWRVLRRLCTVEMFTSRRINETAPAR
EECARELTSWIEQEAHEVGEARGIHVSRLVFLAAFNMIGNLVLSRSVVGY
GSKVGLDFFKAMDGIAEWAGCPNVSDLFPWLSVLDLQGLRKKMDRDKGKA
LAIASGFVKQRIEEREKKVEIRNKDLLDVLLDFEGTGKNEPAKLSEKNVD
VFILEMFMAGSETTSSTVEWVMTELLCNPEAMIKVKAELNKICTPKRKFE
ESYIENLPYLQAVIKETLRLHPPLPFLVPRRATQDVNFMGYHIPKNTQVF
VNTWAIGRDSEYWDDSLLFNPDRFLGSKVDFKGQHFEFIPFGAGRRMCPG
IPLAHRMVPLVLGSLLLEFDWEFDDRVTRETIDMREKMGMTMKKLVPLKA
SPKRVRT
>NjCYP76C2
MDFPSSTTFLLCSILTIISSIFLLRRQRQKTTRLPPGPPAWPVFGHTFNL
GSMPHRTLAGLKQQYGPVIWLKLGSINTMAILSAKAAGELFKNHDLAFAN
RSITETMKSNDYHKGSLALAPYGPYWRVLRRICTVEMVVAKRINETVSVR
RRCIDNMLTWIEKEASEERVVHVARFVFLASFNMLGNLMLSRDLVDPESR
VGSEFFDSMMSLMEWTGHPNVSDLFPWLSWLDVQGLRKKMDRDMGKALGI
ASGFVKERVKERGFGGERKMDFLEVLLEFEGIGKDEPEKLAERDINILIL
EIFQAGTETTSSTTEWAMTELLCNPEKMTKVKTELSSIIGHNRKFEESDI
ENLPYLHAVIQETLRLHPPIPFLVPRKAIQDINFMGYHIPKNTQLFVNVW
ALGRDEECWDDPLSFKPERFLGSKVDYKGQYFEFIPFGGGRRMCAGVPLG
HRMLHIVLGSLLHTFDWKLENHVSPETMDMRDKMGVTVRKLEPLKAIATK
LYGI
>NjCYP76G1
MESQILGFTLALLLWLAWAIKRLSRQSRRYTEEQLQLPPGPRPWPIVGNI
FQLGWAPHVSFAKLSAQHGPIMTLWLGSMCTVVISSSQASREMFKRHDAV
LAGRKIYESMKGNIGNEGSLITAQYGPHWRMLRRLCTTEFFTATRLECTY
EVRARCVDNMVRFIEEAGGSGTKEIDLGRFFFLMAFNLIGNLMFSRDLLD
PKSESGAKFFYHAGKVMEFAGKPNVADFVPVLSRVDPHGIRRKTQFHVKR
AFDIAGEFIKERMESIGNGEEDGKKDYLEVLLKYRGDGVEGPYKFSSTII
NTIVFEMFTAGTDTTTSTLEWAMAELLHNPKTLQKVQSELRTILSPNKKL
KENDIDNLPYLDAVIKETLRLHPPLPFLVPHKAMDTCQMLGYHIPKETQI
LVNVWAIGRDPKNWENPLEFKPERFMDPNGVDYKGHHFEFIPFGSGRRMC
PAVPLATKVLPLALGSILLKFDWVLGGVICLSKMDMSEKMGITLRKRVPL
KAIPIPYNG
>NjCYP76T1
MEMISFIISFSLFSIFAYILSLINRRKLPKPHESLAKLAKKHGPLMTIRL
GSVTSVVATSAEMAREILQKNDEACSGRLVPDAVTALRDNHLAVLWISAG
EEWRIIRRALNTFLTHHQTLDTLTRLRHVAVEEMVVHVKACSEKNLAMDI
GKLAFATALNQMSSTCISRNVDDVEGFLSAVKTLMVVDGKFNIADIFPWL
KPFDPMSIRKRAKAAYGWFDEIIEGFIDRRLKQRESEQMRYGDLLDSLLD
YCDENESLFNLKHIKVLLVDLFLAGTETISNTTEWAMTELMLHPHIMERV
RQEVSGSIKMKGNIEEVDILELPYLQAVVKETMRLHLAVPLLVPHKTEID
VKLSGYIIPKNTQVLINAWAIARDAEYWDNPTSFMPERFINNFDSDFKGQ
NFTFLPFGSGRRMCPGISLAQRVVSLMIASLVYYFDWKLPNGTEALDMND
TFGLTLQRSTPLLVVPTTRNKTNNM

>NjCYP76T2
MEMISFIISFSLFSIFAYILSLINRRKLPPGPIGLPIVGNLFDIGPKPHE
SLAKLAKKHGPLMTIRLGSVTSVVATSAEMAREILQKNDEACSGRLVPDA
VTALRDNHLAVLWISAGEEWRIIRRALNTFLTHHQTLDTLTRLRHVAVEE
MVVHVKACSEKNLAVDIGKLAFATALNQMSSTCISRNVDDVEGFLSAVKT
LMVVDGKFNIADIFPWLKPFDPMSIRKRAKAAYGWFDEIIEGFIDRRLKQ
RESEQMRYGDLLDSLLDYSDENESLFNLKHIKVLLVDLFLAGTETTSNTT
EWAMTELMLHPHIMERVRQEVSGSIKMKGNIEEVDILELPYLQAVVKETM
RLHLAVPLLVPHKTEIDVKLSGYIIPKNTQVLINAWAIARDAEYWDNPTS
FMPERFINNFDSDFKGQNFTFLPFGSGRRMCPGISLAQRVVSLMIASLVY
YFDWKLPNGTEALDMNDTFGLTLQRSTPLLVVPTTRNKTNNM
>NjCYP76T3
MEFLLCFLLPTTCLIIFLHFVSLFHRRKLPPGPLGLPIFKNLFDIGPKPH
ESLANLAKKFGPLITITLGSMTTIVISSPKMAQEVLQKKDEIFSGRTVPD
SMALLDSHTPNAVAWISTGELWRTVRKVLSIFLTSQQKLDSLCDLRHRVV
QEMVQHVKKVGEKSECVDIGKLAFTTALNQMSNTCFSVNVADYETRDFFI
GFHKAVKTIMEIDARFNIGDYFPLLKWFDLQGIRRENKKAYGFLDGLCDK
FIAQRLKDRESKMERFGDLLDCFLDFSQENESQFNLRQIHVLLVELFMAG
TETSSITTEWAMSELMLHPQKMEKLRQEITESVSANGKVDETAIIRLPYL
QAVVKETMRLHLAVPLLLPHKTEKQVELFGYEIPKNTQVLVNAWAIARDP
IFWENPEVFMPERFLDSEIESKGQHFTYLPFGSGRRMCPGIYLAERVVSL
MIASLVYNFEWKLPNGNTPEKLDMNDKFGLTLQRTTPLMIVPTALEI
>NjCYP76T4
MEFLLSFLLPTTCLIIFLHFVSLFHRRKLPPGPLGLPIFKNLFDIGPKPH
ESLANLAKKFGPLITITLGSMTTIVISSPKMAQEVLQKKDEIFSGRTVPD
SMALLDSHTPNAVAWISTGELWRTVRKVLSIFLTSQQKLDSLCDLRHRVV
QEMVQHVKKVGKKSECVDIGKLAFTTALNQMSNTCFSVNVADYETRDFFI
GFHKAVKTIMEIDARFNIGDYFPLLKWFDLQGIRRENKKAYGFLDGLCDK
FIAQRLKDRESKMERFGDLLDCFLDFSQENESQFNLRQIHVLLVELFMAG
TETSSITTEWAMSELMLHPQKMEKLRQEITESVSANGKVDETAIIRLPYL
QAVVKETMRLHLAVPLLLPHKTEKQVELFGYEIPKNTQVRVNAWAIARDP
IFWENPVVFMPERFLDSEIESKGQHFTYLPFGSGRRMCPGIYLAERVVSL
MIASLVYNFEWKLPNGNTPEKKKKKKKKK
>NjCYP76T6
MELLFLFLCATIFFFFLLHFINLHRRKRLPPGPTGLPIIGNLLDIGPKPH
ESLAKLSKKHGPLMTIQLGSITTVVVSTPDAAREILQRNDQACSGRIIPD
AVTALHNHDAAVIWIPANEEWRTIRKALKTYLTHQHKLDTVRDLRENVME
EMLDFLQESGRKKAVVDIGKLAFAVALNQMSNTCLSRNLTTYESDDIGGF
KAVVKKLMEVDGKFNIADIFPVLKPFDPQNIRREAKAAYDWLDKVTESFI
QERLKHRESKMARFGDTLDSLLDYSEDSEADFNLIHIRILLVDIFIAGTE
TNSSTTEWVMTELLLNPAKFSKLREEISTIVGEEGKIQEAKILNLPYLQA
VIKETLRLHLPVPLLVPHKTETEVKLGKYMIPKNTRILVNAWSIARDPRY
WEKPLQFIPERFLGNSVDYKGQHFEFIPFGSGRRICPGIPLAHRVVSLTV
ASFVYHFDWMLPHATEEMDMNNVFGLTLLRAT

>NjCYP77A8
MDSLSSYYHIIFTTLAFFVSAIVFLLSRKSKSKRLNLPPVVPGWPIVGNL
FQVAKSGKHFFLYTRDLLPKYGPIFTLKLGSTTMIVVSNAELAHEALIEK
GQIFASRPPEHATRTVFSCNKFTVNAALYGSVWRSLRKNMVQNMLSSTRL
REFRNIREVAMDRFISRLSAEAEANNGAVWVLKNARFAVFCILLAMCFGV
EMDEEMIEKMDNMMKTVLITTDPRLDDFLPLLRPFFSKQRKRAMEVRKQQ
IETLVPLIEKRRLALENLGSDNKASSFAYLDTLFDLKVENRKSAPTNAEI
VSLCSEFLNGGTDTTGTAIEWAIARFIDNPDIQRKLYEEIKTSVGDRKVD
EKDVEQMPYLNAVVKELLRKHPPTYFSLSHAVVEPAKLGGYDIPPGVNVD
FYLPGIHEDPTIWSEPEKFDPDRFFTGREEADITGMRGVKMIPFGMGRRI
CPGLGMATVHVNLMIARMVQEFEWVAYPENSKVDFTEEVEFTIVMKNTLR
ATIKPRA
>NjCYP77A9
MDSLSPYYHIIFTALAFFLSALIFFFSRKSMSKKVHIPPGPPGWPVVGNL
FQVARSGKPFFLYVRELIPIYGPILTLRMGNRTLIIISSAELAYEALIEK
GQIFATRPRENPTRNIFSCNKFTVNAALYGPVWRSLRRNMVQNMLSSTRL
REFRNVRDAAMNKLVARLEAEAAANDGAVWVLRNARFAVFCILLAMCFGI
DMDDEELIDAVDQMMKTILITLDPRIDDFLPILSPFFSKQQKRANVVRAE
QIKLLVPLIDKRRSVLRNPTSHPKAASFSYIDTLFDLKIEGRKSSPSDAE
IVTLCSEFLNGGTDTTGTALEWAIGRLIENPDIQSRLYDEIRSTVGEKKV
DEKDVEKMPYLSAVVKELLRKHPPTYFSLTHAVLEPAKLAGYDIPTDANV
EFFLPGISDDPKIWSDPENFDPDRFFAGREDADITGVTGVKMMPFGIGRR
ICPGLGMATVHVNLILARMVQEFQWIGYPEDNRVDFSEKLEFTVVMKNTL
RAKIKKRG
>NjCYP77B1
MLFLAALFLLLWWRFWSFTGGGSKNLPPGPPGWPIVGNLVQVILQKRPFM
YVVRDLRKQYGPIFTMQMGQRTLVIVTSSELIHEALVQRGPTFASRPPDS
PIRLMFSVGKCAINSAEYGPLWRTLRRNFVTELINPVRIRQCSWIRKWAL
ETHIKRLQDEASQNGFVEVMTNLRLTVCSILICLCFGAKISEDRIKNIES
ILKDVMMITMPKLPDFLPVLLPLFSRQVKEAKELRKRQMECLVPLVRSRR
AFVENGENPSLEMVSPIGAAYIDSLFGLEPVGRGRLGEEELVTLVSEVIN
AGTDTSATTVEWALLHLVMNQEIQEKLYNEIVDCVGKNGIITENDVESMP
YLGAVVKETFRRHPPSHFVLSHAATEETELGGYTIPAKVNVEFYTAWLTE
DPSLWEDPAEFRPERFLDGDGVDVDVTGTRGVKMMPFGAGRRICPAATLG
TLHVNMLLARMVHEFKWVPVPDSPPDPTETFAFTVVMQNPLKAVILPRQR
IKD
>NjCYP78A3
MTTDIESLWMFVLASKCKSYTSIYPLLVALASAWLVMSLIYWAQPGGPAW
GKLNKNKPIPGPRGFPIIGSINLMSGLAHRKIAAAAQAIPGAKRLMAFSL
GETRAVVTCNPDVAKEILTSSVFVDRPVKESAYSLMFNRAIGFAPYGVYW
RTLRRISAAHLFCPKQIKASEGHRMDISHQMVEMFRAKDNLRVRDALKRA
SLNSMMCSVFGRNYGLDSEKGEIEEVKELVDEGYELLSILNWSDHLPWLL
DFDPQKIRFRCSNLVPRVNRFVGRIIAEHRAQTDRLDRDFVDVLLSLQGA
DKLDNSDMIAVLWEMIFRGTDTVAVLIEWVLARMVLHPEAQSMVQDEIDR

VVGRARGVAESDVTELVYLTAVMKEVLRLHPPGPLLSWSRLSITDTKVDG
YDVPAGTTAMVNMWAITRDPQVWADPLKFNPQRFVVESEATPEFSVLGSD
LRLAPFGSGRRSCPGKTLGLTTATFWVASLLQELEFRACDPNPVDLSEVL
RLSCEMANPLMVKVQPRIRPKSMSP
>NjCYP78A4
MKIFVWANFFLLTPCYVVAFYNLSLPLLFFLSLLSLFPFIANVWLVPGGF
AWRNLNLNPSKIPGPVGWPFVGILPKMGSHAHRKLASMASSMGATRLMAF
SLGSVRVVITSHPETAKELLCGSAFSDRPVKESASLLMFERAIGFAPSGT
YWRHLRKIVAQHMFSPKRVLSLECLRQHVCDEMIEKVLVEMNDKKIVELR
GIFQKSSLMNVMESVFGSGLGLEKEEELGFMVKEGYQLIGEFQWGDYFPI
RVLDFTGVRRRCHKLTLKVKRLVGQIVEERRRESGGINYTGKNDFLSVML
SLPKEDQLSDADMVAVLWEMIFRGVDTVAILLEWIMARMVLHQDIQAKAQ
EEIEEHVGNQRPPQDSDIPNLVYVQAIVKEVLRLHPPGPLLSWARLATRD
VQLGKFFVPAGTTAMVNMWAITHDSSIWKNPWDFNPERFMEEDFPIMGSD
LRLAPFGSGRRVCPGKSLGLATIHLWLARLLQQYKWLPLPSQKVDLSECL
KLSLELQTPLACRAIVR
>NjCYP78A5
MKSFMFSIITLFFFVLSFIATNYQNSCLLLSLMIPFLSLLPFFFNLCLIQ
GGFAWRTYHSSSKNTKKLCGPMGFPILGILPQMGSHAHKKLAAMAHSHGS
TRLMAFSLGSTRAVVSSDPDTAKEILCGASFSDRPIKDSARLLLFGRAIG
FAPSGDYWRHLRRIAANYMFAPRRISGLEGVRQRVANEVIIDGVVKEMKE
KGVVEVRGILQKASLKNVLESVFGCGLGLEEEEELGFMVKEGYELLWQFN
WGDYFGLGFLDFYGVKRRCFKLACKVKCVVGKIIEERRRRRKLGDDIDEL
ISGDFLTVLLSLPKDDQLSDEDMVAVLWEMIFRGTDTVAILLEWIMARMV
LHQDIQSKAREEIDTCVGGKRHVQDSDIPNLPFLQSIVKEVLRLHPPGPL
LSWSRLAIQDVHVDKFFIPAGTTAMVNMWAITHDPSIWNDPWAFKPERFM
DQDFSLMGSDLRLAPFGAGRRVCPGKALGLVTVQLWLARLLQQFKWLPAK
PVDLSECLKLSLEMKKPLACRVISRAHELQFGNIN
>NjCYP79A1
MESNLFTSLNFSTLENTLLCSTMLLLAFVFFVVKSTQKSPPLPPSPKQWP
IIGSLNALRKNKPVFRWIHKLMEEMKTNIICIRFFRTHVIAVSTDYLAKE
FLKKQDVVFSSRPICMSAEITSSGYLTVVMGPMGDQWKKMRRLLSSDIVS
STKHRWLKNKRDEEADHLVRYVYNKTGLTGGNVNVRVVAQHYCGNVIRKL
IFSKRFFGVGGKDGVPGVEEVEHVDALFTILAYLYSFCVSDYVGFLRKRL
DLDGHERIIRKSVESVRKYQDPLIDERVQLWAKGHRTESEDLLDIMISLK
DGTEPLLSVDEIKAQVLELNLATVDNPSNAVEWAIAEMINQPQTLQKAIE
ELDTLVGRNKLVQESDLPKLNYIKACAREIFRLHPFAPFNVPHVSMSDTT
VAGYFIPKGSHVLISRPALGRNPEVWEDPHRFNPERHLKEDGSEVALTDN
ELKLLSFSTGRRGCAGILLGTTIITMLLARLLQGFTWSPPPNETKVDLSE
APDCLLMANPLVAHAIPRLDHHLYPIEM
>NjCYP79A2
MESNFFTSLNFSALENAILCSTMLLLAFVFFVVKSTQKSPPLPPSPKQWP
IIGSLNALRKNKPVFRWIHKLMEEMKTNIICIRFFRTHVIVVSTDYLAKE
FLKKQDVVFSSRPICMSAEITSGGYLSVVTGPMGDQWKKMRRLLSSDIVS

STKHRWLKNKRDEEADHLVRYVYNKTGLTGGNVNVRVIAQHYCGNVIRKL
IFSKRFFGVGGKDGVPGVEEVEHVDALFTILAYVYSFCVSDYVGFLRKRL
DLDGHERIIRKSVESVRKYQDPLIDERVQLWAKGHRTESEDLLDIMISLK
DGTEPLLSVDEIKALVLDLNLATVDNPSNAVEWAIAEMINQPQMLQKAIE
ELDTLVGRNRLVQESDLPKLNYIKACAREIFRLHPFAPFNVPHVSMSDTT
VAGYFIPKGSHVLISRLALGRNPEVWEDPHRFNPERHLKEDGSEVALADN
ELKLLSFSTGRRGCAGILLGTTITTMLLARLLQGFTWSPPPNETKVDLSE
APDCLLMANPLVAHAIPRLDHHLYPIEM
>NjCYP80E1
MVSILLLLPLLLALILAVRLTTKPAATRHDQNLPPGPYSWPIIGNLPHML
KGPHIYLTELSRVYGPLFSVKLGTHLFVVGSSPMAATEIMKAHEKLPTYR
WVPKAGQEGLEQYSLIWATQCTDHWKLLRSLCRTELFSAKGLESQSSIRE
KSVDELVEFMKGNQGRIVNIREVVFASTVNILGKICFSKDLIDLDDDDRR
EKRGLKRALYRLMKLGTTPNIADFYPGFEGVDPQGLKRKTSEAMDEAFSA
WECVIRERRVAGESRKDVGFDEGDFLDTMLRCGFSDLQINQLTVELFSGG
THSTASTIEWALAELLKNKEAMITLKNELKNKIKSTNSIKESQVSHLPYL
HACVKETLRLHPPAPLLHPQAILEGCEIMNYTIPKNSQLIINVWAMGRDP
NLWEDPLVFKPERFFESNVDFKGQDFKFLPFGVGRRMCPGYPYAIKQIHL
MLASLVQKFDWFLPNGIEDLSRLDMSENFGIISQRKKPLMVIPKCIC
>NjCYP80F2
MDLATLASEYSNNIFFFLLITIIIITSLIILKYNSQTSQLLPPGPFSWPI
VGNIFEMRKNLPHETLAKLAEKHGPIMSLRFGAQLIIVGSSPAVASQILK
THDRVLSGRHLTYPVRAKSSKLHNLGLGFSDKCDDGWNSFRTIYRGEIFS
TKALNSQVSLREEKVMEMLKYLISKEGKVVKIKEIVFTTALNILSNTFLS
VDFLDYEGKGFGEGLRNKIRRFGVVGTSNTLSDIFPILYGWDFQGMCNKL
VDVVDKIFDSWAQIIRERRKGRRVIGDFADVLLQNGYKDRQINPLLLELF
SAGTDSTSATSEWALVELLKNPRVMQKLHAELSKITHGGRVKESDLPNLP
YLDACIKETLRMHPPGPLLLPHRATQTCQVMGYTIPKNSHVVVNMWSIGR
DPTIWNDSLSFKPERFLLSGLDFKGQDFEYIPFGSGRRICPGQPLAARVV
PLIVGSIVNTLVYALPDNNEPSKIDMTEMYDITMQKKEALCIVPKARTGV
NLNN
>NjCYP80F3
MDLATLASEYSNNIFFFLLITIIIITSLIILKYNSQTSQLLPPGPFSWPI
VGNIFEMRKNLPHETLAKLAEKHGPIMSLRFGAQLIIVGSSPAVASQILK
THDRVLSGRHLTYPVRAKSSKLHNLGLGFSDKCDDGWNSFRTIYRGEIFS
TKALNSQVSLREEKVMEMLKYLISKEGKVVKIKEIVFTTALNILSNTFLS
VDFLDYEGKGFGEGLRNKIRRFGVVGTSNTLSDIFPILYGWDFQGMCNKL
VDVVDKIFDSWAQIIRERRKGRRVIGDFADVLLQNGYKDRQINPMLLELF
SAGTDSTSATSEWALVELLKNPRVMQKLHAELSKITHGGRVKESDLPNLP
YLDACIKETLRMHPPGPLLLPHRATQTCQVMGYTIPKNSHVVVNMWSIGR
DPTIWDDSLSFKPERFLLSGHDFKGQDFEYIPFGSGRRMCPGQPLAARVV
PLIVGSIVNTLVYALPDNNEPSKIDMTEMYDITMQKKEALCIVPKARTGV
NLNN
>NjCYP80F4

MDLATLASEYSNNIFFFLLITIIIIILKYNSQTSQSLPPGPFSWPIVGNI
FEMRKNLPHETLAKLAEKHGPIMSLRFGAQLVIVGSSPAVASQILKTHDR
VLSGRHLTYPVRAKSSKLHNLGLGFSDKCDDGWNSFRTIYRGEIFSTKAL
NSQVSLREEKVMEMLKYLISKEGKVVKIKEIVFTTALNILSNTFLSVDFL
DYEGKGFGEGLRNKIRRFGVVGTSNTLSDIFPILYGWDFQGMCNKLVDVV
DKIFDSWAQIIRERRKGRRVIGDFADVLLQNGYKDRQINPMLLELFSAGT
DSTSATSEWALVELLKNPRVMQKLHAELSKITHGGRVKESDLPNLPYLDA
CIKETLRMHPPGPLLLPHRATQTCQVMGYTIPKNSHVVVNMWSIGRDPTI
WDDSLSFKPERFLSSGLDFKGQDFKYIPFGSGRRMCPGQPLAARVVPLIV
GSIVNTLVYALPDNNEPSKIDMTEMYDITMQKKEALCIVPKARTGVNLNN
>NjCYP80G1
MNLENFTKGEMIMFLIPWPLLFFFFFILHKIKPSKELPLPPGPHPWPIVG
NLFQIGKNAHIDLAEMAQIHGPIMSLRLGQRILIVGSSSAAASEILKTHD
HVLSGRDVSVLLQNKVSTVHNMNLVFTSETGDNWRKIRNLYTSQIFSNKA
LESRVNMRQEKVMEMVKFIGSKSGESLSIRDAMLVTVTNIMGNVSLSMDL
MDFEGNGIGAKINDSLRRLTMLGGQPPLADFYPIFGRWDMQGWYKKFMQI
VKQELGTIWEEILQMKRNRSNISLDTKDFSDILIEKGCTQQQINALMQEL
FSAGTESMSSTTEWFVTELLRNQQVMQKARDEIMKNIDGNVVKESYLIRL
PFLEACLKETLRLHPPGPLLLPHRATQTCEVMGYTIPKDSQILVNMWAIG
RDPGIWDDPLSFKPERFMGSKLSQKGKDFEYIPFSAGRRMCPGETMASKT
ILLVVASLILNFDWCLVNNMSPEDINMEEVLDVAMRKKEILYVTFKLREQ
LLKP
>NjCYP81C1
MEDFHWYILFLGLACSIHFVPKINNQQPPTLSAFKVLAYLFFFKVIDYLS
SFKQQPLHRSLHRIATLYGPISLLHLGCRPVLLISSSSAAEDLFSNKNDA
AFAHRPKLVVGKEFGSNFTNLAWAPHGGHWRHLRRVSCLEILPFHRHPDQ
FDSLSDEVKVLLRRLYDSEKIVELKPLFLDLVINVMMKMFAGKGYCPKKL
TCKGNGKESISMMDYVTHSFRMTTDEPDVGYFVPILKFLGLISLEQRCKK
LQEQGDSLMDILIEKLRTKMAHIKKSCQKEGKVIEFLLAKQKENPERYPD
QLIKGLVQVLMSAGTDTSVGVLEWAFSLLLNHPDVLRKAQNEIETYVPKD
RFLNQSDIENLPYLLCIVKETLRMYPVAPLLVPHESSKECTIGGYDIPKG
TMLMVNAWAIHNDPKIWPEPEKFKPERFENIVSERDGFKLMPFGYGRRSC
PGKHMAMRVITFTLGSLLHTFNWEKVEKTVDMTELTGLALFKAQPLKAVC
HPRLAMMNLRSQI
>NjCYP81D1
MDFYFISLSFSLSLFFFIFIHKILIKKSNKKLPPSPPSWPIIGHLHLLKE
PVHRCLQDLSYTYGPIFKLKFGSIPIIIISSPKLFEECFSKNDVVFADRP
PLLSGKHLGYNNTSIVFASYGPHWRNMRRVTSIEFFSNNRLNAYLIVRKE
ETVSLLKNLYRDSSLSNFNRVEMKSRLAELSFNVVMRMASGKRYFGVDVE
NAEEANIFRKIINDVFEMSGANQPNEFFKFLQWIDFQGFEKKMVRLQKRS
DAFLQSLVDGIRNKRKEIGINDENNKTLISAMLALQESEPDYYTDDLIKG
NIMLVLMAGTDTSSSAVEWAMSLMLNHPEVLEKARAEIDRNIGHDHIVDE
EDLSKLPYLQCVVNETLRLFPTAPLLVPHMSSEDCVVGGFDIPHGTMLMA
NAWALHRDPNVWDDPSSFKPKRDLRVRELRALNMCHLEWEGDNVLALDLG

IAW
>NjCYP81D2
MDFYFISLSFFLSLFFFIFIHKILIKKSNKKLPPSPPSWPIIGHLHLLKE
PVHRCLQDLSYTYGPIFRLKFGSIPVIIISSPKLFEECFSKNDVVFADRP
PLLSGKHLGYNNTSIVFASYGPHWRNMRRVTSIEFFSNNRLNAYLIVRKE
ETVSLLKNLYRDSSLSNFNRVEMKSRLAELSFNVVMRMASGKRYFGVDVE
NAEANIFRKIINDVFEMSGANQPNEFFKFLQWIDFQGFEKKMVRLQKRSD
AFLQSLVDGIRNKRKEIGINDENNKTLISAMLALQESEPDYYTDDLIKGN
IMLVLMAGTDTSSSAVEWAMSLMLNHPEVLEKARAEIDRNIGHDHIVDEE
DLSKLPYLQCVVNETLRLFPTAPLLVPHLSSEDCVVGGFDIPRGTMLMAN
AWALHRDPNVWDDPSSFKPERFEGGRIEGSKYVPFGMGRRQCPGAGLGNR
MVNLCLAALIQCFEWEIVNEDLVDLSESNGLTMPKKIPLEAMCKQREQAI
NVLQKL
>NjCYP81D3
MEEQTWFYYFLSFSLLLVIFFLSKLFIKKSNKKLPPTPPSYPIIGHLHLL
KPPLHRTLQNLSYTYGPIFRLKFGSLPVIVISSPKFFEECLSKNDINFCN
KPTTLVGKHLGYNDTAVGLAQYGPHWSNIRRVLTQNFFSTNRLNAYYTVR
QDEKARLVKNLYDGSNLNYRRVEMRSRLTELTFNSVMRMATGKRIFGADV
EDVEEGKKFMEIISDNFEMSKMNQANDFFKVLRWIDFQGFEKKLIGLHKK
TDVFMQSLVDQIRSRRKETGTKGQDISKTLIDEMLTLQESDPEYYTDDNI
KANVGSVVSAGTDTSSATIEWAMALLLNHPEVLEKARVEVDINIGHDNLV
DESDVSKLPYIQCIVNETLRLFPIGPIIPPHMSSEDCVVGGFHIPRGTML
LANAWALQRDPKVWDDPTSFRPERFEGAHIEGYKFVPFAVGRRQCPGVGL
ANRVVSMSLAVLIQCFEWKRVNEKLVDLSEADHGATIPMANPLKAMCKPR
EEMINVLSKL
>NjCYP81D4
MEIYSVFLYSVLSLLFIFVLKKLITKNNKKLPPSPPSYPIIGHLHLLKPP
LHRTLENLYHIYGPIFSLKFGSTPVLVVSTPDLFEECFSKNDISFADRPK
VLLGKHLGYNGTAVAFAQYGPHWRNQRRVLTLEFLSATRINQYFTVRKEE
RATLVKNLYAESTSSYTKIEMRSKLTELTFNNVMRMATGQRLFGADVEDV
EGAKGFRQIISEIFEMTGCNMPNDFFKVLQWIDFRNYEKKMAKLQQRSDA
FLQTLVDRIRSQRKANGGHNGKTFIDEMLTLQEAEPSYYTDDIIKGNIST
VLFAGTDSSAAAIEWAMSLLLNHPEKLEKARAEIDMKIGFDRLVDEADVA
KLPYFQCIVNETLRLFPISPIICAHMSSEDCVVGGFDIQRGTLLLANAWA
LQRDPKVWDDPTSFKPERFEGLQSEGWKFVPFGMGRRQCPGVTMANRVVS
MSLAALLQCFKWERVSEDLVDLSEAPMGMTMPKKIPLEAMCKPREELISV
LSKL
>NjCYP81D5
MEEQTWFSFFFLSSFFSLILLFFFLPKLIQTQKSKKKLPPSPPSYPIIGH
LHLLKEPVHRTLENLSHKYGPIFGLKFGSSPVLLVSSPELLEECFTKNDV
IFANRPQFLLGKHIGYNDTAMAFASHGPHWRNLRRVTTLKFFSNSRLNEY
MALRKEETVSLVKNLFNVTRSGKRSVEMKSRLLELTFNVVMRMATGKRYF
GADVEDSEEAERFRGIIADIFDVTGTSNPNDFLKFLQWIDFQGFEKKMVS
LQKRTDEFLQFLVDEIRNQRSAYLKDDFRGQRKESTEEKNSQTLVDEMLA

LQEVEPGYYSDELIKGNISVVLLAGTDTSSSTVEWAMSLLLNHPDVLEKA
RAEIDINIGHDRLVEESDISKLPYIQCIMNETLRMQPVSPLIAPHVASED
CVIGGYHIPRGTMLLPNAWALHRDPNIWEDPTSFKPERFKGAQIEGNKLG
FIFVPFGMGRRQCPGAGLANRVVNHTLAALIQCFDWERVSEELVDLTEGK
GITMPKRDPLEAMCKPREKMINVLLKL
>NjCYP81D7
MEEQTWFSFFFLSSFFSLILLFFFLPKLIQTQKSKKKLPPSPPSYPIIGH
LHLLKEPVHRTLQNLSHKYGPIFGLKFGSSPVLLVSSPELLEECFTKNDV
IFANRPQFLLGKHIGYNDTAMAFASHGPHWRNLRRVTTLKFFSNSRLNEY
MALRKEETVSLVKNLFNVTRSGKRSVEMKSRLLELTFNVVMRMATGKRYF
GADVEDSEEAERFRGIIADIFDVTGTSNPNDFLKFLQWIDFQGFEKKMVS
LQKRTDEFLQFLVDEIRNQRSAYLKDDFRGQRKESTEEKNSQTLVDEMLA
LQEVEPGYYSDELIKGNISVVLLAGTDTSSSTVEWAMSLLLNHPDVLEKA
RAEIDINIGHDRLVEESDISKLPYIQCIMNETLRMQPVSPLIAPHVASED
CVIGGYHIPRGTMLLPNAWALHRDPNIWEDPTSFKPERFKGAQIEGNKLG
FIFVPFGMGRRQCPGAGLANRVVNHTLAALIQCFDWERVSEELVDLTEGK
GITMPKRDPLEAMCKPREKMINVLLKL
>NjCYP81D8
MEEQTWFSFFFLSSFFSLILLFFFLPKLIQTQKSKKKLPPSPPSYPIIGH
LHLLKEPVHRTLENLSHKYGPIFGLKFGSSPVLLVSSPELLEECFTKNDV
IFANRPQFLLGKHIGYNDTAMAFASHGPHWRNLRRVTTLKFFSNSRLNEY
MALRREETVSLVKNLFSVTRSGKRSVEMKSRLLELTFNVVMRMATGKRYF
GADVEDSEEAEKFRGIIADIFDVTGTSNPNDFLKFLQWIDFQGFEKKMVS
LQKRTDEFLQFLVDEIRNQRSAYLKDDFRGQRKESTEEKNSQTLVDEMLA
LQEVEPGYYSDELIKGNISVVLLAGTDTSSSTVEWAMSLLLNHPDVLEKA
RAEIDINIGHDRLVEESDISKLPYIQCIMNETLRMQPVSPLIAPHVASED
CVIGGYHIPRGTMLLPNAWALHRDPNIWEDPTSFKPERFKGAQIEGNKLG
FIFVPFGMGRRQCPGAGLANRVVNHTLAALIQCFDWERVSEELVDLTEGK
GITMPKRDPLEAMCKPREKMINVLLKL
>NjCYP81D9
MEEQTWFSFLSLPSFFTFIILFFLLPKLIQTQKSKKKLPPSPPSYPIIGH
LHLLKIPVHRTLQNLSYTYGPIFSLKFGSSPVLILSSPDLLEECFSKYDV
VFANRPQILLGKHLGYNDSSMSFAPHGPHWRNLRRVLTLKIFSTNRLNAY
MALRREETVLLVKDLFKVTRSGYKRVVMNDRLLELSFNVVMRMACGKRIF
GADVENMEEATKFRGIISEIFEMAGASNPNDYVKFLHWIDFQGFEKKMVS
LQERSDAFLQSLVDGIRSQRKANDSEGHNPQTLIDEMLGLQEAEPEYYTD
DLIKGNISNVLLAGTDTSSSTTEWALALLLNHPEELKKVRDEIDMNIGHD
HLVDETDLSKLPYLQCIVHETLRIFPVSPLLAPHVSSEDCVIGGFDIPRG
TMVLANAWALHRDPKVWEDPLSFRPERFKGAQIEGYKFVPFGMGRRKCPG
AGLANRVVCRSLAALIQCFDWERVSEELVDLSEGHGLTMPKKLPLEAMCK
PREQMMQVFQKL
>NjCYP81D10
MEEQTWYYLVSLSSVFLCFFLYKKLFTKNSNKNLPPGPPSLPIIGHLHLI
KVPVHRTLQKFSYTYGPIFSLKFGSHPVIVVSSPELLEECFSKNDIVFAN

RPHLLVGKHLGYNDTGMAFAAYGPHWRNLRRVTTLKFFSTNRLNAYMSLR
REEAVYLVKDLFNASRSAPSKVEMKDRLLALSFNSVMRMATGKRYFGTDV
ENFEEAEKFRGIICDIFEASGAGNPSDFIKFLRWIDFKGSEKKFISLQER
TDFFLQKILDEIRDQRKATGVVGQTLMDELLDFQEAEPTHYTDTLIKANI
STILLAGTDTSSSNTEWALALLLNHPEVLKKAREEIETNIGYDRLVEETD
ISNLPYIQCIVNETLRIQPVSPLLCPHLSSEDCVIGGYHIPRGTMLLPNA
WALNRDPKVWDDPTSFKPERFQGTKIGAYQFMPFGMGRRQCPGNTLANRV
VNHSLAALIQCFDWERESEELIDLTEGLGITMPIKKPLEAICKPREQMLH
VLTKL
>NjCYP81D11
MEEQTWFYLISLSSFLIFLFIIRKFFTKNDNKNLPPGPPSLPIIGHLHLI
KVPVHRTLQKFSYTYGPIFSLKFGSHPVIVVSSPELLEECFSKNDIIFAN
RPHLLVGKHLGYNDTGLAFAAYGPHWRNLRRVTTLKFFSTNRLNAYMSLR
REEAVYLMKDLFNASRSAPSKVEMKDRLLALSFNSVMRMATGKRYFGTDV
ENFEEAEKFRGIICDIFEASGAGNPSDFIKFLRWIDFKGSEKKFISLQER
TDFFLQKILDEIRDQRKATGVVGQTLMDELLDFQEAEPTHYTDTLIKANI
STILLAGTDTSSSNTEWTLALLLNHPEVLKKAREEIETNVGYDRFVEETD
MSKLPYIQCIVNESLRIQPVSPLLCPHLSSEDCVIGGYHIPRGTMLLPNA
WALNRDPKVWDDPTSFKPERFQGTKIGAYQFMPFGMGRRQCPGNTLANRV
VNHSLAALIQCFDWERESEELIDLTEGLGITMPIKKPLEAICKPREQMLH
ILTKF
>NjCYP81D12
MEEQTWFYLISLSSFLIFLFIILKFFTKNDNKNLPPGPPSLPIIGHLHLI
KVPVHRTLQKFSYTYGPIFSLKFGSHPVIVVSSPELLEECFSKNDIIFAN
RPHLLVGKHLGYNDTGLAFAAYGPHWRNLRRVTTLKFFSTNRLNAYMSLR
REEAVYLMKDLFNASRSAPSKVEMKDRLLALSFNSVMRMATGKRYFGTDV
ENFEEAEKFRGIICDIFEASGAGNPSDFIKFLRWIDFKGSEKKFISLQER
TDFFLQKILDEIRDQRKATGVVGQTLMDELLDFQEAEPTHYTDTLIKANI
STILLAGTDTSSSNTEWTLALLLNHPEVLKKAREEIETNVGYDRFVEETD
MSKLPYIQCIVNESLRIQPVSPLLCPHLSSEDCVIGGYHIPRGTMLLPNA
WALNRDPKVWDDPTSFKPERFQGTKIGAYQFMPFGMGRRQCPGNTLANRV
VNHSLAALIQCFDWERESEELIDLTEGLGITMPIKKPLEAICKPREQMLH
ILTKF
>NjCYP81D13
MEWFYLVSLSSFLIFLFIVRKFFTKNDNKNLPPGPPSLPIIGHLHLIKVP
VHRTLQKFSYTYGPIFSLKFGSHPVIVVSSPELLEECFSKNDIIFANRPH
LLVGKHLGYNDTGLAFAAYGPHWRNLRRVTTLKFFSTNRLNAYMSLRREE
AVYLMKDLFNASRSAPSKVEMKDRLLALSFNSVMRMATGKRYFGTDVENF
EEAEKFRGIICDIFEASGAGNPSDFIKFLRWIDFKGSEKKFISLQERTDF
FLQKILDEIRDQRKATGVVGQTLMDELLDFQEAEPTHYTDTLIKANISTI
LLAGTDTSSSNTEWTLALLLNHPEVLKKAREEIETNVGYDRFVEETDMSK
LPYIQCIVNESLRIQPVSPLLCPHLSSEDCVIGGYHIPRGTMLLPNAWAL
NRDPKVWDDPTSFKPERFQGTKIGAYQFMPFGMGRRQCPGNTLANRVVNH
SLAALIQCFDWERESEELIDLTEGLGITMPIKKPLEAICKPREQMLHILT

KF

>NjCYP81D14

MEEQTWFYLISLSSFLIFLFIVRKFFTKNDNKNLPPGPPSLPIIGHLHLI
KVPVHRTLQKFSYTYGPIFSLKFGSHPVIVVSSPELLEECFSKNDIIFAN
RPHLLVGKHLGYNDTGLAFAAYGPHWRNLRRVTTLKFFSTNRLNAYMSLR
REEAVYLMKDLFNASRSAPSKVEMKDRLLALSFNSVMRMATGKRYFGTDV
ENFEEAEKFRGIICDIFEASGAGNPSDFIKFLRWIDFKGSEKKFISLQER
TDFFLQKILDEIRDQRKATGVVGQTLMDELLDFQEAEPTHYTDTLIKANI
STILLAGTDTSSSNTEWTLALLLNHPEVLKKAREEIETNVGYDRFVEETD
MSKLPYIQCIVNESLRIQPVSPLLCPHLSSEDCVIGGYHIPRGTMLLPNA
WALNRDPKVWDDPTSFKPERFQGTKIGAYQFMPFGMGRRQCPGNTLANRV
VNHSLAALIQCFDWERESEELIDLTEGLGITMPIKKPLEAICKPREQMLH
ILTKF

>NjCYP81D20

MEQTSWTLFLISLSTFLFLFTLSKILIKKQINHKNLPPSPPSIPIIGHLH
LVKEPLHRTLQDFSQKYGPIFSLKFGSRPVVIISSPSAMKQCFTTNDIVF
ANRPLLMFGKYIDYDHTTIGSTPYGPIWRDLRRISVLEFFSTTKLNAYLG
IRQEEIKILLKNLHQDSSKGFRRVEMKSRLSALTFNVVMRMITGKRYIGS
KVEEDSKEAKEFRDLIRETFELGLATNLSDFLSICKLIDFGNIEKRMIAL
HKKMDLVLQGLVDERRSTQEVTTNKTLIGAMLSLQESEPQSYTDITIKGM
ISTLLLAGTDTSSATIEWAMSLLLNHPEELKKARVEIDSYIGHNRLADES
DLPELRYLQCIVNETLRLFPVTPLLVPHEPSEDVRIGGFDIPRGTMLLAN
AWAIHRDPNIWDDPTSFKPERFENGVNEPYTFVPFGVGRRQCPGAGLANR
IVTLCLATMIQCFEWERIDEEFIDLCEGKGFTMHKHEPLGAMCKVRQEMS
HILTML

>NjCYP81E1

MEESNWLFATTLLSLLFLALKFFFFSNTNKKINLPPSPAPALPVIGHLYL
FKPPLYRTFLRLSKKTGPIFSVQFGSRLVVVISSPSAVEECFTKNDVVLA
NRPRFIIGKYIGYNYTTIAGSSYSDHWRNLRRLTSIEIFSSTRLNAFLSI
RRDEVRLMLRKLYDSGASASKVELKTKLSELTFNIILRMIAGKRYFGEDV
EDQEEAVQFRNLIKEVIKYGGASNPGDFLPLLRWIDYGGFKKNLTRIGKQ
MDSLLQGLIEEHRRDKNKNTMVDHLLSLQESEPEYYTDEIIKGLMIVIVT
AGTDTSSVTIEWALSLLLNHPKILKKARDELDREVGHERLVDEPDLAKLG
YLQNIILETLRLFPAAPLLLPHESSEDFRLGGYVIPRGTILLVNAWAIQR
DPEVWDDPTSFKPERFDEISISNTSNVSRGVVVADNKLMPFGMGRRSCPG
SRLAQRVVGLALASMIQCFDWERLSDDRIDLAEGVVGVTMAKAEPLEAVC
RPCMFTHNLLFGGESQMNL

>NjCYP81E2

MEESNWLFATTLLSLLFLALKFFFFSNTNKKINLPPSPARALPVIGHLHL
FKPPLYRMFLDLSKKTGPIFSVQFGSRLVVVISSPSAVEECFTKNDVVLA
NRPRFIIGKYIGYNYTTISGSSYGDHWRNLRRLTSIEIFSSTRLNAFLSI
RRDEVRLMLRKLYDGGASASKVELKTKLSELTFNIILRMIAGKRYFGEDV
EDQEEAVQFRNLIKEVVKYGGASNPGDFLPLLRWIDYGGFKKNLTRIGKQ
MDGLLQGLIEEHRRDKNKNTMVDHLLSLQESEPEYYTDEIIKGLMIVMVT

AGTDTSSVTIEWAMSLLLNHPEILKKAQDELDREVGHGRLVDEPDLAKLG
YLQNIILETLRLFPAAPLLIPHESSEDFRLGEYDIPRGTILLVNSWAIQR
DPEVWDDPTSFKPERFDENSTSNTSSVTGGVVVANNKLMPFGLGRRSCPG
SGLAQRMVGLALASMIQCFDWERISDDLVDLAEGVGVTMPKAEPLEAVCR
PRMFMHNLLFGGESEMNL
>NjCYP81E3
MEETSWLYAATLLSILFLAFKIFVSNTNKKINLPPSPARALPVIGHLHLF
KPPLYRTFLRLSKKTGPIFSVQLGSRLVVVISSPSAVEECFTKNDVVLAN
RPRFIIGKYIGYNYTTISGSSYGDHWRNLRRLTSIEIFSSTRLNAFLSIR
RDEVRLMLRKLYDGGASASKVELKTKLSELTFNIILRMIAGKRYFGEDVE
DQEEAVQFRNLIKEVVKYGGASNPGDFLPLLRWIDYGGFKKNLTRIGKQM
DGLLQGLIEEHRRDKNKNTMVDHLLSLQESEPEYYTDEIIKGLMIVMVTA
GTDTSSVTIEWAMSLLLNHPEILKKAQDELDREVGHGRLVDEPDLAKLGY
LQNIILETLRLFPAAPLLIPHESSEDFRLGEYDIPRGTILLVNSWAIQRD
PEVWDDPTSFKPERFDENSTSNTSSVTGGVVVANNKLMPFGMGRRSCPGS
GLAQRMVGLALASMIQCFDWERISDDQVDLAEGVGVTMPKAEPLEAVCRP
RMFMHNLLFGGESEMNL
>NjCYP81E4
MEETSWLYAATLLSILFLAFKIFVSNTNKKINLPPSPARALPVIGHLHLF
KPPLYRTFLRLSKKTGPIFSVQLGSRLVVVISSPSAVEECFTKNDVVLAN
RPRFIIGKYIGYNYTTISGSSYGDHWRNLRRLTSIEIFSSTRLNAFLSIR
RDEVRLMLRKLYDGGAFASKVELKTKLSELTFNIILRMIAGKRYFGEDVE
DQEEAVQFRNLIKEVVKYGGASNPGDFLPLLRWIDYGGFKKNLTRIGKQM
DGLLQGLIEEHRRDKNKNTMVDHLLSLQESEPEYYTDEIIKGLMIVMVTA
GTDTSSVTIEWAMSLLLNHPEILKKAQDELDREVGHGRLVDEPDLAKLGY
LQNIILETLRLFPAAPLLIPHESSEDFRLGEYDIPRGTILLVNSWAIQRD
PEVWDDPTSFKPERFDENSTSNTSSVTGGVVVANNKLMPFGMGRRSCPGS
GLAQRMVGLALASMIQCFDWERISDDQVDLAEGVGVTMPKAEPLEAVCRP
RMFMHNLLFGGESEMNL
>NjCYP81E5
MLSQKKKKNYMEETSWLYAATLLSLLFLAFKIFFSNTNKKINLPPSPAPA
LPVIGHLYLFKPPLYRTFLRLSKKTGPIFSVQFGSRLVVVVSSPSAVEEC
FTKNDVVLANRPRLIVGKYIGYNYTTVGNSSYGDHWRNLRRLTSIEIFSS
TRLNAFLSIRRDEVRLMLRKLYDGGASASKVELQTKLSELTFNIILRMIA
GKRYFGEDVEDQEEAVRFRNLIKEVVRYGGTSNLGDFLPILQWIDYGGFK
KNLTRIGKQMDGLLQGLIEEHRRDKNKNTMVDHLLLLQESEPEYYTDEII
KGLMIVMVTAGTDTSSVTIEWAMSLLLNHPKILKKAQDELDREVGHERLV
DEPDLAKLGYLHNIILETLRLFPVTPLLVPHESSEDFRLGEYDIPRGTIL
LVNAWAIQRDPEVWDDPTSFKPERFDEISTSSVTGGVVVANNKLMPFGMG
RRSCPGSGLAQRMVGLALASMIQCFDWERLSDDRIDLAEGVVGLTMPKAE
PLEAVCRPRMFTYNLLFGGESGMNL
>NjCYP81F2
MEVPYVILTFLLILVSYLFTSKFRGKFSNLPPTVFPSLPIIGHLYLLKAP
LYRTFAKISARYGPVVLLKFGARRVLLVSSPSAVEECFSKNDIILANRPR

MLFGKIVGVNYTSLAWAPYGDNWRNLRRIASIEILSIHRLNESHNIRAEE
GRFLVRNLLSSSSSPVTMKNVFYELTLNVMMRMIAGKRYFGSDNPKLEEE
GIRFRDMLNETFLLAGASNVGDYLPILSWFGVKGLEKRLIALQEKRDAFF
QGIIEEIRKSKGSEAENKRKTMIEVLLSLQESDPEYYTDALIRNFVLSLL
AAGSDTSTGTMEWAMSLLLNHPEVLKNAQNEIDQIVGKDRLVDESDIPNL
PYLRGIINETLRIKPAGPLLVPHEASEDCTIGGYNIPRGTMVLVNQWAIH
HDPELWRDPESFKPERFEGVEGTRDGFKLLPFGAGRRSCPGEPLAVRVLG
MTLGSIIQCFNWERIGKEMVDMSEGPGLTMPKAIPLVAICKPRLEMQSLL
SQL
>NjCYP81F3
MENFYHLSLFLTFILLYIFTKTFIHKFQNLPPTPSFSFPIIGHLYLLKKP
LHRTLSQISARYGSILLLHLGSRRVLLVSSPEIVEECFTKNDAVFANRPK
LLAGKHLGHDYSTIPWAPYGDHWRNLRRICSVEIFSSHRLQALQGIRADE
VKRMVSRLRRDSRKEVDMKVMFFELMLNVMMRMIADKRFYGEDVEEASRA
KEFREMVSETFRLAGASNLEDFLPVLKLLGKSGLEKKLMVLTKKRDEFMR
ELIEEQRMKMSDVNGERKTLIQVLLSLQQSEDSDYYTDQLIVGIMLAFLV
AGTDTSVGTMEWALSLLLNNPSILKKAQNEIDNHLGQTRLLIESDIQNLP
YLRCIINETLRMYPAGPLLVPHESSEPCMVGGYRIPAGTMLMVNQWSIQN
DPKLWVEPQKFMPERFEGLEGARDGFKLMPFGSGRRGCPGEGLALRVVGL
ALGSLIQCFDWERISEELIDMTEGPGLTLPKSKPLVAKCLPRLAIMELIA
QI
>NjCYP81G1
MTEMPFWFTFSLILTFSLFFLFEIFFNKRKNYKNLPPTPPSLPIIGHLHL
LKDPLYKSLHTLSLKYGPIIRLRFGLRRVLVVTSAEAVEECFTKNDIVFA
NRPRLILEKHLNYDYTTIGAAPYGDLWRRLRRVSSAELLSPARLAATASV
RRSEIGLLAKQLAKSCGNGGANVNLKLRFSEVSINVISILMMGKRYYGDD
VADPTAAVRFKGLMKEFLDLIKLSNIGDFLPFLRWFDFQGMERRMVELME
NMDKFLQEIVDEHRRNLSVSKDADGSEEVNSLTMVDNLLLLQKTEPELYP
DQIIKGLIMVMLIAGTESTYTTMEWAMSLLLNHPQTLLKLKDEIDTNIGR
RQLLEEDDLHKLPYLQNVIAESLRLYTPVPLLVPHHASQDCTVGGYNVPR
GTMLLVNAWAIHRNPKVWESPTEFKPERFEKENEDGGGYRLVPFGAGRRG
CPGVGLANRMLGLVLGTLVQCFEWQRIGGELVDMSSTAGISLSKLNSLEA
ICTPRLDMIPHLV
>NjCYP81Q31
MLCVLTNRLLQKLQNLPPTPFPKLPLIGHLHLLRKPYHLSLYELSNRYGP
IYSFQFGSRRAVIVSSPSAVEECFTKENDVVFANRPRLLVGKHLGYNYTS
LAWAPYGDHWRNLRRISSLELLSPQRLQFLSRIRVDEVRRLTRRLFEDTA
GKLGRTVDLRSAFFEVSYNGLMRMIAGKRYYGHVTNTDEAMRFREIVSDT
SRVGLESAIGDFLPLIAALRGIEIEKNLRELHVKRDNFMQDLLDEHRGFR
FDDTSSSTMIEILLSLQQKDPEYYSDEMIKALLTVLFQAGTDTSAATMEW
AMANLLNNPLVLNKVQTEINNKVGQERLINESDLLELPYLECIINETLRL
HPVFPLILPHESSKDCAVGGFKIKQGTMLIVNLWALQNDPTVWVDPKRFN
PDRFLEGSGVEIAKKCFNFMPFGSGRRRCPGERLGLLMVGLTLGSLLQCF
EWERTDNEFVDLTCSSGATVAMAKPLLATCRPRPPLATLVSQI

>NjCYP81Q32
MEYLFYIPLFLVLYVLTHRVVHKLQNLPPSPFPAIPFIGHLYLIKKPLHR
SLAQLSDRYGPLIYLWLGSRPVLVVSSTSAAEECFTKNDIIFANRPHLLV
GKYFGYNYTSITWGSYGDMWRNFRRISSIEILSSHRLNMLSHIRVDEVRE
LIHHVCQISNEDPGRAIEMRSVFIKFMFNVMTRMISGKRYYHWNDASESK
EAKRFEEIVTETGKIVGQEAMGDFVPLIRWFVWKGVEKRYAELHRKRDQF
MQDLIDEFRRTGSDNSSSGGRDKNLIQILLSLHEKEPENYTDETTRSLMN
SLLHAGTNTSVETMEWAMSLLLNNPKVLKKAQYEINKRVGQDRLINESDV
AELPYVRNIIKETLRMHPAAPLLLPHESSKNCSVGGFHIPRGTMLLVNLA
AIQNDPEVWEEPERFRPERFEIGVEKEGFRLMPFGIGRRACPGEGLALRI
LGLALGSLIQCFDWERVSEELVDMTESSGVTTPKAQPLIAKCSPRPTMVD
ILPKIK
>NjCYP82A1
MDLSQLQQNYTLLAGTLAIIFIILVLTLVPKPAKYRPPPEAGGAWPIVGH
VNLFTDSSNLPHRALAAMAAKYGPIFTVRLGIHKVLVVHSWQIAKEIFTI
HDQIISNRPKYLASKIFGYNYAMFGVAPYGPYWREIRRIISFELLSNSRL
EHLRHVRESELEISIKNLYDLWREKRDDQGKVKVEMTKWFGEFNMNVVLR
MVAGKRYSGGTDGEEDEEKEMSASREVMRKFFYFMGLFVVGDTLPFLGWL
DLGGHEKAMKKAAHELDIMAGKWLDEHRRKRESEEAFEDKDFMDVMISTV
EKGGFGDYDADTIIKSTCMVLIASSADTTTVMLTWVLSLLLNNRNSLRKA
QEEIDKVVGKDRQINESDITNLVYLQAIVKETLRLYPAGRLGGMREFTED
CTVAGYHVPKGTWLMVNLWKLQQDPEIWSEASEFRPERFLDGNLKNIDVK
GTNFELIPFGAGRRSCPGMGLALQMLHLGLAALLQNFNITTPRDGPIDMS
ESAGLTSAKASPLEVLIAPRMPSTFT
>NjCYP82A2
MDFFPLQTNNHIAIVGALAIILLIYYLSTKLRHDKIKPPPSPPQAGGAWP
LIGHLHLLGGGPQLPHITLGAMAEKYGPIFTIRQGVYLVVVVSDWEVAKE
LFTKYDVSISSRPKFLAAKYLGHDYANFAFSPYGPYWREMRKITSQELLS
NGRVEQLKQVIVSETATCVKELYKLWTEKINDSGCIDVEMKRWFNDLTLN
VVVRMVVGKRYYKDAGDEKEARKCQKIMRDFFYFFGLFFVSDSIPFLRFL
DLGGHKKAMKKVGKNMDTMMQKWMEEHRQNNVYGQDFMGAMMNVVEKSQL
ADFDNDTIVKSTCSTIIAGGSDTSAVMLTWTLSLLLNNQNALKRAQEELD
IHVGRERQVDESDINKLVYLQAIVKETLRLYPAASLGGLREFTKDCTVLN
YHIPKGTRLIVNLWKLQRDERVWSNPLEFRPERFLDGRHKDIDVKGKHFE
LLPFGAGRRLCPGVAYGIQILHLVLATLLHGFEISTPSNTPVDMTESAGL
TNIKATPLNVLLSPRLSSHLY
>NjCYP82A3
MDFIPLQTNNHIAIVGALAIILLIYYLSTKLRHDKIKPPPTPPQAGGAWP
IIGHLHLLGGGPQLPHITLGAMAEKYGPIFTIRQGVHLVVVVSDWEVAKE
LFTKYDVSISSRPKFLAAKYLGHDYANFAFSPYGPYWRQMRKITSQELLS
NGRVEQLKQVIVSETATCVKELYKLWTEKRNDSGCIDVEMKRWFNDLTLN
VIVRMVVGKRYYKDAGDEKEARKCQKIMRDFFYFLGLFFVSDSIPFLRFL
DLGGHQKTMKKVGKEMDTMMQKWMEEHRQNNVYDQDFMGAVMNVVEKSQL
ADFDNDTIVKSTCSTIIAGGSDTSAVMLTWTLSLLLNNENALKRAQEELD

IHVGRERQVDESDINKLVYLQAIVKETLRLYPAGPLGGLREFTKDCTVLN
YHIPKGTRLIVNLWKLQRDERVWSNPLEFRPERFLDGRHKDIDVKGKHFE
LLPFGAGRRLCPGVAYGIQILHLVLATLLHGFEISTPSNTPVDMTESAGL
TNIKATPLNVLLSPRLPSHLY
>NjCYP82A4
MDNFLPQPNSLISILGAFALVLLIYYLSTKLTHAKTKSPPSPPRAGGAWP
ILGHLHLLSGKPQLPHITLGAMADKYGPIFKIWFGVHQAVVVSDWEVAKE
LFTTYDANVSSRPKFLAAKYLGNDYVMFAFSPYGSYWREMRKIVSLKLLS
SRRLELLKQVRVSETLTCINQLYKSCTGKRNHSGRTIVEMKQLFVDLTLN
VIIRMVAGKRYFGVDMDGSDEKEARNCQKTIRNFFYFLGLFLLSDAIPFL
RFLDLGGHEKAMKKTAEEMDMMMEKWMEEHRQKKVSSDIKGVQDFMDIMM
TIVEGAEFGDMDKDTIIKSTCSNIIAGGSDTTAVMLTWTLSLLLNNPNVI
QRAQEELDIHVGKERQVDESDISKLVYLQAIVKETLRLYPATPLGGMREF
TEDCTVSHYHIPKGTRLILNLWKLQRDPRVWSNPLEFKPERFLGAHKEVD
VKGQHFELLPFGAGRRVCPGTAFGVQILHLVLATLLHSFQLSTPSNAPVD
MTESAGLTNIKATPLNVLLAPRLPSHLY
>NjCYP82B1
MDLLLSFVTLAASIICFLLFCHTWLKSGVHRSKNPPEASGSLPIIGHLHL
LASSDQAPHKLFGSMADKFGPIFTIKLGVYRALVVNNADMAKECLTTNDR
VFAGRPKIMASELMAYNYASLALAPYGPYWREMRKIVVLELASQHRVQVL
EHIRVSEVKAFVTNMYENWVKNRGSSETIEVDMTQLFGNLNLNMTLRMAF
GDGFSRGDKQKEDEVKNTIKRIVELFGAFVPSDAIPALRWLDIGGYEKEM
KKLAKVLDDIIEGWLQDHKKKMSSTTQHIDEGENQVFMAALLSRVQEAFK
ENIHGFSIDAMVKATCVAVVVAAMDTTTATLTWTLALLVSNPNVLKKAQE
ELENHVGKDRMVEESDLNNLVYLQAIIKESMRLYPALPLSVPHESTEDCI
VGGYTVPKGTRLFVNFWKIQHDPEIWEDPFEFKPERFLTREKEIDVKGHN
FELIPFGSGRRICLGLSVAMKAMQLILASLIHAYDFQNPSSEKIDMTGSS
GATNHKANPLELLVAPRLSPDCMPLVCKV
>NjCYP82B2
MDFLLSLFTLITGVFCFLFIGRLAWTKSEASSSSKVTVKTVPEASGSWPI
IGHLLVLAGSQLPHKLLGSMADKFGPLFTIKLGAYRVLVVNSAEMAKECL
TTNDKVFAGRPKSMATELMGYNYASFSLAPYGSYWRDIRKMIVIELGSQR
SLQMLSHTRATELKSSVTDLYTKWKENRGSSETVKVDMKQWFGNLILNST
LKVIFGKSFSPGEHNEEEIKNTLKQSLQLLGAFVPSDVIPGLRWLDIGGY
SKKMKKTAKYLDVAIERWLEEHTLKTNSTQEDDEGKDHVFMAAFLTRLKQ
ELKDDMYGFSVGAILKSTCLAVFSAATDTTTVTLTWALALLVNNPHVLKK
AQEELETHVGKDRKVEESDLRNLVYLQAIIKESMRLYPPVPLSFPHESTE
DCIVGGYTVPKGTRLFVNLWKIQHDPKIWEDPFKFIPERFLTSKKDIDVK
GQHFELIPFGSGRRICLGIPFALSTVTMVLATIIHGFEFKNPTSEKTDMT
ERFGLTNQIAAPLELLISPRFLPDFKLST
>NjCYP82B3
MAAGAWPITGHLHLLMGKELPYRIFSSMATKYGPLFTLKFGVHQALVVSS
SEMAKECFTTNDKAFGDRPKSLSIELLGYNYAGFGFVPFGPYWRVLRKIA
TLELLSQIRQNTLAHVRTSEMKFFLKDLYRSWKNGKGSSGKATIEMGQWC

DNLIFNIILRMISGKRYSPGDKEGDRFKQTMKDYVHLSGTTVIGDIVPWL
GWLDFRGLEKKMKKTAQEVDDFFQDWLNEQKEKLSSGKHIEPKDQVFIGA
MLARLDNEFEKDLTVFNRETVVKATSLTIMAAGSDTTTMTLAWAIALIVN
HPRVLKKAREEIDKHVGRDRLVEESDLKNLVYLQALVKETMRLYPAAPII
FPHESTEDCIVGGYKIRKGTRLMVNLGKIHRDPKTWSEPEEYRPERFLTT
HKDLDVYGQSYEYFPFSSGRRICIGVALAFQVLQMSLATLIQAFELETPN
GEPIDMTESPGLVNARANPLEVVMSPRLSHKFYEEVDY
>NjCYP82C3
MVFYIHLHQITLYGLIFAIMCAWIIIINPIRNKKKPRGPPEPGRAWPFIG
HLLLLKPNDLLHRKLGAMADEYGPIFSMKLGAHQTLVISSSQLAKECFTV
HDRVFPNRPKNLAVKLMGYNHAMLGFAPYGPYWRDVRKLAVVELLSGRRL
DMLKHVWHTEIDFFMKMLYDQWVGTGGRAPVLVDMKELFGDMAMNIVVRM
VAGKRYFGKGTLFDEESKRCQKAMTDFMYLAGLPMVSDAVPFLGWLDGVN
GYKGEMKRTAKEIDYVIDAWVKEHKQKLIGGKIDEPEQDFIHVMLSVIKD
DDLFAGIDANTTIKSTCLSLILGGSDTTVVTLTWALALLLNNRRVLKKAQ
EELDVHVGRDRQVDESDIKNLPYLQSVVKETFRLYPAVPLSVPREAIEDC
TIAGFSIPAGTRLWVNLWKLQRDPTIWSDPLEFQPERFCGKHADVDIKGQ
DYELIPFGSGRRSCPGTQLAMRVLQLTLARLLHGFELGTVSDSIVDMTER
TGLTVPKATPLEVTLTPRLSSTLYNC
>NjCYP82C4
MEFYIHLHQITLYGLIFAIMCAWIIIINPIRNKKKPRGPPEPGRAWPFIG
HLLLLKPNDLLHRKLGAMADEYGPIFSMKLGAHQTLVISSSQLAKECFTV
HDRVFPNRPKNLAVKLMGYNHAVLGFAPYGPYWRDVRKLAVVELLSGRRL
DMLKHVWHTEIDFFMKMLYDQWVETGGRGPVLVDMKELFGDMAMNIVVRM
VAGKRYFGKGTLFDEESKRCQKAMTDFMYLAGLPMVSDAVPFLGWLDGVN
GYKGEMKRTAKEIDYVIDGWVKEHKQKLIGGKIDEPEQDFIHVMLSVIKD
DDLFAGIDANTTIKSTCLSLILGGSDTTVVTLTWALALLLNNRRVLKKAQ
EELDVHVGRDRQVDESDIKNLPYLQSVVKETFRLYPAVPLSVPREAIEDC
TIAGFSIPAGTRLWVNLWKLQRDPTIWSDPLEFQPERFCGKHADVDIKGQ
DYELIPFGSGRRSCPGTQLAMRVLQLTLARLLHGFELGTVSDSIVDMTER
TGLTVPKATPLEVTLTPRLSSTLYNC
>NjCYP82D44
MYLSSSTIAATIFSSIVFLAFLRHAFFKKKATRNPPQAKGAWPIIGHLHL
LGGRELPHNILGDMAQEHGPIFTIMLGVHKALVVSDWVITKECFTTNDKL
FASRPKSEATKIMGYDYAMFGLAPYGEYWRQMRKMVTREVLSQRHVDMLE
PIQASELRASIKDLYDVWVKNKKSENATMVRVDMGEWFGKLVVNIMVRVI
SGKRFLPNDGEGIRVHRIIKKFFEIMGAFVASDFIPYFRFFDVGGYKKAM
KKTARDLDNIFEGWLKEHKMDIKHEGNQDFINVLMSILQGASKDKFLGFD
HDTIIKSTCQNLLVAGVDTTSLTLTWALALLLNNPKSLQIAQDEIDEHVG
KERLVEESDTKNLVYLDAIIKETFRLYPAGPLSVPHESLNDCIVGGYNIP
KGTRLLVNLYKMQRDPNIWPDPHEFRPERFLTSHKSINVKGNHYELLPFG
SGRRICPGISFALQELSLTLASLIQQFVLKKPSNKPVDMTESMGLTIHKA
TPLDVLLAPRLSSNMYHVSS
>NjCYP82D45

MDILVRFTIASILIFLSLYWVSKITKRSKKTPPPPPQASGAWPIIGHLHR
LGGPQVLHRILASMADKHGPIFTFNLGVHKVLVVSDSNLAKECYTTNDKA
FANRPKSIAVELMAYNNALLGLNPYGPYWRKMRKIVVNDILSKRHMPMLE
QIGVSEIRASMKETYKFWESSNRRNEVAKAEMKEWFGRIVLNMVLKMLIG
KRYSSNDERVKSTKVLKNYTVLLGTFVVGDAIPWLRWLDIGGHEKAMKTV
FMEIDFLLQQALEEHKRKRSLIVSGEIEGDINEEKDFMDMMLSILDGVSH
EELCGFDPDVVVKATCLTLFIGGVDTTMVTLTWALSLLLNNKHMLKMVEE
ELDLHVGKERQVEESDLKNLVYLQAVFKETLRLYPAGPLALPHESMEDCI
VGGYHIPKGTRLLVNLFKIHTDPRIWPNPSEFQPERFLTTQKHIDFRGQN
YELIPFGSGRRVCPGISLAMQNMQLILGSLIHGFEFTTILDEPIDMSEGF
GLSNNKAKSLEVLLKPRLPPYLYE
>NjCYP82D46
MDNLARFTVASILIFLSLYWISKITKRSKKSPPPPQVSGAWPIIGHLHLL
GGPQVLHRTLASMADKHGPIFTFNLGIRKVLVVSDSKLAKECYTTNDKAF
ANRPKSIAVELMCYNYATHGLEPYGPYWRKIKKIVVNDILSKSHMATLER
IGVSEIKASMKETYKFWESSNRRNEVAKAEMKEWFGRIVLNMVLKMVIGK
RFSSNDEQVKYQKVFKEFTVLLGTFVVGDAIPWLRWLDIGRHEKAMKTIF
MEIDFLLQEALEEHKRKRSLVVSGEIEGDINEEKDYMDMMLSILDGVSHE
ELCGFDPDVVTKATCLTLFQGALHTTIVTLTWAISLLLNNKRALNLVEKE
LDLHVGKERHVKESDLKNLVYLQAVFKETLRLYPAGPLSLPHESMEDCIL
GGYYIPKGTRLLFNLFKIHSDPRIWPNPSEFQPERFLTTHKHIDFRGQNY
ELISFGSGRRVCPGISLAMQNMHLILGSLIHGFELTTILDEPIDMSDGFG
ITNNKATSTEVLLKPRLPPYLYE
>NjCYP82D47
MDILVRFTIASILFFLSLFWVSKKTKRSKTTPPPPQASGAWPIIGHLHLL
GGPQVLYRILASMADKHGPIFTFNLGVHKVLVISDSNLAKECYTTNDKAF
ANRPKSIGGELMGYNCTMLGLEPYGPHWRKVKKIVVNDILSKRHMPMLER
IGVSEIRASMKETYKFWESSNRRNEVAKAEMKEWFGRIVLNMVLKMVIGK
RFSSNDERVKYAKVVKDFTLLLGTFVVGDAIPWLRWLDIGGHEKAMKTIF
KEIDFLLQQALDEHKRKGDNINEEKDFMDMMLSNLDGVSQEEHCGLDPDV
VVKANCLTLFLGAIDTTMVTLTWALSLLLNNKHTLNMVKEELDLHVGKER
QVEESDLKNLVYLQAVIKETMRLYPAGPLSLPHESMEDCIVGGYHIHKGT
RLLFNLFKIHSDPRIWPNPSEFQPERFLTTHKHIDFRGQNYELLPFGSGR
RVCPGISLAMQNLHLILGSLIHGFEITTILDEPIDMSETFGLANNKATSI
EVLLTPRLPPYLYE
>NjCYP82G1
MEFYFHPLVLGILLAFLFAFLFRKKDKNSIPEPSGAWPIIGHLHLLWGKI
PVARTLGAMADKYGPIFSLQLGNRRTVVVSSWEVVKECLTTNDKTFASRP
SMAAFKYLGYGYASFPVAPYGPYWREVRKMVTLTLFTNNRLENLKHVRAA
EVNRGVKDLHLRSPKNVAINKWFDDLTLNMTIRTLVGKRLNNEGDLRFKE
AVKKALFLSGVFVVSDVIPGLEWLDIGGYLKSMKQTFKELDLVISKWLEE
HMHKRKLYGTDGDADFMDVMISGLATDPIVSDHTHDIIIKSTTLALVLTG
SESMADTLTWALSLLLNSLDALKTVQDELDTNVGRNKWVEESDIKNLTYL
QVVVKETLRLYPPGPLAGPHEATEGCYISGYNIPKGTRLIVNLLKLHRDP

RVWSDPNEFRPERFLKGHAHVNYSGQNFEYIPFSSGRRMCPATMYGLQVI
HLILASLLQGFHVSTSHGMSVDMSEGLGIVMPKVEPLKVILTPRLPLQLY
Q
>NjCYP82G2
MSYAIHIENHIKEKIIMEFYFHPLVLGILAFLFAFLFWKKNKNSIPEPSG
AWPIIGHLHLLWGKIPVARTLGAMADKYGPIFSLQLGNRRVVVVSSWEVV
KECLTTNDKTFASRPSMAALKYLGYGYASFPVAPYGPYWREVRKMVTLTL
FTNNRLKNLKHVRAAEVNRGVKDLHLRSPKNVAINKWFDDLTLNMTIQTL
VGKRLNNEGDLRFKEAVKKALFLSGVFVVSDVIPGLEWLDIGGYLKSMKQ
TFKELDLVISKWLEEHMHKRKLYGTDGDADFMDVMISGLATNPIVSDHTH
DIIKSTTLALVLTGSESMADTLTWALSLLLNNPDALKTVQDELDTNVGT
NKWVDESDIKNLTYLQAIVKETLRLYPPGPLAGPHEATEGCYINGYNIPK
GTRLIVNLLKLHRDPRVWSDPNEFRPERFLKEHAHVNYSGQNFEYIPFSS
GRRMCPATMYGLQVIHLILASLLQGFHVSTSHGMSVDMSEGLGIVMPKVE
PLKVILTPRLPLQLYQ
>NjCYP84A1
MEVDYLIEALQSMPMTLYFIIPLILFFSISRYRRKLPYPPGPRGWPVIGN
MDMIDKLTHRGLAKLAAEYGGLCHFRIGLLHMVAVSSPDAARQVLQIQDN
IFSNRPATIAIRYLTYDRADMAFAHYGPFWRQMRKICVMKVFSRKRAESW
DSVRDEVDAMVKTVTTASNTRSPINIGDLVFGLTRDIIYRAAFGSISHQG
QDEFIKIMQEFSKLFGAFNIADFFPSLGWVDPQGFNSRIAKARASLDGFI
DSILDEHLEKKKTGFGSDATDSDMVDELLVFYSDHEAKMNESAEFQNSIK
FTRDNIKAIIMDVMFGGTETVASAIEWVMAELMRSPEDLKRVQQELTDVV
GLHRRVEESDFEKLTYLRCCLKETLRLHPPIPLLLHETAEEAEIAGYRIP
ARSRVMINAWAIGRDKNSWVDPDTFKPSRFLDEGAPDFKGSNFEFIPFGS
GRRSCPGMQLGLYALEVAVAHLLQCFTWDLPDGMKPSELDMGDVFGLTAP
RASRLVAVPSKRVLCPLC
>NjCYP84A3
MNSLPQEYLHITFLFIVPLLFLFILSRFRRRLPYPPGPKGWPVIGNMNMV
NQLTHRGLAKLAKQYGGIFHLRIGFLHMVAVSSPEIARQVLQVQDNIFSN
RPATIAISYLTYDRADMAFAHYGPFWRQMRKLCVMKLFSRKRAESWDSVR
DEVDSMVHLVAQNTSSPVNIGELVFGLTKDIIYRAAFGTSTKDGQDEFIK
ILQEFSKLFGAFNISDFFPSIGWADPQGLNKRLTKARSALDGFIDSIIDD
HMQKKKPEDADTDMVDDLLAFYSDEATVNESEDLKDSIKLTRDNIKAIIM
DVMFGGTETVASAIEWAMTELMRSPDELKRVQQELADVIGLNRRVEESDF
DKLTYLKCCLKETLRLHPPIPLLLHETAEEAVVAGYRIPARSRVMINAWA
IGRDNNSWEDPETFKPSRFLDDGVPDFKGSNFEFIPFGSGRRSCPGMQLG
LYALEISVAHLLHCFTWELPDGMKPSQLDMSDVFGLTAPRASRLVAIPTP
RLLCPL
>NjCYP84A5
MDPISILLYVALPLLTFFLLSRLRRKPLPPGPRGWPLIGNMLMMDQLTHR
GLASLGEKYGGLLHLKMGFSHTVAVCSPEIARQVLQVQDNIFSNRPATIA
ISYLTYDRQDMAFANYGPFWRQMRKLCVMKLFSRKRAESWDSVRDEVVSM
IKTTAASSGTAVNLGELVFGLTHDIIYRAAFGSISHEGKEEFIRILQEYT

KLFGAFNLADFVPWLGFIDPAGLNTRLPKARAALDGFIDKIIDEHLTKEK
KTGDDADNDMVDEMLAFYSEEGKLNEGEDLQNAIRLTRNNIKAIIMDVMF
GGTETVASAIEWALTELMHTPESLKRAQQELADVVGLERRVEESDFEKLT
YFKCIIKETLRLHPPIPVLLHQSSEATEVAGYHIPKGTRVMVNAFAINRD
KNAWKDPHTFNPSRFLEEGAPDFKGSNYEFLPFGSGRRSCPGMQLGLYAM
EMAVAHLLHSFTWQLPDGMKPSEIDMSDVFGLTAPKAIRLVAVPTPRLLC
PLY
>NjCYP84A6
MDYLQIPIPIYAIIAILTLFFLAWIRRKPLPPGPMGWPIIGNMLMMDQLT
HRGLAALAEKYGGILHLKMGFGHTIAVSSPEIARQILQEKDNIFANRPAT
IAITYLTYDRVDMAFADYGPFWRQMRKLCVMKLFSRKRAESWDSVRDEVD
TMVKETAINSGLAVNLGELVFGLTHDIIYRAAFGSISHEGKEEFIRILQE
YTKLFGAFNLADFIPFLGFIDPAGLNTRLPAARAALDGFIDKIIDEHLSK
EKKFGDENVDNDMVDEMLAFYSEDGKINEGGDLQNAINLTRDNIKAIIMD
VMFGGTETVASAIEWTMTELMHTPEALKRVQQELTNVVGLDRRVEESDFE
KLTYFKCVIKETLRMHPPIPVLLHQSSEATEVAGYHIPKGTRVMVNAYAI
NRDKNAWEDPHTFNPSRFLENGAPDFRGSNYEFLPFGSGRRSCPGMQLGL
YAMEMAVAHLLHCFTWELPDGMKPSEIDMGDVFGLTAPKAIRLVAVPTPR
LLCPLY
>NjCYP85A1
MAFFILVILVILGLCLFSTALLRWNEVRYRKKGLPPGTMGWPLFGETTEF
LKQGPSFMKNQRARYGSVFKSHILGCPTIVSMDPELNRYILMNEGKGLVP
GYPQSMLDILGKCNIAAVNGSTHKHMRGALLSLVSPTIIREQLLPKIDHF
MRFHLTNWDNKVIDIQQKTKEMALLSALKQIAGIESGTMSQEFMPEFFKL
VLGTLSLPINLPGTDYHRGFQARKNITSMLRQLIEERRASQEKQKDMLGY
LMNGEESRYKLSDEEIIDQIITILYSGYETVSTTSMMAVKYLHDHPNVLE
ELRKEHMAIRRTKKPDDPINWNDYKEMRFTRAVIFETSRLATIVNGVLRK
TTRELELNGLVIPEGWRIYVYTREVNYDPCVYPDPLTFNPWRWLDKSMET
QNYFFIFGGGTRQCPGKELGIAEISTFLHYFVTRYRWDEVGGDKLMKFPR
VEAPNGLHIRVSTH
>NjCYP86A1
MDTIFLTFAITTATSAYLLWFYLLSRHLTGPKAWPLVGSLPSLWINRSRF
HDWMASNLRSTGSSATYQTTTIAVPFLARKQGFYTVTCHPKNIEHILRTR
FDNYPKGPTWQGAFHDLLGQGIFNSDGDTWLIQRKTAALEFTTRTLRQAM
ARWVSRTIKMRLWPILETAAKEKAAVDLQDILLRLTFDNICGLTFGKDPE
TLSPGLPENPFANAFDFATEATLQRLLYPGFLWRLKKLLGIGAEFKLSKS
LEIVEDYMTEALTARKLTPSDDLLSRFMKKRDVDGNLFPSHVLKRIALNF
VLAGRDTSSVALSWFFSLVMNNPDVESKIVGEISTVLKETRGEDRRKWIE
DPLVFDEADKLVYLKAALAETLRLYPSVPEDFKYVINDDVLPDGTKVPAG
STVTYSIYSVGRMKSVWGEDCMEFKPERWLSTGGDRFEPPKDGYKFVAFN
GGPRTCLGKDLAYLQMKSIVSAVLLRYRISLVPGHRVEQKMSLTLFMKNG
LKVYLHPRELGATCA
>NjCYP86A2
MIFRESTFLRKFSAIADMEYVSTALLLFTSITVYMMWFTFISRSLKGPRV

WPLLGSLPGLIENSDRLHDWISDNLRTCGGTYQTCICAIPFLAKKQGHVT
VTCDPKNIEHVLKTRFDNYPKGPTWQGAFHDLLGQGIFNSDGDTWLFQRK
TAALEFTTRTLRQAMARWVNRAINLRFCPILEAAQASAEPVDLQDLLLRL
TFDNICGLAFGKDPQTLAPGLPANGFALAFDRATEATLQRFILPEIVWKL
RKWLRLGMEPELSRSVIHVDDYLSSVISTRKHELMSQHKDDNPHDDLLSR
FMKKKQSYTDKYLKHVALSFILAGRDTSSVALCWFFWSVIQNPTVEHKIL
HEICTVLIETRGDDVASWINEPLDFEEVDRLIYLKAALSETLRLYPSVPE
DSKHVVADDVFPDGTFVPAGSSVTYSIYSVGRMKTVWGDDCLDFRPERWL
SPDGKQFVMHDSYRFVAFNAGPRICLGKDLAYLQMKSVAASVLMHHRLTV
AAGHKVEQKISLTLFMKYGLKVEVHGRDLAAVLESVKRENEMLEGKVKM
>NjCYP86A3
MDISTAFILFTAITAYLLWFTFISRLLKGPRVWPLLGSLPGLIDNSDRLH
DWIADNLRACGGTYQTCICAFPFLARKQGLVTVTCDPKNLEHILKTRFDN
YPKGPTWQAVFHDLLGKGIFNSDGDTWLFQRKTAALEFTTRTLRQAMARW
VSRAIKLRFCPILEAAQLKAEPVDLQDLLLRITFDNICGLAFGQDPQTLA
PGLPDNSFSSAFDRATEASLQRFIFPEVIWKLKKWLRLGMEVSLSRSLVH
VDKYLSSVIEARKVELLNFQKGGNPHDDLLSRFMKKKECYSDKFLQQVAL
NFILAGRDTSSVALSWFFWLVTQNPLVEEKILRELCSVLVETRGDDVATW
VDEPLGFEEVDRLTYLKAALTETLRLYPSVPEDSKHAVADDVLPDGTFVP
AGSSVTYSIYSAGRMKSTWGEDCLEYKPERWLSPDGTRFEAKDSFKFVAF
NAGPRICLGKDLAYLQMKSIAAAVLLRHRLTVVPGHQVEQKMSLTLFMKY
GLKVEVHRRDLAAVLSSFKREKDLWLLV
>NjCYP86A4
MDSSTVMMILAIVAAYLLWFRFITRPLRGPRVWPLLGSLPGLIDNSNRMH
EWIADNLRACGGTYQTCISAVPFLARKQGLVTVTCDPKNLEHILKVKFDN
YPKGPTWQAVFHDLLGEGIFNSDGDTWLFQRKTAALEFTTRTLRQAMARW
VSRAIKMRFCPILKTAQLEEKPVDLQDLLLRLTFDNICGLAFGKDPHTLS
PGLPDNSFASAFDRATEATLQRFILPEIMWKLKKWLRLGMEVNLSHSLEH
VDEYMTNVINTRKLELLSQQKGSTPHDDLLSRFMKKKESYTDKFLQQVAL
NFILAGRDTSSVALSWFFWLVTENPRVEQKILTEICTVLMETRGTDTSKW
LEDPLVFEEVDRLIYLKAALSETLRLYPSVPEDSKHVIADDILPDGTFVP
AGSSITYSIYSAGRMKFIWGEDSLEFRPERWLTEDGKKFEVSDSYKFVAF
NAGPRICLGKDLAYLQMKSIAAAVLLHHRLTLAPGHRVEQKMSLTLFMKY
GLKVNVHQRDLTPILVNIGQ
>NjCYP86B2
MTNLTNSTSYFLSGDIAGSTSLRQGLFSLPEIQILELCVSVFVFIAIHSL
RQKKHHGLPVWPLVGMLPSLVSAVRVDMYEWLSGILCSRNGTFIFKGPSF
TNLNCVVTSDPPNLEHILKSKFSIYPKGEYFRNSLRDLLGNGIFNADDDV
WQKQRKTASIEFHSAKFRNMTTESLFDLVHARLLPVLENSINESTTIDLQ
DILLRLTFDNVCIIAFGVDPGCLSPGLPEIPFAQSFEAATEAAILRFVMP
TFIWKAMRYLNLGSEKKLKESIEGVDLFANEVIRKRKEDILLESEDNKLR
SDLLTIFVRLKDEKGDAFSDQFLRDICVNFILAGRDTSSVALSWFFWLIG
RNPAVEERILEEMYRIVGERGDSDKKDSDGEFVFKAEEVKKMDYLQAALS
ESLRLYPSVPVDHKEVVEDDVFPDGTVLKKGTKVVYAIYAMGRMEAIWGK

DCREFKPDRWLRNGRYMSESAYKFTAFNGGPRLCLGKDFAYYQMKFVAAS
ILYRYHVKVVKNHPVRPKLALTMYMKHGLKVKLCRRDESEIQK
>NjCYP87A1
MWGVEIYIIVGLITICISYLVQKWRNSNKCINGGILPPGSVGFPLLGETL
HLLIPGRSLDLHPFLKNRFRRYGPIFRTNLVGQPVVISADPEFNHKLLLH
EGRLVELWYMDSFSKLFNQDGKSSTTEFGSVHRYIRSIILNHFGPEPLKQ
KLLAQIHVMVDKTLHSWSTQQSIEVKHAVSSIVFDFTAKRMVGYSPETSS
EKIKSEKLAHLVKGLMSFPINIPGTEFHRCMKVQKMVTNMIKKKIKEKQN
NLFTTLEGDLLDQAVKDMETEDFLTEEFLVPFLFGAIFASFESISSALTL
TFELLSEHPAVIEELIAEHDRVLKNRGENLKSEITWEEYKSMTFTHNVVS
EALRMSNVAPGFLRRALKDIQVNGYTIPAGWTIMVASSAQQLNPNTYKDP
LQFNPWRWKDMDSNVVSKNFLPFGGGMRQCAGAEFSKAFMATFLHVVITK
FRWTKIAGGEIGRTPFLDFGKGIHIKVEKRN
>NjCYP87A2
MWGVEIYIIVGLITICISYLVQKWRNSNKCINGGILPPGSVGFPLLGETL
HLLIPGRSLDLHPFLKNRFRRYGPIFRTNLVGQPVVISADPEFNHKLLLH
EGRLVELWYMDSFSKLFNQDGKSSTTEFGSVHRYIRSIILNHFGPEPLKQ
KLLAQIHVMVDKTLHSWSTQQSIEVKHAVSSIVFDFTAKRMVGYSPETSS
EKIKSEKLAHLVKGLMSFPINIPGTEFHRCMKVQKMVTNMIKKKIKEKQN
NLFTTLEGDLLDQAVKDMETEDFLTEEFLVPFLFGAIFASFESISSALTL
TFELLSEHPAVVEELIVEHDRVLKNRGENPKSEITWEEYKSMTFTHNVVS
EALRMSNVAPGFLRRALKDIQVNGYTIPAGWTIMVASSAQQLNPNTYKDP
LQFNPWRWKDMDSNVVSKNFLPFGGGMRQCAGAEFSKAFMATFLHVVITK
FRWTKIAGGEIGRTPFLDFGKGIHIKVEKRN
>NjCYP87A3
MWGVEIYIIVGLITIYISYLVQKWRNSNKCINGGILPPGSVGFPLLGETL
HLLIPGRSLDLHPFLKNRFRRYGPIFRTNLVGQPVVISADPEFNHKLFLH
EGRLVELWYMDSFSKLFNQDGKSSTTEFGSVHRYIRSIILNHFGPEPLKQ
KLLAQIHVMVDKTLHSWSTQQSIEVKHAVSSFVFDFTAKRMVGYSPETSS
EKIKSEKLADLVKGLMSFPINIPGTEFHRCMKVQKMVTNMIKKKIKEKQN
NLFTTLEGDLLDQAVKDMETEDFLTEEFLVPFLFGAIFASFESISSALTL
TFELLSEHPAVIEELIAEHDRVLKNRGENLKSEITWEEYKSMTFTHNVVS
EALRMSNVAPGFLRRALKDIQVNGYTIPAGWTIMVASSAQQLNPNTYKDP
LQFNPWRWKDMDSNVVSKNFLPFGGGMRQCAGAEFSKAFMATFLHVVITK
FRWTKIAGGEIGRTPFLDFGKGIHIKVEKRN
>NjCYP87B1
MYFTKMWSVCLVTGVVCVLISIIYWVYFWNNPKCNGKLPPGSMGWPLLGE
TLPFFAPNTTNDISPFVKKRMKRYGPIFRSSLVGRPVIISTDSNLNHLIF
QQEGELFQSWYPDTFTKILGEKNVSTLHGFMYKYLKNMVLNLFGPESLKR
MIAQVEETAKKNLKMWSDSESVELKEATSSMIFHLTAKKLIGYDSGKPSD
NLRKCFVDFIEGLISFPLNIPGTAYNKCLKGRKKAMRLLKNLLEERRANP
KKIQTDFFDYVLEELEKKDTVLTEEIALDLMFALLFASFETTSSALTLAT
KFLSDNLLVLKELTDEHEKILLQRNNLESGLSWNEYKSMTFTFKFINETV
RLANIVPVIFRKSLKDVKFKDYTIPANWTVLVCPPAVHLNPNRYENPLEF

NPWRWDGMESISASKHFMAFGGGMRFCVGADFSKLQMAVLLHCLVTKYKW
QSIRGGEILRTPGLQFPNGFHVRFTEKNKEENTLM
>NjCYP88A1
MDVNNNITTEMWVAVVVLGVVPLAGWLLWWWNDIWYCLPVKMRWWSSGGT
AKLPPGHMGIPFFGEMINFLWYFKVVRRPDDFINSKRRKYGDGEGIYRTH
LFGSPAIIACSPLLNKFIFKSESDFILEWPAVEIVGTSSLVAVQGAAHAR
LRNYVSRAINQPEALRKISLMVQPRIIAALHSWAQTGRVSAHEQAKKVTF
ENIGKFFANFEPGSKLDLLDDLFTGLMHGVRSYPLDFPGTSYHYALKCRE
KAVAIFAEELEKRRKEKEDGGLDLLSGLMKLKDDEGKLLSDKEVLDNIVS
FVVAGYESTSLAIMWSFYYLAKYPKVLQKLRDENIPFSKSKNGELISSDD
ISKLKYTNKVVEETIRMANIAAVVFRTATRDVEYKGFKIPKGWKVLLWIR
YLHTNSENFADPMCFNPDRWNEPAKPGTFQVFGGGSRICAGNMLARIQVA
IFLHYLSTGYKWELVNPDADMVYLSHPKPVDGVEILINKL
>NjCYP88B1
MGLIWIICGGLIISVLWVLKNVNLWVYESKLGDNKRFNLPPGDLGWPFIG
HMWSFLRAFKSNNPDSFISSFITRFGKTGIYKTFMFGNPSIIVTTAEACR
KVLSDDEAFRPGWPKSTMKLIGKKSFIGISYDEHKRLRRLTSAPVNGHEA
LSLYMQYIEEIVVSTLDKWANMGEIEFLTELRRLTFRIIMYIFLSSESEQ
VMDALEREYTTLNYGVRAMAINIPGFAYHKALKARKKLEAIFQTIVDERR
DRKSRNEPKPKKDMMDALLDVEDENGRKLNDEEIIDLLIMYLNAGHESSG
HITMWATLFLHRHPDIFKKAKDEQETIVKNRSPTQKGLTLKEVRQMEYLS
KVIDETLRLVTFSFVVFREAKKDFRLNGYTIPQGWKVLVWFRTIHLDSEI
YPDPKEFNPSRWDDLVPKAGTFLPFGAGSRLCPGNDLAKLEISIFLHYFL
LNYKLEQTNPGCPTMYLPHCRPKDNCLARLRRVSSPSL
>NjCYP88B2
MGFDELGGVSYYVGILVGFMLILKWVLKSVNVWIYERNLGKKKRECLPPG
DMGWPFIGNMWSFLRAFKSSNPDAFISTLLLRYGSKGLYKSFMFGSPSII
VTIPEACRKVLYDDDAFQPGWPTSTMELIGKKSFIGISYEEHKRLRKLTA
APVNGHEALTTYMRYIETNVVSTLEEWSKMGRIEFLTKLRKLTFQIIMHI
FLSSEGENVMEALEKEYTALNYGVRAMAINIPGFAYYNALKARKKLVAIL
QKIVQERRKTRETTEGMSKKDMLDALLDSADDKGRKLDDEEIIDTLVMYL
NAGHESSGHITMWSTILLQEHPECLRIAKEEQERIVKNMPSTQKALTLKE
YRQMEYLSKVIDETLRLVTFSLMTFREAKKDVDITGYFIPKGWKVLLWFR
SVHHDPEIYPQPKEFNPSRWDDIVPKPGTFLPFGAGSRLCPGNDLAKLEI
AIFLHHFLLNYEFERENPKCRIMYLPHSRPKDNCLGRIRRVSK
>NjCYP89A1
METWFVIILSLCIAAIIKPLFFTNNKKKLPPRPLTVPLIGNLLWLRKSFA
EIPEIELILREFKLKYGPLISLPLGSRPAIFIGNHTLAHRALVQNGAVFS
DRPKPLPTTNQHNISSASYGATWRILRRNLTSEILHPSRVKSYTSARLWV
LDVLIRRLREDSAAGAAVKVVDHLQYAMFCLLVLMCFGDKLDDDKITKIK
TVQRRLLLSFGRFNVLNIFPRLGKIIFRRRWKQFMELREEQERVLILLIE
TRIQYKQQKHDDDDKDHVVAYVDTLLDLQLTEGEEKRKLSKEEIVSLCSE
FLNAGTDTTSTALQWILANLVKYPQIQSKLYQEIAGVMNGPSPPLPSGET
GSISEVVKEEDVQKMPYLKSVVLEGLRRHPPGHFVLPHSVSEEVEFEGYV

VPKNATINFMVADMGWDPEVWEDPMEFKPERFSGEEGGGFDITGSKEIKM
MPFGAGRRICPGLGLAMLHLEYFVANLIWYLEWKGVDGEEVDLSEKQEFT
MVMKNPLKAHISSR
>NjCYP89A2
METWFVIILSLCIAAIIKPLFFTNNKKKLPPRPLTVPLIGNLLWLRKSFA
EIPEIELILREFKLKYGPLISLPLGSRPAIFIGNHTLAHRALVQNGAVFS
DRPKPLPTTNQHNISSASYGATWRILRRNLTSEILHPSRVKSYTSARLWV
LDVLIRRLREDSAAGAAVKVVDHLQYAMFCLLVLMCFGDKLDDDKITKIK
TVQRRLLLSFGRFNVLNIFPRLGKIIFRRRWKQFMELREEQERVLIPLIE
TRIQYKQQKHDDDDDDHVVAYVDTLLDLQFTEGEEKRKLSKEEIVSLCSE
FLNAGTDTTSTALQWILANLVKYPQIQSKLYQEIAGVMNGPSPPLPSGET
GSISEVVKEEDVQKMPYLKSVVLEGLRRHPPGHFVLPHSVSEEVELEGYV
VPKNATINFMVADMGWDPEVWEDPMEFKPERFSGEGGGFDITGSKEIKMM
PFGAGRRICPGLGLAMLHLEYFVANLIWYLEWKGVDGEEVDLSEKQEFTM
VMKNPLKAHISPR
>NjCYP89A4
METWFIIILSLCIAAIIKPLFFTNNKKKLPPGPLTVPLIGNLLWLRKSFA
EIEPLLREFKLKYGPLISLTIGSRPAIFIGNHTLAHRALVQNGAVFSDRP
KPPPTTKIMSSNQHNISSASYGATWRILRRNLTSEILHPSRVKSYTSARL
WVLDVLIRRLREDSAAGAAVKVVNHLQYAMFCLLVLMCFGDKLDDDKITK
IETVQRRLLLSFGRFNVLNIFPRLGKIIFRRRWKQFMELREEQERVLIPL
IETRIQFKQQKHDDDKDHVVAYVDTLLDLQLTEGEEKRKLSKEEIVSLCS
EFLNAGTDTTSTALQWILANLVKYPQIQSKLYQEIAGVMNGPSPPLPSGE
TGSISEVVKEEDVQKMPYLKSVVLEGLRRHPPGHFVLPHSVSEEVELEGY
VVPKNATINFMVADMGWDPEVWEDPMEFKPERFSGEGFDITGSKEIKMMP
FGAGRRICPGLGLAMLHLEYFVANLIWYLEWKGVDGEEVDLSEKQEFTMV
MKNPLKAHISPR
>NjCYP89A5
METWFIIILSLCIAAIIKPLFFTNNKKKLPPGPLTVPLIGNLLWLRKSFA
EIEPLLREFKLKYGPLISLTIGSRPAIFIGNHTLAHRALVQNGAVFSDRP
KPPPTTKIMSSNQHNISSASYGATWRILRRNLTSEILHPSRVKSYTSARL
WVLDVLIRRLREDSAAGAAVKVVDHLQYAMFCLLVLMCFGDKLDDDKITK
IETVQRRLLLSFGRFNVLNIFPRLGKIIFRRRWKQFMELREEQERVLIPL
IETRIQYKQQKHDDDDDDHVVAYVDTLLDLQLTEKRKLSKEEIVSLCSEF
LNAGTDTTSTALQWILANLVKYPQIQSKLYQEIAGVMNGPSPPLPSGETG
SISEVVKEEDVQKMPYLKSVVLEGLRTHPPGHFVLPHSVSEEVELEGYVV
PKNATINFMVADMGWDPEVWEDPMEFKPERFSGEGGGFDITGSKEIKMMP
FGAGRRICPGLGLAMLHLEYFVANLIWYLEWKGVDGEEVDLSEKQEFTMV
MKNPLKAHISPR
>NjCYP89A6
METWFIIILSLCIAAIIKPLFFTNNKKKLPPGPLTVPLIGNLLWLRKSFA
EIEPLLREFKLKYGPLISLTIGSRPAIFIGNHTLAHRALVQNGAVFSDRP
KPPPTTKIMSSNQHNISSASYGATWRILRRNLTSEILHPSRVKSYTSARL
WVLDVLIRRLREDSAAGAAVKVVDHLQYAMFCLLVLMCFGDKLDDDKITK

IETVQRRLLLSFGRFNVLNIFPRLGKIIFRRRWKQFMELREEQERVLIPL
IETRIQYKQQKHDDDDDDHVVAYVDTLLDLQLTEGEEKRKLSKEEIVSLC
SEFLNAGTDTTSTALQWILANLVKYPQIQSKLYQEIAGVMNGPSPPLPSG
ETGSISEVVKEEDVQKMPYLKSVVLEGLRRHPPGHFVLPHSVSEEVELEG
YVVPKNATINFMVADMGWDPEVWEDPMEFKPERFSGEGFDITGSKEIKMM
PFGAGRRICPGLGLAMLHLEYFVANLIWYLEWKGVDGEEVDLSEKQEFTM
VMKNPLKAHISPR
>NjCYP89A7
METWFIIILSLCIAAIIKPLFFTNNKKKLPPGPLTVPLIGNLLWLRKSFA
EIEPLLREFKLKYGPLISLTIGSRPAIFIGNHTLAHRALVQNGAVFSDRP
KPPPTTKIMSSNQHNISSASYGATWRILRRNLTSEILHPSRVKSYTSARL
WVLDVLIRRLREDSAAGAAVKVVDHLQYAMFCLLVLMCFGDKLDDDKITK
IETVQRRLLLSFGRFNVLNIFPRLGKIIFRRRWKQFMELREEQERVLIPL
IETRIQYKQQKHDDDDDDHVVAYVDTLLDLQLTEGEEKRKLSKEEIVSLC
SEFLNAGTDTTSTALQWILANLVKYPQIQSKLYQEIAGVMNGPSPPLPSG
ETGSISEVVKEEDVQKMPYLKSVVLEGLRRHPPGHFVLPHSVSEEVELEG
YVVPKNATINFMVADMGWDPEVWEDPMEFKPERFSGEGGGFDITGSKEIK
MMPFGAGRRICPGLGLAMLHLEYFVANLIWYLEWKGVDGEEVDLSEKQEF
TMVMKNPLKAHISPR
>NjCYP90A1
MDSFTSLLVLLSTSFSLLIYLLYTNHFRRRKLRLPPGNLGLPFLGETLQL
ISAYKTENPEPFIDHRVARYGSVFTTHVFGEPTVFSADPDTNRFILQNEG
RLFESSYPDSISNLLGRHSLLLMRGTLHKKMHSLTMSFANSSIIKDHLLV
DIDRLVRLNLETWTGRVLLMEEAKKITFQLTVKQLMSFDPCEWTENLMKE
YMLVIEGFFSIPLPLFSITYRRAIQARRKVAEELILVVRERRRESERGER
KNDMLEALMDGGEGDGDGGGFSNEEIVDFLLALLVAGYETTSTIMTLAVK
FLTETPLALAQLKEEHDEIRGKKGEKEGLEWEDYKSMPFTQCVVNETLRV
ANIISGVFRRAMTDVNIKGYTIPKGWKVFASLRAVHLDHNHFKDARSFNP
WRWQSNSAATSSVNMFTPFGGGPRRCPGSELARVELAVFLHHLVTRFSWI
PAEEDKLVFFPTTRTQKRYPILVERRVKSM
>NjCYP90A3
MITMELFTLSLLLTVSLSTIIFFLFRTTTRKLRLPPGSIGLPFIGETFQL
ISAYKTENPEPFIDTRVSKYGSVFTTHVFGERTVFSADAETNRFILQNEG
RLFESSYPGSIANLVGKHSLLLMRGSLHRRMHSLTMSFANSTIIKDHLLV
DIDRLVRLNLDSWTGRILLMEEAKKITFELTLKQLLSIDPCEWSENLRKE
YMLVIEGFFCIPLPFFSLTYRRAIQARERVTEALNLVVGERRKERERGVK
KKDMLAALFDSAGDDAVFHDDEIVDFLVSLLVAGYDTTSTTMTLAVKFLT
DTPSALSQLQEEHDEIKARRGTSVALEWEDYKSMPFTQCVVNETLRLSNI
ISGVFRRAMTDVKIKGYTIPKGSKVFTSLRAVHLGQENFKDARVFNPWRW
QNTSDPTNFMPFGGGPRRCPGYELARVELSVFLHHLVTRFNWKAAEEDKL
VFFPTTRTQKRYPMVVEHRNLGG
>NjCYP90B1
MADLDFLIFIFSSIFTLLLIFNLITKHRKISLNLPPGNSGWPFIGETIAY
LKPYSAISIGDFMEQHISRYGKIYKSNLFGEPTIVSADAGLNRFILQNEG

RLFECSYPTSIGGILGKWSMLVLVGDMHRDMRIISLNFLSNARLRTHLLP
EVEKHTLLVLNSWKHNSTFCAQDEAKKFTFNLMAKHIMSLDPGKPETEQL
KTEYITFMKGVVSPPLNFPGTAYRKALKSRSTILKFIERRMEDRISEIGR
ENLEEDDLLNWVLKNSNLSKEQILDLVLSLLFAGHETSSVSIALAIYFLQ
ACPLAVQQLREEHSEIARVKRELGERELKWEDYKKMEFTQCVISETLRLG
NVVRFLHRKALKDVRYNGYDIPKGWKVLPVIAAVHLDPSLFDHPQHFNPW
RWYHQGNRLSSSTATNNTNFMPFGGGPRLCAGSELAKMEMAVFIHHLILN
FDWELADSDQAFAFPFVDFPKGLPITVRQHTNNYVVI
>NjCYP90B2
MADLDFLIFIFSSIFTLLLIFNHITKHRKISLNLPPGNSGWPFIGETIAY
LKPYSAISIGDFMEQHISRYGKIYKSNLFGEPTIVSADAGLNRFILQNEG
RLFECSYPTSIGGILGKWSMLVLVGDMHRDMRIISLNFLSNARLRTHLLP
EVEKHTLLVLNSWKHNSTFCAQDEAKKFTFNLMAKHIMSLDPGKPETEQL
KTEYITFMKGVVSPPLNFPGTAYRKALKSRSTILKFIERRMEERIREIGR
ENLEEDDLLNWVLKNSNLSKEQILDLVLSLLFAGHETSSVSIALAIYFLQ
ACPLAVQQLREEHSEIARVKRELGERELKWEDYKKMEFTQCVISETLRLG
NVVRFLHRKALKDVRYNGYDIPKGWKVLPVIAAVHLDPSLFDHPQHFNPW
RWYHQGNRLSSSTVTNNTNFMPFGGGPRLCAGSELAKMEMAIFIHHLILN
FDWELADSDQAFAFPFVDFPKGLPITVRQHTNNYVII
>NjCYP90C1
MEGVLVGMISLIIILLASLYANKELRIRLRRKIKKEEVEDKDGDSKIKIK
GNKGWPFIGETLDFIASGYSSNPLSFMDKRKSLYGKVFRTHILGMPIVVS
TDPDVNKVVLQNNGSVFIPCYPKSITELLGKSSILQMNGNLQKRVHALIG
GFFRSPELKARITRDIEKSVKQSLLNWNHHKHVFLQEETKKITFEVLVKV
LMGLGPGEEMEFIKKEFEEFIKGLICLPIKLPGTRLYKSLKAKERLLKIV
KKIVEERKLGMEKNNKIGLSNDAIDLLLRETREWGEKQERIPLDFISGNI
IEMIIPGEDSVPMVMTLAVKYLTHSPAALARIVEENMELKKQKAHSSSEY
AWTDYMSLPFTQNVISETLRMANIINAVWRKAVKDVEIKGYLIPKGWCVL
ASFSAIHMDEKNYDNPYHFDPSRWERREASANNSTFTPFGGGQRLCPGLE
LSRLEISIFLHHFVTTYRWVAEKDEIVTFPTVKMKRKLPITVTPI
>NjCYP90C2
MEGVLVGMISLIIILLVSLYANKELRIRLRRKIKKEEVEDKDGDSKIKIK
GNKGWPFIGETLDFIASGYSSNPLSFMDKRKSLYGKVFRTHILGMPIVVS
TDPDVNKVVLQNNGSVFIPCYPKSITELLGKSSILQMNGNLQKRVHALIG
GFFRSPELKARITRDIEKSVKQSLLNWNHHKHVYLQEETKKITFEVLVKV
LMGLGPGEEMEFIKKEFEEFIKGLICLPIKLPGTRLYKSLKAKERLLKIV
KKIVEERKLGMEKNSKIGLPNDAIDLLLRETREWGEKQERIPLDFISGNI
IEMIIPGEDSVPMVMTLAVKYLTHSPAALARIGEENMELKKQKAHSSSEY
AWTDYMSLPFTQNVISETLRMANIINAVWRKAVKDVEIKGYLIPKGWCVL
ASFSAIHMDEKNYDNPYHFDPSRWERREASANNSTFTPFGGGQRLCPGLE
LSRLEISIFLHHFVTTYRWVAEKDEIVTFPTVKMKRKLPITVTPI
>NjCYP90D1
MEINNIWILFAASIIFFFFIFNRIFFKKVKTNINVTNISPLKFKSSLPLG
TLGWPFIGETIDFISSAYTDNPESFMNKRRLMYGNVFKSHIFGSATIVST

DAELSRCVLQSDSKTFVPSYPKSLTELMGKSSILLINGSLQKRIHGLIGS
FFKSPLLKAQITTDMQKYVQQSMESWKEDQPIYIQDETKKIAFQVLVKAL
ISLDPGEEMEFLKKQFQEFIAGLMSLPINIPGSRLYRSLQAKKKMVKLVQ
EIIQEKRKSCGVNSTVPKDIADVLLNDTSEQLTDDLISDNMIDLMIPGED
SVPVLMSLAIKYLSDCPSALQQLTEENMMLKRQKDELLCWSDYLSLTFTQ
NVITETLRMGNIISGVMRKAMKDVKIKGYLIPKGWCVFMYFRSVHLDHNQ
YDSPYKFNPWRWQGKDMSSCSCSFTPFGGGQRLCPGLDLARLEVSIFLHH
FVTQFRSGGRQKRIQLSTSLQ
>NjCYP93A1
MADIQSYFILFPIAAISIILILFKSSSTKSKFRLPPTPFSLPIIGHLHLL
GPSPHQAFHKLSFKYGPVFRLFLGSKPCVVFSSSETGKELFKTFDNVFLN
RPYNSSMDYISYGGRGFIFAPYGPYWKFLKKIVVSELLNSKTLDSLLPVR
HDEINRFLRYLSQKAKVGESVELEGDLMKMANNIISRMLMGKRCSDENDD
AGDIKSIVTDIGELMFTFNLSDHIWFLKNIDVQGIGNRSKEIRGRFDSLI
EKIIDKHEEARKEKKGKGEVKDLLDLLLDISEDESMEIKMTRDNIKAFIL
DIFGAGTDTSAITTEWALAELVNHPKIMKKALEEIDQVVGKDRLLQESDI
PNLPYLQAIVKESLRLHPTAPIVQRISTEDSTIGGYDIPADTVIFYNIWS
VGRDPAHWENPLEFWPERFIEKQMDVRGQSFELLPFGSGRRMCPGTSLGL
MVVYVTLGCMIQCFDWKAGKNGDLSSVDMEEGIGITLPRANPLVCVPVAR
LDPIPFSG
>NjCYP93B1
MLTMHQVISLGIGLVFILFVVFLKRRHIGRRMPGPLALPVIGHLHLLEPL
LHHCFHRLSTRYGPLFHLKIGSQPCVVASTPELAKVFLKTHELKFSSRKT
STAIKLLTYNASFAFAPYAPYWKYIKRVCTYELLGTRNLNHFSPIRTSEI
RSLLKLLMDRAQASEAVNLTEELMKLTNNIISQMMLSSKSSGTEEDAEEV
RRLVREVTMLFGEFNVSDFISLFKNIDVGGFKKRSRATKKRYDALLEKII
TERERERIRAKNGELLIRDEEKGKDFLDMMLDAIEDGNCEVHITRDHIKA
LVLDFLTAATDTTAISIEWTLSELINSPKVLKKAQDEIDKVVGNERLVKE
SDAPNLPYIQAIIKENFRLHPPIPLLIRKSVEDCTVEDFYISANTVLFIN
IWSIGRNPKYWENPMEFLPERFLEDDMSSMDVKGHNYELLPFGSGRRGCP
GISLAMQELPFVLAAMIQNFNWKPTTLIGDKLDMSERSGLTAPRANDLVC
IPVVRNELL
>NjCYP94A1
MTELELSTSLIFCIIPLVSLFLFKFLQTKSKNGNPASYPLIGSFIEILAN
KERVPQWSSDLIKKSPSSTIIVNRPLGAVNIMTANPSIVQHILKTRFDIY
QKGDVFRSTLSDLLGNGIFNVDGQTWKFQRQVSSHEFNTKSLRKFVENVV
DIELNERLVPILAAAAANETVLDLQDILQRFAFDNICKIAFGYDPAYLLP
SLPQEKFAVAFEEAVRISSSRFSSIIPLYWKAKKFFNIGSERQLKIAVAQ
VREFAGKLLREKKQELKENSSLESVDLLSRFLNNGHADEDFVIDIVISFI
LAGRDTTSAALTWFFWLLSKNSEIENEILKEIKEKSESPIYDEVKDMIYM
HASLSESMRLYPPVPTDSKEASKDDVLPDGTIVRKGTRVTYHPYAMGRSE
ELWGPDWPEFRPERWLEIDEVTGKRSFKARDTYIYPVFQAGPRICLGKEM
AFLQMKRVVAGVLRRFRVVPALEEGVEPVLVNYLTAKMKGGFPVRILDRV
DA

>NjCYP94A2
MIELELSTSLIFCIIPLVSLFLLKFLQTKSTNGNPASYPLIGSFIELLAN
KERLPQWSTDLIKKSPSSTIIVNRPLGAVNIITANPSIVQHILKTRFDIY
HKGDVTRSTLSDLLGDGIFNADGQTWKFQRQVSSHEFNTKSLRKFVENVV
DIELNERLVPILAAAAANETVLDLQDILQRFAFDNICKIAFGYDPAYLLP
SLPQEKFAIAFEEAVRITSSRFSSIIPLYWKAKKFFNIGSEKQLKIAVAQ
VREFAGKILREKKQELKEKSSLESVDLLSRFLSSGHADEDFVIDIVISFI
LAGRDTTSAALTWFFWLLSKNSQIENEIIKEINQKSESPIYDEVKDMIYM
QASLCESMRLYPPVPTDSKEASRDDVLPDGTIVRKGTRVTYHPYAMGRSE
ELWGSDWPEFRPERWLEIDALTGKRNFKARDSYIYPVFQAGPRICLGKEM
AFLQMKRVVAGVLRRFRVVPAMEEGVEPVLVNYLTAKMKGGFPVRILERV
DA
>NjCYP94A3
MLANKHRRIQWTADLIKSSPSYTVVLHRPLGAVSILTGNPSNVQHILKTR
FDIYQKGDGIRRILSDLLGDGIFNADGEIWKFQRQLSSHEFNTKSLRNFV
ENVVDTELNDRLIPILAGAVENQTVLDFQDILQRFAFDNICKIAFGYDPA
YLLPSLPQAEFAVAFEEAVRISSERFVSITPLFWKIKRLLNIGSEKKLKL
AIADVREFARKILREKKKELMEKSSLDSVDLLSRFLSSGHSEEDFVTDIV
ISFILAGRDTTSAALTWFFWLVSKNTAVENEILREIKQKSESPIYDEVKD
MIYTHASLCESMRLFPPVPTDSKEANNDDVLPDGTLVKKGNRVMYHPYAM
GRMECLWGSDWSEFRPERWLETDPVTGKRSFAGKDSYTYPVFQAGPRICL
GKEMAFLQMKRVVAGVLRRFRVVPVVDENGVEPVFMAYLTSKMKGGFPVR
IVERKRNGV
>NjCYP94B4
MFISLLIIISLAIAILLSFYQSATNTVYGPTSYPFIGCLISFYKNRRRLV
DWYTDLLSDSPNQTILVHRLGAHRTIVTANPANVEYILKTNFNNFPKGKP
FTEILGDLLGSGIFNVDGEMWSTQRKLASHEFSAKSLREFVVKTLEDKVH
NRLVPLLEAAARDNTVLDLQEILRRFAFDTICHVSLGYDPCCLDITRPVP
PLVTAFDTASMICAMRGAAPVCAVWKAKRALTAGNERTLKESVQLVHNSV
DDIIRSKRQSLQANDGAGGDLLSKLLVAGHGDEVVRDMVISFLLAGRDTT
SAAMTWLFWLLTKHDKVKNELLKEIMESKADGVEYDDLKDMNYIKACLCE
SMRLYPPVVWDSKHAAGDDVLPDGTPVFRGDRVTYFPYGMGRMEELWGKD
RFEFKPERWFNGPGRVMKMVSAYKFPVFQAGPRVCLGKEMAFIQMKYVVS
CLMRRFDFRPISSKQPVFVPLLTAHMAGGLKVRVSKREYYK
>NjCYP94C1
MDNYFTFSLLFLIFSLLFFIFLCTKQCCNCDICHAYVTASWKSEYNNLCD
WYTHLMQKSPTGTIHIHVLGNIITSNPTNVEHMLKTKFDNYPKGKKFSAI
LGDLLGKGIFNVDGDSWKFQRKMASLELGSVSIRSYAFEIVKSEISSRLI
PLLCSVKSDSDSTMKSTNSTSLDLQDAFRRFSFDSICKFSFGVDPGCLNL
SLPVSNLAASFDLASKLSAERAMAVSPIVWKIKRLLNVGSEKKLAESIKS
VNHIADKVIEHKRDRDFSSQQDLLGRFMNSIDDDSYLRDIIKSFLLAGRD
TVASALTSFFWLLANHPEVESAIRAESDRIMGENEEIASFEQMRGLHYLQ
AALYESMRLYPPVQFDSKFCQKDDVLPDGTFVQKGTRVTYHPYAMGRMEK
IWGLDCLEFKPQRWLQNGVFKSESLFKYPVFQGGLRVCLGKELALVEMKS

VALALIRRFDIRLATSNKSLQFAPGLSATLNGGLPVVVRERMAS
>NjCYP94C2
MDFQSLFSFVFFTFTAVFFLFSLCLYILRLKPCCDCDVCTAFITRSWAEE
FVNISDWYTHLLRKSATGTIHLHVLNNVITANPDNVEYMLKTNFDNYPKG
KAFSAILGDLLGRGIFNVDGKCWRFQRKMASLELGSVSIRSHALNVVVDE
IESRLIPFFSSVADNNDGSVLDLQDVFRRFSFDSICKFSFGLDPGSLKPS
LPVSELESAFDLSSKLCAERGMAPSPLIWKIKRLFNMGSEKKLKASIKTV
KKLADQVIKKRRETGSSSTNDLLSRFMGSISDDDYLRDIVISFLLAGRDT
VASALTSFFLLLSQNPKVVKKIREESDRVMGTTRETLASFNDLQNLHYLQ
AALHESMRLYPPVQFDSKFATQDDVFPDGTFVSRGSRVTYHPYAMGRMER
IWGPDSMEFKPERWICDGEFKQETAYKYPVYQGGVRVCLGKEMSLVEMKV
VALCLIRRFHVRVVNPSQVLRFAPGLTATVSGGLPAFVQPIDLPR
>NjCYP94E1
MLELKVDSLSILILSLSTVSLIYYFFFRSATVTPTTTPCPHSYPIIGNII
ALLRNRHRFHDWITDMLSTTTTIKADGFLHLSQGICTVHPPNLHHLLRSN
FSNYIKGSRFHSVLSELLGDGIFNADGDLWSVQRKIASHEFNTKSLKNFI
SNTVTSQISNRLIPCLSTASDNHQTLDLQDVLNRFTFDNICNVAFGVDPA
TNPASSLFVRAFDFAVEGSLNRFISPLHVIWKLQRFLNIGSERKYRDAIE
LIDEFTMNIIRTKERELEMKKNDEEEEKLDLLSRFMDSSTNLGLASEDEK
RKFLRDIVISFILAGKDTTSTALTWFFWLIAGHPRCERLIYNEIVTAAES
PENSPAGSFNYDDLKKLHYLHAALSESMRLFPPVPMNSKLTVDDDILPDG
THVAKGWFADYSAYAMGRMESLWGPDCREYKPERWLDDDGVYQPSDQFKY
PVFHCGPRICLGKDMAYVQMKCVVAAVLYEFEIEAVDGGGSAERMVDPPY
MLSLLLKKRDGLRVRLRRRQH
>NjCYP94E3
MVADLVYDFFPHVIISLLVILFVHLSRRSRRSGVKTPCPQSYPIVGNLIG
FLRNRHRFHDWVCDMLSTTPSLTLQVNGFLGLSHGICTADPTNLHHLLRS
NFPNYIKGQRYTSVLHELLGRGIFNSDGHIWSSQRKIASYEFNTKSLRTF
IADTVKSQLSRSLIPHLLSASDAGETIDLQQVLRKFGFDNICNVAFGVDP
GLLRSNSKDSNNLLFVHAFDLAVEHVSHRFMSPLPAVWKIQRFFNIGNER
KYKQAIEIVNQFAMDIIISKETQIDHAQSNEDLLSRFMVSSWDMGFNDEE
RRKFVRDIIISFVLAGKDSTATALTWFFWLLAGHPRCRDLVHKEFSTVMA
SSHYIHPTNLTFDDLKNLHYLHAALSESMRLFPPVPINSRLTVDHDKLPD
GTYVGKGWFADYSAYAMGRMERLWGSDCREFKPERWLDNDGVFQPVDQFK
YPVFHGGFRMCLGKEMAYLQMKSVVVGIMYEFEVEVIGGGGTPERMVAPP
YTLSLLLGMKNGLRVRLKKVQQH
>NjCYP96B1
MAITIYLEILLVLFLFKFLLYYLRTTITTTHRRSTLPTNWPSLGMLPAIL
RNAHRLHDFATDILKESGGTFVVKGPWFTKMDIMATADPANIHHILSRNF
SNYPKGPEFRKIFDILGDGIFNADSELWEIHRKTTMSLMNLAEFKDLSAR
VIWRKVEKGLVPVLESICQNAKETDLQDIFQRFTFDSICTLVLDYDPQSL
SVKLPFIECDKAFIDTEEALLYRHILPEGFWKLQKRLQIGKEKKLSKAWT
SFDRFIYPCIGKNQNEMGFDSFNLLKSFTKAYKEESGSSGDKNKFLRDTF
LNLMIAGRDTTSTALTWFFWLLSKNPIAEIKIREEIKSKLNKKNDEKQSF

FLKTEDLGKLVYLHGALCESLRLYPPVALEHKAPVQPDTLPSGHWIDKNT
KTVLCFYSMGRMETIWGKDCLDFKPERWITEKGGIKHEPSYKFPAFNAGP
RTCIGKEMAFTQMKIIAASIIYDYSVEVVQDHPITPSDSIILQMKYGLKV
RITKRSV
>NjCYP96B2
MTPDLILNAYRVHDFATDLLKQSHGTFMLKGPWFANMDMLITSDPANIHY
MLSKNFPNYPKGPEFRKIFDILGDGIFNSDNHVWEIQRKTTMSLLKHPDF
NSHLEINIRNKIEKGLLPLLDMVSKDEQESDLQEIFQRFTFDAICVLLLD
YDPETLSVDLPYNACEKAFTDAEEALLWRHVLPERIWKLQQRFGIGKEKK
LMEASKVFDEFIYKCLSRKEDAMDDDRRVEKEETGLLKSLMTSFQGQTGT
SGDSRTFLKDTILNLMIAGRDTTSTGLSWFFYLLAQNPRIERKIREEIKK
EIGGANWKCLGVEQLKGLVYLTGGLCEALRLYPPVALEHKAPSMADVLPS
GHAVNQHSKIILSFYSMGRMDWIWGEDCLEFKPERWFSEGGKIKHEPSYK
FTAFHAGPRTCLGKEMGFIQMKMVASAIIYHYNVEVAEGHEVCPGDSIIL
QMKYGLKVRLNPISQIKN
>NjCYP97A1
MAANCPLLQFSSHSLEAKFHSNRLRVSSPSASIAKLNRTCRWSVIRCSSS
NGREPYSLDGGEKNAEKALEEKRRAELSARIASGEFTVQKTGTSFQSVLI
NGLSKLGVPSEIIEPLSKLINGGEDYPKIPEAKGAISAIRSEAFFIPLYE
LYLTYGGIFRLTFGPKSFLIVSDPSIAKHILRDNSKAYSKGILAEILEFV
MGTGLIPADGDVWRVRRRAIVPSLHQKYVAAMISLFGQATDRLCKKLDAA
AYDGEDVEMESLFSRLTLDIIGKAVFNYDFDSLTTDTGIVEAVYTVLREA
EDRSVSPIPFWEIPIWKDISPKQKKVNEALKLINGTLDNLIAICKRMVDE
EELQFHEEYMNETDPSILHFLLASGDDVSSKQLRDDLMTMLIAGHETTAA
VLTWTFYLLTKEPSVMAKLQNESLRLYPQPPVLIRRSIIDDTLGDYPIKR
GEDIFISVWNLHRCPKHWEDAEKFNPERWPLDGPNPNETNQNFSYLPFGG
GPRKCVGDMFASFEAVVAVAMLVRRFNFQMALSAPPVEMTTGATIHTTQG
LNMSVTRRINPPLVSTFPVLQVNSPADADPLPKGEVSSAARS
>NjCYP97A2
MAANCPLLQFSSHSLEAKFHSNRLRVSTPSASIAKLNRTCRWSVIRCSSS
NGREPYSLDGGEKNAEKALEEKRRAELSARIASGEFTVQKTGTSFQSVLI
NGLSKLGVPSEIIEPLSKLINGGEDYPKIPEAKGAFSAIRSEAFFIPLYE
LYLTYGGIFRLTFGPKSFLIVSDPSIAKHILRDNSKAYSKGILAEILEFV
MGTGLIPADGDVWRVRRRAIVPSLHQKYVAAMISLFGQATDRLCKKLDAA
AYDGEDVEMESLFSRLTLDIIGKAVFNYDFDSLTTDTGIVEAVYTVLREA
EDRSVSPIPFWEIPIWKDISPKQKKVNEALKLINGTLDNLIAICKRMVDE
EELQFHEEYMNETDPSILHFLLASGDDVSSKQLRDDLMTMLIAGHETTAA
VLTWTFYLLTKEPSVMAKLQNEVDSVLGDRIPTIEDMKKLRYTTRVINES
LRLYPQPPVLIRRSIIDDTLGDYPIKRGEDIFISVWNLHRCPKHWEDAEK
FNPERWPLDGPNPNETNQNFSYLPFGGGPRKCVGDMFASFEAVVAVAMLV
RRFNFQMALSAPPVEMTTGATIHTTQGLNMSVTRRINPPLVSTFPVLQVN
SPADADPLPKAEVSSAARS
>NjCYP97A3
MAANCPLLQFSSHSLEAKFHSNRLRVSSPSASIAKLNRTCRWSVIRCSSS

NGREPYSLDGGEKNAEKALEEKRRAELSARIASGEFTVQKTGTGFQSVLI
NGLSKLGVPSEIIEPLSKLINGGEDYPKIPEAKGAFSAIRSEAFFIPLYE
LYLTYGGIFRLTFGPKSFLIVSDPSIAKHILRDNSKAYSKGILAEILEFV
MGTGLIPADGDVWRVRRRAIVPSLHQKYVAAMISLFGQATDRLCKKLDAA
AYDGEDVEMESLFSRLTLDIIGKAVFNYDFDSLTNDTGIVEAVYTVLREA
EDRSVSPIPFWEIPIWKDISPKQKKVNEALKLINGTLDNLIAICKRMVDE
EELQFHEEYMNETDPSILHFLLASGDDVSSKQLRDDLMTMLIAGHETTAA
VLTWTFHLLTKEPSVMAKLQNEVDSVLGDRIPTIEDMKKLRYTTRVINES
LRLYPQPPVLIRRSIIDDTLGDYPIKRGEDIFISVWNLHRCPKHWEDAEK
FNPERWPLDGPNPNETNQNFSYLPFGGGPRKCVGDMFASFEAVVAVAMLV
RRFNFQMALSAPPVEMTTGATIHTTRGLNMSVTRRINPPLVSTFPVLQVN
SPANADPLPKGEVSSAARS

>NjCYP97A4

MAANCPLLQFSSHSLEAKFHSNRLRVSTPSASIAKLNRTCRWSVIRCSSS
NGREPYSLDGGEKNAEKALEEKRRAELSARIASGEFTVQKTGTSFQSVLI
NGLSKLGVPSEIIEPLSKLINGGEDYPKIPEAKGAISAIRSEAFFIPLYE
LYLTYGGIFRLTFGPKSFLIVSDPSIAKHILRDNSKAYSKGILAEILEFV
MGTGLIPADGDVWRVRRRAIVPSLHQKYVAAMISLFGQATDRLCKKLDAA
AYDGEDVEMESLFSRLTLDIIGKAVFNYDFDSLTNDTGIVEAVYTVLREA
EDRSVSPIPFWEIPIWKDISPKQKKVNEALKLINGTLDNLIAICKRMVDE
EELQFHEEYMNETDPSILHFLLASGDDVSSKQLRDDLMTMLIAGHETTAA
VLTWTFHLLTKEPSVMAKLQNEVDSVLGDRIPTIEDMKKLRYTTRVINES
LRLYPQPPVLIRRSIIDDTLGDYPIKRGEDIFISVWNLHRCPKHWEDAEK
FNPERWPLDGPNPNETNQNFSYLPFGGGPRKCVGDMFASFEAVVAVAMLV
RRFNFQMALSAPPVEMTTGATIHTTQGLNMSVTRRINPPLVSTFPVLQVN
SPANADPLPKGEVSSAARS

>NjCYP97A9

MAASLTTLQFPSPYLKSHHSHTVKFKLKSAPIANPNGSSRSCGIKCSYSN
GRKPDSSSDEKSGKSLEALREEKRRAELSARIASGEFTVEKPSFGSLLVN
SLTKLGVPSEFLESLSQLINLDDKYPKIPEAKGAISAIRSEAFFIPLYEL
FLTYGGIFRLTFGPKSFLIVSDPIIAKRILKDNAKAYSKGILAEILEFVM
GTGLIPADGEVWRVRRRVIVPALHLKYVAAMIGLFGEATDRLCKKLDAAA
YNGEDVEMESLFSRLTLDIIGKAVFNYDFDSLTTENGIVEAVYTVLREAE
DRSVSPIPTWEIPIWKDISPRQKKVNESLKLINTTLDDLIAICKRMVDEE
DVQFNEEYMNESDPSILHFLLASGDNVSSKQLRDDLMTMLIAGHETTAAV
LTWTFYLLSKDPSVVSKLQNEVDAVLGDRFPTIEDMKKLKYTTRVINESL
RLYPQPPVLIRRSLEDDVLGKYPIKRGEDFFISVWNLHRSPTHWEDADKF
NPERWPLDGPNPNETNQNFSYLPFGGGPRKCVGDMFASFEAIVAVAMLIR
RFNFQMALGAPPVKMTTGATIHTTEGLNMTVTRRIHPPIVPTLQNMEPSA
NVPEADPVVS

>NjCYP97B2

MASLTSTIFHHQTFHDFAPNSHVNYFPFLSRHGRTLSHSPSSSTTHLYNS
KLRRSQIRCKSTEEEVKKKKRNIMDNASNLLTNLLNGGSLECMPTAEGAV
SDLFGKPLFFSLYDWFLEHGSVYKLAFGPKAFVVVSDPIVARYILRENAF

SYDKGVLADILEPIMGKGLIPADLDTWKLRRRVIAPGFHGLYLEAMVKLF
TECSERTILKFEKLLEGGKEIELDLEAEFSSLALDIIGLGVFNYDFGSVT
KESPVIKAVYGTLFEAEHRSTFYIPYWKVPLARWVVPRQRKFQKDLKVIN
DCLDGLIKNAKDTRQETDVEKLQQRDYLNLKDASLLRFLVDMRGVDVDDR
QLRDDLMTMLIAGHETTAAVLTWATFLLAQHPDKMKKAQAEIDSVLGQER
TTFESLKKLQYLRLIVVETLRLYPQPPLLIRRSLAPDQLPGGYMGDKDGY
AIPEGTDIFISVYNLHRSPYYWDKPDEFEPERFLVQKKSQGIEGWAGFDP
SRSPGALYPNEIISDFAFLPFGGGPRKCVGDQFALMESTVALCMLLQKFD
VELKGSPESVQLVTGATIHTKNGLWCNLKKRSTTTTLH
>NjCYP97C1
MLSSQSFSLLTTPHSFNRTSLHPKIFTIKSSTQKPSIKPPQNKTKSWVSP
DWLTSITRSLSSGQDESNIPIASAKLEDVSDLLGGALFLPLFKWMNQYGP
IYRLAAGPRNFVIVSDPAIAKHVLRNYGKYSKGLVAEVSEFLFGSGFAIA
EGPLWTARRRAVVPSLHKKYLSVIVDRVFCKCAVRLVEKLKTSAISGSAV
NMEEQFSQLTLDVIGLAVFNYNFDSLNADSPVIEAVYTALKEAEARSTDL
LPYWKIKALCKIIPRQIKAEQSVTVIRRTVEELIQKCKDMVESEGERINE
EDYVNDADPSILRFLLASREEVSSLQLRDDLLSMLVAGHETTGSVLTWTS
YLLSKNPSSLKKAQEEVDRVLQGRLPNYDDIKDLKFVTRCINESMRLYPH
PPVLIRRAQGTDILPGDYKVNAGQDIMISVYNIHHSSQVWDRADEFVPDR
FDLEGPVPNETNTDFRFIPFSGGPRKCVGDQFAMLEAIVSLAIFVQNIEF
ELIPNQIINMTTGATIHTTNGLYMKVSQRETKPEFAPSGSSR
>NjCYP98A1
MPSLIILLLTLLPILLYFYKSLKFKLPPGPHPWPIIGNLYQIKPVRFRCF
YEWAQSYGPIISVWFGSTLNVVVSNTELAKEVLKEKDQQLADRHRSRSAA
KFSRDGKDLIWADYGPHYVKVRKVCMLELFTPKRLEALRPIREDEVTAMV
ESIFNDCTSSDLKGKSLLVKKYLSAVAFNNITRLAFGKRFVNSEGVIDEQ
GKEFKAIVANGLKLGASLAMAEHIPWLRWFFPLEEEAFAKHGARRDRLTR
AIMEEHTLARQKSGGAKQHFVDALLTLQDQYDLSEDTIIGLLWDMITAGM
DTTAISVEWAMAELIKNPRVQQKAQEELDRVIGLKSVLTESDFSNLPYLQ
SVAKEALRLHPPTPLMLPHRANTTVKIGGYDIPKGSNVHVNVWAVGRDPA
VWKNGSEFRPERFFEEDVDMKGHDFRLLPFGAGRRVCPGAQLGINLVTSM
LGHLLHHFSWAPPVGIKAEDIDFSENPGLVTYMRTPLEAVPNPRLPRHLY
KRVATDM
>NjCYP98A2
MALPLLPILAVLFPIYLLYTLIQRLRFNLPPGPRPLPIVGNVYDIKPIKF
RCYAEWAQTYGPIFSLYLDSRLNVVVNNTELAKEVLKENDQLLADRHRNR
ATMTFSRGGKDLIWADYGPHYVKVRKVCNLELFSPKRLEALRPIREDEVT
AMVESIFNHCTNPDNIGKSLSMRSYFGSVAFNNITRLAFGKRFMNSDGSI
DDQGKEFKGIVSNGIKIGGKVFMGETVPWLRWMFAGENEILEKHESRRAK
LTREIMEEHTLARKKTGGAKEHFVDALLTLQKQYDLSDDTVIALLWDMIT
AGMDTTSISVEWAMAELVRNPRVQQKAQEELDRVIGSDRVMTESDFSSLP
YLQSIAKEALRLHPPTPLMLPHKANSKVKIGGYDIPKGSIVHVNVWAIAR
DPATWRDPLEFRPERFLEEDVDMKGHDFRLLPFGAGRRICPGAQLAINLV
TSMLGHLLHHFTWAPPAGVKPEELDMDENPGMVTYMKTPLQAIPTPRLPS

KLYARVLVEC
>NjCYP98A3
MALPLLPILAVLFPIYLLYTLIQRLRFNLPPGPRPLPIVGNVYDIKPIKF
RCYAEWAQTYGPIFSLYLDSRLNVVVNNTELAKEVLKENDQLLADRHRNR
ATMTFSRGGKDLIWADYGPHYVKVRKVCNLELFSPKRLEALRPIREDEVT
AMVESIFNHCTNPDNIGKSLSMRSYFGSVAFNNITRLAFGKRFMNSDGSI
DDQGKEFKGIVSNGIKIGGKVFMGETVPWLRWMFAGENEILEKHESRRAK
LTREIMEEHTLARKKTGGAKEHFVDALLTLQKQYDLSDDTVIALLWDMIT
AGMDTTSISVEWAMAELVRNPRVQQKAQEELDRVIGSDRVMTESDFSRLP
YLQSIAKEALRLHPPTPLMLPHKANSKVKIGGYDIPKGSIVHVNVWAIAR
DPATWRDPLEFRPERFLEEDVDMKGHDFRLLPFGAGRRICPGAQLAINLV
TSMLGHLLHHFTWAPPAGVKPEELDMDENPGMVTYMKTPLQAIPTPRLPR
NCTHVCRWNVKEKITFFFVI
>NjCYP98A4
MALPLLPILAVLFPIYLLYTLIQRLRFNLPPGPRPLPIVGNVYDIKPIKF
RCYAEWAETYGPIFSLYLDSRLNVVVNNTELAKEVLKENDQLLADRHRNR
ATMTFSRGGKDLIWADYGPHYVKVRKVCNLELFSPKRLEALRPIREDEVT
AMVESIFHDCTNPDNIGKSLSMRSYFGSVAFNNITRLAFGKRFMNSDGSI
DDQGKEFKGIVSNGIKIGGKVFMGETVPWLRWMFAGENEILEKHESRRAK
LTREIMEEHTLARKKTGGAKEHFVDALLTLQKQYDLSDDTVIALLWDMIT
AGMDTTSISVEWAMAELVRNPRVQQKAQEELDRVIGSDRVMTESDFSRLP
YLQSIAKEALRLHPPTPLMLPHKANSKVKIGGYDIPKGSIVHVNVWAIAR
DPATWRDPLEFRPERFLEEDVDMKGHDFRLLPFGAGRRICPGAQLAINLV
TSMLGHLLHHFTWAPPAGVKPEELDMDENPGMVTYMKTPLQAIPTPRLPS
KLYARVPVEC
>NjCYP701A1
MALHEIIQATLGTAVALGGLSLLFNGENERRSFNPPPLPEIPGLPVVGNL
LQLKEKKPHKTFTKWAETYGPIYSIKAGSTSMIVLNSNLVAKEAMVTRFP
SISTRKLSNALKILTLDKTMVAMSDYDEFHKTVKRHLLTSVLGPNAQKKH
RHHRDALVENTSSNLHSHLKNYPLQAVNLREIFESELFGLSVKQALGKDV
ESIYVGDLGTTLTRKEIFDVLVTDPMEGSIEVDWRDFFPNLKWVPNTSFE
TKLERMYMRREAVMKALFQQHRKRIESGEEMNSYIDYLLSEAKTLTEKQM
SMLLWETIIETADTTLVSTEWAMYELAKNPKQQERLFHEIQSVFGSDKIS
EDKICKLPYLSAVFHETLRKYSPAPIIPLRYVHEDTELGGFHIPAGSEIA
INIYGCNMDPTVWENPEEWRPERFLDEGNDPVELHKTMAFGGGKRVCAGA
LQAMLIACTSIGRLVQEFEWKLEDGEEDKIDTLGLTTRKLHPMKAFITPR
N
>NjCYP701A2
MEFLEAISIIFACICIWFFKRFFFNRTQRSSNLPPLPEVPGIPILGNLLQ
LKEKKPHKTFTKWAHKYGPIYSIRTGATTLVVLNNNHVAKEAMVTRFSSI
STRKLSTALTILTSDKSMVAMSDYNDFHKAAKRCILTHILGPAAQKRLRS
HRDTLIENMSKHLHDFSKNNPLEPVNFRHYFESELFGLALKQSLGKDVES
IYVEKLGTTFSREELFDIFVLEPLMGALDVDWRDFFPYLKWVPNKAIEER
VQKMHIRREAAMKTLIQQSKKRIDSGEKTDCFHEFLLSLEKPLTENQVLM

MLWEIIIETSDTMMVTTEWAMYELAKNPKQQEKLYQELQTVCVSDKITEE
KLSQLPYLCAVFHETLRKYSPVPVIPLRYVHEDTEIGGYHVPAGSEIAIN
IYGCNMDNKVWENPEDWNPGRFLENKNNTMDLHVTMAFGGGRRVCAGALQ
AFHISCVTIGRLIQEFKWKLADGEEENVDMLGLTTHKLHPMKAIIEPRA
>NjCYP701A3
MDAVSDMQTIPLSTTIAIGGTAVAIVGAIYFWFLRSFASRHSDQRNHPPP
VPEVPGIPVLGNLLQLKEKKPYMTFTKWAEMYGPIYSIRTGATSMVVVSS
NEIAKEVLVTRFPSISTRKLSYALKVLTEDKTMVAMSDYNDYHKTVKRNI
LTAVLGPNAQKKHRVHRDTMMDNVSNELHAFSKNSPNQEVNLRKIFQSEL
FGLAMKQAMGKDVESIYVKDLKTTMKRDEIFQVLVVDPMMGAIEVDWRDF
FPYLKWVPNKTFENIIHRMYTRREAVMKALIHEHKNRIASGQNLNSYIDY
LLSEAQTLTDKQLLMSLWEPIIESSDTTMVSTEWAMYELAKNPNIQDRLY
DEIKSVCGSDKITEEKLPQLPYLYAVFQETLRKHCPVPIIPLRYVHEDTV
LGGYHVAAGTEVAINIYGCNMEKKVWENPEEWNPGRFLGENEGMDLYKTM
TFGGGKRMCAGSLQAMVISCMGIGRLVQEFEWRLKDEDAEEDVSTLGLTT
QKLHPLLAVIKPRK
>NjCYP704B1
MLSQLIHFNRLFDYQTSLAQKQTTYRLITPSHSEIYTADPVNIEYFLKTN
FSNYSKGEYNRGIMSDLFGDGIFAVDGEKWRHQRKLASYEFSTKILRDFS
TSVFISNAVKLASKVSSAAISPHIIDLQDLLMKSTLDSMFKVGFGVELDT
LSGLDEKSNEFMKAFDESNVIVYRRYVDVLWRIKRFFNIGLEASLKQNIQ
IIDNFVYDLIQRKREQMQNAKFDGEKEDILSRFIMEGDKEDKSKKMSDKY
LRDISLSFIIAGKDTSANTLTWFFYMMCKYPLIQEKIAQEVREATHYNKN
NNNDDDDDDDDDDDDDDDDESLRILDENLLNIEGFGQKLMSEGALDKMQY
LHAALTETLRLYPAVPVDGKRAEEDDVLPDGLKIKKGDGINYMAYAMGRM
TYIWGNDALQFLPQRWLDHNNLFQPQSPFKFTAFQGGPRICLGKEFAYRQ
MKILAAMLLYFFKFKLVDETKDASYRTMFTLHMDKGLNLYASFRHK
>NjCYP704B2
MNTLFMVSTSTIFFLLTGLYFSPLFILTIIGFLLLFLIALSAVLLVPYFS
ELTSNDHRPPVVGTTFNMLIHFNHLLDYITVIARNHRTFRFVKPTHSEVY
VADPINVEHILKTNFPNYTKGDYHKGIMGDLFGKGIFAADGDIWRHQRKL
ASHEFSTKVLRDFSTVVFRSNTAKLVRKVSKSAADNQIINLQDLLMKSTL
DSIFKVGFGFDLDTLSGLSDVSNQFMTAFDDSNSIIFWRYVDVFWKVKRY
FSIGSEAALKKNIKVIDNFVYDLIEHKREQMKSGKLNDDKNDILSRFLIE
SEKNPTTLSDEYLRDISLSFIIAGKDTSASTLTWFFYMLCKHSLIQEKIV
LEIKEATGCNYMDSIDEFSLKLTESALEKMHYLHAAITETLRLYPPVPLD
GKCSENDDVLPDGFKIKKGDGVSYMAYPMGRMKYIWGEDAEEFRPERWIH
DGVFQHESPFKFTAFQGGPRICLGKEFAYRQMKIMAAFLVFFFKFQLVDQ
NREATYRIMFTLHMDKGLDLYALPRTKSKY
>NjCYP704C1
MASIHFLSVSISLAVLAVAIYFLFLQIFPRKLVQSKRKYHPIGGTVLNQL
INFSRLHHYMTDLASKYKTYRVLNLFHNEIYTADPVNVEYILKTNFENYG
KGYHNYSILSGLLGDGIFTVDGDKWREQRKISSYEFSTKILRDFSSVIFR
KNVVKLANILSEAATSNQIIDIQDLFMKSTLDSIFKVAFGIELDSMCGSS

EEGAKFSNAFDDASALILKRYVDVTWKIKKALNVGAEAKLKKNVKVIDEF
VVYKLIRGKTEKMQNSQDDSSWKKEDILSRFLQFNDTNPKYLRDIILNFII
AGKDTTATTISWFIYLLCKHPDIQEKVAMEIKKATGNTKTANIDEFMANL
SEEALENMHYLHAALTETLRIYPAVPVDAKVCFSDDIFPDGFSVRKGDMV
AYQPYAMGRMKFIWGDDVLDFKPHRWIDDNGCFRPESPFKFTTFQAGPRI
CLGKEFAYRQMKIFSAVLLGCFVFKLADETKPVNYRTMINLHIDGGLHVR
AFHRY
>NjCYP704C2
MAISMDFLSIAVSTSILLLCLYVYNQQKQGKKTKKHHPILGTMFHQLINF
NRLHDYMTDLATKHKTYRLISPFRYEVYTSDPVNVEYILKTNFENYVKGT
YNHDILRDLLGDGIFTVDGDRWREQRKVSSYEFSTKVLRDFSSVIFRKNA
VKLAHILSQASSNNQILDINDFFMKATLDSIFKVAFGIDLDSMCGSSEEG
VRFSNAFDDASALTLKRYVDPIWKTKKFFNIGSEAKLKENVKVVDEFVYK
LIKTKTQQMHKNQHDFNLKKEDILSRFLQINDTDPKYLRDIILNFIIAGK
DTTSTAMTWFIYMLCKHPEVQDKVAKEIKKATKFINKNVINVEEFANGVS
EEALEKMQYLHAALTETLRLFPAVPVDAKICAKDDVLPDGYNVNKGDMVA
YQPYAMGRMKFIWGDDAHEFKPERWIDENGCFQPESPFKFTAFQAGQRIC
LGREFAYRQMKIFGSILLGCFVFKLSDENKTANYKTMLNLHIDGGLHIRV
SNRFG
>NjCYP706A2
MSKLITTAVSDAWSRLWGSANGNEEVTGTALIIAAILSIFWLVWMLFSKE
TNPPLPPGPRSLPLVGNLLSLDPELHSYFATLAKTYGPISRLWLGKKLGI
LITSPALAREVLKLNDITFANRDVPVAVVEAGYGGNDIVWSPYGDQWRML
RRICVHEMLSNKTLDSVYSLRRKEIRNTVNYLYNRAGSPVNIGEQMFLTV
LNVITGMLWGGTVKAEDRESLGAEFRQVINEMTGYLGMPNLSDFYPGLAR
FDLQGVQKNMKVLTKRFDGIFEMMIAQRRTMGGGDGNKDFLQFLLQREDD
KDSKTPFTMEHLKSLLMDMVVGGTDTTSNTVEFALAEMMNQPEILKKAQQ
ELETVVGKENIVEESHINKLPYLYAIMKEVLRMHPTLPLLVPHCPSESCV
IGGYMIPKGARVFINAWAIHRDPTIWKNPSEFLPERFLTSDWDYSGNDFS
YFPFGSGRRICAGTAMAERMFMLLLASLIHSFDWELGPGEKHDLSEKFGI
VLKKKVALMAIPTPRLSGPTMYE
>NjCYP706A3
MYEKLTTISSSFWSWWPQTGNQNDENTPILLPITLAAIILSWLWWVIVIR
SRTRAPPLPPGPRPLPLLGNLLSLDPDLHTYFASLARSHGPILTLWLGKK
VGIVISSPATAKEILKDHDITFANRDVPVAGKEAAYGGRDIVWNPHGPEW
RMLRKVCVRDMLSNATLDSVYDLRRREIRGTVRFFYNRVGSPVNIGEQMF
LAVLNVITSMLWGGTVEGEERESIGAEFREVVTGMTELLGTPNVSDFYPG
LARFDLQGIQKNMRGLALRFDKIFEKMINQRLKMNGQSESKDFLQVLLEL
KNEGGDTKTPFTMIHLKALLMDMVVGGTDTTSNSVEFAMAELLKKPEIMI
KAQQELEEVVGKDNIVEESHTQKLPYLHLIMKEVLRLHPVLPLLVPHCPS
QSSTISGYTVPKGARVFVNVWAIHRDPLIWESPLEFRPERFLDGKWDYKG
NDFNYFPFGSGKRICAGTAMAERMFLYSLASLIHSFDWKLPQGVKLDVDE
KFGIVLKKKVPLVVIPTPRLASAKLYI
>NjCYP706B1

MISEITSGKSLEITILVVASSLAILFYIWRKSTSRLPPGPFGLPLLGYFP
FLGKELHSDFATLAKRFGPIYTLQLGRRTCIIVNSSDVANVVVHEQDDTF
ANRAPPLVGLLLTYGGKNITWSDNNLYWRNMRKVLVYEVMSNKNMEASLS
FREGGVRKTIKNMYESMGTKVCIGDIAFETSLAVTTSLIWGKSLNQNLGG
VAFRDVIVKIVDLVGLPNISDFFPMLARFDLQGVQGKMYEQVQKLDEILE
KIIEDRMSVKSQESKDGKMDFLQILLDLRQKNSYTIPQIKALLVDIFLAG
TDTSSGMTEWTMAEVLTYPEVMKKIREELEQVVGLKNIVQESHLPKLRYL
DAVIKETFRLHPPLPLLIGRSPSKPCKVGEYIVPKGSTIYMNAWAIHRDP
KYWENPLEFNPNRFINSDGTTKFNYNGSNTNFVPFGSGRRICPGIPVGEK
MLMYLLASLIHSFDWTLPNPKEHDLSDKFGIILRKRNPLIAIPSQRLADK
SLYM
>NjCYP706C1
MAQPNNHDHSWWWEVMISSNKAELTLAISAIILATFCYKWAVSSSFNGSP
PLPPGPRSLPIIGYLPFLDRDLHKQFLKMAQTYGPIFKFKIGSKLHVVIN
SPDLAKVVVREKDEIFANRNSTIAALATSYGGRDIVWSDNNSDWRNLRKI
FVHEVLSNKNLEASRYFRRDEVRKTIKNVFGKIGTSINISEIAFSTEANV
LTSMVWGNTSAEIAEGSNFGTELQMISANIVELIGQANVSDIFPSLAWLD
LLGVERNMKRQLNQLDRVFTRIIEDRIKSNSKKTKDAIGHEEKKDLLQVL
LELMDQNDAGSINLTQLKALISDIMIAGTETSTTLIDWAMAEIMHNNNIM
KRVQKELEEIVGLDNIVEESHLPKLQYLEATIKETFRLHPVVPFILPRSP
SQDCTVGGYTIPKGCTVFLNAWAIHRDPRYWDNPLEFNPERFLLNKFDYI
GSNLNFFPFGSGRRSCPGIPLAEKMQMYILASLLHSFNWSLPEGKEHDLS
EKFGITLKKREPLIAVPSQRLPNECLYM
>NjCYP706D1
MISQVGYRIYLTICTFWLWWWEADNKKDELGRTVLTILVPTLLLLWYKWT
VSYSRQRIIPPLPPGPYGLPIVGFLPFLSSNLHERFTEMAHRYGPIFSLR
LGTKLHVVVNSIDLVKTVANDQDHIFANRRPPITALTITYGGNDIVWSNN
NTYWRHMRKLLVSHVLNSANINACQSFRTHEVRKTVMEVYSKVGTKIDVN
EIAFLTEVNVVTSMLWGRSKSGGGKDAHIIGDGFREVESKIIELIGALNI
SDYLPILSWFDLQGRQREMKRQLEYIDRIFDNIIQQRIDGEVNQDGRKDF
VQIMLELKDRKDAPTSFTVIQIKALLVNIVVAATDSTSTQVEWVMAELLN
NPGVMRKVEDELTEVIGMNIVEESHLPKLTYLEAVIKETFRLHPPLPLLI
QRCPDESCIVGGYTIPKGTIVYMNIWAIHRDPKNWINPLEFEPERFLDGK
WDYNGGNLKFLPFGVGRRICPGIALGDKILMYILASLLHSFNWSLPKDEV
FEISDEFGLVTKKRKPLIAIPSQRLSDVSLYQHH
>NjCYP707A1
MESTTILCSFAFIFSLYLFHSLLKFFCSSDRRRNLPLPPGTMGWPYIGET
FQLYSKNPNVFFASKVKKFGSIFKTHILGCPCVMISSPEAAKFVLVTKSD
IFKPTFPASKERMLGKQAIFFHQGDYHTKLRKLVLRSFMPEAIRSIVPDI
ESIAIDSLKSFEGRMINTFQEMKTFTFNVALLSIFGKDEVLYREDLKRCY
YILEKGYNSMPINLPGTLFHKSMKARKELAQILAKILSMRRESSEDRHDL
LGSFMGDKEGLTDEQIADNIIGVIFAARDTTASVMTWIVKYLAENPSVLQ
AVMKEQEAILKEKEECDNEGNNSKALTWADTKKMPLTTRVIQETLRVASI
LSFTFREAVEDVEFEGYFIPKGWKVLPLFRNIHHSSDNFPNPEKFDPSRF

EVAPKPNTFMPFGNGVHSCPGNELAKLEILVFLHHLTTKYRWSMVGPQNG
IQYAPFALPQNGLPIRLSLRTQS
>NjCYP707A2
MELITVFSCFFILLSTVFLFKSVVASAARRKSLPLPPGTLGWPYIGETFQ
LYSQNPNVFFASKVKKYGSIFKTHVLGCRCVMISSPAAAKLVLVTKSHLF
KPTFPASKERMLGKQAIFFHQGDYHSKLRRLVLRAFTPESIKNIIPDIES
IAVDSLQGWENRLINTFQEMKTFTFNVALLSIFGKDEVLYREDLKRCYYI
LEKGYNSMPINLPGTLFNKSMKARRELAQILAKILLLRRESKKEEHKDLL
ASFMEEKEGLTDEQIADNIIGVIFAARDTTASVLTWIVKYLGENPTVLKA
VTEEQEGIMKAKDDKALTWADTKKMPITSRVIQETLRVASILSFTFREAV
EDVEFEGYLIPKGWKVLPLFRNIHHSPENFTEPEKFDPSRFEVAPKPNTF
MPFGNGTHSCPGNELAKLEILVLIHHMTTKYRWSMVGPQNEIHYAPFALP
QNGLPVRLFPRNSRKEER
>NjCYP707A3
MENSRVFVYVVIFLISLLSYILLRKKRRQVGNKGNLPPGSMGWPYIGETL
QLYSQDPQLFFATKQTRYGEIFKSHILGCPCVMLASPEGARFVLVTHAHL
FKPTYPKSKEVLIGPSAVFFHQGDYHIRLRKLIQGSLSLDSIRKLVPSIE
SIASSAIDSWADSGHVINTFHEMKKLSFEVGILTIFGDLEARYKEELKKN
YSIVEKGYNSFPTNLPGTLYKKALLARKRLSRVLSEIICERKEKKSIDKD
LLGCLLNSKDDKSEILTDDQIADNIIGVLFAAQDTTASALTWILKYLHDH
PKLLEAVKAEQKEIYQFNNGGNSHLTWSQTRYMPITHRVILESLRMASII
SFTFREAVADVEYKGYLIPKGWKVMPLFRNLHHNPEFFMNPEKFDPSRFE
VAPKPNTFMPFGSGVHACPGNELAKLEMLIMTHHLVNKLRWEVVGSESGI
QYGPFPVPLNGLPAKFWTA
>NjCYP710A11
MDFYYSVWPYFFSLLIFILLMEQISYLKKKRSLPGPSFVLPFLGSAVALV
RNPTAFWDNQSLLATSSPHGISTNYIIGRFILFVRSTDLSHKIFANVRPD
AFHLVGHPFGKKLFGDHNLIYMTGQQHKDLRRRIAPNFTPRALSTYTSIQ
QHIILKHLKAWLHLSLSNNNKLIPLRFLCRDMNLETSQTVFVGPYLKNES
RDSFNSDYNFFNVGLMKLPIDLPGFAFRNARLAVSRLVETLAACADESKS
KMKEGHVATCLIDFWMQENLSEIAATGDGATTELPHSSSLEIGGYLFDFL
FAAQDASTSSLLWAVTLLDSHPDVLRKVREEVSGIWSPESDKLITGEQLR
EMRYTEAVAKEVIRYRAPATMVPHIAGKDFQLTENYKIPKGTIVFPSVFE
SSFQGFSEPELFDPDRFMEERQEDRVYKKNFLAFGAGAHQCVGQRYAINH
LVLFIATFTSLIDFKRYRMDGCDEIAYIPTICPKDDCKVTLSKRCTRFPS
LS
>NjCYP711A1
MELMRESLGIIASILTIILGLIIGYFYGPYWSVRRVPGPPLIPFLGHLPL
LANYGPNVFSLLANRYGPIFRFHMGRQPLVIVADAELCRVVGIKKFKDIP
NRSIPSPISASPLHQKGLFFTRDARWSTMRNTILSVYQPSHLAKLIPTMQ
DFIDSASQTFDTKQEDEDIIFSDISLKLATDVIGQAAFGVNFGLSNPIPK
INNNNNDDQVQDFINQHIYSTTMLKMDLSGSFSIILGLLIPILQEPFRQI
LKRMPGTVDSKVDKTNKNLTNRLDEIVANKMNEENRDAGSKDFLSLILKA
RESETESKNIFTPDYISAAAYEHLLAGSATTSFTLSSIVYLVAGHPVVEK

KLLEEIDGFGPVDRIPTAQDLQQKFPYLDQVIKEAMRLYLVSPLVARETS
TQVEIGGYILPKGTWVWLALGVLAKDPKNFPEPDKFKPERFDPDCEEEKK
RHPYANIPFGIGPRSCIGQKFSLQEIKLSVIHLYRRYVFRHSPNMEKPLE
LEYGIVLNFKHGVKVRAIKRL
>NjCYP714A2
MKPQRLRRKLEKQGIRGPKPSFPYGNVVEMQKIQAATIAMNVSRSSGGDF
VGDDYTCTLFPYFEQWRKQYGSIYTYSTGNKQHLYINDPELVKEMNQSIT
LGLGKPSYVTKRLSPLLGNGILRSNGHFWVHQRKIIAPEFFMDKVKGMVG
LMSESVEPLLKKWEACIEGQGGQQADIRVDDDLRAVSADVISRACFGSSY
AKGKEIFSKLRTLQKTISSKGMLFGLPTYGLRKDVKSLEKEIDSLIWEAV
CERKCQETRLLKKDLLQMILEEAMDHFASKDESKHFIVDNCKNIYFAGHE
STSVAASWCLMLLALHPQWQTRISDEMSEACPNGVLDVDSLPKLKSVTMV
IQEAMRLFPPAAFVSREALERTRIGHVDVPKGVCIWSLIPTLHRDPEIWG
PDAHVFRPERFSNGVIKACKSPQAYVPFGVGARSCLGRNFAMAQLKVVIS
LITSKFMFSLSPNYQHSPAYRMVVQPGHGVNIIIQKS
>NjCYP714A3
MKLILLSIFSLLILLSAMMKNFSSLAALLILIVPLILLSPYFLYNLWLKP
NIIRLKLRRQGIKGPKPYFMYGNVPQMQKIQSAAVESANRHHGEFIAHDY
TSTLFPYFEQWRKQYGLIYTYSTGNKQHLYINNAELVKEMNQCITFDLGK
PSYVTKRLAPMLGNGILRSNGHFWAQQRKIVAPEFFMDKVKGMLGLMLES
TEPLMKKWEESIESQGGKMAEIKIEEDLRSVSADVISRTCFGSSYFKGKQ
IFSKLRTLQQTISSGSFLFGLPTFGFLATKNQKEIKGLEKEIESLIWDAV
KERKRECLEKSSSEKDLLQMILEEAINDQCVGAESSKQFIVDNCKNIYFA
GHESTAVAASWSIMLLALHPEWQSRIRKELSQVSNNGILDSDSLSKLKTA
TMVIQEALRLYPPAAFVSREAFEETTIGNIKIPKGVCIWTLIPTLHRDPD
NWGLDANEFKPERFANGVSNACKIPQAYVPFGLGPRLCLGRNFAMVQLKV
VISLIISKFKFSLSPNYKHSPHYKMIVEPGHGVNILIQKI
>NjCYP714B1
MEVPSALKMFMSIFLIALTALFLRLYNVLIKEPKRVRSLLKNQGIKGPEP
RLLIGNLKEFRQPHNGVKGKPTSSLTSTGELNHNWVATLFPCFEQWKKLY
GDLFMFSLGNTHILYVNQTNLVNEITTCTSFDLGKPTYHRKEFGPLLGDG
ILTSNGPTWAYHRKVLAPQLYMEKVKGMMKLFTESIDILVDTWIEKIDSD
GGLSDFDIEPYMRTFSGDVISRACFGSNYVKGKEIFFRLRTLQEATSAKL
MSVGIPGLSHIPTKSNRFAWKLEKEIHNLILQVAAERKENGVEQQDLLQM
VMEGAENGDMTKEVRDHFIVDNCKNIYLAAHEATAIGASWCLMLMAANPE
WQTRVREEVFEITGGRTPDGDMLRKMKQLTMVLQETLRLYPSGPALSRET
LSDMTIGGLKIPKGVNLWTMVVTMHTDTKTWGPDARSFNPERFANGVNSA
CKNPHSFFPFGFGPRICVGQHLAMVELKMLVAALVTNFSFTLSSNYVHSP
ALRLVIKPGYGVNLLMNKL
>NjCYP714B2
MEIQIVIKFFISIGVMGIFGTILRLYNSLVRKPNKLRSILKKQGISGPPP
SFLLGNILEIKRSRDSVLKSSTPLTEPPVYHNCGALLLPFFDPWRQKYGE
KFMFALGNTQILLVTQPDMVRDITTCTSLDLGKPSYQARERGSLLGQGIL
TSNGPYWAHQRKIIAPELYMEKVKGMYNLITESTVTLLNSWKSIIDDNGG

IADIKIDGYMRSFSGDVISRACFGSNFSKGEEIFLRLRALQEAASKKILA
TGIPGMRHLPTKGNREQWALEKEIKELILQIVRERNEVGYEKDLLQMILE
GAKNSNLSEDTIDRFIVDNCKNIYLAGYETTAVAATWCLMLLAANPEWQD
RVRTEAIQICKGKIPDADMVRKMKQLTMVINESLRLYPPVVVVSREALKD
MKFGDINVPQGVNLWTLVTVLHTDPEIWGPDSYKFNPDRFANGITGACKF
PHLYMPFGVGPRVCLGQNLAAVELKILVSLILANFSFTLSPKYVHSPSLN
LVVEPGQGVNLLVKNL
>NjCYP716A1
MEIFYVSLLSVFVIAVSFSLHFLFYKNKSSHAGGKLPPGRTGWPLVGESL
EFLSTGWKGHPEKFIFDRMAKYSPQVFRTSLLGEPAAVFCGAAGNKFLFS
NENKLVRAWWPASVDKVFPSSGQKNSTSQDEAIKMRKMLPNIFKPEALRS
YIGIMDQITERHFAAGWDNKDEVVVFPLAKRFTFWLACRLFLSIEDPNHV
AKFADPFDLLASGLISIPIDLPGTPFRRAINASNYIRKELTVIIKQRKID
LGEGKATPTQDILSHMLLATDEDGKFMTELDIADKILGLLIGGHDTASSA
CTFIVKYLAELPHVYQKVYQEQMEIAKSKAEGELLNWDDVQKMKYSWNVA
CEVLRLAPPLQGAFREATKDFMYSGFSIPKGWKIYWSAMSTHKNAEFFPD
PLKFDPSRFEGSGPAPYSYVPFGGGPRMCPGKEYARLEILVFMYHLVRRF
KWEKIIPEEKIVVNPMPIPEKGLPVRLFSHKA
>NjCYP716A2
MEFLYISLVSLFVLVSASLGFFYKSRSETNQKLPPGRTGWPIVGESFEFL
STGWKGQPEKFIFDRMSKYSPQVFRTSLLGEKAAVLCGAAGNKFLFSNEN
KLVQAWWPASMDKVFPSSNQNQSSSKEEAIKMRKMLPNFFKPEALRGYIG
IMDQITERHFASKWDGKNEVVVFPLAKHFTFWLACRLFVSIEDPNHVEKF
ADPFDLLASGLISIPIDLPGTPFRRAINAANYIRTELILIIKQRKIDLAE
GKATPTQDILSYMLLTSDEDGKFMNELDIADKILGLLIGGHDTASSACTF
IIKYLAELPEVYEKVYNEQMAISKAKGQGELLNWDDIQKMKYSWNVACEV
LRLAPPLQGAFREALSDFMYGGFSIPKGWKLYWSANSTHRNAEFFPEPLK
FDPSRFEGSGPAPYTFVPFGGGPRMCPGKEYARLEILVFMHHLVKRFRWE
KMIPQEKIIVNPMPIPEKGLPVRLYPHKT
>NjCYP716A3
MDIFYASLVSLFVVVVSFSLHFLFYKTKPAIDGKLPPGRTGWPVIGESIE
FLATGWKGHPEKFIFDRMKKFSPQVFRTSLMLEDAAVFCGSAGNKFLFSN
ENKLVQAWWPSSVDKIFPSSNQNSKIEAIKMRKMLPSFFKPEALHRYVPI
MDMVTQKHFQNGWEGKDQIVTYELTKNFTFWLACKIFVSIDDPERVKYLS
GPFESIALGLLSIPVDLPGTPFRRGINSANFIRKELIAIIKQRRSDLADG
KASPTQDILSHMILYSDVDGKFMVDTDIADKILGLLIGGHDTASSACAFI
VKYLAELPEIYEGVYKEQIEIAKSKAPGELLNWEDLSRMKYSWNVACEVL
RLAPPLQGAFREAITDFMYNGYSIPKGWKLYWSANSTHKNPEFFSEPQKF
DPSRFDGKGPAPYTFVPFGGGPRMCPGKEYARLEILVFMHHLVTKFKWEK
VIPDEQIIVNPMPSPAKGLPIRLYPHNA
>NjCYP716A4
MELFLISLFSILLLYLIFFNKRKPTLPPGRTGWPVIGEALDMVSCGRTGQ
PEKFFQDRMQKHSPEIFKTSLAGETLAVFCGPSGNKFLFSNENKLVVSWW
PPAFEKISISTKSSSIVMTKKMRNIIPEFLKPEALQKYIPIMDFTARKQV

EKEWSNGEVKVFPLSRKLTFSLACKLFMSVEDPQLIARIANSFELVSKGI
LSVPIDFPGTAFNRAIKAANMIRKELSPVIKQRKKDLLDKKEVTSDYDLL
FKILKSTDEDGKFLNEMEIADMILGILVASHDTTSTVITFVVYYLADNPH
VFAKVLEEQMEIAKTKGQEELLSWEDIQKMKYSRNVVNEVLRLEPPSQGA
FRQTISDFSYAGFSIPKGWKAFWSVYSTHKDPKYFANPEKFEPSRFEGKG
PLPFTFVPFGGGARMCPGSEFARVEVLVFIHRLVTKFKWEKLIPNEKIVY
QPSPIPVNGLPIRLLPHASSLKP
>NjCYP716B1
METILIIPYIVSILVIFTSLSLIFLKKTTPTTKNLPPGSTGWPLLGENIG
FVLAGPQKFISKRAAKYSPLIFKSSLWGEKMAVFCGPAGNKFLFLNENKF
LTSWWPLSLRKALLYPTYEHKSVKEITAKNMNVLREFLKPEALKRYVPVM
DSMVREHVEKEWKGDIKVFPLAKKYTFVLACRIFLSVKDYDLVKRFGEPF
NDVISGLFSVPLDLPGTAYNRAVRGGAKVRGELLKIIRNKRNELLENKKG
ADEDLLASLLMGTSGSDPVVSEMEISNSIIGLLVASYDTTSAAVTFVFKY
LAELPHIYNEVYKEQMEIANSKPAGEVLNWDDIQKMKYSWNVACEVLRIT
PPVLGAFKQATTDFTYAGFTIPKGWKTFWSTYSTHKNPEYFKNPEEFDPT
RFEGSGPKPYTFVPFGGGPRMCPGKEYARLEILVFMHSVITKFKLQKLSP
NEKVKFHSSPTPMEGLPIRLHPHKK
>NjCYP716B2
MDTLFSSYILPFAFLPLSLSLLFFIQKRYSSKSNNPPGTSGWPMIGENIV
FVMSGPQKFVYDRMRKYSPDVFQTSLLGEKMAVFCGATGNKFLFTNENKL
LTSWWPQSMKKALLFPSFVENNLKEVSALKRSFLHDILKPEALKQYIPLM
DSMARDHLETEWFPFDEVKVFPKLKKYTFDLACKLFMSLDDPEHVNRLAK
HFTLVTSGMFSVPIDLPGTAYNGAIKGGKLVRDELLKIITARKKELMESK
ETAGRDLLSRMLLVTDENGEFMSEMEISNNIIGLLVASYETTSTAVTFVL
KYLAELPHIYSEVFKEQMEICKSKGPNDLLSWEDIQKMKYSWNVACEALR
LTPPAQGAFREAITDFTYAGFTVPKGWKTFWTVHTTHKNPKYFPDPEKFD
PSRFEGTGPAPYTFVPFGGGPRMCPGKEYARLEILVFMYNVVTKFKLEKS
IPDEKIVFHQSPTPMNGLPVRLQLHKK
>NjCYP716C1
MAPIILLIASLLTLLCFFISFRFFTAKKYSNLPPGTSGWPILGESLEFLR
AGKDGAPVNFIKERMDKYGTDLVFKTSILGETMAVFCTPAGNKFLFGNEN
KLVSVWWPASVTKVIGTCLLTSVGEEAKWMRKMLSTFFNPDAFTRLYIST
MELVTRHHFQTHWEGKEELNVYQTVKLYTFELACRLFISLEDPEHIEKLA
TQFNIMVKGIIELPINFPGTRFYRAIRAANAIRTELFAIIKQRKVALEQK
TASPSQDLLSHLLVSCDENGKFLTEEEIVNNFLVLLFAGHDTSSVTITLL
MKKLGELPRVYNKVLQGQFLNIYIERIISREPDSHKPGAKKEVAKMLHLF
CTLVQANQVCVRDLLCLYICFYLYMIIFSESNKFVSNDECGIEQLEIAES
KEAGELLQWEDIQKMKYSWYVVSEVMRLHPPILGTFREALVDFTYAGYTI
PKGWKLYWSAAWTHNDPYFFKNTENFDESRFEGAGPTPFSYVPFGGGPRM
CLGKEFARLEILIFLHIVLKRFRWDLLIPDEKIVYDPMANPINGLPIRLR
PHNF
>NjCYP716D1
MDTLLLVSSIVLPIITIFFLHHFLSEKSTMPGRYGWPLIGESIDYFKKLR

SGTNEKFVMHRRKLFGDVFKTSILGEKMAFLCGPEGNKFLFSNENKLVEV
WWPSSVESIIKKSNNKSVTAESAKVRQLLPPFLRAHAVKNYISDMDSELR
QHLDDYWTGREEVEVCPFVAKYTFALAVKLLLGVHDATELEKLATPFVEA
AGGIIAVPINIPGTRFNRGVKASNRIREVIMGIIAERRKDLADGTATASQ
DLLSHMIVEVDKRNQDSGNAPTTDGDMSSDLLGLLIGGYDTINTTVVFIM
MTLVDHPDVYQGVLKEQMEIAKAKSPGELLDWDDLRKMKYSWNVACEVLR
MRPPTVGAFRVAKTDFTYAGFKVLKGWKLHYIPHYTQKNHEFFPNPEKFD
PSRFEGAGPAPYTFVPFGGGARMCPGNEYARAEILVFMYNIITRYNWERL
IPDEKVVIDPLPRPVHGLPIKLIPHETNA
>NjCYP716E1
MNLIILILITLLSSFFLLKLLKRNNGSKNLPKGSLGFPLIGETFSFLQAQ
MKDKGDDWVQKRVNKYGPIFKTSLLGSPAVIIVGQAGNKFILGSDEDILV
AKQPKTLSAIAGEFALPALTGPRYKLLKGAMVSFLKPESIQNYIKQMDDL
VTTMLLKETLKKDTIETVIFMKKLTFNIAFKILFGIQDEFTRDAFFDDFS
IAFKAVWSLPLNFPGTIFWRGKRARSRIISRILPIIKRKKDDLLKGNLNP
SSDMISALLALQEKNGEIINEQMVLDNFVVLTIAGHDTSAILLSMMVWKL
SRDPEIYKMILKEQMEILKDREGREEKNLRWSDIQKMKLTWRVAQELMRI
IPPVFAFSRRSLKDTSFGGYHIPKGWQVFCVAHGTHMDKEIFENPMEFDP
SRFDNSNTIIPPYTYIPFGAGLHTCIGNEFARVETLITIHRLITMYQWSQ
IHPHEFITRQPMPYPSMGLPIKINPI
>NjCYP720A1
MDSIAWVFTIIITAFFIIFVSKYKRKEFEKKSNNNKNSLPPGRRGWPLIG
DSLNWYNAVASSHPPAFVEQQVQRYGKIFSCSLFGKWAVVSADPTFNRYV
MQNEGKLFKSSYPKSFRELVGINGVITAQGDQQRKLHSIASNMMRLDKLK
FHFLKDIQVVMIRTITNFPDNHVLLLQDVCRKVAINLMVNQLMGVSSESE
VNEMAQLFSDFVDGCLSVPIDLPGFAFHTAMKARANIVSKINKTIETCRL
QEKKEGEEVGNGLLGRLVEEGSLPDDGVADFIINLLFAGNETTAKTMLFA
VYFLTRSPEAMTELLEEQEKLNQRGGEEMLTWQDYKSMPFAQCVIDETLR
LGGIAIWLMREAKEDVPYQDFVIPKGCFVVPFLSAVHLDENLYEEPLSFN
PWRWMDPKNQEKRNWRNSPLYSPFGGGGRLCPGAELSRLQIALFLHYFVT
SYRWAQTKEDKMSFFPSARLVNGFPIQLSRRHSHSHDHTNSSC
>NjCYP720A2
MMVWGYVIPVLVVFIYIAKSVNLKRNKDKLPPGGRGWPVVGDSISWYNSV
ASSHPATFVQQQALRYGKIFSCNIFGRRAVVSIDPIFNRYVMHNEGKLFK
SSYPKSFRDLVGTNGVITAQGDQHRKLHTIASIMMRTDTLKSRFLIDIQL
VMHQTFANLHHNDTILLQDVCRKLAINLMVNQLLGVSSETEVGEMAQLFS
TFVDGCLSLPISFPGFAYHTAMNARNNITRKIKKIIDETRHGVSDGDGGL
VGRLVEEGRLPDDAISDFIINLLFAGNETTAKTMLFAIYFLTTSPQAANK
LLEEHQNIRSKRLDSGGEIDMLTWEDYKSMSFTQNVIDETLRLGGIAIWL
MREAKEDVEYQDYIIPKGSFVVPFLSAVHQDENFYEEATNFNPWRWIDSQ
NQDKRNWRISPFYSPFGGGARLCPGAELARLQIALFLHYFVTSYRWSQVK
EDKMSFFPSARLVNGFPIQLSKLT
>NjCYP722A1
MKMVKLSKELLFFEMKNYNNNIFLASLLSIGITIFFLILKKSTSSTVKVG

VPGRLGLPFVGETISFLFAVNSTKGVYNFVTLRRLRYGKWFKTRLLGKVQ
VFVPTATGAKMIFTNEFMKFNKGYLKSMKNAVGPKSLFCVTHESHKRIRR
LLSDPFSMNSLSGFVPKFDKLLCDRLKKLENDGNSFVVLDFNMKITFDAM
CDMLMSITDASLLTQIENDCAAVSASIISFPFMIPGTTYYKGMKARERLM
ETFEEIIGRRRSGKECHEDFLQTMLKRESYPADEKLDDSEIKDNLLTLII
AGQATTAAAMMWSLKFLDENRTVQDKLREESLAILRNKTNGALLTLEDLN
NMSYASKVVKETLRMSNGVLWFPRRALEDCIVDGVEIKKGWNVNIDATCI
HYDPTIYKDPLQFNPSRFDEIQKPYTYMPFALGARTCLGIDMAKMTMVVY
LQRLTSGYKWKVDDHDPRLGTKTHIPRLRSGLPITLTALENKN
>NjCYP722A2
MVNLQSKEIIFFIKNYHIFLISLLSVAITFFVLRKFKSVKDGTVPGRLGF
PFIGETLSFLSATNSTKGCYDFVNLRRLCRYGKWFKTRLFGKIHVFCPSV
EGAKTIFTNDFKVFNKGYVKSMADAVGPKSLLCIPHESHRRIRRLLSDPF
SMNSLSRFVQKFDKLLSDRLKKLENDRKSFVLLDFNMKITFDAMCDMLMS
ITDVSLLKQIERDCTAVSSAMLSFPVMIPGKTYYKGIKARERLMKTFEET
IARRRSGKECHEDFLQSMLLRDSYPIDEKLDDSEIKDNLLTLIIAGQTTT
AAAMMWSVKFLDENTAVQDKLREEALTILSNKTNGALFTLEDLNSMSYAS
KVVKETLRMSNVLLWFPRIALDDCTIDGFEMKKGWHVNIDSTCIHYDPTL
HKDPFQFNPSRFDVIQKPYTYIPFGSGPRTCLGINMARVTMLVFLQRLTS
GYKWTIDDQDPSLERKAHIPRLRSGCPITLTPLHNGDYIKT
>NjCYP722A3
MVNLQSKEIIFFIKNYHIFLISLLSVAITFFVLRKFKSVKDGTVPGRLGF
PFIGETLSFLSATNSTKGCYDFVNLRRLWYGKWFKTRLFGKIHVFCPSVE
GAKTIFTNDFKVFNKGYVKSMADAVGPKSLLCIPHESHRRIRRLLSDPFS
MNSLSKFVQKFDTLLTHRLNKLQNDRKSFVLLDFNMKITFDAMCDMLMSI
TDVSLLKQIERDCTAVSSAMLSFPVMIPGTTYYKGIKARERLMKTFEETI
ARRRSGKECHEDFLQSMLLRDSYPIDEKLDDSEIKDNLLTLIIAGQTTTA
AAMMWSVKFLDENTAVQDKLREEALTILSNKTNGALFTLEDLNSMSYASK
VVKETLRMSNVLLWFPRIALDDCTIDGFEIKKGWHVNIDSTCIHYDPTLH
KDPFQFNPSRFDVIQKPYTYIPFGSGPRTCLGINMARVTMLVFLQRLTSG
YKWTIDDQDPSLERKAHIPRLRSGCPITLTPLHNIKT
>NjCYP722B1
MEMNMQFLFLPYVSYCYCCFYTTLYIVLVWFVTRIIIMVLWHSDTFPPGN
RGLPIIGETLQFMAAINSNKGFYDFVQIRRLRYGKCFKTKLFGKEQVFIS
STEWAKKILNNDLGKFGKRYIQSIAELVGDQSLLCAHEHHKFIRSRLSHL
LSPPSISSFVIQFDQLIVNTLTSWIHKSTIVIHHEALKITSKAMCKILMS
LEDEEELEMLQKDVDHICEAMLAFPFRFPFTRFYKGLQARKRIMSRLDNM
INERRRLHKQIRYQDLLQHLLTKQKDEAISLTDEQIKDNILTMIIAGQDT
TASAITWMIKYLDENQDVLNTLRAEQLSLAERISTNSYLTLEDLNEMSYA
SKVVKESLRMASVVPWFPRLALHDCDIQGYQIKKGWNINIDARSVHVDPN
VYDNPDKFIPSRFDDESKAYNFLAFGMGGRTCLGMNLARAMMLIFVHRLV
ITYRWRVTNTDSSIEKWALFSRLKNGCPIQITPLMKEGDIDSTN
>NjCYP722C1
MEGIPGSLGWPIVGESFSFISEFSSPSGIFSFMHKRQQRYGKVFKSYVLG

RFTVFMTGREASKILLTGKDGIVSLNLFYTGQQVLGPTSLLQQTGEQHKR
LRRLIAEPLSLDGLKKYFNFINNLAIQTLDQWSGRQIFVLEEASTFTLKV
IGSMMISLEPSGEDQEKFRENFKIISSSFASLPFKIPGTAFHNGIKARDR
MYAMLDSIISERRNGEGFEQDFLESLIKKKSKKASEGEENKINNNNNNDD
KLTDKQLKDNILTLLVAGHDTTTAALTWLIKFLHENPLVLQRLREEHMEI
RDNRKDGTTLTWSEVMNMPYTTKVISETLRRATILPWFSRKAAQDFEIDG
YKIKKGWSINLDVVSIHHDPEVFVDPYKFDPTRFDEHIKPYNFLGFGNGP
RMCPGLNLAKVEICVFIHHLVCRYKWTPLEKDDSVQPTLVRMPKNKYPVL
VEPL
>NjCYP734A1
MEEKILYGLKLFVGSFILLVFVLKIVVLLWWKPRKIEEHFAKQGIKGPPY
RFFIGNAKELVSLMLKASSKPMPFSHNILPRVLSFYFHWKKIYGPTFLVW
FGPTVRLTVADPDLIREIFSSKSEFYEKNEAHPLIKQLEGDGLLSLKGEK
WAHHRKIITPTFHMENLKLLMPVAAESLIGMLDKWEEKPNSDEVEIEVSD
WFKTLTEDIVARAAFGRSYEDGKAIFRLQAEQMVLASEAFQKIFIPGYRF
IPTKRNIRSWKLEREIKKSLMRVIEQRRDKWDDEVLENGPKDLLGLMIKA
STKKTAPEFSSPATSSETTITVRDIAEECKSFFFAGEQTTSNLLTWTTVL
LAMHPRWQQLARDEVIKLCGTRGVPTKDDVVKFKTLSMILNESLRLYPPI
VATIRRAKADVDLGGCKVPRGTELLIPILAVHHDHNIWGSDAHEFNPARF
SDGVARAAKHPIGFIPFGLGVRMCIGQNLAILQAKLTLAIILQRFSFRLA
PQYQHAPTVLMLLYPQYGAPVLFRRLSNPKDDQD
>NjCYP734A2
MEDDMMFLKWWLKVVLIFLIVMVFGVKIFVLLWWKPRKIEEHFAKQGIRG
PPYCFFIGNAKELVSLMVKASSKPMPFSHNILPRVLSFYHHWKKIYGPTF
LVWFGPTVRLTVAEPDLIREIFSSKSEFYEKNEAHPLIKQLEGDGLLSLK
GEKWALHRKIITPTFHMENLKLLIPMATSSVVRMLEKWLDMSNSDEVEIE
VSQWYQNLTEETVTRTAFGSSYEDGKHIFQLQAQQMVLASEAFQKVTIPG
FRFLPTKRNRESWRLEREIKKSLMRVIERRRENWDKEGMENGPKDLLGLM
IQASTKESVNSSPATAAAITARDIAEECKSFFFAGEQTTSNLLTWTTVLL
AMHPQWQVIARDEVLKVCGPRDIPTKDDVSKLKTLTMILNESLRLYPPIV
ASIRRAKADVQLGGCKVPRGTELLIPILAVHHDQAIWGNDANEFNPSRFS
DGVARAAKHPVSFIPFGIGVRTCIGQNLAMLQAKLTLAIILQRFSFRLSP
KYQHAPTVLMLLYPQYGAPIIFKSLSTDSSIKEDQGL
>NjCYP734B1
MHLLLVLILLPLLIYLSKFINKIIWVPLKIQTHFRQQGITGPIYRPIFFF
GNQAQIRRRMIAEAESTTMVDHDIVKRVMPHYHNWSTVYGKTFLYWFGST
PRLGISEPDMVKEVLLNSGGGGGCFGKIEFNPLSRLLIGDGLVGLNGHKW
ALHRRITSQAFNMERIKDWVPEIVSSTSKMLGKWKDKCEEFELDVHKEVH
ILSADIISRIAFGSNFEEGKHIFELQQQQISLVLQALRSVYIPGFKFLPT
KKNRMRWKLESETRNSIKKLIEMNKTREKNPKALLTLLMSPYKNQENQEE
RLSVEEIIDECKTFYFAGKETNANHLTWAILLLALNQDWQQKARQEISHV
CKDNQEILTAQNLPELKIISMILNETLRLYPPAVMLMRQTLKNVKLGNLN
IPAKTQLYLAMTAIHHDTEIWGEDANLFNPERFANPKKHLGSFFPFGLGP
RICVGQNLAVVEAKIVLAMIIRRYSFVVSPLYVHAPMQALTLQPQYGAHI

IFSRISS
>NjCYP735A3
MGTVILLGLLGVCIYFLVRIAYDFVSCYWLTPRRIKMIMEKQGIRGPKPR
FLVGNILDMAALVSKSTSSDMDSINHDIVGRLLPHLLLWSKLYGKRFVYW
NGIEPRMCLTETELIKELLIKNSMVSGKSWLQQQGSKHFIGRGLLMANGH
DWYHQRHIVAPAFIGDKLKSYAGYMVDCTDKLIGSLEEEIGSGRVEFEIG
EYMARLTADIISRTEFDSSYEKGKQIFDLLTLLQNLCAQASRHLCLPGSR
FLPSKYNREIKSLKMEVERLLMEIIQSRKDCVEIGRSSSYGNDLLGMLLN
EMQKKRNNGFSLNLQLIMDECKTFFFAGHETTALLLTWTVMLLASNPTWQ
QKVRDEVKRVCNGGHPTVDHLSKLTTLNMVINESLRLYPPASLLPRMAFE
DIKLGDLNIPKGLSIWIPVLAIHHSEEIWGKDVNEFNPDRFATKSFAPGR
HFIPFAAGPRNCVGQSFAMMEAKIILSMLISRFSFTISDHYRHAPIIVLT
IKPKYGVQICLKPLNHL
>NjCYP736A222
MFPLILAVALSLIGSLWMIHYRRIRKLPPGPSGLPILGCLHLLGSLPHIA
LQRLATKYGHIMSLRLGSKLTIVVSSPRAAELFLKTHDSVFASRPKVQSA
EIFFYGAKGMALTPYGAYWRSVRKFCTLQLLSPAKIDELAGMRSGELGSL
VESLKEAALARDVVDVSEKIITLIEDVVYKMLFGNIKNENFDIKGCIVEY
MHLTGAFNLSDYVPFLRPFDLQGLNKRMKATAKTIDEMLEIMIDEHEQNA
RMGTQKRDLDFIDVMLSQKNESIDSHDESSYIIDRTNIKAVVLDMIAGTL
DTSYTAIEWILSELIRNPRVTKKLQEELNAFLGIREIVEETDLSNFKYLD
MVVKETLRLHPVAPLLVPHESMKDTVIEGFHIPKESRIYINSWAIGRDPS
IWSENAEEFYPERFIDNKIDLRGKDFELLPFGSGRRGCPGMYLGLIHIRL
VVAQLVHCFEWELPNATSAGDLDMSEKFGITLPRANHLFAVPTYRL
>NjCYP736A223
MSPLIVAALCILLVTICLLRCRKTTHKLPPPGPSGLPIIGNLHMLGSLPH
RSLTEFSKKYGPIMSLRLGKVLTVVVSSAQAAELFLKTHDDVFASRPKAQ
ASEYVSYGNMAMALRPYGTYWRNVRRFCTLELLSVKKIDCFAGMRREELG
FLVEDVKEAAAAREAVDLSEKVAGLIENMTCRMIFGRCKYDKLNLKSIIG
EMFDLIGAFNVADYLPWLAPLDLQGLTRRMKKASKSFDKILEPIIQEHEQ
NPKDESSTQDDFIDIMLSMKNKSTEKNDDDNSFTVDRTNMKAIVLDMIAG
ATDTSHVSIQWVLSELIKNPRTMKRLQEELKNVVGPNKMVEESDLPNLNY
LTMVVKEGLRLHPVAPLLVPHESLEDIVINGYYIPKNSRVIINFWAIGRD
PHAWSDNAEEFLPERFIGSDIDLRGRDFKLIPFGSGRRGCPGINLGLINI
RLVVAQLVHCFDWELPNGMSPADLDMTEVFGLTVPRAKHLLAVPTYRL
>NjCYP736A224
MSPLIFAALCILLVTICLLRRRKTTHKPPPPGPSGLPIIGNLHMLGSLPH
RSLAEFSKTYGPIMSLRLGKVLTVVVSSAQAAELFLKTHDDVFASRPKAQ
ASEYLSYGNMAMAFRPYGAYWRNVRRFCTLELLSVKKIEGFAGMRREELG
FLVEDVKAAAAAREAVDLSEKVAGLIENMSCRMIFGRCQYDKLNLKSIIG
EMFDLFGAFNVADYVPWLAPLDLQGLTRRMKTASKTFDKILEPIIQEHEQ
NPNDESSTQDDFIDIMLSMKNKSSEKNDDDNSLTIDRTNMKAIVLDMIAA
SIDTSHVSIQWVLSELIKNPRTMKRLQEELKNVVGPNKMVEESDLPNLNY
LTMVVKEGLRLHPVAPLLVPHESLEDIVINGYYIPKNSRVIINCWAIGRD

PHAWSDNAEEFLPERFIGSDIDLRGRDFKLIPFGSGRRGCPGINLGLINI
RLVVAQLVHCFDWELPNGMSPADLDMTEVFGLAVPRAKHLLAVPTYRL
>NjCYP736A225
MSPQIFAALCILVVTICLLRRKTTHKPPPPGPSGLPIIGNLHMLGSLPHR
TLAEFSKKYGPIMSLRLGKVLTVVVSSAQAAELFLKTHDDVFASRPKAQS
SEYLSYGNMAMAFRPYGAYWRNVRRFCTLELLSVKKIEGFAGMRREELGF
LVEDVKAAAAAREAVDLSEKVAGLIENMSCRMIFGRCKYDKLNLKSIIGE
MFDLVGAFNVADYVPWLAPLDLQGLTRRMKTASKTFDKILEPIIQEHEQN
PNDESSTQDDFIDIMLSMKNKSSEKNDDDNSLTIDRTNMKAIVLDMIAAS
IDTSHVSIQWVLSELIKNPRTMKRLQEELKNVVGPNKMVEESDLPNLNYL
TMVVKEGLRLHPVAPLLVPHESLEDIVINGYYIPKNSRVIINCWAIGRDP
HAWSDNAEEFLPERFIGSDIDLRGRDFKLIPFGSGRRGCPGINLGLINIR
LVVAQLVHCFDWELPNGMSPADLDMTEVFGLAVPRAKHLLAVPTYRL
>NjCYP749A15
MVTLLVFNCLGLYFFVVLVKFLHRVWWTPLNVKRLMEKQGIKGPSYKFIH
GNKKEISSMIHNSIAQPMDISHHIFPRLQPHIYSWIQIYGMSFIHWNGPI
AELLVTEPELIKEILNNKDQIYSKPEVESYMKKIVGDGIFTTEGKKWSKL
RKFANHSFHAESLKNMVPAMVESADMMLKKWISYEGKEIEVSEEFRLLTS
EVISRTAFGSSYLQGEHIFQMLRKLTIIAARNAYTTSFPGLSFVKNEDDV
ESDKLEKRIGDLFLKIVDKKEKAMREKVEKSETDFLGLLVNAKNDSDENN
RISIENIIDECKTFYAAGHGTTTLLLSWMILLLSIHTDWQDKARQEVADV
LGDQLPNPEAITRLKIINIIIHETLRLYPPGSGIVRKVMREAKLGNLRLA
ANMNVQIPVLALHHDPKLWGEDSHIFKPERFSQGIAQATNHNPSIYLPFG
FGPRVCVGFNFAINEAKITLAMILQRYKFTLSPNYIHSPILVMTVRPKNG
VQIMLHKI
>NjCYP749A16
MVIYIAPFQEKSMRAQGIKGPSYKFLHGSTTEMTHLRKQALSNPMDLSHD
IFPRILPHFHSWFNTYGKNFIYWIGYRGQLVVTETELIKEILNNKDKTYP
KSETEGYIKKLVGEGLVTIQDGEKWRKLRKLANHAFHGEGLKNMVPSMIA
SVEVMMERWKEYEGKEIEVFEEFRVLTSEVISRTAFGSSYEQGKDIFDML
GKLSLMVARNSYKIRIPGISRIWKSSDELESEKLEQRMRESIVGIIEERD
KGWNGEEAEKLGNDFLGMLVKAKRDDDVDNKISIQDIIDECKTFYVAGHE
TTTSLLSWTVLLLAMYTDWQEKARKEVVDLFGNENPYSDGIPRLKTMNMI
INESLRLYPPVISLLRKVKQETKLGKLVVPKNLNFTIPVLALHHDTQIWG
EDAQRFNPDRFSQGVAKATNNNPSVFLPFGLGPRSCVGLNFATNEAKIAL
AMILQRYSFTLSSTYTHSPFQVLTIRPQHGVQIILHPM
>NjCYP749A17
MKSQGLKGPSYKFLHGNTKEILEFRKESTSSPMDLSHDVFSRIQPHIYSW
IKLYGNNIISWYGPQPVLIITEPELIKEVLSNKDGFYPKPKLQNYMKKLV
GDGLVASEGEKWSKLRKLANHTFHAESLKEMVPAMIASVEAMLERWKENE
GKEIEVCEEFRLLTSEVISRTAFGSSYLEGKHIFEMLTKLGYIISRNDFK
IRFPGSGFFFKSSDDIESDETKQAIHNSVMEIIEKREKRATMEGEKERFG
SDFLGLLIRAKHESNDSMRISVNDIIDECKTFYIAGQETTTTLLSWTILL
LAIHTDWQEKARNEVLQNPNSEGIARLKNMNMIINETLRLYNPVVNLIRR

VKERVKLGKYDLPANVDVLIPPLALHLNPKIWGKDAHLFRPERFEYGVGK
ATNNNPTAFLPFGFGPRTCVGLNFAITEAKIALSMILQRYKFTLSPTYIH
SPVQILTIRPQHGVQILLQEF
>NjCYP749A21
MVGLLTILISSSLFLFLLSVLIKFVHKVWWVPIQIQKSMRAQGIKGPSYI
FLHGSTTEMAHLRKQALTKPMDFSHDIFPRILPHLNSWFNTYGKNIMYWF
GYRGQLVITETEHIKEILNNKEKNYSKRESEGFAKKLLGNGLVTIQDGEK
WFKLRKLANHAFHAEGLKNMVPSMIASVEVMMERWKEYEGKEIEVFEEFR
VLTSEVISRTAFGSSYEQGKDIFDMLGKLSLMVARNSYKIRIPGISRIWK
SSDELESEKLEQRMRESIVGIIEERDKGWNGEEAEKLGNDFLGMLVKAKR
DDDVDNKISIQDIIDECKTFYVAGHETTTSLLSWTVLLLAMYTDWQEKAR
KEVVDLFGNENPYSDGIPRLKTMNMIINESLRLYPPVISLLRKVKQETKL
GKLVVPKNLNFTIPVLALHHDTQIWGEDAQKFNPDRFSQGVAKATNNNPS
VFIPFGLGPRSCVGLNFATNEAKIALAMILQRYSFTLSSTYTHSPFQVLT
IRPQHGVQIILHPM
>NjCYP749A22
MVALLTILISSSLFLFLLSVLIKFVHKVWWVPIQIQKSMRAQGIKGPSYK
FLHGSTTEMAHLRKQALTKPMDFSHDIFPRILPHLNSWFNTYGKNIMYWF
GYRGQLVITETEHIKEILNNKEKNYSKRESEGFAKKLLGNGLVTIQDGEK
WFKLRKLANHAFHAEGLKNMVPSMIASVEVMMERWKEYEGKEIEVFEEFR
VLTSEVISRTAFGSSYEQGKDIFDMLGKLSLMLARNSYKIRIPGISRIWK
SSDELESEKLEQRMRESIVGIIEERDKGWNGEEAEKLGNDFLGMLVKAKR
DDDVDNKISIQDIIDECKTFYVAGHETTTSLLSWTVLLLAMYTDWQEKAR
KEVVDLFGNENPYSDGIPRLKTMNMIINESLRLYPPVISLLRKVKQETKL
GKLVVPKNLNFTIPVLALHHDTQIWGEDAQRFNPDRFSQGVAKATNNNPS
VFLPFGLGPRSCVGLNFATNEAKIALAMILQRYSFTLSSTYTHSPFQVLT
IRPQHGVQIILHPM
