# Supplementary material for: Metabolome and transcriptome associated analysis of sesquiterpenoid metabolism in Nardostachys jatamansi
Source: Front Plant Sci. 2022 Nov 29;13:1041321. doi: 10.3389/fpls.2022.1041321 (PMC9746346; doi:10.3389/fpls.2022.1041321)
Supplement: Additional file 1 — This file includes all additional tables ( Tables S1 - S8 ) used in this manuscript. Table numbers and titles were listed as follows: [file DataSheet_1.zip › Data Sheet 5.pdf]

>*NjActin*

GGGAAACTCCAACAAGCTTAGCTCAGCTATGGACAACAAAAACGTCGTCGTTTGCGA
CAATGGCACCGGGTATGTCAAATGTGGTTTTGCCGGAGAGAATTTTCCTACCTCTGTAT
TCCCTTGTGTGGTTGGAAGGCCTATGCTTCGATACGAAGAATCCCTCATGGAACAAGA
ACTGAAGGATATTGTTGTTGGAGAGTCATGTTTAAACTTGAGACATCAACTCGATATTT
CTTACCCTGTCAATAACGGCATCGTTCAAAATTGGGATGATATGGGTCATGTATGGGAC
CATGCGTTTTACAATGAACTAAAAGTAGATCCGACATCATGTAAAATTTTGCTGACAGA
CCCGCCACTGAATCCATCCAAGAATCGCGAAAAGATGGTTGAGACAATGTTTGAGAAG
TATAACTTTGCTGGTGTCTTCATCCAAATTCAAGCTGTCTTAACTTTGTATGCTCAAGGT
CTACTCACTGGGCTAGTAATCGACTCTGGTGATGGCGTGACTCATGTGGTTCCAGTTGT
CGATGGCTACTCTTTCCCTCATCTTACAAAAAGAATGAATGTAGCAGGCCGACACATAA
CGTCATACCTTGTTGATTTACTTTTACGAAGGGGGTATGCAATGAATAGGACCGCCGATT
TTGAGACTGTTAGGGATATTAAAGAGAAACTGTGCTACATAAGTTATGATTACAAAAGG
GAATATCAGTTAGGACTGGAGACCACAATCCTTGTAAAGAACTATACTTTGCCTGATGG
AAGGGTCTTAAAAGTTGGCACTGAAAGATTTCAAGCCCCTGAAGCTCTCTTTACTCCT
GAACTCATAGATGTTGAAGGGGATGGAATGGCTGACATGGTTTTTCGATGCATACAAGA
GATGGATATTGACAATCGAATGACGCTGTACCAACATATAGTGTTGAGTGGAGGGAGTA
CAATGTATCCCGGATTACCTAGCCGGCTAGAGAAAGAAATTTCAGATCGTTATCTTGAC
GTTGTTTTGAAAGGAAACAAAGATGGATTAAAGAAACTGCGGTTGAGGATAGAGGATC
CACCGCGAAGAAAACACATGGTTTACCTCGGAGGTGCAGTTCTTGCTGGAATAATGAA
GGATGCACCTGAGTTTTGGATAAGCAGAGAAGAATATTTGGAAGAAGGAGTTGGGTGT
TTAAGCAAGTGCGGCTCTGCGTGATTTTTGAATTTATGCTTTTTTTTTTCTTTTTACAGT
TTGTTTTCTACTTTGTTTCTTGATCATTTGTACTCTGTTTCTTGATCATGTGTACTCTGTTT
CTTGATCATGATAATGACTGGAATTTGAATCAAAAAAAAAAAAAAAAAAAAAAAAAA
AAAAAAAAAAAAGT
